# Comparative safety evaluation of pentavalent (DTaP-IPV-Hib) and hexavalent (DTaP-IPV-Hib-HepB) vaccines in infants: a real-world analysis based on VAERS

**DOI:** 10.3389/fcimb.2025.1666509

**Published:** 2025-10-30

**Authors:** Zhen Wei, Bai Li, Hong-Ling Jia

**Affiliations:** ^1^ School of Acupuncture and Tuina, Shandong University of Traditional Chinese Medicine, Jinan, Shandong, China; ^2^ Second School of Clinical Medicine, Shandong University of Traditional Chinese Medicine, Jinan, Shandong, China; ^3^ Department of Acupuncture, Second Affiliated Hospital of Shandong University of Traditional Chinese Medicine, Jinan, Shandong, China

**Keywords:** combination vaccines safety, pentavalent vaccine, hexavalent vaccine, disproportionality analysis, multivariable logistic regression, VAERS

## Abstract

**Background:**

Combination vaccines simplify immunization schedules and improve compliance, making them a global priority in pediatric immunization strategies. The DTaP-IPV-Hib pentavalent vaccine has been widely adopted, and with the incorporation of the hepatitis B vaccine (HepB), the DTaP-IPV-Hib-HepB hexavalent vaccine was developed. However, whether the addition of antigens in the hexavalent formulation is linked to differences in the reporting of adverse events following immunization (AEFIs) remains a matter of ongoing debate.

**Objective:**

This study aims to compare the safety profiles and differences in AEFIs between the pentavalent vaccine and the hexavalent vaccine in infants aged 6 weeks to 2 years, based on real-world data from the U.S. Vaccine Adverse Event Reporting System (VAERS). The study also seeks to identify potential safety signals and evaluate correlates of death classification among reports.

**Methods:**

AEFIs reported to the VAERS from 2018 to 2024 were analyzed. Four disproportionality analysis methods—including Reporting Odds Ratio (ROR), Proportional Reporting Ratio (PRR), Bayesian Confidence Propagation Neural Network (BCPNN), and Multi-Item Gamma Poisson Shrinker (MGPS)—were used to identify potential safety signals. A multivariable logistic regression model was employed to examine factors associated with reports classified as death.

**Results:**

A total of 4,980 AEFI reports were included (3,259 for the pentavalent vaccine and 1,720 for the hexavalent vaccine). Reports following hexavalent vaccination more frequently involved serious AEFIs—particularly hospitalization and life-threatening events—than reports following pentavalent vaccination, especially among infants aged 6 weeks to 4 months, in whom apnea and cyanosis were more frequently reported. Disproportionality analysis showed that reports for the hexavalent vaccine generated stronger disproportionality signals in multiple systems, including nervous system disorders (ROR = 1.95; IC025 = 0.70), vascular disorders (ROR = 2.89; IC025 = 1.17), cardiac disorders (ROR = 1.92; IC025 = 0.45), and respiratory disorders (ROR = 1.33; IC025 = 0.19). In the multivariable model, increasing age and female sex were associated with lower odds of reports being classified as death. Co-administration with other vaccines was associated with higher odds of death classification in the pentavalent subset, with no clear association observed in the hexavalent subset.

**Conclusions:**

While reports for both vaccines were generally consistent with known safety profiles, those following hexavalent vaccination showed stronger disproportionality signals in younger infants. These findings are hypothesis-generating and highlight the importance of targeted post-vaccination monitoring; they do not establish causality.

## Introduction

Vaccination is one of the most effective public health interventions for reducing the incidence and mortality of infectious diseases in infants and young children (Nandi and Shet, 2020). To enhance vaccination efficiency, simplify immunization schedules, and improve compliance and coverage, the development and implementation of combination vaccines has become a key focus of global immunization strategies (Elliman and Bedford, 2003; Marshall et al., 2007). The Advisory Committee on Immunization Practices (ACIP) in the United States also recommends the preferential use of combination vaccines whenever feasible (National Center for Immunization and Respiratory Diseases General recommendations on immunization, 2011). Among the infectious diseases that pose serious threats to children’s health, common examples include diphtheria, poliomyelitis, pertussis, Haemophilus influenzae type b (Hib) infection, and tetanus. Traditional immunization strategies require separate administration of the Hib vaccine, inactivated poliovirus vaccine (IPV), and diphtheria-tetanus-acellular pertussis (DTaP) vaccine (Centers for Disease Control and Prevention, 2024). To achieve full immunization, infants under two years of age may need to receive up to 12 doses in total. The diphtheria-tetanus-acellular pertussis–inactivated poliovirus–Haemophilus influenzae type b combination vaccine (DTaP-IPV-Hib), widely used as a routine vaccine in pediatric immunization programs, provides simultaneous protection against five major infectious diseases. By consolidating the original 12-dose schedule into just four doses, it significantly reduces the number of injections, saves time, and improves parental and recipient compliance as well as the timeliness of vaccination (Centers for Disease Control and Prevention, 2008).

With the continuous advancement of vaccine technology and the ongoing optimization of immunization strategies, the hepatitis B vaccine (HepB) has been incorporated into the combination formulation, resulting in the development of a hexavalent vaccine (DTaP-IPV-Hib-HepB) (Oliver and Moore, 2020). This hexavalent vaccine has now been approved for routine immunization of infants and young children in numerous countries and regions. The promotion of this combination vaccine further simplifies the immunization process, reduces the complexity of vaccination schedules and parental hesitancy, and improves vaccination coverage. It aligns with the modern public health concept of “fewer injections, broader protection (Government of Canada, 2025).” Hepatitis B virus (HBV) is transmitted through blood and bodily fluids and can cause both acute and chronic hepatitis, with potential progression to liver cirrhosis and hepatocellular carcinoma. Since infection during infancy is more likely to result in chronic carrier status, the World Health Organization (WHO) recommends that all newborns receive the HepB vaccine as soon as possible after birth (World Health Organization, 2017). The introduction of the hexavalent vaccine, which retains all the protective functions of the pentavalent vaccine while additionally targeting hepatitis B prevention, offers an integrated and optimized immunization strategy (Icardi et al., 2020). However, whether the increased number of vaccine components may be linked to differences in the reporting of adverse events following immunization (AEFIs) remains a concern that warrants close attention.

Previous studies have demonstrated that both pentavalent and hexavalent vaccines exhibit good immunogenicity and overall safety (Marshall et al., 2015; Hansen et al., 2016); however, differences have been reported in both the frequency and types of adverse reactions reported. Some reports indicate that local or systemic immune responses—such as fever and injection site redness—occur slightly more frequently following administration of the hexavalent vaccine, which may be related to variations in antigen load or adjuvant composition (Marshall et al., 2015; Block et al., 2017). Nevertheless, most of these studies are based on clinical trials or controlled populations and lack systematic evaluations from large real-world datasets. Therefore, conducting large-scale, adverse event database–driven real-world analyses is of great importance to validate safety differences between the two vaccines.

The Vaccine Adverse Event Reporting System (VAERS), jointly managed by the U.S. Food and Drug Administration (FDA) and the Centers for Disease Control and Prevention (CDC), serves as a national surveillance platform for monitoring vaccine safety and provides important insights into post-marketing safety profiles (Shimabukuro et al., 2015). In this study, we utilized the VAERS database to collect AEFIs reports associated with pentavalent and hexavalent vaccines administered to infants aged 6 weeks to 2 years between 2018 and 2024. Multiple disproportionality analysis methods were employed to detect potential safety signals and compare the types of AEFIs between the two vaccines. To account for potential confounding factors such as age and sex, multivariable logistic regression was also conducted for adjustment. The reporting patterns identified in this study may offer signal-based insights for the clinical use, safety monitoring, and immunization policy optimization of combination vaccines.

## Methods

### Data source

The data for this study were obtained from the Vaccine Adverse Event Reporting System (VAERS), a national passive surveillance system co-managed by the U.S. Centers for Disease Control and Prevention (CDC) and the Food and Drug Administration (FDA). VAERS is designed to monitor vaccine safety and generate hypotheses by collecting spontaneous, voluntary reports of adverse events following immunization (AEFIs) from healthcare providers, manufacturers, vaccine recipients, and the public. The database encompasses a wide range of data, including patient demographics, vaccination details (date, type, lot number), AEFI onset timing, unstructured clinical descriptions, medical history, and concomitant medications. All reported AEFIs are standardized and coded using the Medical Dictionary for Regulatory Activities (MedDRA), version 27.0, meaning each report can be associated with multiple System Organ Classes (SOCs) and Preferred Terms (PTs) (Brown, 2004; Shimabukuro et al., 2015).

It is critical to note that as a passive surveillance system, VAERS has inherent limitations. It relies on voluntary reporting and lacks denominator data (i.e., the total number of doses administered). Consequently, our analysis focuses on describing the frequency and characteristics of reported AEFIs and identifying disproportionality signals; it does not allow for the calculation of incidence rates or conclusions about the relative incidence between vaccines. All reports are de-identified to protect privacy, and this study was granted an exemption from ethical review by the Human Research Ethics Committee of the University of Adelaide.

### Data selection

This study extracted relevant reports from the VAERS database involving administration of the pentavalent vaccine (DTaP-IPV-Hib) and the hexavalent vaccine (DTaP-IPV-Hib-HepB) to infants aged 6 weeks to 2 years between January 1, 2018, and December 31, 2024. Reports were identified based on the “VAX_NAME” field, with “DTAP + IPV + HIB” indicating the pentavalent vaccine and “DTAP + IPV + HEPB + HIB” indicating the hexavalent vaccine. Reports were then filtered to include only those in which the patient’s age was within the specified range. Duplicate entries and reports related to vaccination errors without any described AEFIs (e.g., incomplete vaccination schedules) were excluded. The resulting dataset served as the basis for the AEFI analysis.

### Statistical analysis

In this study, four widely used disproportionality analysis methods were employed to evaluate potential associations between vaccines and AEFIs: Reporting Odds Ratio (ROR), Proportional Reporting Ratio (PRR), Bayesian Confidence Propagation Neural Network (BCPNN), and Multi-Item Gamma Poisson Shrinker (MGPS). The ROR was selected as the primary method because of its well-established application in pharmacovigilance and its effectiveness in analyzing large-scale spontaneous reporting data (Rothman et al., 2004). PRR does not rely on external data and is well-suited for the rapid identification of both known and potential safety signals (Evans et al., 2001). BCPNN estimates the Information Component (IC) using Bayesian posterior probabilities, making it suitable for rare events and capable of providing uncertainty intervals (Bate et al., 1998). MGPS applies a Bayesian shrinkage mechanism, offering advantages in false positive control when analyzing multiple vaccine-event combinations (Dumouchel, 1999).To address potential confounding and bias, stratified analyses were conducted by age and sex. All four methods were calculated using 2×2 contingency tables, with detailed formulas and signal detection thresholds provided in **Supplementary Tables 1** and **2**. Signal detection thresholds were established based on literature reviews and empirical evidence to balance sensitivity and specificity. For PRR, a threshold of PRR ≥ 2 with N ≥ 3 was adopted, in line with guidelines from the European Medicines Agency (Evans et al., 2001). For ROR, a signal was defined as ROR ≥ 3 with a 95% confidence interval lower bound exceeding 1, improving specificity and reducing false positives (van Puijenbroek et al., 2002). BCPNN considered IC025 (the lower 2.5 percentile of the IC) > 0 as a signal, striking a balance between sensitivity and robustness (Bate et al., 1998). For MGPS, a conservative threshold of EB05 (the empirical Bayes 5th percentile) > 2 was used to mitigate the risk of false signals due to multiple comparisons (Hauben et al., 2005).The robustness of these thresholds was validated by comparison with known AEFIs, sensitivity analyses, and systematic performance evaluations using positive and negative controls, ensuring the scientific rigor and reliability of signal detection.

To further explore correlates of severe report classifications, a multivariable logistic regression model was constructed, incorporating age, sex, and vaccination co-administration status (whether the vaccine was administered alone or co-administered with other vaccines) as independent variables. Age was categorized into three groups: 6 weeks to 4 months, 4 to 8 months, and 8 months to 2 years. The outcome was whether a VAERS report was classified as death; results are interpreted as odds that a report received this classification, not as risk. A p-value < 0.05 was considered statistically significant.

## Results

### Baseline characteristics

**Figure 1:**
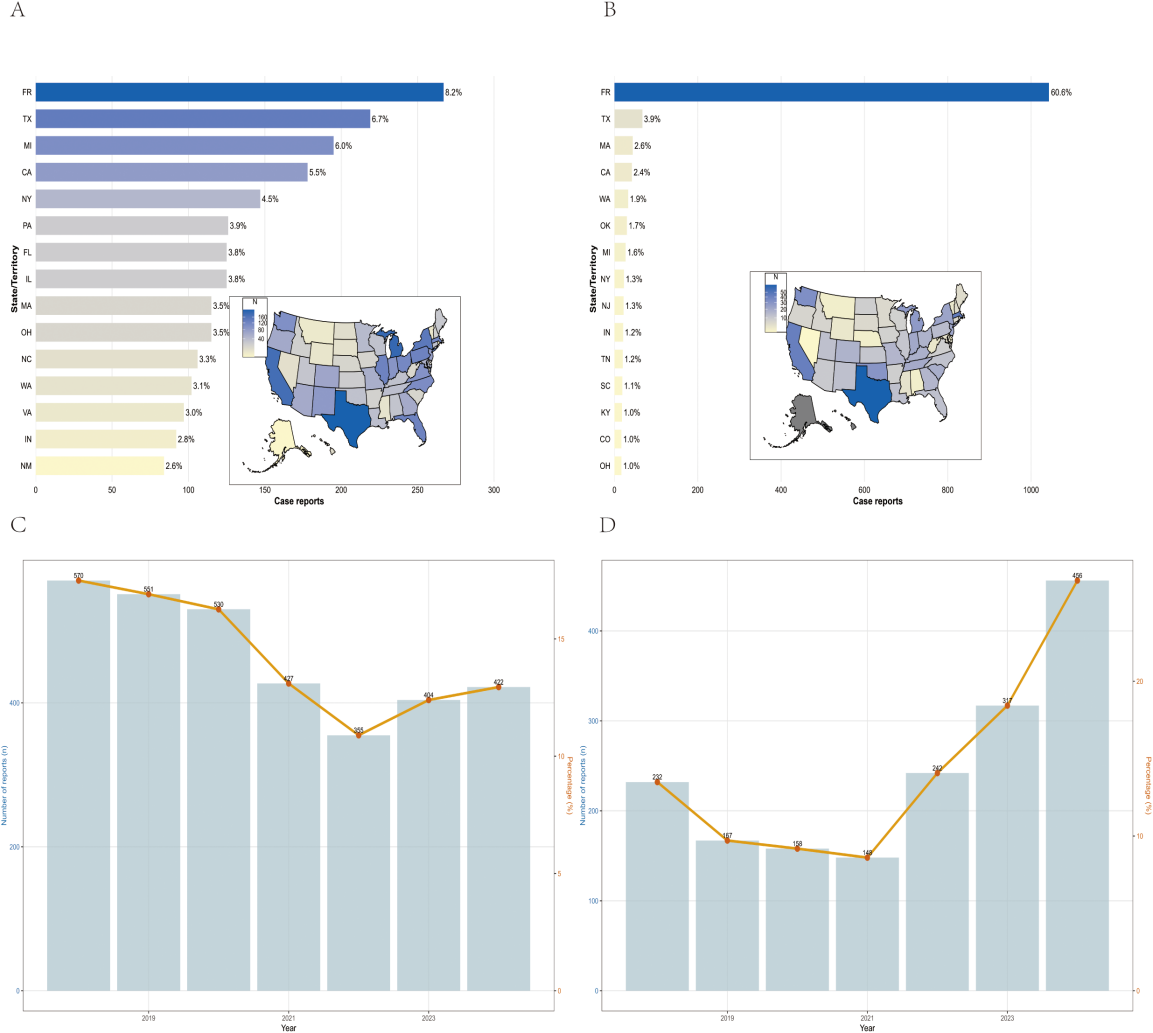
Geographical and national distribution of reported AEFIs in the pentavalent **(A)** and hexavalent **(B)** vaccine groups. Trends in AEFI reports for pentavalent **(C)** and hexavalent **(D)** vaccines, 2018–2024.

**Figure 2:**
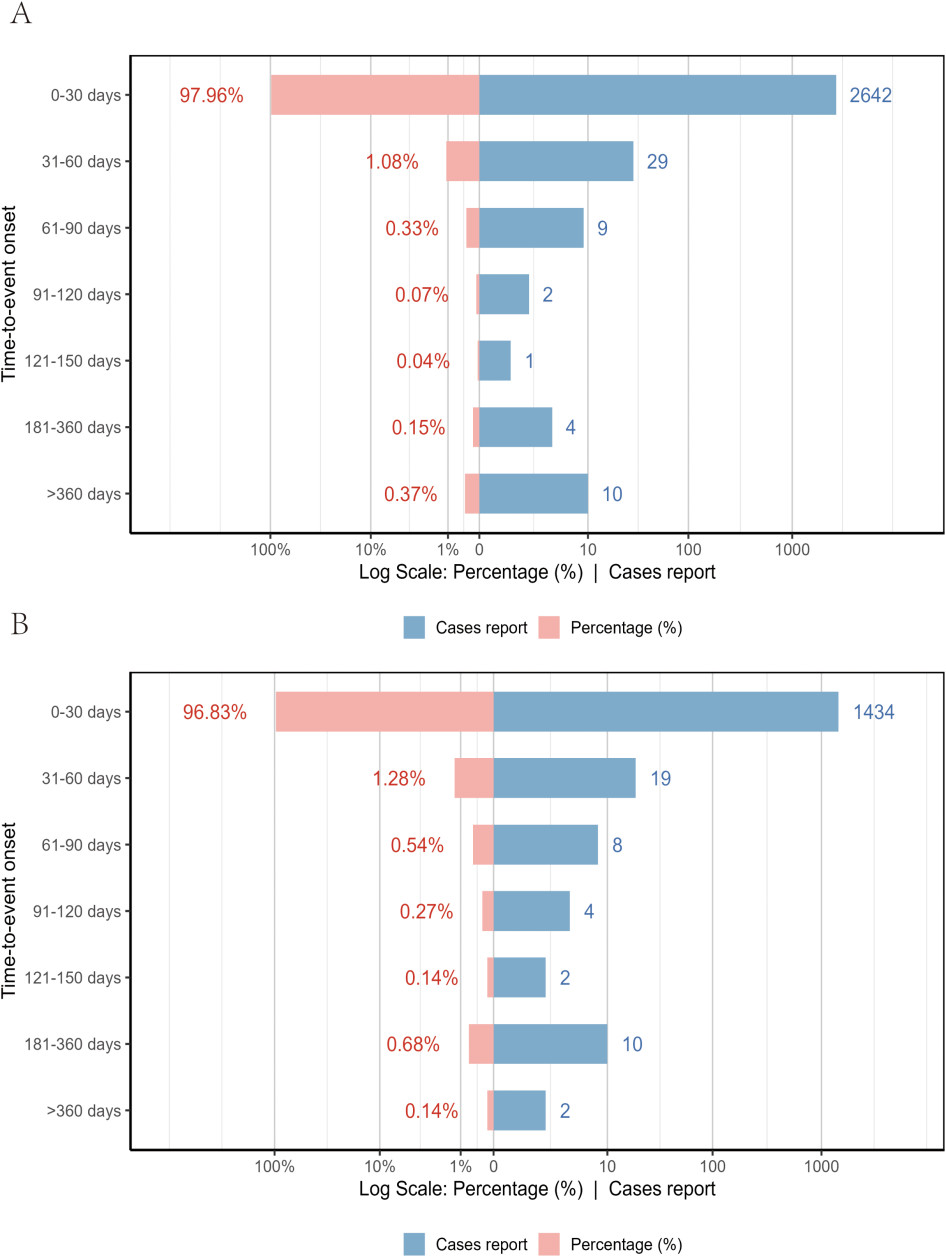
Time to onset of AEFIs associated with the pentavalent **(A)** and hexavalent **(B)** vaccine groups.

Between January 1, 2018, and December 31, 2024, VAERS received a total of 3,259 reports of AEFIs related to the pentavalent vaccine and 1,720 reports related to the hexavalent vaccine, all involving infants aged 6 weeks to 2 years. As shown in **Table 1**, in terms of sex distribution, the majority of reports for both vaccines involved male recipients, accounting for 48.7% and 49.3%, respectively. Regarding the severity of AEFIs, non-serious reports predominated, comprising 86.5% for the pentavalent vaccine and 61.7% for the hexavalent vaccine. Among VAERS reports, serious AEFIs were more frequently recorded following hexavalent vaccination than following pentavalent vaccination. Specifically, among serious cases, the proportions of death (2.3%), hospitalization (34.0%), prolonged hospitalization (0.7%), and life-threatening events (5.1%) were all higher for the hexavalent vaccine than for the pentavalent vaccine (2.1%, 10.1%, 0.3%, and 1.5%, respectively). In addition, both vaccines had a higher proportion of multi-dose than single-dose reports, with the pentavalent vaccine at 32.0%, significantly higher than the hexavalent vaccine at 12.0%. As for geographic distribution (**Figures 1A, B**), the top five regions reporting AEFIs for the pentavalent vaccine were foreign countries, Texas, Michigan, California, and New York; for the hexavalent vaccine, the top regions were foreign countries, Texas, Massachusetts, California, and Washington. Notably, reports from foreign countries far outnumbered those from any single U.S. state. In terms of yearly distribution (**Figures 1C, D**), the number of pentavalent vaccine reports peaked in 2018, while reports for the hexavalent vaccine reached their highest level in 2024. Regarding the onset time of AEFIs (**Figure 2**), most events for both vaccines were acute, occurring within 0–30 days post-vaccination. Only a small proportion of infants experienced AEFIs beyond 30 days after vaccination. These reporting patterns underscore the importance of continuous monitoring for potential AEFIs throughout the entire course of pentavalent and hexavalent vaccination.

**Table 1:** Characteristics of reports for pentavalent and hexavalent combination vaccines in the Vaccine Adverse Event Reporting System, January 1, 2018–December 31, 2024.

Characteristics	Pentavalent vaccine	Hexavalent vaccine
N	3259	1720
Gender		
Males	1588 (48.7%)	848 (49.3%)
Females	1483 (45.5%)	753 (43.8%)
Unknown	188 (5.8%)	119 (6.9%)
Serious symptom		
No	2819 (86.5%)	1062 (61.7%)
Yes	440 (13.5%)	658 (38.3%)
Vaccine alone		
No	2215 (68.0%)	1514 (88.0%)
Yes	1044 (32.0%)	206 (12.0%)
Died		
Not Available	3189 (97.9%)	1681 (97.7%)
Yes	70 (2.1%)	39 (2.3%)
Life-threatening		
Not Available	3211 (98.5%)	1632 (94.9%)
Yes	48 (1.5%)	88 (5.1%)
Hospital^a^		
Not Available	2929 (89.9%)	1135 (66.0%)
Yes	330 (10.1%)	585 (34.0%)
Prolonged hospitalization^b^		
Not Available	3250 (99.7%)	1708 (99.3%)
Yes	9 (0.3%)	12 (0.7%)
Disable^c^		
Not Available	3198 (98.1%)	1688 (98.1%)
Yes	61 (1.9%)	32 (1.9%)
Recovered^d^		
Not Available	284 (8.7%)	65 (3.8%)
Yes	1203 (36.9%)	904 (52.6%)
No	444 (13.6%)	362 (21.0%)
Unknown	1328 (40.7%)	389 (22.6%)

a Hospital: Reports of hospitalization following vaccination.

b Prolonged hospitalization: Reports of prolonged hospitalization due to vaccine-related adverse events.

c Disable: Reports of disability following vaccination.

d Recovered: Reports of recovery from adverse events following vaccination.

### AEFI analysis

To assess the potential safety impact of incorporating HepB into the pentavalent formulation to form the hexavalent vaccine, we compared the distribution and signal strength of AEFIs associated with the two vaccines at the SOC and PT levels. The detailed disproportionality findings at the SOC and PT levels are provided in **Supplementary Tables 3**-**5**.

After excluding SOCs unrelated to vaccination, disproportionality analyses identified a similar number of SOCs for both vaccines, with the hexavalent vaccine showing one additional SOC related to pregnancy and perinatal conditions. “General disorders and administration site conditions” and “Investigations” were commonly reported for both vaccines, while “Nervous system disorders” were notably more frequent with the hexavalent vaccine. Several SOCs were identified with potential safety signals by meeting at least two out of the four disproportionality methods. Notably, the hexavalent vaccine showed potential safety signals in a broader range of SOCs, including nervous system disorders (ROR = 1.95; IC025 = 0.70), vascular disorders (ROR = 2.89; IC025 = 1.17), cardiac disorders (ROR = 1.92; IC025 = 0.45), and respiratory disorders (ROR = 1.33; IC025 = 0.19). In contrast, the pentavalent vaccine generated significant signals in only three SOCs (e.g., investigations, injury). These disproportionality findings indicate broader or stronger potential safety signals in reports for the hexavalent vaccine in certain organ systems, warranting further attention and verification. For full details on the disproportionality analysis for these SOCs, please refer to **Supplementary Table 3**.

**Figure 3:**
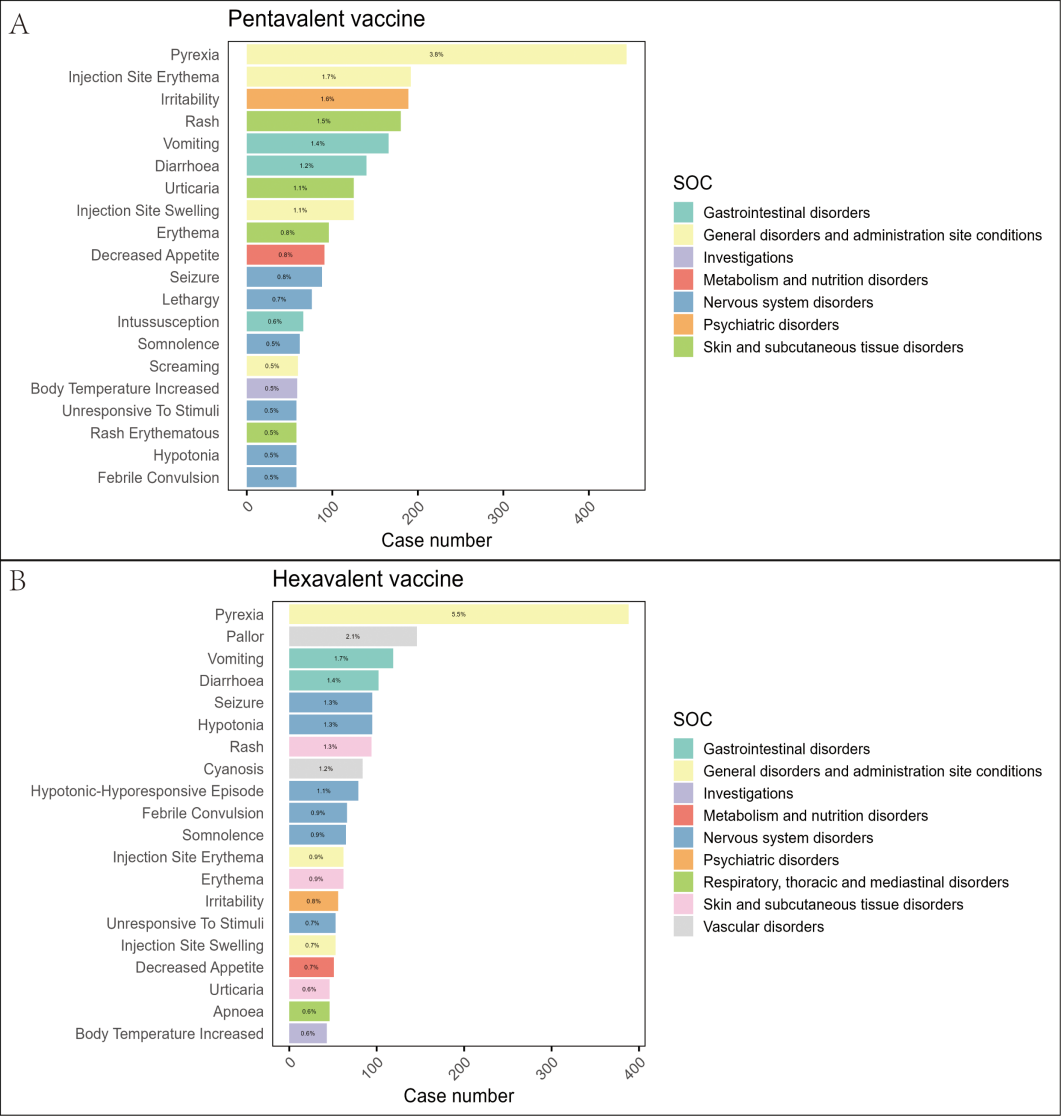
Distribution of the top 20 most frequently reported AEFIs at the PT level and their SOC classification in the pentavalent **(A)** and hexavalent **(B)** vaccine groups.

At the PT level, we applied the four disproportionality analysis methods to identify and exclude PTs unrelated to vaccination or lacking essential information. A total of 1,027 PTs were identified for the pentavalent vaccine (**Supplementary Table 4**) and 877 for the hexavalent vaccine (**Supplementary Table 5**). **Figure 3** illustrates the top 20 most frequently reported PTs. The results showed substantial overlap in commonly reported AEFIs between the two vaccines, primarily involving the nervous and gastrointestinal systems. In addition, we identified several PTs not explicitly listed in the package inserts, which may represent potential safety signals warranting further evaluation. For the pentavalent vaccine, these included seizure-like phenomena, oculomotor disturbances, pupillary abnormalities, elevated platelet count, hyperkalemia, and abnormal leukocyte differentials. For the hexavalent vaccine, additional PTs included oculomotor disturbances, seizure-like phenomena, developmental delay, abnormal fontanelle, and elevated platelet count. These findings enhance our understanding of the safety profiles of both vaccines and suggest that the addition of the HepB component in the hexavalent vaccine may be associated with additional safety considerations in specific organ systems.

### Subgroup analysis

**Figure 4:**
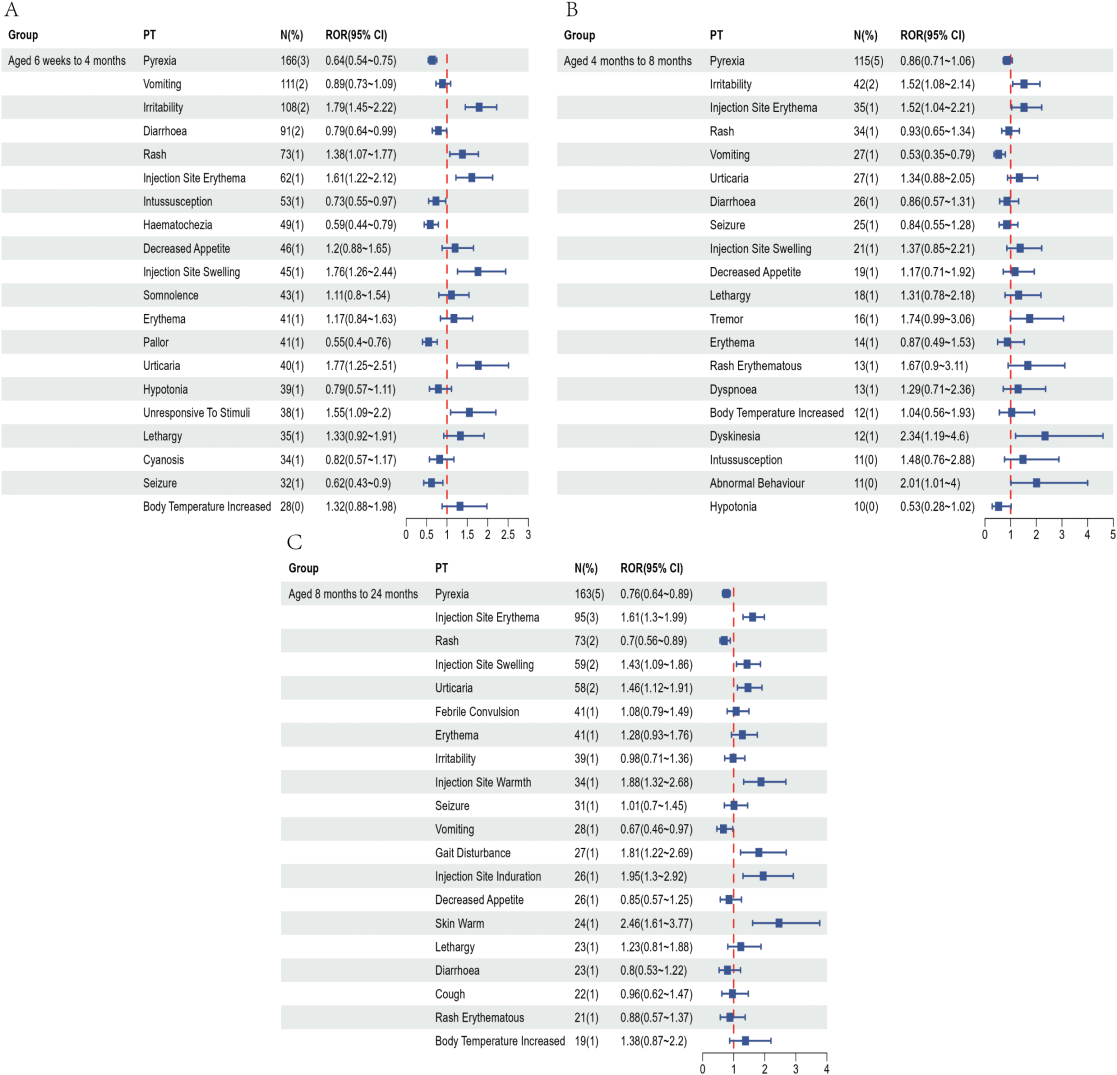
Top 20 most frequently reported AEFIs at the PT level and their RORs with 95% confidence intervals following pentavalent vaccination in infants aged 6 weeks to 4 months **(A)**, over 4 to 8 months **(B)**, and over 8 months to 2 years **(C)**.

**Figure 5:**
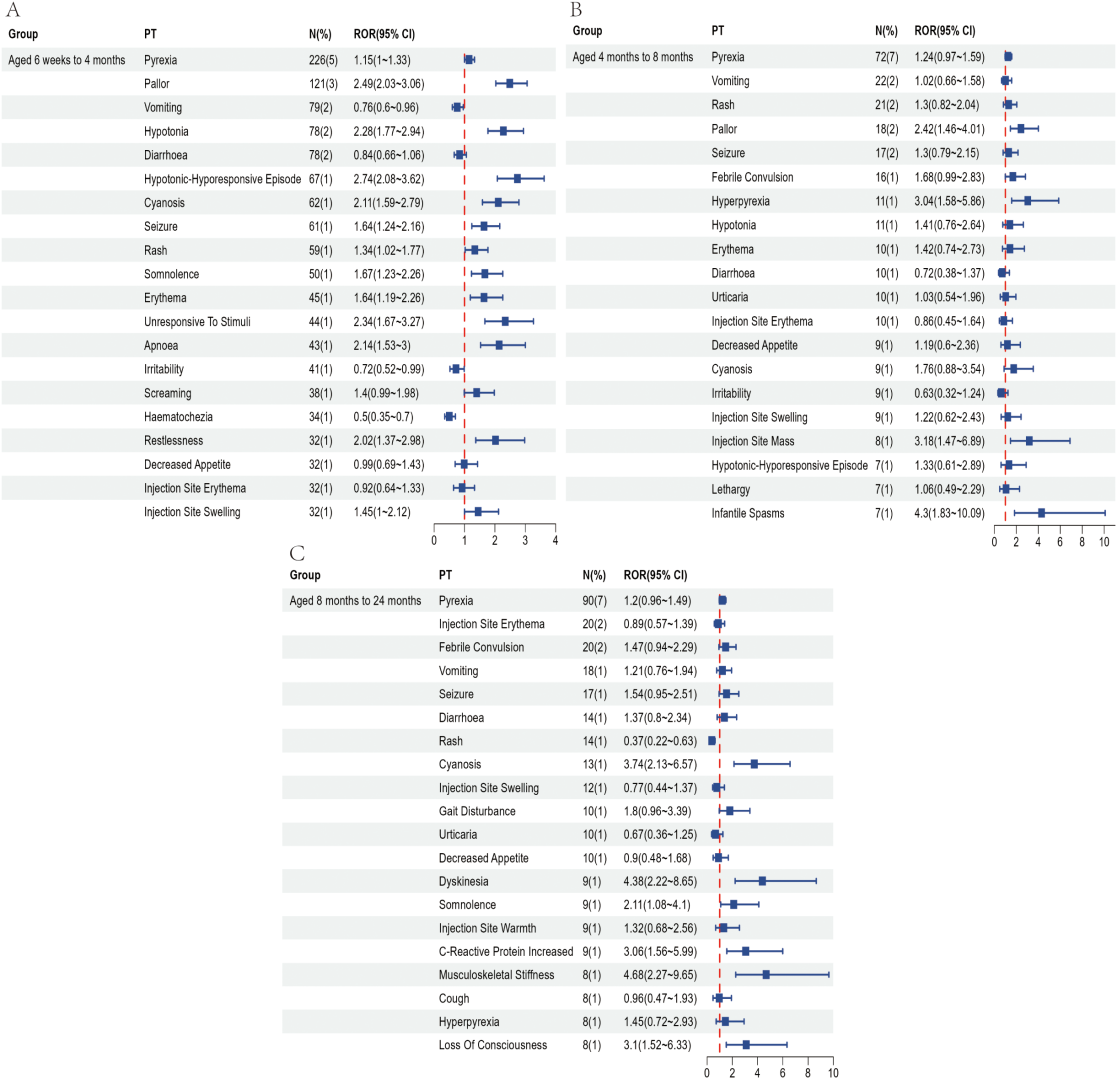
Top 20 most frequently reported AEFIs at the PT level and their RORs with 95% confidence intervals following hexavalent vaccination in infants aged 6 weeks to 4 months **(A)**, over 4 to 8 months **(B)**, and over 8 months to 2 years **(C)**.

To partially control for the confounding effects of demographic characteristics, we conducted a subgroup analysis based on age at vaccination. Recipients of the pentavalent and hexavalent vaccines were stratified into three subgroups: 6 weeks to 4 months, 4 to 8 months, and 8 months to 2 years. As shown in **Supplementary Table 6**, the highest number of AEFI reports for both vaccines occurred in the 6 weeks to 4 months subgroup. A further comparison of the top 20 most frequently reported PTs between the two vaccines in this age group (**Figures 4**, **5**) revealed that the hexavalent-related reports more often included serious respiratory AEFIs such as apnea and cyanosis, as well as hypotonic-hyporesponsive episodes. In addition, systemic reactions such as screaming, hematochezia, and loss of responsiveness were more frequently reported following hexavalent vaccination in this age group. Among infants aged 4 to 8 months, reports following hexavalent vaccination more frequently included neurological AEFIs, including febrile seizures, high fever, and infantile spasms, compared to the pentavalent vaccine. In the 8 months to 2 years subgroup, AEFIs related to the pentavalent vaccine were primarily mild, including local injection-site reactions (e.g., erythema, induration, fever) and minor systemic symptoms (e.g., drowsiness, elevated body temperature), consistent with typical post-vaccination profiles. In contrast, reports for the hexavalent vaccine described a broader range of serious neurological and systemic reactions, including cyanosis, loss of consciousness, elevated C-reactive protein, high fever, musculoskeletal rigidity, and movement disorders. These reporting differences could reflect differential reactogenicity or reporting behavior; mechanistic inferences cannot be drawn from VAERS data. In addition, stratified analyses were performed based on sex (**Supplementary Figure 1**) and AEFI severity (serious vs. non-serious, **Supplementary Figure 2**). These findings highlight reporting patterns that warrant attention and may inform clinical monitoring practices and future research on vaccination strategies for infants.

### Multivariate logistic regression

**Table 2** summarizes the results of the multivariate logistic regression analysis assessing factors associated with VAERS reports being classified as death following vaccination among infants. The results indicate that increasing age was significantly associated with lower odds of a report being classified as death in both the pentavalent and hexavalent vaccine groups. Compared with infants aged 6 weeks to 4 months, those aged 4 to 8 months had a significantly lower risk of death in both groups—OR = 0.212 (95% CI: 0.081–0.458, P = 0.0003) for the pentavalent group, and OR = 0.199 (95% CI: 0.032–0.660, P = 0.0273) for the hexavalent group. Similarly, infants aged 8 months to 2 years showed further reductions in death risk, with OR = 0.170 (95% CI: 0.065–0.367, P < 0.0001) for the pentavalent group and OR = 0.098 (95% CI: 0.006–0.458, P = 0.0224) for the hexavalent group. Female infants exhibited a significantly lower risk of death compared to males in both vaccine groups. In the pentavalent group, the adjusted odds ratio for females was 0.440 (95% CI: 0.256–0.732, P = 0.0021), while in the hexavalent group it was 0.390 (95% CI: 0.179–0.785, P = 0.0117).Regarding co-administration with other vaccines, a notable difference was observed between the two groups. In the pentavalent group, co-administration significantly increased the risk of death (OR = 6.694, 95% CI: 2.745–22.126, P = 0.0002), whereas in the hexavalent group, the association was not statistically significant (OR = 1.734, 95% CI: 0.516–10.799, P = 0.4540).

**Table 2:** Multivariable logistic regression analysis of death following pentavalent and hexavalent vaccination.

Characteristics	Pentavalent OR (95% CI)	*P*-value	Hexavalent OR (95%CI)	*P*-value
Age group				
6 weeks to 4 months	Reference			
4 to 8 months	0.212 (0.081–0.458)	0.0003	0.199(0.032–0.660)	0.0273
8 months to 2 years	0.170 (0.065–0.367)	0.0000	0.098(0.006–0.458)	0.0224
Gender				
Male	Reference			
Female	0.440 (0.256–0.732)	0.0021	0.390(0.179–0.785)	0.0117
Vaccine alone				
Yes	Reference			
No	6.694 (2.745–22.126)	0.0002	1.734(0.516–10.799)	0.4540

## Discussion

This study utilized data from the VAERS to systematically evaluate the characteristics of AEFI reports associated with the pentavalent vaccine (DTaP-IPV-Hib) and the hexavalent vaccine (DTaP-IPV-Hib-HepB) among infants aged 6 weeks to 2 years. Using four disproportionality analysis methods and multivariate logistic regression, we compared the safety signals between the two vaccines. Overall, both vaccines demonstrated favorable safety profiles. However, in our analysis, reports related to the hexavalent vaccine more frequently involved serious AEFIs and exhibited stronger and broader safety signals across multiple system organ classes, particularly within certain subgroups, highlighting the need for enhanced safety surveillance during its widespread implementation.

In terms of overall reporting characteristics, the proportion of serious AEFIs—including death, hospitalization, and life-threatening events—was significantly higher for the hexavalent vaccine compared to the pentavalent vaccine, with hospitalization and life-threatening events being particularly elevated. Previous clinical studies have reported that the incidence of serious AEFIs following hexavalent vaccination ranges from approximately 2.4% to 6.0%, which is notably higher than that observed with the pentavalent vaccine (Kosalaraksa et al., 2011; Silfverdal et al., 2016). For the latter, serious AEFIs are typically reported at a rate of 1.5 to 8 cases per 100,000 doses administered (Li et al., 2020; Pan et al., 2022). This difference in reporting patterns might be explained by the inclusion of HBV antigen in the hexavalent vaccine. The recombinant HBV surface antigen is typically adsorbed onto aluminum hydroxide adjuvant to enhance immunogenicity (Mast et al., 2005). When the HBV antigen is incorporated into the hexavalent vaccine, additional or more potent aluminum-based adjuvants are often required to ensure adequate immune response (Syed, 2019). This heightened immune stimulation may contribute to more pronounced systemic reactions, thereby increasing the proportion of reported serious AEFIs (Petrovsky and Aguilar, 2004). Although the inclusion of the hepatitis B component in the hexavalent formulation is intended to simplify the immunization schedule and improve coverage, it may also lead to more complex immune responses—particularly in young infants whose immune systems are not yet fully developed and are more susceptible to overstimulation.

At the SOC level, disproportionality analysis revealed that the hexavalent vaccine was associated with signals spanning multiple systems, including the nervous, respiratory, cardiovascular, metabolic, and musculoskeletal systems. Notably, the signal intensity for most SOCs was higher for the hexavalent vaccine compared to the pentavalent vaccine, suggesting a broader range of potential immune responses. Particular attention should be given to serious manifestations such as neurological disorders (e.g., seizure-like episodes, infantile spasms), cardiovascular events (e.g., cyanosis, arrhythmias), and respiratory depression (e.g., apnea). These findings were further supported by the distribution of specific PTs. In the nervous system domain, previous studies have shown that the hexavalent vaccine is more likely to induce fever than the pentavalent vaccine, potentially increasing the risk of febrile seizures (Kim et al., 2017; Oliver and Moore, 2020). In the respiratory system, clinical reports indicate that approximately 11%–13% of preterm infants experience apnea or bradycardia following hexavalent vaccination (Schulzke et al., 2005; Faldella et al., 2007). The European Medicines Agency has also advised that for extremely preterm infants (≤28 weeks of gestational age), the potential risk of post-vaccination apnea should be considered during the primary immunization series, recommending 48–72 hours of respiratory monitoring, especially in those with a history of respiratory immaturity (European Medicines Agency, 2020). Regarding the cardiovascular system, case reports have described sudden unexpected death in a 3-month-old girl following hexavalent vaccination. Autopsy findings revealed underdevelopment of the arcuate nucleus in the brainstem and abnormalities in the cardiac conduction system, suggesting that in infants with immature autonomic regulatory function, vaccination may trigger arrhythmias or abnormal vagal reflex responses (Ottaviani et al., 2006). Nevertheless, current evidence from clinical trials and post-marketing surveillance generally supports the overall safety of the hexavalent vaccine, with serious AEFIs remaining extremely rare (Fortunato et al., 2022). It is important to emphasize, however, that our signal detection analysis highlights the need for heightened vigilance in high-sensitivity populations, such as young infants and preterm neonates. During widespread vaccine implementation, proactive surveillance systems should be employed to facilitate comprehensive risk–benefit assessments, particularly for high-risk groups, to ensure both the safety and effectiveness of immunization strategies.

Subgroup analysis showed that the timing of vaccination corresponded to differences in reporting patterns within VAERS. Among infants aged 6 weeks to 4 months, the incidence of hexavalent vaccine–associated AEFIs was notably higher, particularly involving serious systemic reactions such as apnea, cyanosis, persistent crying, and loss of responsiveness. This phenomenon may be attributed to the fact that this developmental window represents a critical transition period in which the infant’s immune system is shifting from innate to adaptive immunity. During this stage, the immune response to multiple antigens and adjuvants may be heightened, increasing susceptibility to excessive immune activation (Sanchez-Schmitz and Levy, 2011). Previous studies have demonstrated that preterm and extremely preterm infants are at elevated risk of experiencing AEFIs such as apnea, bradycardia, and oxygen desaturation following administration of the hexavalent vaccine (Knuf et al., 2023). These findings suggest that infants in this age group may experience higher frequencies of certain reported events. Although VAERS cannot establish causality or quantify risk, enhanced post-vaccination monitoring is warranted, and careful consideration should be given to the necessity and safety of combination vaccines when designing immunization strategies for this vulnerable population.

Multivariable logistic regression analysis revealed a significant inverse association between increasing infant age and the risk of death, which may reflect the progressive maturation of the immune system during early development and improved vaccine tolerance. Moreover, reports involving female infants had lower odds of being classified as deaths. This finding is consistent with existing literature on sex-related immune differences; however, VAERS data cannot elucidate the underlying mechanisms, and factors such as differential care-seeking behavior or reporting patterns may also contribute. Although females are generally more prone to mild-to-moderate AEFIs, their risk of severe adverse outcomes appears to be lower (Klein et al., 2015). Previous studies have demonstrated sex-based differences in immune responses during infancy, with females tending toward a Th2-dominant profile characterized by antibody production and immune regulation, while males exhibit a Th1-skewed response involving cytotoxicity and inflammation (Noho-Konteh et al., 2014). Although Th1 responses may facilitate rapid pathogen clearance, they are also more likely to trigger systemic inflammatory reactions such as encephalopathy or respiratory arrest—potentially explaining the approximately 40% higher mortality risk observed in male infants (Goldman and Miller, 2012). Notably, this sex disparity is most pronounced within the first six months of life, consistent with prior findings on gender-related immunological differences. Furthermore, co-administration of vaccines was significantly associated with higher odds of reports being classified as deaths among pentavalent-related cases, whereas no such association was observed in the hexavalent group. This finding suggests that vaccine co-administration strategies may need to be tailored rather than uniformly applied across different vaccine types or populations. Instead, careful consideration of antigenic load and individual immune characteristics is warranted to avoid excessive immune burden and the potential for adverse outcomes.

Despite the use of large-scale real-world data from the VAERS database, which enhances the external validity and practical relevance of our findings, several limitations should be acknowledged. First, and most importantly, VAERS lacks denominator data (i.e., the number of doses administered), which prevents the calculation of incidence rates and means that differences in reporting frequencies between the pentavalent and hexavalent vaccines may reflect differences in vaccine uptake or reporting practices rather than true differences in safety. Second, as a passive surveillance system, VAERS is subject to underreporting, incomplete event descriptions, and reporting biases. Because VAERS is a passive surveillance system without reliable denominators, medical record adjudication, or control for stimulated reporting, these analyses describe reporting patterns and disproportionality signals only. They cannot estimate incidence or infer causality. External clinical and epidemiologic studies are needed to confirm or refute these signals. Third, the classification of AEFI severity and the assessment of causal relationships partly rely on manual standardization processes, which may introduce subjective errors or inconsistencies. Fourth, the disproportionality analysis methods employed in this study are designed to detect potential safety signals through association patterns, rather than to establish definitive causal relationships. Taken together, these limitations highlight the need for continuous improvement in vaccine safety surveillance systems and the integration of complementary data sources to support more accurate and comprehensive risk assessment.

## Conclusion

This study, based on data from the VAERS, compared the characteristics of AEFIs associated with the pentavalent vaccine (DTaP-IPV-Hib) and the hexavalent vaccine (DTaP-IPV-Hib-HepB) in infants aged 6 weeks to 2 years. Overall, both vaccines demonstrated favorable safety profiles. However, reports for the hexavalent vaccine were more frequently for serious AEFIs and generated stronger disproportionality signals across multiple organ systems in our analysis, particularly in reports concerning younger infants. Increasing age and female sex were associated with lower odds of reports being classified as deaths, whereas vaccine co-administration was linked to higher odds of death classification within the pentavalent subset, with no clear association observed for the hexavalent subset. These findings are exploratory and hypothesis-generating, reflecting reporting patterns captured in VAERS rather than measured incidence or causality. They highlight the need for careful and focused post-vaccination monitoring, particularly among younger infants, to maintain scientific rigor and ensure the ongoing safety of immunization strategies as the hexavalent vaccine becomes more widely adopted.

## Data Availability

The original contributions presented in the study are included in the article/**Supplementary Material**. Further inquiries can be directed to the corresponding author.
