# Supplementary material for: Comparative safety evaluation of pentavalent (DTaP-IPV-Hib) and hexavalent (DTaP-IPV-Hib-HepB) vaccines in infants: a real-world analysis based on VAERS
Source: Front Cell Infect Microbiol. 2025 Oct 30;15:1666509. doi: 10.3389/fcimb.2025.1666509 (PMC12611864; doi:10.3389/fcimb.2025.1666509)
Supplement: Supplementary file 3 [file Table1.docx]

**Supplementary Information and Data**

**Comparative Safety Evaluation of Pentavalent (DTaP-IPV-Hib) and Hexavalent (DTaP-IPV-Hib-HepB) Vaccines in Infants: A Real-World Analysis** **Based on VAERS**

**Table of content**

Table 1: Fourfold table of disproportionality method.

Table 2: Formulas and signal detection criterias for reporting odds ratio (ROR), proportional reporting ratio (PRR) and bayesian confidence propagation neural network (BCPNN).

Table 3: PT-level distribution and signal strength of reported AEFIs in the pentavalent vaccine group.

Table 4: PT-level distribution and signal strength of reported AEFIs in the hexavalent vaccine group.

Table 5: Reported frequencies of AEFIs for pentavalent and hexavalent vaccines in infants aged 6 weeks to 4 months, 4 to 8 months, and 8 months to 2 years.

Figure 1: Top 20 most frequently reported AEFIs at the PT level and their RORs with 95% confidence intervals following vaccination: pentavalent vaccine in females (B) and males (A), and hexavalent vaccine in males (C) and females (D).

Figure 2: Top 20 most frequently reported AEFIs at the PT level and their RORs with 95% confidence intervals: serious reports following pentavalent vaccination (A), non-serious reports following pentavalent vaccination (B), serious reports following hexavalent vaccination (C), and non-serious reports following hexavalent vaccination (D).
