# Supplementary material for: Comparative safety evaluation of pentavalent (DTaP-IPV-Hib) and hexavalent (DTaP-IPV-Hib-HepB) vaccines in infants: a real-world analysis based on VAERS
Source: Front Cell Infect Microbiol. 2025 Oct 30;15:1666509. doi: 10.3389/fcimb.2025.1666509 (PMC12611864; doi:10.3389/fcimb.2025.1666509)
Supplement: Supplementary file 4 [file Table2.docx]

**Table 1:** Fourfold table of disproportionality method.

|  | **Target adverse event reported** | **Other adverse event reported** | **Total** |
| --- | --- | --- | --- |
| Target vaccine | a | b | a+b |
| Other vaccine | c | d | c+d |
| Total | a+c | b+d | a+b+c+d |
