# Supplementary material for: Comparative safety evaluation of pentavalent (DTaP-IPV-Hib) and hexavalent (DTaP-IPV-Hib-HepB) vaccines in infants: a real-world analysis based on VAERS
Source: Front Cell Infect Microbiol. 2025 Oct 30;15:1666509. doi: 10.3389/fcimb.2025.1666509 (PMC12611864; doi:10.3389/fcimb.2025.1666509)
Supplement: Supplementary file 5 [file Table3.docx]

**Table 3. SOC-level distribution of AEFIs and signal strength between** **the pentavalent and hexavalent vaccine groups.**

| **SOC** | **N** | **ROR(95%Cl)** | **PRR(X^2^)** | **EBGM(EBGM05)** | **IC(IC025)** |
| --- | --- | --- | --- | --- | --- |
| **Pentavalent vaccine** | | | | | |
| General disorders and administration site conditions | 2796 | 0.95 ( 0.91 - 0.99 ) | 0.96 ( 5.06 ) | 0.97 ( 0.93 ) | -0.05 ( -0.11 ) |
| Investigations | 2333 | 1.2 ( 1.14 - 1.25 ) | 1.16 ( 54.2 ) | 1.14 ( 1.1 ) | 0.19 ( 0.12 ) |
| Injury, poisoning and procedural complications | 1921 | 1.16 ( 1.11 - 1.23 ) | 1.14 ( 33.54 ) | 1.12 ( 1.08 ) | 0.17 ( 0.1 ) |
| Nervous system disorders | 902 | 0.96 ( 0.9 - 1.03 ) | 0.97 ( 1.13 ) | 0.97 ( 0.91 ) | -0.05 ( -0.15 ) |
| Skin and subcutaneous tissue disorders | 845 | 0.89 ( 0.82 - 0.95 ) | 0.9 ( 10.34 ) | 0.9 ( 0.85 ) | -0.15 ( -0.25 ) |
| Gastrointestinal disorders | 672 | 0.94 ( 0.86 - 1.02 ) | 0.94 ( 2.45 ) | 0.95 ( 0.88 ) | -0.08 ( -0.2 ) |
| Psychiatric disorders | 496 | 1.12 ( 1.02 - 1.23 ) | 1.11 ( 5.46 ) | 1.1 ( 1.02 ) | 0.14 ( 0 ) |
| Respiratory, thoracic and mediastinal disorders | 270 | 0.73 ( 0.65 - 0.83 ) | 0.74 ( 23.78 ) | 0.76 ( 0.68 ) | -0.4 ( -0.58 ) |
| Infections and infestations | 241 | 0.51 ( 0.45 - 0.59 ) | 0.52 ( 102.83 ) | 0.55 ( 0.49 ) | -0.87 ( -1.06 ) |
| Metabolism and nutrition disorders | 210 | 1.11 ( 0.96 - 1.29 ) | 1.11 ( 2.16 ) | 1.1 ( 0.98 ) | 0.14 ( -0.07 ) |
| Surgical and medical procedures | 206 | 1.47 ( 1.27 - 1.7 ) | 1.46 ( 26.3 ) | 1.4 ( 1.24 ) | 0.49 ( 0.27 ) |
| Musculoskeletal and connective tissue disorders | 172 | 1.06 ( 0.91 - 1.24 ) | 1.06 ( 0.56 ) | 1.06 ( 0.92 ) | 0.08 ( -0.15 ) |
| Vascular disorders | 148 | 0.82 ( 0.69 - 0.97 ) | 0.82 ( 5.57 ) | 0.83 ( 0.72 ) | -0.26 ( -0.51 ) |
| Eye disorders | 118 | 1.12 ( 0.92 - 1.35 ) | 1.12 ( 1.28 ) | 1.1 ( 0.94 ) | 0.14 ( -0.14 ) |
| Blood and lymphatic system disorders | 86 | 0.76 ( 0.61 - 0.95 ) | 0.76 ( 5.99 ) | 0.78 ( 0.65 ) | -0.36 ( -0.68 ) |
| Immune system disorders | 62 | 0.74 ( 0.57 - 0.96 ) | 0.74 ( 5.16 ) | 0.76 ( 0.61 ) | -0.4 ( -0.77 ) |
| Cardiac disorders | 35 | 0.75 ( 0.53 - 1.05 ) | 0.75 ( 2.8 ) | 0.76 ( 0.57 ) | -0.39 ( -0.89 ) |
| Product issues | 28 | 0.93 ( 0.63 - 1.37 ) | 0.93 ( 0.13 ) | 0.94 ( 0.68 ) | -0.09 ( -0.65 ) |
| Renal and urinary disorders | 20 | 1.14 ( 0.72 - 1.81 ) | 1.14 ( 0.32 ) | 1.13 ( 0.77 ) | 0.17 ( -0.49 ) |
| Congenital, familial and genetic disorders | 19 | 1.26 ( 0.78 - 2.02 ) | 1.26 ( 0.89 ) | 1.23 ( 0.82 ) | 0.3 ( -0.38 ) |
| Ear and labyrinth disorders | 12 | 0.67 ( 0.37 - 1.2 ) | 0.67 ( 1.83 ) | 0.69 ( 0.42 ) | -0.54 ( -1.36 ) |
| Reproductive system and breast disorders | 6 | 0.95 ( 0.41 - 2.19 ) | 0.95 ( 0.01 ) | 0.95 ( 0.47 ) | -0.07 ( -1.21 ) |
| Neoplasms benign, malignant and unspecified (incl cysts and polyps) | 5 | 0.76 ( 0.31 - 1.88 ) | 0.76 ( 0.36 ) | 0.77 ( 0.36 ) | -0.37 ( -1.6 ) |
| Hepatobiliary disorders | 5 | 0.41 ( 0.17 - 1.01 ) | 0.41 ( 3.98 ) | 0.44 ( 0.21 ) | -1.2 ( -2.4 ) |
| Pregnancy, puerperium and perinatal conditions | 1 | 0.87 ( 0.11 - 6.69 ) | 0.87 ( 0.02 ) | 0.88 ( 0.16 ) | -0.18 ( -2.37 ) |
| **Hexavalent vaccine** | | | | | |
| General disorders and administration site conditions | 1557 | 0.84 ( 0.79 - 0.89 ) | 0.88 ( 34.54 ) | 0.88 ( 0.84 ) | -0.18 ( -0.26 ) |
| Investigations | 1438 | 1.21 ( 1.14 - 1.28 ) | 1.16 ( 37.94 ) | 1.15 ( 1.1 ) | 0.21 ( 0.12 ) |
| Nervous system disorders | 989 | 1.95 ( 1.82 - 2.09 ) | 1.82 ( 358.73 ) | 1.74 ( 1.64 ) | 0.8 ( 0.7 ) |
| Gastrointestinal disorders | 560 | 1.34 ( 1.23 - 1.47 ) | 1.31 ( 41.84 ) | 1.29 ( 1.2 ) | 0.37 ( 0.24 ) |
| Injury, poisoning and procedural complications | 433 | 0.36 ( 0.33 - 0.4 ) | 0.4 ( 440.85 ) | 0.42 ( 0.38 ) | -1.27 ( -1.41 ) |
| Skin and subcutaneous tissue disorders | 433 | 0.73 ( 0.66 - 0.81 ) | 0.75 ( 37.78 ) | 0.76 ( 0.7 ) | -0.4 ( -0.54 ) |
| Psychiatric disorders | 306 | 1.13 ( 1 - 1.27 ) | 1.12 ( 4.1 ) | 1.12 ( 1.01 ) | 0.16 ( -0.01 ) |
| Respiratory, thoracic and mediastinal disorders | 281 | 1.33 ( 1.17 - 1.5 ) | 1.31 ( 20.32 ) | 1.29 ( 1.17 ) | 0.37 ( 0.19 ) |
| Vascular disorders | 278 | 2.89 ( 2.54 - 3.28 ) | 2.81 ( 284.76 ) | 2.57 ( 2.3 ) | 1.36 ( 1.17 ) |
| Infections and infestations | 173 | 0.62 ( 0.53 - 0.73 ) | 0.63 ( 37.24 ) | 0.64 ( 0.57 ) | -0.63 ( -0.86 ) |
| Musculoskeletal and connective tissue disorders | 138 | 1.43 ( 1.2 - 1.7 ) | 1.42 ( 16.13 ) | 1.39 ( 1.2 ) | 0.47 ( 0.22 ) |
| Metabolism and nutrition disorders | 132 | 1.15 ( 0.96 - 1.37 ) | 1.14 ( 2.29 ) | 1.14 ( 0.98 ) | 0.18 ( -0.08 ) |
| Surgical and medical procedures | 80 | 0.89 ( 0.71 - 1.11 ) | 0.89 ( 1.1 ) | 0.89 ( 0.74 ) | -0.16 ( -0.49 ) |
| Eye disorders | 77 | 1.2 ( 0.95 - 1.51 ) | 1.19 ( 2.3 ) | 1.18 ( 0.97 ) | 0.24 ( -0.1 ) |
| Immune system disorders | 59 | 1.2 ( 0.92 - 1.57 ) | 1.2 ( 1.86 ) | 1.19 ( 0.95 ) | 0.25 ( -0.14 ) |
| Cardiac disorders | 51 | 1.92 ( 1.44 - 2.57 ) | 1.92 ( 20.26 ) | 1.83 ( 1.43 ) | 0.87 ( 0.45 ) |
| Blood and lymphatic system disorders | 48 | 0.7 ( 0.52 - 0.93 ) | 0.7 ( 5.91 ) | 0.71 ( 0.56 ) | -0.49 ( -0.91 ) |
| Congenital, familial and genetic disorders | 17 | 1.89 ( 1.15 - 3.12 ) | 1.89 ( 6.44 ) | 1.8 ( 1.19 ) | 0.85 ( 0.14 ) |
| Renal and urinary disorders | 9 | 0.82 ( 0.42 - 1.61 ) | 0.82 ( 0.32 ) | 0.83 ( 0.48 ) | -0.26 ( -1.2 ) |
| Ear and labyrinth disorders | 6 | 0.55 ( 0.24 - 1.25 ) | 0.55 ( 2.11 ) | 0.57 ( 0.29 ) | -0.82 ( -1.93 ) |
| Product issues | 5 | 0.26 ( 0.11 - 0.64 ) | 0.26 ( 10.15 ) | 0.27 ( 0.13 ) | -1.87 ( -3.05 ) |
| Neoplasms benign, malignant and unspecified (incl cysts and polyps) | 2 | 0.49 ( 0.12 - 2.01 ) | 0.49 ( 1.01 ) | 0.51 ( 0.16 ) | -0.98 ( -2.68 ) |
| Hepatobiliary disorders | 2 | 0.28 ( 0.07 - 1.11 ) | 0.28 ( 3.75 ) | 0.29 ( 0.09 ) | -1.8 ( -3.49 ) |
| Reproductive system and breast disorders | 1 | 0.25 ( 0.03 - 1.8 ) | 0.25 ( 2.21 ) | 0.26 ( 0.05 ) | -1.94 ( -4.01 ) |
