# Supplementary material for: Comparative safety evaluation of pentavalent (DTaP-IPV-Hib) and hexavalent (DTaP-IPV-Hib-HepB) vaccines in infants: a real-world analysis based on VAERS
Source: Front Cell Infect Microbiol. 2025 Oct 30;15:1666509. doi: 10.3389/fcimb.2025.1666509 (PMC12611864; doi:10.3389/fcimb.2025.1666509)
Supplement: Supplementary file 6 [file Table4.docx]

Table 4: PT-level distribution and signal strength of reported AEFIs in the pentavalent vaccine group.

| PT | N | ROR(95%Cl) | PRR(X^2^) | EBGM(EBGM05) | IC(IC025) |
| --- | --- | --- | --- | --- | --- |
| Pyrexia | 444 | 0.68 ( 0.62 - 0.75 ) | 0.69 ( 60.38 ) | 0.71 ( 0.66 ) | -0.49 ( -0.63 ) |
| Injection Site Erythema | 192 | 1.27 ( 1.09 - 1.47 ) | 1.26 ( 9.49 ) | 1.23 ( 1.09 ) | 0.3 ( 0.08 ) |
| Irritability | 189 | 1.46 ( 1.25 - 1.7 ) | 1.45 ( 23.85 ) | 1.4 ( 1.23 ) | 0.48 ( 0.26 ) |
| Rash | 180 | 0.71 ( 0.61 - 0.83 ) | 0.72 ( 19.41 ) | 0.73 ( 0.65 ) | -0.45 ( -0.67 ) |
| Vomiting | 166 | 0.89 ( 0.76 - 1.04 ) | 0.89 ( 2.1 ) | 0.9 ( 0.79 ) | -0.15 ( -0.39 ) |
| Diarrhoea | 140 | 0.98 ( 0.82 - 1.16 ) | 0.98 ( 0.07 ) | 0.98 ( 0.85 ) | -0.03 ( -0.28 ) |
| Injection Site Swelling | 125 | 1.2 ( 0.99 - 1.44 ) | 1.19 ( 3.58 ) | 1.17 ( 1.01 ) | 0.23 ( -0.04 ) |
| Urticaria | 125 | 1.23 ( 1.02 - 1.48 ) | 1.22 ( 4.61 ) | 1.2 ( 1.03 ) | 0.26 ( -0.01 ) |
| Erythema | 96 | 1.04 ( 0.84 - 1.28 ) | 1.04 ( 0.12 ) | 1.03 ( 0.87 ) | 0.05 ( -0.26 ) |
| Decreased Appetite | 91 | 0.99 ( 0.8 - 1.23 ) | 0.99 ( 0.01 ) | 0.99 ( 0.83 ) | -0.02 ( -0.33 ) |
| Seizure | 88 | 0.82 ( 0.66 - 1.02 ) | 0.82 ( 3.32 ) | 0.83 ( 0.69 ) | -0.27 ( -0.58 ) |
| Lethargy | 76 | 1.27 ( 1 - 1.61 ) | 1.26 ( 3.76 ) | 1.24 ( 1.01 ) | 0.3 ( -0.04 ) |
| Intussusception | 66 | 1.31 ( 1.02 - 1.7 ) | 1.31 ( 4.33 ) | 1.28 ( 1.03 ) | 0.35 ( -0.02 ) |
| Somnolence | 62 | 1.15 ( 0.88 - 1.49 ) | 1.15 ( 1.07 ) | 1.13 ( 0.91 ) | 0.18 ( -0.2 ) |
| Screaming | 60 | 1.28 ( 0.98 - 1.68 ) | 1.28 ( 3.32 ) | 1.25 ( 1 ) | 0.32 ( -0.07 ) |
| Body Temperature Increased | 59 | 1.28 ( 0.98 - 1.68 ) | 1.28 ( 3.2 ) | 1.25 ( 0.99 ) | 0.32 ( -0.07 ) |
| Rash Erythematous | 58 | 1 ( 0.76 - 1.31 ) | 1 ( 0 ) | 1 ( 0.8 ) | 0 ( -0.39 ) |
| Hypotonia | 58 | 0.99 ( 0.76 - 1.3 ) | 0.99 ( 0.01 ) | 0.99 ( 0.79 ) | -0.01 ( -0.41 ) |
| Unresponsive To Stimuli | 58 | 1.41 ( 1.07 - 1.85 ) | 1.41 ( 6 ) | 1.36 ( 1.08 ) | 0.44 ( 0.04 ) |
| Febrile Convulsion | 58 | 0.57 ( 0.43 - 0.74 ) | 0.57 ( 18.33 ) | 0.59 ( 0.47 ) | -0.76 ( -1.15 ) |
| Injection Site Warmth | 57 | 1.35 ( 1.03 - 1.79 ) | 1.35 ( 4.66 ) | 1.31 ( 1.04 ) | 0.39 ( -0.01 ) |
| Pallor | 57 | 0.75 ( 0.57 - 0.98 ) | 0.75 ( 4.51 ) | 0.77 ( 0.61 ) | -0.39 ( -0.78 ) |
| Haematochezia | 53 | 0.89 ( 0.67 - 1.18 ) | 0.89 ( 0.63 ) | 0.9 ( 0.71 ) | -0.15 ( -0.56 ) |
| Cyanosis | 48 | 0.91 ( 0.68 - 1.23 ) | 0.91 ( 0.37 ) | 0.92 ( 0.72 ) | -0.12 ( -0.55 ) |
| Injection Site Induration | 46 | 1.23 ( 0.91 - 1.67 ) | 1.23 ( 1.76 ) | 1.2 ( 0.93 ) | 0.27 ( -0.17 ) |
| Tremor | 45 | 1.27 ( 0.93 - 1.73 ) | 1.27 ( 2.26 ) | 1.24 ( 0.96 ) | 0.31 ( -0.14 ) |
| Fatigue | 44 | 0.98 ( 0.72 - 1.33 ) | 0.98 ( 0.02 ) | 0.98 ( 0.75 ) | -0.03 ( -0.48 ) |
| Cough | 43 | 0.68 ( 0.5 - 0.93 ) | 0.68 ( 6.04 ) | 0.7 ( 0.54 ) | -0.51 ( -0.96 ) |
| Eye Movement Disorder | 39 | 1.66 ( 1.19 - 2.34 ) | 1.66 ( 8.9 ) | 1.57 ( 1.18 ) | 0.65 ( 0.16 ) |
| Dyskinesia | 39 | 1.9 ( 1.35 - 2.67 ) | 1.89 ( 13.97 ) | 1.76 ( 1.32 ) | 0.81 ( 0.32 ) |
| Skin Warm | 38 | 1.55 ( 1.1 - 2.17 ) | 1.54 ( 6.36 ) | 1.47 ( 1.11 ) | 0.56 ( 0.07 ) |
| Ultrasound Abdomen Abnormal | 37 | 1.83 ( 1.29 - 2.59 ) | 1.82 ( 11.72 ) | 1.7 ( 1.27 ) | 0.77 ( 0.26 ) |
| Injection Site Reaction | 36 | 1.08 ( 0.77 - 1.52 ) | 1.08 ( 0.2 ) | 1.07 ( 0.8 ) | 0.1 ( -0.4 ) |
| Rash Macular | 36 | 1.25 ( 0.88 - 1.76 ) | 1.24 ( 1.55 ) | 1.22 ( 0.91 ) | 0.29 ( -0.21 ) |
| Dyspnoea | 34 | 0.9 ( 0.63 - 1.28 ) | 0.9 ( 0.36 ) | 0.91 ( 0.68 ) | -0.14 ( -0.65 ) |
| Pain | 33 | 0.88 ( 0.61 - 1.25 ) | 0.88 ( 0.53 ) | 0.89 ( 0.66 ) | -0.17 ( -0.69 ) |
| Injection Site Rash | 31 | 0.94 ( 0.65 - 1.36 ) | 0.94 ( 0.1 ) | 0.95 ( 0.7 ) | -0.08 ( -0.61 ) |
| Injection Site Pain | 31 | 0.63 ( 0.43 - 0.9 ) | 0.63 ( 6.55 ) | 0.65 ( 0.48 ) | -0.63 ( -1.15 ) |
| Gait Disturbance | 30 | 1 ( 0.69 - 1.46 ) | 1 ( 0 ) | 1 ( 0.73 ) | 0 ( -0.54 ) |
| Rash Papular | 30 | 1.22 ( 0.84 - 1.79 ) | 1.22 ( 1.1 ) | 1.2 ( 0.87 ) | 0.26 ( -0.28 ) |
| Swelling | 29 | 0.77 ( 0.53 - 1.13 ) | 0.77 ( 1.81 ) | 0.79 ( 0.57 ) | -0.34 ( -0.89 ) |
| Eczema | 29 | 1.72 ( 1.16 - 2.55 ) | 1.72 ( 7.53 ) | 1.62 ( 1.16 ) | 0.69 ( 0.13 ) |
| Abnormal Behaviour | 28 | 1.2 ( 0.81 - 1.77 ) | 1.2 ( 0.83 ) | 1.18 ( 0.85 ) | 0.24 ( -0.33 ) |
| Infant Irritability | 27 | 1.66 ( 1.11 - 2.49 ) | 1.66 ( 6.1 ) | 1.57 ( 1.12 ) | 0.65 ( 0.07 ) |
| Insomnia | 27 | 1.28 ( 0.86 - 1.9 ) | 1.28 ( 1.44 ) | 1.25 ( 0.89 ) | 0.32 ( -0.26 ) |
| C-Reactive Protein Increased | 27 | 0.92 ( 0.62 - 1.36 ) | 0.92 ( 0.19 ) | 0.92 ( 0.66 ) | -0.12 ( -0.68 ) |
| Rhinorrhoea | 26 | 0.7 ( 0.47 - 1.04 ) | 0.7 ( 3.24 ) | 0.72 ( 0.51 ) | -0.48 ( -1.06 ) |
| Musculoskeletal Stiffness | 26 | 1.49 ( 0.99 - 2.25 ) | 1.49 ( 3.7 ) | 1.43 ( 1.01 ) | 0.52 ( -0.07 ) |
| Restlessness | 26 | 0.85 ( 0.57 - 1.26 ) | 0.85 ( 0.68 ) | 0.86 ( 0.61 ) | -0.22 ( -0.8 ) |
| Skin Discolouration | 25 | 1.37 ( 0.91 - 2.09 ) | 1.37 ( 2.25 ) | 1.33 ( 0.94 ) | 0.41 ( -0.19 ) |
| Blood Culture Negative | 25 | 1.79 ( 1.17 - 2.74 ) | 1.79 ( 7.43 ) | 1.67 ( 1.17 ) | 0.74 ( 0.13 ) |
| Discomfort | 25 | 1.52 ( 1 - 2.31 ) | 1.52 ( 3.86 ) | 1.45 ( 1.02 ) | 0.54 ( -0.06 ) |
| Hypotonic-Hyporesponsive Episode | 25 | 0.6 ( 0.4 - 0.9 ) | 0.6 ( 6.26 ) | 0.62 ( 0.44 ) | -0.68 ( -1.26 ) |
| Influenza Virus Test Negative | 25 | 1.37 ( 0.9 - 2.08 ) | 1.37 ( 2.18 ) | 1.32 ( 0.93 ) | 0.41 ( -0.19 ) |
| Immediate Post-Injection Reaction | 24 | 0.96 ( 0.63 - 1.46 ) | 0.96 ( 0.04 ) | 0.96 ( 0.68 ) | -0.05 ( -0.66 ) |
| Peripheral Swelling | 24 | 1.05 ( 0.69 - 1.6 ) | 1.05 ( 0.05 ) | 1.04 ( 0.73 ) | 0.06 ( -0.54 ) |
| White Blood Cell Count Increased | 24 | 0.94 ( 0.62 - 1.43 ) | 0.94 ( 0.08 ) | 0.95 ( 0.67 ) | -0.08 ( -0.68 ) |
| Sars-Cov-2 Test Negative | 24 | 1.05 ( 0.69 - 1.6 ) | 1.05 ( 0.05 ) | 1.04 ( 0.73 ) | 0.06 ( -0.54 ) |
| Staring | 23 | 1.62 ( 1.05 - 2.52 ) | 1.62 ( 4.77 ) | 1.54 ( 1.07 ) | 0.62 ( -0.01 ) |
| Apnoea | 23 | 0.98 ( 0.64 - 1.5 ) | 0.98 ( 0.01 ) | 0.98 ( 0.68 ) | -0.03 ( -0.65 ) |
| Loss Of Consciousness | 23 | 0.77 ( 0.51 - 1.18 ) | 0.77 ( 1.44 ) | 0.79 ( 0.55 ) | -0.34 ( -0.95 ) |
| Electroencephalogram Abnormal | 22 | 2.4 ( 1.51 - 3.81 ) | 2.39 ( 14.53 ) | 2.13 ( 1.45 ) | 1.09 ( 0.44 ) |
| Respiratory Arrest | 21 | 1.47 ( 0.93 - 2.33 ) | 1.47 ( 2.79 ) | 1.41 ( 0.96 ) | 0.5 ( -0.15 ) |
| Platelet Count Increased | 21 | 2.47 ( 1.53 - 3.97 ) | 2.46 ( 14.8 ) | 2.18 ( 1.47 ) | 1.13 ( 0.45 ) |
| Hypersensitivity | 21 | 0.64 ( 0.41 - 0.99 ) | 0.64 ( 4.14 ) | 0.66 ( 0.45 ) | -0.61 ( -1.24 ) |
| Petechiae | 21 | 0.98 ( 0.63 - 1.54 ) | 0.98 ( 0.01 ) | 0.98 ( 0.68 ) | -0.02 ( -0.66 ) |
| Muscle Twitching | 21 | 2.01 ( 1.26 - 3.21 ) | 2.01 ( 8.97 ) | 1.85 ( 1.25 ) | 0.89 ( 0.22 ) |
| Feeling Abnormal | 20 | 1.67 ( 1.04 - 2.68 ) | 1.67 ( 4.65 ) | 1.58 ( 1.06 ) | 0.66 ( -0.02 ) |
| Condition Aggravated | 20 | 0.97 ( 0.61 - 1.53 ) | 0.97 ( 0.02 ) | 0.97 ( 0.66 ) | -0.04 ( -0.7 ) |
| Pruritus | 20 | 0.79 ( 0.5 - 1.24 ) | 0.79 ( 1.06 ) | 0.8 ( 0.55 ) | -0.32 ( -0.97 ) |
| Hypersomnia | 20 | 2.07 ( 1.28 - 3.35 ) | 2.07 ( 9.22 ) | 1.89 ( 1.27 ) | 0.92 ( 0.24 ) |
| Posture Abnormal | 20 | 2.35 ( 1.45 - 3.82 ) | 2.35 ( 12.63 ) | 2.1 ( 1.4 ) | 1.07 ( 0.38 ) |
| Sleep Disorder | 19 | 0.99 ( 0.62 - 1.59 ) | 0.99 ( 0 ) | 0.99 ( 0.67 ) | -0.01 ( -0.68 ) |
| Infantile Spitting Up | 19 | 3.26 ( 1.95 - 5.45 ) | 3.25 ( 22.62 ) | 2.72 ( 1.77 ) | 1.44 ( 0.72 ) |
| Seizure Like Phenomena | 18 | 2.21 ( 1.33 - 3.68 ) | 2.21 ( 9.87 ) | 2 ( 1.31 ) | 1 ( 0.28 ) |
| Hypophagia | 18 | 1.49 ( 0.91 - 2.45 ) | 1.49 ( 2.56 ) | 1.43 ( 0.95 ) | 0.52 ( -0.19 ) |
| Swelling Face | 18 | 1.33 ( 0.82 - 2.18 ) | 1.33 ( 1.33 ) | 1.3 ( 0.86 ) | 0.37 ( -0.33 ) |
| Mucous Stools | 18 | 1.19 ( 0.73 - 1.94 ) | 1.19 ( 0.49 ) | 1.17 ( 0.78 ) | 0.23 ( -0.47 ) |
| Viral Infection | 18 | 1.62 ( 0.99 - 2.67 ) | 1.62 ( 3.71 ) | 1.54 ( 1.01 ) | 0.62 ( -0.09 ) |
| Rash Generalised | 18 | 0.66 ( 0.41 - 1.07 ) | 0.66 ( 2.88 ) | 0.68 ( 0.46 ) | -0.55 ( -1.23 ) |
| Vaccination Complication | 17 | 1.04 ( 0.63 - 1.71 ) | 1.04 ( 0.02 ) | 1.03 ( 0.68 ) | 0.05 ( -0.66 ) |
| Pain In Extremity | 17 | 0.9 ( 0.55 - 1.47 ) | 0.9 ( 0.19 ) | 0.9 ( 0.6 ) | -0.14 ( -0.85 ) |
| Asthenia | 17 | 0.68 ( 0.42 - 1.11 ) | 0.68 ( 2.4 ) | 0.7 ( 0.46 ) | -0.51 ( -1.22 ) |
| Cardiac Arrest | 17 | 3.35 ( 1.94 - 5.79 ) | 3.35 ( 21.23 ) | 2.78 ( 1.76 ) | 1.47 ( 0.71 ) |
| Developmental Delay | 17 | 2.07 ( 1.23 - 3.48 ) | 2.06 ( 7.8 ) | 1.89 ( 1.22 ) | 0.92 ( 0.18 ) |
| Abdominal Pain | 17 | 0.91 ( 0.55 - 1.49 ) | 0.91 ( 0.15 ) | 0.91 ( 0.6 ) | -0.13 ( -0.84 ) |
| Emotional Distress | 17 | 1.6 ( 0.96 - 2.67 ) | 1.6 ( 3.32 ) | 1.52 ( 0.99 ) | 0.6 ( -0.12 ) |
| Poor Feeding Infant | 16 | 1.28 ( 0.76 - 2.14 ) | 1.28 ( 0.85 ) | 1.25 ( 0.81 ) | 0.32 ( -0.42 ) |
| Faeces Discoloured | 16 | 1.14 ( 0.68 - 1.92 ) | 1.14 ( 0.26 ) | 1.13 ( 0.73 ) | 0.18 ( -0.56 ) |
| Ear Infection | 16 | 0.86 ( 0.52 - 1.43 ) | 0.86 ( 0.33 ) | 0.87 ( 0.57 ) | -0.2 ( -0.93 ) |
| Local Reaction | 16 | 1.33 ( 0.79 - 2.23 ) | 1.33 ( 1.14 ) | 1.29 ( 0.83 ) | 0.37 ( -0.37 ) |
| Platelet Count Decreased | 16 | 0.61 ( 0.37 - 1.01 ) | 0.61 ( 3.84 ) | 0.63 ( 0.41 ) | -0.67 ( -1.39 ) |
| Differential White Blood Cell Count | 15 | 3.02 ( 1.7 - 5.36 ) | 3.01 ( 15.66 ) | 2.56 ( 1.58 ) | 1.36 ( 0.56 ) |
| Speech Disorder | 15 | 1.67 ( 0.97 - 2.88 ) | 1.67 ( 3.45 ) | 1.57 ( 1 ) | 0.66 ( -0.12 ) |
| Culture Urine Negative | 15 | 1.67 ( 0.97 - 2.88 ) | 1.67 ( 3.45 ) | 1.57 ( 1 ) | 0.66 ( -0.12 ) |
| Diet Refusal | 15 | 1.8 ( 1.04 - 3.12 ) | 1.8 ( 4.56 ) | 1.68 ( 1.06 ) | 0.75 ( -0.02 ) |
| Vomiting Projectile | 14 | 1.32 ( 0.76 - 2.3 ) | 1.32 ( 0.95 ) | 1.28 ( 0.8 ) | 0.36 ( -0.43 ) |
| Infantile Spasms | 14 | 2.81 ( 1.56 - 5.08 ) | 2.81 ( 12.88 ) | 2.43 ( 1.48 ) | 1.28 ( 0.45 ) |
| Muscle Spasms | 14 | 1.35 ( 0.78 - 2.36 ) | 1.35 ( 1.15 ) | 1.31 ( 0.82 ) | 0.39 ( -0.4 ) |
| Malaise | 14 | 0.45 ( 0.27 - 0.77 ) | 0.45 ( 8.83 ) | 0.48 ( 0.3 ) | -1.07 ( -1.83 ) |
| Injection Site Urticaria | 14 | 0.83 ( 0.48 - 1.42 ) | 0.83 ( 0.48 ) | 0.84 ( 0.53 ) | -0.25 ( -1.03 ) |
| Dehydration | 14 | 0.84 ( 0.49 - 1.45 ) | 0.84 ( 0.39 ) | 0.85 ( 0.54 ) | -0.23 ( -1.01 ) |
| Autism Spectrum Disorder | 14 | 0.96 ( 0.56 - 1.66 ) | 0.96 ( 0.02 ) | 0.97 ( 0.61 ) | -0.05 ( -0.83 ) |
| Constipation | 13 | 1.01 ( 0.57 - 1.78 ) | 1.01 ( 0 ) | 1.01 ( 0.62 ) | 0.01 ( -0.8 ) |
| Breath Holding | 13 | 2.26 ( 1.24 - 4.13 ) | 2.26 ( 7.53 ) | 2.04 ( 1.23 ) | 1.03 ( 0.19 ) |
| Respiratory Syncytial Virus Test Negative | 13 | 0.98 ( 0.56 - 1.74 ) | 0.98 ( 0 ) | 0.99 ( 0.61 ) | -0.02 ( -0.83 ) |
| Rash Pruritic | 13 | 0.99 ( 0.56 - 1.75 ) | 0.99 ( 0 ) | 0.99 ( 0.62 ) | -0.01 ( -0.82 ) |
| Vaccination Site Swelling | 13 | 0.82 ( 0.47 - 1.45 ) | 0.82 ( 0.46 ) | 0.84 ( 0.52 ) | -0.26 ( -1.06 ) |
| Wrong Drug Administered | 13 | 1.56 ( 0.87 - 2.8 ) | 1.56 ( 2.28 ) | 1.49 ( 0.91 ) | 0.57 ( -0.25 ) |
| Product Temperature Excursion Issue | 13 | 1.56 ( 0.87 - 2.8 ) | 1.56 ( 2.28 ) | 1.49 ( 0.91 ) | 0.57 ( -0.25 ) |
| Epilepsy | 12 | 0.99 ( 0.55 - 1.78 ) | 0.99 ( 0 ) | 0.99 ( 0.6 ) | -0.02 ( -0.85 ) |
| Immune Thrombocytopenia | 12 | 1.09 ( 0.6 - 1.98 ) | 1.09 ( 0.08 ) | 1.08 ( 0.66 ) | 0.11 ( -0.73 ) |
| Generalised Tonic-Clonic Seizure | 12 | 0.88 ( 0.49 - 1.59 ) | 0.88 ( 0.17 ) | 0.89 ( 0.54 ) | -0.17 ( -1 ) |
| Haemoglobin Decreased | 12 | 1.61 ( 0.88 - 2.95 ) | 1.61 ( 2.38 ) | 1.53 ( 0.92 ) | 0.61 ( -0.25 ) |
| Occult Blood Positive | 12 | 1.67 ( 0.91 - 3.08 ) | 1.67 ( 2.79 ) | 1.58 ( 0.95 ) | 0.66 ( -0.2 ) |
| Fontanelle Bulging | 12 | 3.92 ( 2.02 - 7.61 ) | 3.92 ( 18.96 ) | 3.12 ( 1.79 ) | 1.64 ( 0.73 ) |
| Urine Output Decreased | 12 | 2.92 ( 1.54 - 5.53 ) | 2.91 ( 11.8 ) | 2.5 ( 1.46 ) | 1.32 ( 0.43 ) |
| Eye Swelling | 12 | 1.47 ( 0.81 - 2.7 ) | 1.47 ( 1.61 ) | 1.42 ( 0.85 ) | 0.5 ( -0.35 ) |
| Anxiety | 12 | 0.99 ( 0.55 - 1.8 ) | 0.99 ( 0 ) | 1 ( 0.61 ) | -0.01 ( -0.84 ) |
| Injection Site Discolouration | 12 | 1.96 ( 1.06 - 3.63 ) | 1.96 ( 4.74 ) | 1.81 ( 1.08 ) | 0.85 ( -0.01 ) |
| Decreased Eye Contact | 11 | 0.7 ( 0.38 - 1.3 ) | 0.7 ( 1.28 ) | 0.72 ( 0.43 ) | -0.47 ( -1.33 ) |
| Mobility Decreased | 11 | 0.88 ( 0.47 - 1.62 ) | 0.88 ( 0.18 ) | 0.89 ( 0.53 ) | -0.17 ( -1.04 ) |
| Skin Lesion | 11 | 0.72 ( 0.39 - 1.32 ) | 0.72 ( 1.14 ) | 0.74 ( 0.44 ) | -0.44 ( -1.3 ) |
| Chills | 11 | 0.61 ( 0.33 - 1.12 ) | 0.61 ( 2.58 ) | 0.63 ( 0.38 ) | -0.66 ( -1.52 ) |
| Peripheral Coldness | 11 | 2.21 ( 1.15 - 4.24 ) | 2.21 ( 6.01 ) | 2 ( 1.16 ) | 1 ( 0.09 ) |
| Injection Site Inflammation | 11 | 2.05 ( 1.08 - 3.92 ) | 2.05 ( 4.96 ) | 1.88 ( 1.09 ) | 0.91 ( 0.01 ) |
| Balance Disorder | 11 | 1.55 ( 0.82 - 2.93 ) | 1.55 ( 1.88 ) | 1.48 ( 0.87 ) | 0.57 ( -0.32 ) |
| Vaccination Error | 11 | 1.85 ( 0.98 - 3.52 ) | 1.85 ( 3.67 ) | 1.72 ( 1.01 ) | 0.79 ( -0.11 ) |
| Diarrhoea Haemorrhagic | 11 | 1.29 ( 0.69 - 2.42 ) | 1.29 ( 0.64 ) | 1.26 ( 0.75 ) | 0.33 ( -0.55 ) |
| Allergy To Metals | 11 | 11.5 ( 4.88 - 27.08 ) | 11.49 ( 50.16 ) | 5.99 ( 2.93 ) | 2.58 ( 1.55 ) |
| Ultrasound Scan Abnormal | 11 | 1.13 ( 0.6 - 2.1 ) | 1.13 ( 0.14 ) | 1.11 ( 0.66 ) | 0.16 ( -0.72 ) |
| Injection Site Nodule | 11 | 0.75 ( 0.4 - 1.38 ) | 0.75 ( 0.89 ) | 0.76 ( 0.46 ) | -0.39 ( -1.25 ) |
| Rotavirus Test Positive | 11 | 0.36 ( 0.2 - 0.66 ) | 0.36 ( 11.79 ) | 0.39 ( 0.23 ) | -1.37 ( -2.22 ) |
| Injection Site Pruritus | 11 | 1.21 ( 0.65 - 2.26 ) | 1.21 ( 0.36 ) | 1.19 ( 0.7 ) | 0.25 ( -0.63 ) |
| Flushing | 10 | 1.31 ( 0.68 - 2.52 ) | 1.31 ( 0.64 ) | 1.27 ( 0.73 ) | 0.35 ( -0.57 ) |
| Sudden Infant Death Syndrome | 10 | 1.36 ( 0.7 - 2.62 ) | 1.36 ( 0.83 ) | 1.32 ( 0.76 ) | 0.4 ( -0.53 ) |
| Gait Inability | 10 | 0.77 ( 0.4 - 1.46 ) | 0.77 ( 0.65 ) | 0.78 ( 0.46 ) | -0.35 ( -1.25 ) |
| Bacterial Test Negative | 10 | 1.74 ( 0.89 - 3.4 ) | 1.74 ( 2.7 ) | 1.63 ( 0.93 ) | 0.71 ( -0.23 ) |
| Muscular Weakness | 10 | 1.45 ( 0.75 - 2.81 ) | 1.45 ( 1.23 ) | 1.4 ( 0.8 ) | 0.48 ( -0.45 ) |
| Scratch | 10 | 2.18 ( 1.1 - 4.3 ) | 2.18 ( 5.26 ) | 1.97 ( 1.12 ) | 0.98 ( 0.03 ) |
| Apathy | 10 | 0.87 ( 0.46 - 1.66 ) | 0.87 ( 0.18 ) | 0.88 ( 0.51 ) | -0.18 ( -1.09 ) |
| Leukocytosis | 10 | 1.77 ( 0.91 - 3.46 ) | 1.77 ( 2.87 ) | 1.66 ( 0.95 ) | 0.73 ( -0.21 ) |
| Pneumonia | 10 | 0.4 ( 0.21 - 0.74 ) | 0.4 ( 8.92 ) | 0.42 ( 0.25 ) | -1.26 ( -2.15 ) |
| Rash Maculo-Papular | 10 | 0.56 ( 0.29 - 1.05 ) | 0.56 ( 3.38 ) | 0.58 ( 0.34 ) | -0.79 ( -1.68 ) |
| Poor Quality Product Administered | 10 | 0.68 ( 0.36 - 1.29 ) | 0.68 ( 1.44 ) | 0.7 ( 0.41 ) | -0.52 ( -1.42 ) |
| Injection Site Bruising | 10 | 1.14 ( 0.59 - 2.18 ) | 1.14 ( 0.15 ) | 1.12 ( 0.65 ) | 0.17 ( -0.75 ) |
| Hyporesponsive To Stimuli | 10 | 0.84 ( 0.44 - 1.6 ) | 0.84 ( 0.27 ) | 0.85 ( 0.5 ) | -0.23 ( -1.13 ) |
| Vaccination Site Erythema | 10 | 0.61 ( 0.32 - 1.16 ) | 0.61 ( 2.35 ) | 0.63 ( 0.37 ) | -0.66 ( -1.56 ) |
| Oxygen Saturation Decreased | 10 | 1.27 ( 0.66 - 2.46 ) | 1.27 ( 0.52 ) | 1.24 ( 0.72 ) | 0.31 ( -0.61 ) |
| Aphasia | 9 | 0.76 ( 0.39 - 1.49 ) | 0.76 ( 0.65 ) | 0.77 ( 0.44 ) | -0.37 ( -1.32 ) |
| Dermatitis Diaper | 9 | 1.77 ( 0.87 - 3.6 ) | 1.77 ( 2.6 ) | 1.66 ( 0.92 ) | 0.73 ( -0.25 ) |
| Agitation | 9 | 1.01 ( 0.51 - 2 ) | 1.01 ( 0 ) | 1.01 ( 0.57 ) | 0.01 ( -0.94 ) |
| Kawasaki's Disease | 9 | 0.72 ( 0.36 - 1.41 ) | 0.72 ( 0.94 ) | 0.74 ( 0.42 ) | -0.44 ( -1.39 ) |
| Upper Respiratory Tract Infection | 9 | 0.59 ( 0.3 - 1.16 ) | 0.59 ( 2.41 ) | 0.61 ( 0.35 ) | -0.71 ( -1.65 ) |
| Viral Test Negative | 9 | 1.21 ( 0.6 - 2.4 ) | 1.21 ( 0.28 ) | 1.18 ( 0.66 ) | 0.24 ( -0.72 ) |
| Hyperhidrosis | 9 | 1.06 ( 0.53 - 2.1 ) | 1.06 ( 0.02 ) | 1.05 ( 0.59 ) | 0.07 ( -0.89 ) |
| Poor Quality Sleep | 9 | 1.03 ( 0.52 - 2.05 ) | 1.03 ( 0.01 ) | 1.03 ( 0.58 ) | 0.04 ( -0.91 ) |
| Respiratory Tract Congestion | 9 | 0.62 ( 0.32 - 1.21 ) | 0.62 ( 2 ) | 0.64 ( 0.36 ) | -0.64 ( -1.58 ) |
| Induration | 9 | 0.7 ( 0.35 - 1.37 ) | 0.7 ( 1.12 ) | 0.72 ( 0.41 ) | -0.48 ( -1.43 ) |
| Lip Swelling | 9 | 1.57 ( 0.78 - 3.16 ) | 1.57 ( 1.6 ) | 1.49 ( 0.83 ) | 0.58 ( -0.4 ) |
| Wheezing | 9 | 0.8 ( 0.4 - 1.57 ) | 0.8 ( 0.43 ) | 0.81 ( 0.46 ) | -0.3 ( -1.25 ) |
| Laboratory Test Abnormal | 9 | 1.36 ( 0.68 - 2.73 ) | 1.36 ( 0.77 ) | 1.32 ( 0.74 ) | 0.4 ( -0.57 ) |
| Anaemia | 9 | 1.57 ( 0.78 - 3.16 ) | 1.57 ( 1.6 ) | 1.49 ( 0.83 ) | 0.58 ( -0.4 ) |
| Thrombocytopenia | 9 | 0.81 ( 0.41 - 1.6 ) | 0.81 ( 0.37 ) | 0.82 ( 0.47 ) | -0.28 ( -1.23 ) |
| Product Administration Error | 9 | 0.85 ( 0.43 - 1.67 ) | 0.85 ( 0.23 ) | 0.86 ( 0.49 ) | -0.22 ( -1.17 ) |
| Incorrect Route Of Product Administration | 9 | 0.43 ( 0.22 - 0.84 ) | 0.43 ( 6.5 ) | 0.45 ( 0.26 ) | -1.14 ( -2.07 ) |
| Vaccination Site Oedema | 8 | 0.77 ( 0.37 - 1.57 ) | 0.77 ( 0.53 ) | 0.78 ( 0.43 ) | -0.35 ( -1.35 ) |
| Developmental Regression | 8 | 0.91 ( 0.44 - 1.87 ) | 0.91 ( 0.07 ) | 0.92 ( 0.5 ) | -0.13 ( -1.13 ) |
| Gaze Palsy | 8 | 0.55 ( 0.27 - 1.11 ) | 0.55 ( 2.87 ) | 0.57 ( 0.31 ) | -0.81 ( -1.8 ) |
| Weight Bearing Difficulty | 8 | 1.37 ( 0.66 - 2.86 ) | 1.37 ( 0.71 ) | 1.33 ( 0.72 ) | 0.41 ( -0.61 ) |
| Blood Potassium Increased | 8 | 2.39 ( 1.11 - 5.15 ) | 2.39 ( 5.25 ) | 2.13 ( 1.12 ) | 1.09 ( 0.03 ) |
| Blood Sodium Decreased | 8 | 1.71 ( 0.81 - 3.6 ) | 1.71 ( 2.01 ) | 1.61 ( 0.86 ) | 0.68 ( -0.35 ) |
| Gastrooesophageal Reflux Disease | 8 | 1.58 ( 0.75 - 3.32 ) | 1.58 ( 1.47 ) | 1.5 ( 0.81 ) | 0.59 ( -0.44 ) |
| Haematocrit Decreased | 8 | 1.1 ( 0.53 - 2.28 ) | 1.1 ( 0.07 ) | 1.09 ( 0.59 ) | 0.12 ( -0.89 ) |
| Food Refusal | 8 | 2.26 ( 1.05 - 4.85 ) | 2.26 ( 4.61 ) | 2.03 ( 1.07 ) | 1.02 ( -0.03 ) |
| Abnormal Faeces | 8 | 1.13 ( 0.54 - 2.34 ) | 1.13 ( 0.11 ) | 1.12 ( 0.61 ) | 0.16 ( -0.85 ) |
| Strabismus | 8 | 1.67 ( 0.79 - 3.53 ) | 1.67 ( 1.86 ) | 1.58 ( 0.85 ) | 0.66 ( -0.37 ) |
| Vaccination Site Reaction | 8 | 0.55 ( 0.27 - 1.12 ) | 0.55 ( 2.81 ) | 0.57 ( 0.32 ) | -0.81 ( -1.79 ) |
| Decreased Activity | 8 | 1.19 ( 0.57 - 2.48 ) | 1.19 ( 0.23 ) | 1.17 ( 0.64 ) | 0.23 ( -0.78 ) |
| Infection | 7 | 0.8 ( 0.37 - 1.73 ) | 0.8 ( 0.31 ) | 0.82 ( 0.43 ) | -0.29 ( -1.35 ) |
| Inflammation | 7 | 0.75 ( 0.35 - 1.62 ) | 0.75 ( 0.53 ) | 0.77 ( 0.41 ) | -0.38 ( -1.44 ) |
| Purpura | 7 | 1.12 ( 0.52 - 2.45 ) | 1.12 ( 0.09 ) | 1.11 ( 0.58 ) | 0.15 ( -0.92 ) |
| Erythema Multiforme | 7 | 0.84 ( 0.39 - 1.82 ) | 0.84 ( 0.2 ) | 0.85 ( 0.45 ) | -0.23 ( -1.29 ) |
| Respiratory Failure | 7 | 1.78 ( 0.8 - 3.98 ) | 1.78 ( 2.06 ) | 1.67 ( 0.85 ) | 0.74 ( -0.36 ) |
| Choking | 7 | 0.89 ( 0.41 - 1.93 ) | 0.89 ( 0.09 ) | 0.9 ( 0.47 ) | -0.15 ( -1.22 ) |
| Blood Glucose Increased | 7 | 1.03 ( 0.47 - 2.24 ) | 1.03 ( 0.01 ) | 1.03 ( 0.54 ) | 0.04 ( -1.03 ) |
| Fall | 7 | 0.88 ( 0.41 - 1.91 ) | 0.88 ( 0.1 ) | 0.89 ( 0.47 ) | -0.17 ( -1.23 ) |
| Blood Creatinine Decreased | 7 | 2.22 ( 0.98 - 5.01 ) | 2.22 ( 3.85 ) | 2 ( 1.01 ) | 1 ( -0.11 ) |
| Lymphadenopathy | 7 | 0.41 ( 0.19 - 0.88 ) | 0.41 ( 5.62 ) | 0.44 ( 0.23 ) | -1.2 ( -2.24 ) |
| Neutrophil Percentage Decreased | 7 | 0.67 ( 0.31 - 1.44 ) | 0.67 ( 1.06 ) | 0.69 ( 0.36 ) | -0.53 ( -1.59 ) |
| Lymphocyte Percentage Increased | 7 | 0.76 ( 0.35 - 1.64 ) | 0.76 ( 0.49 ) | 0.78 ( 0.41 ) | -0.36 ( -1.42 ) |
| Skin Test Positive | 7 | 6.1 ( 2.4 - 15.49 ) | 6.09 ( 18.82 ) | 4.22 ( 1.93 ) | 2.08 ( 0.87 ) |
| Chest X-Ray Abnormal | 7 | 0.74 ( 0.34 - 1.59 ) | 0.74 ( 0.61 ) | 0.76 ( 0.4 ) | -0.4 ( -1.46 ) |
| Pulse Absent | 7 | 3.66 ( 1.55 - 8.65 ) | 3.66 ( 10 ) | 2.97 ( 1.44 ) | 1.57 ( 0.41 ) |
| Injection Site Oedema | 7 | 0.75 ( 0.35 - 1.62 ) | 0.75 ( 0.53 ) | 0.77 ( 0.41 ) | -0.38 ( -1.44 ) |
| Injection Site Cellulitis | 7 | 0.73 ( 0.34 - 1.57 ) | 0.73 ( 0.65 ) | 0.75 ( 0.39 ) | -0.42 ( -1.48 ) |
| Hemiparesis | 7 | 4.3 ( 1.78 - 10.38 ) | 4.3 ( 12.56 ) | 3.34 ( 1.6 ) | 1.74 ( 0.57 ) |
| Hypertonia | 7 | 0.79 ( 0.37 - 1.71 ) | 0.79 ( 0.35 ) | 0.81 ( 0.43 ) | -0.31 ( -1.37 ) |
| Postictal State | 7 | 1.7 ( 0.76 - 3.78 ) | 1.7 ( 1.74 ) | 1.6 ( 0.82 ) | 0.68 ( -0.42 ) |
| Frequent Bowel Movements | 7 | 1.2 ( 0.55 - 2.62 ) | 1.2 ( 0.21 ) | 1.18 ( 0.61 ) | 0.24 ( -0.84 ) |
| Dry Skin | 7 | 1.35 ( 0.62 - 2.98 ) | 1.35 ( 0.57 ) | 1.31 ( 0.68 ) | 0.39 ( -0.69 ) |
| Granuloma | 7 | 5.22 ( 2.11 - 12.95 ) | 5.22 ( 15.93 ) | 3.81 ( 1.79 ) | 1.93 ( 0.74 ) |
| Food Allergy | 7 | 1.2 ( 0.55 - 2.62 ) | 1.2 ( 0.21 ) | 1.18 ( 0.61 ) | 0.24 ( -0.84 ) |
| Tenderness | 6 | 0.94 ( 0.41 - 2.16 ) | 0.94 ( 0.02 ) | 0.94 ( 0.47 ) | -0.09 ( -1.23 ) |
| Cellulitis | 6 | 0.43 ( 0.19 - 0.97 ) | 0.43 ( 4.38 ) | 0.45 ( 0.23 ) | -1.15 ( -2.26 ) |
| Alanine Aminotransferase Increased | 6 | 1.01 ( 0.44 - 2.34 ) | 1.01 ( 0 ) | 1.01 ( 0.5 ) | 0.01 ( -1.13 ) |
| Neutropenia | 6 | 1.04 ( 0.45 - 2.42 ) | 1.04 ( 0.01 ) | 1.04 ( 0.52 ) | 0.06 ( -1.09 ) |
| Eating Disorder | 6 | 2.09 ( 0.87 - 5.02 ) | 2.09 ( 2.84 ) | 1.91 ( 0.92 ) | 0.93 ( -0.26 ) |
| Flatulence | 6 | 0.82 ( 0.36 - 1.89 ) | 0.82 ( 0.21 ) | 0.84 ( 0.42 ) | -0.26 ( -1.39 ) |
| Barium Double Contrast | 6 | 1.23 ( 0.53 - 2.86 ) | 1.23 ( 0.23 ) | 1.2 ( 0.59 ) | 0.27 ( -0.89 ) |
| Blood Test Abnormal | 6 | 0.95 ( 0.41 - 2.19 ) | 0.95 ( 0.01 ) | 0.95 ( 0.47 ) | -0.07 ( -1.21 ) |
| Joint Swelling | 6 | 1.74 ( 0.73 - 4.13 ) | 1.74 ( 1.62 ) | 1.63 ( 0.79 ) | 0.71 ( -0.47 ) |
| Weight Decreased | 6 | 0.99 ( 0.43 - 2.3 ) | 0.99 ( 0 ) | 1 ( 0.49 ) | -0.01 ( -1.15 ) |
| Respiration Abnormal | 6 | 1.16 ( 0.5 - 2.7 ) | 1.16 ( 0.12 ) | 1.14 ( 0.56 ) | 0.19 ( -0.96 ) |
| Middle Insomnia | 6 | 1.18 ( 0.51 - 2.75 ) | 1.18 ( 0.15 ) | 1.16 ( 0.57 ) | 0.22 ( -0.93 ) |
| Oedema Peripheral | 6 | 1.04 ( 0.45 - 2.42 ) | 1.04 ( 0.01 ) | 1.04 ( 0.52 ) | 0.06 ( -1.09 ) |
| Nuclear Magnetic Resonance Imaging Brain Abnormal | 6 | 4.48 ( 1.72 - 11.65 ) | 4.48 ( 11.34 ) | 3.43 ( 1.54 ) | 1.78 ( 0.52 ) |
| Polymerase Chain Reaction Positive | 6 | 0.45 ( 0.2 - 1.02 ) | 0.45 ( 3.85 ) | 0.47 ( 0.24 ) | -1.08 ( -2.2 ) |
| Food Intolerance | 6 | 3.69 ( 1.45 - 9.35 ) | 3.69 ( 8.68 ) | 2.99 ( 1.37 ) | 1.58 ( 0.34 ) |
| Movement Disorder | 5 | 1.27 ( 0.5 - 3.22 ) | 1.27 ( 0.26 ) | 1.24 ( 0.57 ) | 0.31 ( -0.94 ) |
| Ocular Hyperaemia | 5 | 0.89 ( 0.36 - 2.21 ) | 0.89 ( 0.07 ) | 0.89 ( 0.42 ) | -0.16 ( -1.39 ) |
| Neutrophil Count Increased | 5 | 0.89 ( 0.36 - 2.21 ) | 0.89 ( 0.07 ) | 0.89 ( 0.42 ) | -0.16 ( -1.39 ) |
| Body Temperature Decreased | 5 | 1.21 ( 0.48 - 3.07 ) | 1.21 ( 0.17 ) | 1.19 ( 0.55 ) | 0.25 ( -0.99 ) |
| X-Ray Abnormal | 5 | 1.14 ( 0.45 - 2.86 ) | 1.14 ( 0.07 ) | 1.12 ( 0.52 ) | 0.17 ( -1.08 ) |
| Magnetic Resonance Imaging Head Abnormal | 5 | 0.99 ( 0.39 - 2.47 ) | 0.99 ( 0 ) | 0.99 ( 0.46 ) | -0.02 ( -1.26 ) |
| General Physical Health Deterioration | 5 | 0.84 ( 0.34 - 2.1 ) | 0.84 ( 0.14 ) | 0.85 ( 0.4 ) | -0.23 ( -1.46 ) |
| Bronchitis | 5 | 0.64 ( 0.26 - 1.59 ) | 0.64 ( 0.92 ) | 0.67 ( 0.31 ) | -0.59 ( -1.81 ) |
| Syringe Issue | 5 | 0.89 ( 0.36 - 2.21 ) | 0.89 ( 0.07 ) | 0.89 ( 0.42 ) | -0.16 ( -1.39 ) |
| Full Blood Count Abnormal | 5 | 1.27 ( 0.5 - 3.22 ) | 1.27 ( 0.26 ) | 1.24 ( 0.57 ) | 0.31 ( -0.94 ) |
| Tic | 5 | 1.8 ( 0.7 - 4.65 ) | 1.8 ( 1.52 ) | 1.68 ( 0.76 ) | 0.75 ( -0.52 ) |
| Meningitis | 5 | 0.9 ( 0.36 - 2.24 ) | 0.9 ( 0.05 ) | 0.91 ( 0.42 ) | -0.14 ( -1.37 ) |
| Streptococcus Test Negative | 5 | 0.45 ( 0.18 - 1.09 ) | 0.45 ( 3.3 ) | 0.47 ( 0.22 ) | -1.09 ( -2.3 ) |
| Influenza | 5 | 1 ( 0.4 - 2.51 ) | 1 ( 0 ) | 1 ( 0.47 ) | 0.01 ( -1.23 ) |
| Retching | 5 | 1.16 ( 0.46 - 2.92 ) | 1.16 ( 0.1 ) | 1.14 ( 0.53 ) | 0.19 ( -1.05 ) |
| Regressive Behaviour | 5 | 1.27 ( 0.5 - 3.22 ) | 1.27 ( 0.26 ) | 1.24 ( 0.57 ) | 0.31 ( -0.94 ) |
| Lymphocyte Count Increased | 5 | 0.78 ( 0.31 - 1.93 ) | 0.78 ( 0.29 ) | 0.79 ( 0.37 ) | -0.33 ( -1.56 ) |
| Heart Rate Increased | 5 | 0.87 ( 0.35 - 2.17 ) | 0.87 ( 0.09 ) | 0.88 ( 0.41 ) | -0.18 ( -1.41 ) |
| Appetite Disorder | 5 | 4.35 ( 1.53 - 12.36 ) | 4.35 ( 9.11 ) | 3.37 ( 1.41 ) | 1.75 ( 0.39 ) |
| Rash Vesicular | 5 | 0.29 ( 0.12 - 0.7 ) | 0.29 ( 8.64 ) | 0.31 ( 0.15 ) | -1.71 ( -2.91 ) |
| High-Pitched Crying | 5 | 1.19 ( 0.47 - 2.99 ) | 1.19 ( 0.13 ) | 1.17 ( 0.54 ) | 0.22 ( -1.02 ) |
| Abdominal Distension | 5 | 0.77 ( 0.31 - 1.9 ) | 0.77 ( 0.33 ) | 0.78 ( 0.37 ) | -0.35 ( -1.58 ) |
| Mean Cell Volume Decreased | 5 | 0.83 ( 0.33 - 2.06 ) | 0.83 ( 0.16 ) | 0.84 ( 0.39 ) | -0.25 ( -1.48 ) |
| Drug Ineffective | 5 | 1.63 ( 0.64 - 4.19 ) | 1.63 ( 1.06 ) | 1.55 ( 0.7 ) | 0.63 ( -0.64 ) |
| Monocyte Percentage Increased | 5 | 0.95 ( 0.38 - 2.37 ) | 0.95 ( 0.01 ) | 0.95 ( 0.44 ) | -0.07 ( -1.3 ) |
| Nausea | 5 | 0.43 ( 0.18 - 1.06 ) | 0.44 ( 3.52 ) | 0.46 ( 0.22 ) | -1.13 ( -2.33 ) |
| Infantile Vomiting | 5 | 1 ( 0.4 - 2.51 ) | 1 ( 0 ) | 1 ( 0.47 ) | 0.01 ( -1.23 ) |
| Blood Lactate Dehydrogenase Increased | 5 | 0.86 ( 0.34 - 2.13 ) | 0.86 ( 0.11 ) | 0.87 ( 0.4 ) | -0.21 ( -1.44 ) |
| Grunting | 5 | 1.37 ( 0.54 - 3.49 ) | 1.37 ( 0.45 ) | 1.33 ( 0.61 ) | 0.41 ( -0.84 ) |
| Tachycardia | 5 | 0.45 ( 0.19 - 1.11 ) | 0.45 ( 3.15 ) | 0.48 ( 0.23 ) | -1.07 ( -2.28 ) |
| Immunisation Reaction | 5 | 0.58 ( 0.24 - 1.43 ) | 0.58 ( 1.44 ) | 0.6 ( 0.28 ) | -0.73 ( -1.95 ) |
| Contusion | 5 | 0.47 ( 0.19 - 1.16 ) | 0.47 ( 2.78 ) | 0.5 ( 0.24 ) | -1.01 ( -2.22 ) |
| Swelling Of Eyelid | 5 | 1.37 ( 0.54 - 3.49 ) | 1.37 ( 0.45 ) | 1.33 ( 0.61 ) | 0.41 ( -0.84 ) |
| Dizziness | 5 | 0.79 ( 0.32 - 1.96 ) | 0.79 ( 0.26 ) | 0.81 ( 0.38 ) | -0.31 ( -1.54 ) |
| Occult Blood Negative | 5 | 2.27 ( 0.86 - 5.97 ) | 2.27 ( 2.92 ) | 2.04 ( 0.91 ) | 1.03 ( -0.26 ) |
| Nasopharyngitis | 5 | 0.44 ( 0.18 - 1.08 ) | 0.44 ( 3.37 ) | 0.47 ( 0.22 ) | -1.1 ( -2.31 ) |
| Product Administered At Inappropriate Site | 5 | 0.87 ( 0.35 - 2.17 ) | 0.87 ( 0.09 ) | 0.88 ( 0.41 ) | -0.18 ( -1.41 ) |
| Abdominal Pain Upper | 5 | 0.77 ( 0.31 - 1.9 ) | 0.77 ( 0.33 ) | 0.78 ( 0.37 ) | -0.35 ( -1.58 ) |
| Thrombocytopenic Purpura | 5 | 0.25 ( 0.1 - 0.6 ) | 0.25 ( 11.1 ) | 0.27 ( 0.13 ) | -1.91 ( -3.1 ) |
| Nystagmus | 5 | 2.61 ( 0.98 - 6.96 ) | 2.61 ( 3.98 ) | 2.29 ( 1.01 ) | 1.19 ( -0.11 ) |
| Echocardiogram Abnormal | 5 | 1.45 ( 0.57 - 3.7 ) | 1.45 ( 0.61 ) | 1.4 ( 0.64 ) | 0.48 ( -0.78 ) |
| Blister | 5 | 0.35 ( 0.14 - 0.86 ) | 0.35 ( 5.75 ) | 0.37 ( 0.18 ) | -1.42 ( -2.62 ) |
| Injection Site Vesicles | 5 | 1.04 ( 0.42 - 2.62 ) | 1.04 ( 0.01 ) | 1.04 ( 0.48 ) | 0.06 ( -1.18 ) |
| Speech Disorder Developmental | 5 | 0.54 ( 0.22 - 1.34 ) | 0.54 ( 1.82 ) | 0.57 ( 0.27 ) | -0.82 ( -2.03 ) |
| Partial Seizures | 5 | 1.02 ( 0.41 - 2.57 ) | 1.02 ( 0 ) | 1.02 ( 0.47 ) | 0.03 ( -1.21 ) |
| Respiratory Tract Infection | 5 | 0.93 ( 0.37 - 2.33 ) | 0.93 ( 0.02 ) | 0.94 ( 0.44 ) | -0.09 ( -1.33 ) |
| Injection Site Granuloma | 5 | 5.22 ( 1.79 - 15.29 ) | 5.22 ( 11.38 ) | 3.81 ( 1.55 ) | 1.93 ( 0.55 ) |
| Injection Site Haemorrhage | 5 | 2.27 ( 0.86 - 5.97 ) | 2.27 ( 2.92 ) | 2.04 ( 0.91 ) | 1.03 ( -0.26 ) |
| Headache | 5 | 0.32 ( 0.13 - 0.77 ) | 0.32 ( 7.17 ) | 0.34 ( 0.16 ) | -1.57 ( -2.77 ) |
| Sepsis | 4 | 0.27 ( 0.1 - 0.73 ) | 0.27 ( 7.73 ) | 0.29 ( 0.13 ) | -1.8 ( -3.11 ) |
| Nervousness | 4 | 0.8 ( 0.29 - 2.22 ) | 0.8 ( 0.18 ) | 0.82 ( 0.35 ) | -0.29 ( -1.64 ) |
| Illness | 4 | 1.19 ( 0.42 - 3.36 ) | 1.19 ( 0.11 ) | 1.17 ( 0.49 ) | 0.23 ( -1.14 ) |
| Mental Status Changes | 4 | 1.67 ( 0.58 - 4.8 ) | 1.67 ( 0.93 ) | 1.58 ( 0.65 ) | 0.66 ( -0.74 ) |
| Epistaxis | 4 | 0.79 ( 0.29 - 2.18 ) | 0.79 ( 0.21 ) | 0.8 ( 0.34 ) | -0.32 ( -1.66 ) |
| Aspartate Aminotransferase Increased | 4 | 0.51 ( 0.19 - 1.39 ) | 0.51 ( 1.8 ) | 0.53 ( 0.23 ) | -0.91 ( -2.24 ) |
| Atrial Septal Defect | 4 | 3.48 ( 1.12 - 10.8 ) | 3.48 ( 5.31 ) | 2.86 ( 1.11 ) | 1.52 ( 0.05 ) |
| Drooling | 4 | 0.79 ( 0.29 - 2.18 ) | 0.79 ( 0.21 ) | 0.8 ( 0.34 ) | -0.32 ( -1.66 ) |
| Motor Dysfunction | 4 | 1.99 ( 0.68 - 5.8 ) | 1.99 ( 1.65 ) | 1.83 ( 0.75 ) | 0.87 ( -0.54 ) |
| Anaphylactic Reaction | 4 | 0.35 ( 0.13 - 0.96 ) | 0.35 ( 4.56 ) | 0.38 ( 0.16 ) | -1.41 ( -2.73 ) |
| Blood Albumin Decreased | 4 | 1.35 ( 0.48 - 3.82 ) | 1.35 ( 0.32 ) | 1.31 ( 0.55 ) | 0.39 ( -0.99 ) |
| Rotavirus Test Negative | 4 | 0.93 ( 0.33 - 2.58 ) | 0.93 ( 0.02 ) | 0.93 ( 0.4 ) | -0.1 ( -1.45 ) |
| Csf Culture Negative | 4 | 0.97 ( 0.35 - 2.71 ) | 0.97 ( 0 ) | 0.97 ( 0.41 ) | -0.04 ( -1.39 ) |
| Sneezing | 4 | 1.31 ( 0.46 - 3.69 ) | 1.31 ( 0.25 ) | 1.27 ( 0.53 ) | 0.35 ( -1.03 ) |
| Urine Analysis Abnormal | 4 | 1.35 ( 0.48 - 3.82 ) | 1.35 ( 0.32 ) | 1.31 ( 0.55 ) | 0.39 ( -0.99 ) |
| Urine Ketone Body Present | 4 | 2.79 ( 0.92 - 8.39 ) | 2.79 ( 3.61 ) | 2.41 ( 0.96 ) | 1.27 ( -0.17 ) |
| Fluid Intake Reduced | 4 | 0.91 ( 0.33 - 2.52 ) | 0.91 ( 0.03 ) | 0.92 ( 0.39 ) | -0.13 ( -1.48 ) |
| Muscle Rigidity | 4 | 0.8 ( 0.29 - 2.22 ) | 0.8 ( 0.18 ) | 0.82 ( 0.35 ) | -0.29 ( -1.64 ) |
| Encephalopathy | 4 | 0.64 ( 0.23 - 1.76 ) | 0.64 ( 0.75 ) | 0.66 ( 0.28 ) | -0.59 ( -1.93 ) |
| Status Epilepticus | 4 | 0.58 ( 0.21 - 1.59 ) | 0.58 ( 1.15 ) | 0.6 ( 0.26 ) | -0.73 ( -2.06 ) |
| Rhinitis | 4 | 0.75 ( 0.27 - 2.06 ) | 0.75 ( 0.32 ) | 0.76 ( 0.33 ) | -0.39 ( -1.73 ) |
| Opisthotonus | 4 | 0.67 ( 0.25 - 1.85 ) | 0.67 ( 0.59 ) | 0.69 ( 0.3 ) | -0.53 ( -1.87 ) |
| Eye Discharge | 4 | 1.35 ( 0.48 - 3.82 ) | 1.35 ( 0.32 ) | 1.31 ( 0.55 ) | 0.39 ( -0.99 ) |
| White Blood Cell Count Decreased | 4 | 0.5 ( 0.18 - 1.36 ) | 0.5 ( 1.94 ) | 0.52 ( 0.22 ) | -0.94 ( -2.27 ) |
| Depressed Level Of Consciousness | 4 | 0.45 ( 0.17 - 1.24 ) | 0.45 ( 2.52 ) | 0.48 ( 0.21 ) | -1.07 ( -2.39 ) |
| Pharyngeal Erythema | 4 | 0.44 ( 0.16 - 1.2 ) | 0.44 ( 2.74 ) | 0.46 ( 0.2 ) | -1.11 ( -2.44 ) |
| Respiratory Rate Increased | 4 | 0.8 ( 0.29 - 2.22 ) | 0.8 ( 0.18 ) | 0.82 ( 0.35 ) | -0.29 ( -1.64 ) |
| Nervous System Disorder | 4 | 1.19 ( 0.42 - 3.36 ) | 1.19 ( 0.11 ) | 1.17 ( 0.49 ) | 0.23 ( -1.14 ) |
| Neutrophil Count Decreased | 4 | 0.52 ( 0.19 - 1.41 ) | 0.52 ( 1.73 ) | 0.54 ( 0.23 ) | -0.89 ( -2.22 ) |
| Influenza Like Illness | 4 | 1.44 ( 0.51 - 4.1 ) | 1.44 ( 0.47 ) | 1.39 ( 0.58 ) | 0.47 ( -0.91 ) |
| Incomplete Course Of Vaccination | 4 | 0.87 ( 0.31 - 2.41 ) | 0.87 ( 0.07 ) | 0.88 ( 0.37 ) | -0.18 ( -1.53 ) |
| Human Rhinovirus Test Positive | 4 | 0.77 ( 0.28 - 2.14 ) | 0.77 ( 0.25 ) | 0.79 ( 0.34 ) | -0.34 ( -1.69 ) |
| Otitis Media Acute | 4 | 1.19 ( 0.42 - 3.36 ) | 1.19 ( 0.11 ) | 1.17 ( 0.49 ) | 0.23 ( -1.14 ) |
| Scar | 4 | 3.21 ( 1.05 - 9.86 ) | 3.21 ( 4.66 ) | 2.69 ( 1.05 ) | 1.43 ( -0.03 ) |
| Red Cell Distribution Width Increased | 4 | 2.09 ( 0.71 - 6.11 ) | 2.09 ( 1.89 ) | 1.91 ( 0.78 ) | 0.93 ( -0.48 ) |
| Hyperaesthesia | 4 | 1.61 ( 0.56 - 4.61 ) | 1.61 ( 0.79 ) | 1.53 ( 0.63 ) | 0.61 ( -0.78 ) |
| Viral Upper Respiratory Tract Infection | 4 | 3.21 ( 1.05 - 9.86 ) | 3.21 ( 4.66 ) | 2.69 ( 1.05 ) | 1.43 ( -0.03 ) |
| Mean Cell Haemoglobin Decreased | 4 | 0.8 ( 0.29 - 2.22 ) | 0.8 ( 0.18 ) | 0.82 ( 0.35 ) | -0.29 ( -1.64 ) |
| Breath Sounds Abnormal | 4 | 0.65 ( 0.24 - 1.79 ) | 0.65 ( 0.7 ) | 0.67 ( 0.29 ) | -0.57 ( -1.91 ) |
| Needle Issue | 4 | 1.27 ( 0.45 - 3.57 ) | 1.27 ( 0.2 ) | 1.24 ( 0.52 ) | 0.31 ( -1.07 ) |
| Red Blood Cell Sedimentation Rate | 4 | 1.67 ( 0.58 - 4.8 ) | 1.67 ( 0.93 ) | 1.58 ( 0.65 ) | 0.66 ( -0.74 ) |
| Vaccination Site Pain | 4 | 0.36 ( 0.13 - 0.97 ) | 0.36 ( 4.48 ) | 0.38 ( 0.16 ) | -1.4 ( -2.72 ) |
| Vaccination Site Warmth | 4 | 0.82 ( 0.3 - 2.27 ) | 0.82 ( 0.15 ) | 0.83 ( 0.36 ) | -0.26 ( -1.61 ) |
| Stridor | 4 | 1.9 ( 0.65 - 5.51 ) | 1.9 ( 1.44 ) | 1.76 ( 0.72 ) | 0.82 ( -0.59 ) |
| Foaming At Mouth | 4 | 0.97 ( 0.35 - 2.71 ) | 0.97 ( 0 ) | 0.97 ( 0.41 ) | -0.04 ( -1.39 ) |
| Catarrh | 4 | 1.49 ( 0.52 - 4.25 ) | 1.49 ( 0.57 ) | 1.43 ( 0.6 ) | 0.52 ( -0.87 ) |
| Gastrointestinal Disorder | 4 | 0.57 ( 0.21 - 1.57 ) | 0.57 ( 1.21 ) | 0.59 ( 0.26 ) | -0.75 ( -2.08 ) |
| Procalcitonin Increased | 4 | 0.95 ( 0.34 - 2.64 ) | 0.95 ( 0.01 ) | 0.95 ( 0.4 ) | -0.07 ( -1.42 ) |
| Vaccination Site Bruising | 4 | 3.48 ( 1.12 - 10.8 ) | 3.48 ( 5.31 ) | 2.86 ( 1.11 ) | 1.52 ( 0.05 ) |
| Vaccination Site Induration | 4 | 0.51 ( 0.19 - 1.39 ) | 0.51 ( 1.8 ) | 0.53 ( 0.23 ) | -0.91 ( -2.24 ) |
| Injection Site Abscess | 4 | 0.93 ( 0.33 - 2.58 ) | 0.93 ( 0.02 ) | 0.93 ( 0.4 ) | -0.1 ( -1.45 ) |
| Syncope | 4 | 0.28 ( 0.1 - 0.76 ) | 0.28 ( 7.12 ) | 0.3 ( 0.13 ) | -1.73 ( -3.04 ) |
| Infantile Apnoea | 4 | 1.1 ( 0.39 - 3.08 ) | 1.1 ( 0.03 ) | 1.09 ( 0.46 ) | 0.12 ( -1.24 ) |
| Feeding Disorder | 4 | 0.84 ( 0.3 - 2.31 ) | 0.84 ( 0.12 ) | 0.85 ( 0.36 ) | -0.24 ( -1.59 ) |
| Psychomotor Hyperactivity | 4 | 1.82 ( 0.63 - 5.25 ) | 1.82 ( 1.25 ) | 1.7 ( 0.7 ) | 0.76 ( -0.64 ) |
| Head Banging | 4 | 0.97 ( 0.35 - 2.71 ) | 0.97 ( 0 ) | 0.97 ( 0.41 ) | -0.04 ( -1.39 ) |
| Gastroenteritis | 4 | 0.28 ( 0.1 - 0.76 ) | 0.28 ( 7.12 ) | 0.3 ( 0.13 ) | -1.73 ( -3.04 ) |
| Eye Infection | 4 | 8.36 ( 2.24 - 31.13 ) | 8.36 ( 14.39 ) | 5.09 ( 1.69 ) | 2.35 ( 0.77 ) |
| Hair Growth Abnormal | 4 | 13.93 ( 3.12 - 62.25 ) | 13.93 ( 20.57 ) | 6.54 ( 1.87 ) | 2.71 ( 1.06 ) |
| Excessive Eye Blinking | 4 | 2.32 ( 0.79 - 6.86 ) | 2.32 ( 2.46 ) | 2.08 ( 0.84 ) | 1.06 ( -0.37 ) |
| Listless | 4 | 0.6 ( 0.22 - 1.63 ) | 0.6 ( 1.03 ) | 0.62 ( 0.27 ) | -0.69 ( -2.03 ) |
| Injection Site Papule | 4 | 1.61 ( 0.56 - 4.61 ) | 1.61 ( 0.79 ) | 1.53 ( 0.63 ) | 0.61 ( -0.78 ) |
| Tearfulness | 3 | 0.56 ( 0.18 - 1.79 ) | 0.56 ( 0.99 ) | 0.58 ( 0.22 ) | -0.78 ( -2.27 ) |
| Varicella | 3 | 0.54 ( 0.17 - 1.72 ) | 0.54 ( 1.12 ) | 0.56 ( 0.21 ) | -0.83 ( -2.32 ) |
| Thrombocytosis | 3 | 2.85 ( 0.79 - 10.21 ) | 2.85 ( 2.83 ) | 2.45 ( 0.84 ) | 1.29 ( -0.33 ) |
| Coma | 3 | 2.24 ( 0.64 - 7.79 ) | 2.24 ( 1.69 ) | 2.02 ( 0.71 ) | 1.01 ( -0.58 ) |
| General Physical Condition Abnormal | 3 | 2.24 ( 0.64 - 7.79 ) | 2.24 ( 1.69 ) | 2.02 ( 0.71 ) | 1.01 ( -0.58 ) |
| Culture Stool Negative | 3 | 0.87 ( 0.27 - 2.83 ) | 0.87 ( 0.05 ) | 0.88 ( 0.33 ) | -0.18 ( -1.7 ) |
| Skin Test Negative | 3 | 2.61 ( 0.74 - 9.26 ) | 2.61 ( 2.39 ) | 2.29 ( 0.79 ) | 1.19 ( -0.42 ) |
| Basophil Percentage Decreased | 3 | 0.85 ( 0.26 - 2.75 ) | 0.85 ( 0.08 ) | 0.86 ( 0.32 ) | -0.22 ( -1.73 ) |
| Eosinophil Percentage Decreased | 3 | 0.8 ( 0.25 - 2.6 ) | 0.8 ( 0.13 ) | 0.82 ( 0.31 ) | -0.29 ( -1.8 ) |
| Protein Total Decreased | 3 | 1.42 ( 0.43 - 4.76 ) | 1.42 ( 0.33 ) | 1.37 ( 0.5 ) | 0.46 ( -1.09 ) |
| Blood Bilirubin Decreased | 3 | 2.61 ( 0.74 - 9.26 ) | 2.61 ( 2.39 ) | 2.29 ( 0.79 ) | 1.19 ( -0.42 ) |
| Periorbital Swelling | 3 | 1.08 ( 0.33 - 3.55 ) | 1.08 ( 0.02 ) | 1.07 ( 0.4 ) | 0.1 ( -1.43 ) |
| Csf Protein Increased | 3 | 1.49 ( 0.44 - 5 ) | 1.49 ( 0.43 ) | 1.43 ( 0.52 ) | 0.52 ( -1.04 ) |
| Motor Developmental Delay | 3 | 2.09 ( 0.6 - 7.22 ) | 2.09 ( 1.42 ) | 1.91 ( 0.68 ) | 0.93 ( -0.66 ) |
| Herpes Simplex Test Negative | 3 | 0.67 ( 0.21 - 2.14 ) | 0.67 ( 0.47 ) | 0.69 ( 0.26 ) | -0.54 ( -2.04 ) |
| Magnetic Resonance Imaging Abnormal | 3 | 1.65 ( 0.49 - 5.57 ) | 1.65 ( 0.66 ) | 1.56 ( 0.56 ) | 0.64 ( -0.92 ) |
| Henoch-Schonlein Purpura | 3 | 0.82 ( 0.25 - 2.67 ) | 0.82 ( 0.1 ) | 0.84 ( 0.31 ) | -0.26 ( -1.77 ) |
| Off Label Use | 3 | 0.55 ( 0.17 - 1.76 ) | 0.55 ( 1.05 ) | 0.57 ( 0.22 ) | -0.81 ( -2.29 ) |
| Lividity | 3 | 0.58 ( 0.18 - 1.86 ) | 0.58 ( 0.86 ) | 0.6 ( 0.23 ) | -0.73 ( -2.22 ) |
| Oropharyngeal Pain | 3 | 0.6 ( 0.19 - 1.93 ) | 0.6 ( 0.74 ) | 0.62 ( 0.24 ) | -0.68 ( -2.17 ) |
| Cerebral Disorder | 3 | 3.48 ( 0.94 - 12.86 ) | 3.48 ( 3.98 ) | 2.86 ( 0.96 ) | 1.52 ( -0.13 ) |
| Secretion Discharge | 3 | 0.82 ( 0.25 - 2.67 ) | 0.82 ( 0.1 ) | 0.84 ( 0.31 ) | -0.26 ( -1.77 ) |
| Nodule | 3 | 0.9 ( 0.28 - 2.91 ) | 0.9 ( 0.03 ) | 0.9 ( 0.34 ) | -0.15 ( -1.66 ) |
| Skin Mass | 3 | 1.84 ( 0.54 - 6.29 ) | 1.84 ( 0.98 ) | 1.72 ( 0.61 ) | 0.78 ( -0.79 ) |
| Pharyngitis | 3 | 0.39 ( 0.12 - 1.22 ) | 0.39 ( 2.81 ) | 0.41 ( 0.16 ) | -1.29 ( -2.77 ) |
| Aggression | 3 | 0.48 ( 0.15 - 1.53 ) | 0.48 ( 1.6 ) | 0.5 ( 0.19 ) | -0.99 ( -2.47 ) |
| Cytomegalovirus Test Negative | 3 | 1.16 ( 0.35 - 3.83 ) | 1.16 ( 0.06 ) | 1.14 ( 0.42 ) | 0.19 ( -1.34 ) |
| Measles | 3 | 0.54 ( 0.17 - 1.72 ) | 0.54 ( 1.12 ) | 0.56 ( 0.21 ) | -0.83 ( -2.32 ) |
| Hyperventilation | 3 | 5.22 ( 1.31 - 20.89 ) | 5.22 ( 6.83 ) | 3.81 ( 1.2 ) | 1.93 ( 0.22 ) |
| Tonsillar Hypertrophy | 3 | 0.95 ( 0.29 - 3.1 ) | 0.95 ( 0.01 ) | 0.95 ( 0.35 ) | -0.07 ( -1.59 ) |
| Influenza A Virus Test Negative | 3 | 0.48 ( 0.15 - 1.53 ) | 0.48 ( 1.6 ) | 0.5 ( 0.19 ) | -0.99 ( -2.47 ) |
| Milk Allergy | 3 | 0.7 ( 0.22 - 2.24 ) | 0.7 ( 0.37 ) | 0.72 ( 0.27 ) | -0.48 ( -1.98 ) |
| Abscess | 3 | 2.41 ( 0.69 - 8.46 ) | 2.41 ( 2.01 ) | 2.15 ( 0.75 ) | 1.1 ( -0.5 ) |
| Enterovirus Test Positive | 3 | 0.87 ( 0.27 - 2.83 ) | 0.87 ( 0.05 ) | 0.88 ( 0.33 ) | -0.18 ( -1.7 ) |
| Red Blood Cell Sedimentation Rate Increased | 3 | 0.53 ( 0.17 - 1.69 ) | 0.53 ( 1.18 ) | 0.55 ( 0.21 ) | -0.85 ( -2.34 ) |
| Enterococcus Test Positive | 3 | 31.34 ( 3.26 - 301.32 ) | 31.33 ( 22.02 ) | 8.58 ( 1.29 ) | 3.1 ( 1.17 ) |
| Metabolic Acidosis | 3 | 1.65 ( 0.49 - 5.57 ) | 1.65 ( 0.66 ) | 1.56 ( 0.56 ) | 0.64 ( -0.92 ) |
| Staphylococcus Test Positive | 3 | 2.41 ( 0.69 - 8.46 ) | 2.41 ( 2.01 ) | 2.15 ( 0.75 ) | 1.1 ( -0.5 ) |
| Respiratory Syncytial Virus Infection | 3 | 0.95 ( 0.29 - 3.1 ) | 0.95 ( 0.01 ) | 0.95 ( 0.35 ) | -0.07 ( -1.59 ) |
| Blood Urea Decreased | 3 | 1.57 ( 0.47 - 5.27 ) | 1.57 ( 0.53 ) | 1.49 ( 0.54 ) | 0.58 ( -0.98 ) |
| Increased Appetite | 3 | 7.83 ( 1.75 - 35.01 ) | 7.83 ( 10.22 ) | 4.9 ( 1.4 ) | 2.29 ( 0.53 ) |
| Pupillary Light Reflex Tests Abnormal | 3 | 10.45 ( 2.11 - 51.76 ) | 10.44 ( 12.81 ) | 5.72 ( 1.5 ) | 2.52 ( 0.71 ) |
| Skin Exfoliation | 3 | 0.78 ( 0.24 - 2.53 ) | 0.78 ( 0.17 ) | 0.8 ( 0.3 ) | -0.32 ( -1.83 ) |
| Haematoma | 3 | 1.31 ( 0.39 - 4.34 ) | 1.31 ( 0.19 ) | 1.27 ( 0.47 ) | 0.35 ( -1.2 ) |
| Gastrointestinal Oedema | 3 | 6.27 ( 1.5 - 26.23 ) | 6.27 ( 8.3 ) | 4.29 ( 1.3 ) | 2.1 ( 0.37 ) |
| Fontanelle Depressed | 3 | 3.92 ( 1.04 - 14.77 ) | 3.92 ( 4.74 ) | 3.12 ( 1.03 ) | 1.64 ( -0.02 ) |
| Injection Site Irritation | 3 | 2.09 ( 0.6 - 7.22 ) | 2.09 ( 1.42 ) | 1.91 ( 0.68 ) | 0.93 ( -0.66 ) |
| Neurological Examination Abnormal | 3 | 2.85 ( 0.79 - 10.21 ) | 2.85 ( 2.83 ) | 2.45 ( 0.84 ) | 1.29 ( -0.33 ) |
| Lacrimation Increased | 3 | 0.75 ( 0.23 - 2.41 ) | 0.75 ( 0.24 ) | 0.76 ( 0.29 ) | -0.39 ( -1.89 ) |
| Arthritis | 3 | 2.24 ( 0.64 - 7.79 ) | 2.24 ( 1.69 ) | 2.02 ( 0.71 ) | 1.01 ( -0.58 ) |
| Gram Stain Negative | 3 | 3.48 ( 0.94 - 12.86 ) | 3.48 ( 3.98 ) | 2.86 ( 0.96 ) | 1.52 ( -0.13 ) |
| Covid-19 | 3 | 0.26 ( 0.08 - 0.81 ) | 0.26 ( 6.3 ) | 0.27 ( 0.11 ) | -1.86 ( -3.33 ) |
| Neutrophilia | 3 | 2.85 ( 0.79 - 10.21 ) | 2.85 ( 2.83 ) | 2.45 ( 0.84 ) | 1.29 ( -0.33 ) |
| Inflammatory Marker Increased | 3 | 0.85 ( 0.26 - 2.75 ) | 0.85 ( 0.08 ) | 0.86 ( 0.32 ) | -0.22 ( -1.73 ) |
| Gastroenteritis Rotavirus | 3 | 0.13 ( 0.04 - 0.4 ) | 0.13 ( 17.85 ) | 0.14 ( 0.05 ) | -2.86 ( -4.32 ) |
| Nasal Congestion | 3 | 0.3 ( 0.1 - 0.96 ) | 0.3 ( 4.64 ) | 0.32 ( 0.12 ) | -1.63 ( -3.1 ) |
| Enterovirus Test Negative | 3 | 1.25 ( 0.38 - 4.15 ) | 1.25 ( 0.14 ) | 1.23 ( 0.45 ) | 0.29 ( -1.25 ) |
| Arthralgia | 3 | 0.42 ( 0.13 - 1.32 ) | 0.42 ( 2.34 ) | 0.44 ( 0.17 ) | -1.18 ( -2.66 ) |
| Muscle Tightness | 3 | 0.92 ( 0.28 - 3 ) | 0.92 ( 0.02 ) | 0.93 ( 0.35 ) | -0.11 ( -1.62 ) |
| Extensive Swelling Of Vaccinated Limb | 3 | 0.67 ( 0.21 - 2.14 ) | 0.67 ( 0.47 ) | 0.69 ( 0.26 ) | -0.54 ( -2.04 ) |
| Conjunctivitis | 3 | 0.41 ( 0.13 - 1.31 ) | 0.41 ( 2.42 ) | 0.43 ( 0.17 ) | -1.2 ( -2.68 ) |
| Exposure Via Skin Contact | 3 | 1.31 ( 0.39 - 4.34 ) | 1.31 ( 0.19 ) | 1.27 ( 0.47 ) | 0.35 ( -1.2 ) |
| Skin Irritation | 3 | 1.57 ( 0.47 - 5.27 ) | 1.57 ( 0.53 ) | 1.49 ( 0.54 ) | 0.58 ( -0.98 ) |
| Staphylococcal Infection | 3 | 2.09 ( 0.6 - 7.22 ) | 2.09 ( 1.42 ) | 1.91 ( 0.68 ) | 0.93 ( -0.66 ) |
| Moaning | 3 | 0.68 ( 0.21 - 2.19 ) | 0.68 ( 0.42 ) | 0.7 ( 0.26 ) | -0.51 ( -2.01 ) |
| Muscle Contractions Involuntary | 3 | 4.48 ( 1.16 - 17.32 ) | 4.48 ( 5.67 ) | 3.43 ( 1.11 ) | 1.78 ( 0.09 ) |
| Respiratory Distress | 3 | 0.51 ( 0.16 - 1.61 ) | 0.51 ( 1.39 ) | 0.53 ( 0.2 ) | -0.92 ( -2.41 ) |
| Facial Paralysis | 3 | 0.78 ( 0.24 - 2.53 ) | 0.78 ( 0.17 ) | 0.8 ( 0.3 ) | -0.32 ( -1.83 ) |
| Protrusion Tongue | 3 | 2.85 ( 0.79 - 10.21 ) | 2.85 ( 2.83 ) | 2.45 ( 0.84 ) | 1.29 ( -0.33 ) |
| Skin Wound | 3 | 6.27 ( 1.5 - 26.23 ) | 6.27 ( 8.3 ) | 4.29 ( 1.3 ) | 2.1 ( 0.37 ) |
| Encephalitis | 3 | 0.41 ( 0.13 - 1.31 ) | 0.41 ( 2.42 ) | 0.43 ( 0.17 ) | -1.2 ( -2.68 ) |
| Varicella Post Vaccine | 3 | 0.32 ( 0.1 - 1.01 ) | 0.32 ( 4.22 ) | 0.34 ( 0.13 ) | -1.56 ( -3.03 ) |
| Cold Sweat | 3 | 0.87 ( 0.27 - 2.83 ) | 0.87 ( 0.05 ) | 0.88 ( 0.33 ) | -0.18 ( -1.7 ) |
| Bowel Movement Irregularity | 3 | 3.92 ( 1.04 - 14.77 ) | 3.92 ( 4.74 ) | 3.12 ( 1.03 ) | 1.64 ( -0.02 ) |
| Failure To Thrive | 3 | 1.57 ( 0.47 - 5.27 ) | 1.57 ( 0.53 ) | 1.49 ( 0.54 ) | 0.58 ( -0.98 ) |
| Selective Eating Disorder | 3 | 3.13 ( 0.86 - 11.39 ) | 3.13 ( 3.35 ) | 2.64 ( 0.9 ) | 1.4 ( -0.24 ) |
| Myoclonus | 3 | 1.01 ( 0.31 - 3.31 ) | 1.01 ( 0 ) | 1.01 ( 0.37 ) | 0.01 ( -1.51 ) |
| Injection Site Scar | 3 | 6.27 ( 1.5 - 26.23 ) | 6.27 ( 8.3 ) | 4.29 ( 1.3 ) | 2.1 ( 0.37 ) |
| Abdominal Discomfort | 3 | 0.73 ( 0.23 - 2.35 ) | 0.73 ( 0.28 ) | 0.75 ( 0.28 ) | -0.42 ( -1.93 ) |
| Viral Rash | 3 | 0.67 ( 0.21 - 2.14 ) | 0.67 ( 0.47 ) | 0.69 ( 0.26 ) | -0.54 ( -2.04 ) |
| Oedema | 3 | 0.61 ( 0.19 - 1.97 ) | 0.61 ( 0.69 ) | 0.64 ( 0.24 ) | -0.65 ( -2.15 ) |
| Dermatitis Contact | 3 | 3.48 ( 0.94 - 12.86 ) | 3.48 ( 3.98 ) | 2.86 ( 0.96 ) | 1.52 ( -0.13 ) |
| Immune Thrombocytopenic Purpura | 3 | 0.61 ( 0.19 - 1.97 ) | 0.61 ( 0.69 ) | 0.64 ( 0.24 ) | -0.65 ( -2.15 ) |
| Urinary Tract Infection | 3 | 0.39 ( 0.12 - 1.22 ) | 0.39 ( 2.81 ) | 0.41 ( 0.16 ) | -1.29 ( -2.77 ) |
| Blood Urea Increased | 3 | 1.74 ( 0.51 - 5.91 ) | 1.74 ( 0.81 ) | 1.63 ( 0.59 ) | 0.71 ( -0.86 ) |
| Coordination Abnormal | 2 | 0.75 ( 0.18 - 3.13 ) | 0.75 ( 0.16 ) | 0.76 ( 0.23 ) | -0.39 ( -2.14 ) |
| Rhinovirus Infection | 2 | 0.84 ( 0.2 - 3.53 ) | 0.84 ( 0.06 ) | 0.85 ( 0.25 ) | -0.24 ( -1.99 ) |
| Enterovirus Infection | 2 | 1.1 ( 0.26 - 4.72 ) | 1.1 ( 0.02 ) | 1.09 ( 0.32 ) | 0.12 ( -1.65 ) |
| Head Titubation | 2 | 1.23 ( 0.28 - 5.32 ) | 1.23 ( 0.08 ) | 1.2 ( 0.35 ) | 0.27 ( -1.52 ) |
| Reduced Facial Expression | 2 | 2.32 ( 0.5 - 10.74 ) | 2.32 ( 1.23 ) | 2.08 ( 0.58 ) | 1.06 ( -0.81 ) |
| Haematuria | 2 | 2.61 ( 0.55 - 12.3 ) | 2.61 ( 1.59 ) | 2.29 ( 0.63 ) | 1.19 ( -0.68 ) |
| Emotional Disorder | 2 | 1.31 ( 0.3 - 5.68 ) | 1.31 ( 0.13 ) | 1.27 ( 0.37 ) | 0.35 ( -1.45 ) |
| Demyelination | 2 | 3.48 ( 0.7 - 17.25 ) | 3.48 ( 2.65 ) | 2.86 ( 0.75 ) | 1.52 ( -0.41 ) |
| Ataxia | 2 | 0.58 ( 0.14 - 2.41 ) | 0.58 ( 0.58 ) | 0.6 ( 0.18 ) | -0.73 ( -2.46 ) |
| Metabolic Disorder | 2 | 2.09 ( 0.46 - 9.54 ) | 2.09 ( 0.95 ) | 1.91 ( 0.54 ) | 0.93 ( -0.92 ) |
| Skin Reaction | 2 | 0.72 ( 0.17 - 3.02 ) | 0.72 ( 0.2 ) | 0.74 ( 0.22 ) | -0.44 ( -2.18 ) |
| Urinary Incontinence | 2 | 2.32 ( 0.5 - 10.74 ) | 2.32 ( 1.23 ) | 2.08 ( 0.58 ) | 1.06 ( -0.81 ) |
| Faeces Soft | 2 | 0.99 ( 0.23 - 4.24 ) | 0.99 ( 0 ) | 1 ( 0.3 ) | -0.01 ( -1.77 ) |
| Asphyxia | 2 | 0.8 ( 0.19 - 3.39 ) | 0.8 ( 0.09 ) | 0.82 ( 0.25 ) | -0.29 ( -2.04 ) |
| Paralysis | 2 | 0.67 ( 0.16 - 2.82 ) | 0.67 ( 0.3 ) | 0.69 ( 0.21 ) | -0.53 ( -2.27 ) |
| Acidosis | 2 | 1.9 ( 0.42 - 8.57 ) | 1.9 ( 0.72 ) | 1.76 ( 0.5 ) | 0.82 ( -1.02 ) |
| Eructation | 2 | 4.18 ( 0.81 - 21.54 ) | 4.18 ( 3.45 ) | 3.27 ( 0.83 ) | 1.71 ( -0.24 ) |
| Salmonella Test Negative | 2 | 1.31 ( 0.3 - 5.68 ) | 1.31 ( 0.13 ) | 1.27 ( 0.37 ) | 0.35 ( -1.45 ) |
| Parasite Stool Test Negative | 2 | 1.49 ( 0.34 - 6.57 ) | 1.49 ( 0.28 ) | 1.43 ( 0.41 ) | 0.52 ( -1.29 ) |
| Red Cell Distribution Width | 2 | 1.61 ( 0.36 - 7.12 ) | 1.61 ( 0.4 ) | 1.53 ( 0.44 ) | 0.61 ( -1.21 ) |
| Basophil Count Decreased | 2 | 0.65 ( 0.16 - 2.72 ) | 0.65 ( 0.35 ) | 0.67 ( 0.2 ) | -0.57 ( -2.31 ) |
| Hypopnoea | 2 | 0.55 ( 0.13 - 2.28 ) | 0.55 ( 0.7 ) | 0.57 ( 0.17 ) | -0.81 ( -2.53 ) |
| Lumbar Puncture Abnormal | 2 | 1.23 ( 0.28 - 5.32 ) | 1.23 ( 0.08 ) | 1.2 ( 0.35 ) | 0.27 ( -1.52 ) |
| Apnoeic Attack | 2 | 2.32 ( 0.5 - 10.74 ) | 2.32 ( 1.23 ) | 2.08 ( 0.58 ) | 1.06 ( -0.81 ) |
| Csf Red Blood Cell Count Positive | 2 | 0.99 ( 0.23 - 4.24 ) | 0.99 ( 0 ) | 1 ( 0.3 ) | -0.01 ( -1.77 ) |
| Rash Morbilliform | 2 | 0.1 ( 0.03 - 0.41 ) | 0.1 ( 15.7 ) | 0.11 ( 0.03 ) | -3.18 ( -4.86 ) |
| Regurgitation | 2 | 0.54 ( 0.13 - 2.22 ) | 0.54 ( 0.77 ) | 0.56 ( 0.17 ) | -0.84 ( -2.57 ) |
| Eyelid Oedema | 2 | 0.8 ( 0.19 - 3.39 ) | 0.8 ( 0.09 ) | 0.82 ( 0.25 ) | -0.29 ( -2.04 ) |
| Cerebral Palsy | 2 | 6.96 ( 1.16 - 41.68 ) | 6.96 ( 6.13 ) | 4.58 ( 1.02 ) | 2.19 ( 0.15 ) |
| Hypoglycaemia | 2 | 1.31 ( 0.3 - 5.68 ) | 1.31 ( 0.13 ) | 1.27 ( 0.37 ) | 0.35 ( -1.45 ) |
| Rash Rubelliform | 2 | 0.5 ( 0.12 - 2.05 ) | 0.5 ( 0.97 ) | 0.52 ( 0.16 ) | -0.94 ( -2.66 ) |
| Infantile Back Arching | 2 | 0.65 ( 0.16 - 2.72 ) | 0.65 ( 0.35 ) | 0.67 ( 0.2 ) | -0.57 ( -2.31 ) |
| Papule | 2 | 0.28 ( 0.07 - 1.13 ) | 0.28 ( 3.64 ) | 0.3 ( 0.09 ) | -1.75 ( -3.45 ) |
| Tonsillar Erythema | 2 | 20.89 ( 1.89 - 230.42 ) | 20.89 ( 12.62 ) | 7.63 ( 1.02 ) | 2.93 ( 0.73 ) |
| Vaccination Site Granuloma | 2 | 2.32 ( 0.5 - 10.74 ) | 2.32 ( 1.23 ) | 2.08 ( 0.58 ) | 1.06 ( -0.81 ) |
| Acute Kidney Injury | 2 | 2.09 ( 0.46 - 9.54 ) | 2.09 ( 0.95 ) | 1.91 ( 0.54 ) | 0.93 ( -0.92 ) |
| Splenomegaly | 2 | 2.32 ( 0.5 - 10.74 ) | 2.32 ( 1.23 ) | 2.08 ( 0.58 ) | 1.06 ( -0.81 ) |
| Streptococcus Test Positive | 2 | 0.43 ( 0.1 - 1.75 ) | 0.43 ( 1.48 ) | 0.45 ( 0.14 ) | -1.16 ( -2.87 ) |
| Hyperacusis | 2 | 1.39 ( 0.32 - 6.09 ) | 1.39 ( 0.2 ) | 1.35 ( 0.39 ) | 0.43 ( -1.37 ) |
| Lip Discolouration | 2 | 0.91 ( 0.21 - 3.85 ) | 0.91 ( 0.02 ) | 0.92 ( 0.27 ) | -0.13 ( -1.89 ) |
| Adenovirus Test Positive | 2 | 0.7 ( 0.17 - 2.91 ) | 0.7 ( 0.25 ) | 0.72 ( 0.22 ) | -0.48 ( -2.22 ) |
| Growth Retardation | 2 | 2.32 ( 0.5 - 10.74 ) | 2.32 ( 1.23 ) | 2.08 ( 0.58 ) | 1.06 ( -0.81 ) |
| Blepharospasm | 2 | 1.61 ( 0.36 - 7.12 ) | 1.61 ( 0.4 ) | 1.53 ( 0.44 ) | 0.61 ( -1.21 ) |
| Blood Gases Abnormal | 2 | 3.48 ( 0.7 - 17.25 ) | 3.48 ( 2.65 ) | 2.86 ( 0.75 ) | 1.52 ( -0.41 ) |
| Musculoskeletal Disorder | 2 | 1.39 ( 0.32 - 6.09 ) | 1.39 ( 0.2 ) | 1.35 ( 0.39 ) | 0.43 ( -1.37 ) |
| Dysuria | 2 | 2.98 ( 0.62 - 14.37 ) | 2.98 ( 2.05 ) | 2.54 ( 0.68 ) | 1.35 ( -0.55 ) |
| Blood Bicarbonate Decreased | 2 | 0.95 ( 0.22 - 4.04 ) | 0.95 ( 0 ) | 0.95 ( 0.28 ) | -0.07 ( -1.83 ) |
| Escherichia Test Positive | 2 | 0.99 ( 0.23 - 4.24 ) | 0.99 ( 0 ) | 1 ( 0.3 ) | -0.01 ( -1.77 ) |
| Gastrointestinal Necrosis | 2 | 2.09 ( 0.46 - 9.54 ) | 2.09 ( 0.95 ) | 1.91 ( 0.54 ) | 0.93 ( -0.92 ) |
| Large Intestine Perforation | 2 | 10.45 ( 1.47 - 74.16 ) | 10.44 ( 8.54 ) | 5.72 ( 1.11 ) | 2.52 ( 0.41 ) |
| Respiratory Syncytial Virus Test Positive | 2 | 0.7 ( 0.17 - 2.91 ) | 0.7 ( 0.25 ) | 0.72 ( 0.22 ) | -0.48 ( -2.22 ) |
| Bronchial Wall Thickening | 2 | 10.45 ( 1.47 - 74.16 ) | 10.44 ( 8.54 ) | 5.72 ( 1.11 ) | 2.52 ( 0.41 ) |
| Eosinophil Percentage Increased | 2 | 1.04 ( 0.24 - 4.47 ) | 1.04 ( 0 ) | 1.04 ( 0.31 ) | 0.06 ( -1.71 ) |
| Fibrin D Dimer Increased | 2 | 4.18 ( 0.81 - 21.54 ) | 4.18 ( 3.45 ) | 3.27 ( 0.83 ) | 1.71 ( -0.24 ) |
| Blood Fibrinogen Decreased | 2 | 2.32 ( 0.5 - 10.74 ) | 2.32 ( 1.23 ) | 2.08 ( 0.58 ) | 1.06 ( -0.81 ) |
| Staphylococcal Scalded Skin Syndrome | 2 | 6.96 ( 1.16 - 41.68 ) | 6.96 ( 6.13 ) | 4.58 ( 1.02 ) | 2.19 ( 0.15 ) |
| Hyperaemia | 2 | 0.99 ( 0.23 - 4.24 ) | 0.99 ( 0 ) | 1 ( 0.3 ) | -0.01 ( -1.77 ) |
| Irritability Postvaccinal | 2 | 0.75 ( 0.18 - 3.13 ) | 0.75 ( 0.16 ) | 0.76 ( 0.23 ) | -0.39 ( -2.14 ) |
| Bicytopenia | 2 | 20.89 ( 1.89 - 230.42 ) | 20.89 ( 12.62 ) | 7.63 ( 1.02 ) | 2.93 ( 0.73 ) |
| Head Injury | 2 | 1.04 ( 0.24 - 4.47 ) | 1.04 ( 0 ) | 1.04 ( 0.31 ) | 0.06 ( -1.71 ) |
| Hepatic Enzyme Increased | 2 | 0.95 ( 0.22 - 4.04 ) | 0.95 ( 0 ) | 0.95 ( 0.28 ) | -0.07 ( -1.83 ) |
| Red Blood Cells Urine Positive | 2 | 1.39 ( 0.32 - 6.09 ) | 1.39 ( 0.2 ) | 1.35 ( 0.39 ) | 0.43 ( -1.37 ) |
| Ear Disorder | 2 | 5.22 ( 0.96 - 28.52 ) | 5.22 ( 4.55 ) | 3.81 ( 0.92 ) | 1.93 ( -0.06 ) |
| Ecchymosis | 2 | 0.49 ( 0.12 - 2.01 ) | 0.49 ( 1.04 ) | 0.51 ( 0.16 ) | -0.98 ( -2.69 ) |
| Blood Calcium Increased | 2 | 1.74 ( 0.39 - 7.78 ) | 1.74 ( 0.54 ) | 1.63 ( 0.47 ) | 0.71 ( -1.12 ) |
| Lymphocyte Percentage Decreased | 2 | 0.84 ( 0.2 - 3.53 ) | 0.84 ( 0.06 ) | 0.85 ( 0.25 ) | -0.24 ( -1.99 ) |
| Eyelid Ptosis | 2 | 1.31 ( 0.3 - 5.68 ) | 1.31 ( 0.13 ) | 1.27 ( 0.37 ) | 0.35 ( -1.45 ) |
| Poor Sucking Reflex | 2 | 0.91 ( 0.21 - 3.85 ) | 0.91 ( 0.02 ) | 0.92 ( 0.27 ) | -0.13 ( -1.89 ) |
| Asthma | 2 | 0.43 ( 0.1 - 1.75 ) | 0.43 ( 1.48 ) | 0.45 ( 0.14 ) | -1.16 ( -2.87 ) |
| Neurological Symptom | 2 | 1.04 ( 0.24 - 4.47 ) | 1.04 ( 0 ) | 1.04 ( 0.31 ) | 0.06 ( -1.71 ) |
| Dysstasia | 2 | 0.3 ( 0.07 - 1.22 ) | 0.3 ( 3.21 ) | 0.32 ( 0.1 ) | -1.65 ( -3.35 ) |
| Arthritis Bacterial | 2 | 3.48 ( 0.7 - 17.25 ) | 3.48 ( 2.65 ) | 2.86 ( 0.75 ) | 1.52 ( -0.41 ) |
| Productive Cough | 2 | 0.39 ( 0.09 - 1.59 ) | 0.39 ( 1.88 ) | 0.41 ( 0.13 ) | -1.29 ( -3 ) |
| Liver Function Test Increased | 2 | 2.98 ( 0.62 - 14.37 ) | 2.98 ( 2.05 ) | 2.54 ( 0.68 ) | 1.35 ( -0.55 ) |
| Blood Iron Decreased | 2 | 2.98 ( 0.62 - 14.37 ) | 2.98 ( 2.05 ) | 2.54 ( 0.68 ) | 1.35 ( -0.55 ) |
| Culture Positive | 2 | 2.98 ( 0.62 - 14.37 ) | 2.98 ( 2.05 ) | 2.54 ( 0.68 ) | 1.35 ( -0.55 ) |
| Blood Urine Absent | 2 | 2.32 ( 0.5 - 10.74 ) | 2.32 ( 1.23 ) | 2.08 ( 0.58 ) | 1.06 ( -0.81 ) |
| Rotavirus Infection | 2 | 0.15 ( 0.04 - 0.61 ) | 0.15 ( 9.48 ) | 0.16 ( 0.05 ) | -2.62 ( -4.31 ) |
| Bronchiolitis | 2 | 0.43 ( 0.1 - 1.75 ) | 0.43 ( 1.48 ) | 0.45 ( 0.14 ) | -1.16 ( -2.87 ) |
| Sars-Cov-2 Test Positive | 2 | 0.23 ( 0.06 - 0.95 ) | 0.23 ( 4.88 ) | 0.25 ( 0.08 ) | -1.99 ( -3.68 ) |
| Electrocardiogram Abnormal | 2 | 0.72 ( 0.17 - 3.02 ) | 0.72 ( 0.2 ) | 0.74 ( 0.22 ) | -0.44 ( -2.18 ) |
| Personality Change | 2 | 0.58 ( 0.14 - 2.41 ) | 0.58 ( 0.58 ) | 0.6 ( 0.18 ) | -0.73 ( -2.46 ) |
| Rectal Tenesmus | 2 | 20.89 ( 1.89 - 230.42 ) | 20.89 ( 12.62 ) | 7.63 ( 1.02 ) | 2.93 ( 0.73 ) |
| Alopecia | 2 | 1.04 ( 0.24 - 4.47 ) | 1.04 ( 0 ) | 1.04 ( 0.31 ) | 0.06 ( -1.71 ) |
| Alopecia Areata | 2 | 6.96 ( 1.16 - 41.68 ) | 6.96 ( 6.13 ) | 4.58 ( 1.02 ) | 2.19 ( 0.15 ) |
| Infrequent Bowel Movements | 2 | 3.48 ( 0.7 - 17.25 ) | 3.48 ( 2.65 ) | 2.86 ( 0.75 ) | 1.52 ( -0.41 ) |
| Hypertransaminasaemia | 2 | 4.18 ( 0.81 - 21.54 ) | 4.18 ( 3.45 ) | 3.27 ( 0.83 ) | 1.71 ( -0.24 ) |
| Antinuclear Antibody Negative | 2 | 3.48 ( 0.7 - 17.25 ) | 3.48 ( 2.65 ) | 2.86 ( 0.75 ) | 1.52 ( -0.41 ) |
| Prothrombin Time Prolonged | 2 | 1.9 ( 0.42 - 8.57 ) | 1.9 ( 0.72 ) | 1.76 ( 0.5 ) | 0.82 ( -1.02 ) |
| Red Blood Cell Count Decreased | 2 | 0.63 ( 0.15 - 2.64 ) | 0.63 ( 0.4 ) | 0.65 ( 0.2 ) | -0.61 ( -2.35 ) |
| Infantile Diarrhoea | 2 | 1.16 ( 0.27 - 5 ) | 1.16 ( 0.04 ) | 1.14 ( 0.34 ) | 0.19 ( -1.59 ) |
| Bilirubin Urine | 2 | 1.1 ( 0.26 - 4.72 ) | 1.1 ( 0.02 ) | 1.09 ( 0.32 ) | 0.12 ( -1.65 ) |
| Blood Urine Present | 2 | 2.09 ( 0.46 - 9.54 ) | 2.09 ( 0.95 ) | 1.91 ( 0.54 ) | 0.93 ( -0.92 ) |
| White Blood Cells Urine Negative | 2 | 0.87 ( 0.21 - 3.68 ) | 0.87 ( 0.04 ) | 0.88 ( 0.26 ) | -0.18 ( -1.94 ) |
| Altered State Of Consciousness | 2 | 0.25 ( 0.06 - 1.04 ) | 0.25 ( 4.26 ) | 0.27 ( 0.08 ) | -1.88 ( -3.57 ) |
| Hepatitis B Surface Antibody Positive | 2 | 1.9 ( 0.42 - 8.57 ) | 1.9 ( 0.72 ) | 1.76 ( 0.5 ) | 0.82 ( -1.02 ) |
| Hypoaesthesia | 2 | 1.49 ( 0.34 - 6.57 ) | 1.49 ( 0.28 ) | 1.43 ( 0.41 ) | 0.52 ( -1.29 ) |
| Floppy Infant | 2 | 0.7 ( 0.17 - 2.91 ) | 0.7 ( 0.25 ) | 0.72 ( 0.22 ) | -0.48 ( -2.22 ) |
| Cytogenetic Analysis Abnormal | 2 | 2.09 ( 0.46 - 9.54 ) | 2.09 ( 0.95 ) | 1.91 ( 0.54 ) | 0.93 ( -0.92 ) |
| Haemolysis | 2 | 3.48 ( 0.7 - 17.25 ) | 3.48 ( 2.65 ) | 2.86 ( 0.75 ) | 1.52 ( -0.41 ) |
| Hyperreflexia | 2 | 3.48 ( 0.7 - 17.25 ) | 3.48 ( 2.65 ) | 2.86 ( 0.75 ) | 1.52 ( -0.41 ) |
| Swollen Tongue | 2 | 0.99 ( 0.23 - 4.24 ) | 0.99 ( 0 ) | 1 ( 0.3 ) | -0.01 ( -1.77 ) |
| Mood Altered | 2 | 0.18 ( 0.04 - 0.71 ) | 0.18 ( 7.62 ) | 0.19 ( 0.06 ) | -2.4 ( -4.09 ) |
| Bacterial Infection | 2 | 0.56 ( 0.14 - 2.34 ) | 0.56 ( 0.64 ) | 0.59 ( 0.18 ) | -0.77 ( -2.5 ) |
| Mouth Swelling | 2 | 4.18 ( 0.81 - 21.54 ) | 4.18 ( 3.45 ) | 3.27 ( 0.83 ) | 1.71 ( -0.24 ) |
| Tubulointerstitial Nephritis | 2 | 10.45 ( 1.47 - 74.16 ) | 10.44 ( 8.54 ) | 5.72 ( 1.11 ) | 2.52 ( 0.41 ) |
| Chromaturia | 2 | 0.95 ( 0.22 - 4.04 ) | 0.95 ( 0 ) | 0.95 ( 0.28 ) | -0.07 ( -1.83 ) |
| Eosinophil Count Decreased | 2 | 0.7 ( 0.17 - 2.91 ) | 0.7 ( 0.25 ) | 0.72 ( 0.22 ) | -0.48 ( -2.22 ) |
| Limb Mass | 2 | 1.49 ( 0.34 - 6.57 ) | 1.49 ( 0.28 ) | 1.43 ( 0.41 ) | 0.52 ( -1.29 ) |
| Macule | 2 | 0.75 ( 0.18 - 3.13 ) | 0.75 ( 0.16 ) | 0.76 ( 0.23 ) | -0.39 ( -2.14 ) |
| Dermatitis Bullous | 2 | 0.91 ( 0.21 - 3.85 ) | 0.91 ( 0.02 ) | 0.92 ( 0.27 ) | -0.13 ( -1.89 ) |
| Eosinophil Count Increased | 2 | 1.04 ( 0.24 - 4.47 ) | 1.04 ( 0 ) | 1.04 ( 0.31 ) | 0.06 ( -1.71 ) |
| Pemphigoid | 2 | 2.98 ( 0.62 - 14.37 ) | 2.98 ( 2.05 ) | 2.54 ( 0.68 ) | 1.35 ( -0.55 ) |
| Ear Swelling | 2 | 1.39 ( 0.32 - 6.09 ) | 1.39 ( 0.2 ) | 1.35 ( 0.39 ) | 0.43 ( -1.37 ) |
| Cardio-Respiratory Arrest | 2 | 0.44 ( 0.11 - 1.79 ) | 0.44 ( 1.41 ) | 0.46 ( 0.14 ) | -1.13 ( -2.84 ) |
| Skin Haemorrhage | 2 | 1.74 ( 0.39 - 7.78 ) | 1.74 ( 0.54 ) | 1.63 ( 0.47 ) | 0.71 ( -1.12 ) |
| Mass | 2 | 0.75 ( 0.18 - 3.13 ) | 0.75 ( 0.16 ) | 0.76 ( 0.23 ) | -0.39 ( -2.14 ) |
| Nuclear Magnetic Resonance Imaging Abnormal | 2 | 2.61 ( 0.55 - 12.3 ) | 2.61 ( 1.59 ) | 2.29 ( 0.63 ) | 1.19 ( -0.68 ) |
| Vaccination Site Rash | 2 | 0.52 ( 0.13 - 2.16 ) | 0.52 ( 0.83 ) | 0.54 ( 0.17 ) | -0.88 ( -2.6 ) |
| Face Oedema | 2 | 1.49 ( 0.34 - 6.57 ) | 1.49 ( 0.28 ) | 1.43 ( 0.41 ) | 0.52 ( -1.29 ) |
| Disturbance In Social Behaviour | 2 | 1.39 ( 0.32 - 6.09 ) | 1.39 ( 0.2 ) | 1.35 ( 0.39 ) | 0.43 ( -1.37 ) |
| Stereotypy | 2 | 1.23 ( 0.28 - 5.32 ) | 1.23 ( 0.08 ) | 1.2 ( 0.35 ) | 0.27 ( -1.52 ) |
| Myelitis Transverse | 2 | 1.9 ( 0.42 - 8.57 ) | 1.9 ( 0.72 ) | 1.76 ( 0.5 ) | 0.82 ( -1.02 ) |
| Brain Injury | 2 | 0.77 ( 0.18 - 3.25 ) | 0.77 ( 0.12 ) | 0.79 ( 0.24 ) | -0.34 ( -2.09 ) |
| Tonsillitis | 2 | 0.39 ( 0.09 - 1.59 ) | 0.39 ( 1.88 ) | 0.41 ( 0.13 ) | -1.29 ( -3 ) |
| Language Disorder | 2 | 0.8 ( 0.19 - 3.39 ) | 0.8 ( 0.09 ) | 0.82 ( 0.25 ) | -0.29 ( -2.04 ) |
| Grimacing | 2 | 2.61 ( 0.55 - 12.3 ) | 2.61 ( 1.59 ) | 2.29 ( 0.63 ) | 1.19 ( -0.68 ) |
| Enuresis | 2 | 6.96 ( 1.16 - 41.68 ) | 6.96 ( 6.13 ) | 4.58 ( 1.02 ) | 2.19 ( 0.15 ) |
| Varicella Virus Test Positive | 2 | 0.27 ( 0.07 - 1.1 ) | 0.27 ( 3.82 ) | 0.29 ( 0.09 ) | -1.79 ( -3.48 ) |
| Skin Hypertrophy | 2 | 6.96 ( 1.16 - 41.68 ) | 6.96 ( 6.13 ) | 4.58 ( 1.02 ) | 2.19 ( 0.15 ) |
| Wound | 2 | 1.49 ( 0.34 - 6.57 ) | 1.49 ( 0.28 ) | 1.43 ( 0.41 ) | 0.52 ( -1.29 ) |
| Cafe Au Lait Spots | 2 | 20.89 ( 1.89 - 230.42 ) | 20.89 ( 12.62 ) | 7.63 ( 1.02 ) | 2.93 ( 0.73 ) |
| Vasodilatation | 2 | 20.89 ( 1.89 - 230.42 ) | 20.89 ( 12.62 ) | 7.63 ( 1.02 ) | 2.93 ( 0.73 ) |
| Intentional Self-Injury | 2 | 1.16 ( 0.27 - 5 ) | 1.16 ( 0.04 ) | 1.14 ( 0.34 ) | 0.19 ( -1.59 ) |
| Morbillivirus Test Positive | 2 | 0.63 ( 0.15 - 2.64 ) | 0.63 ( 0.4 ) | 0.65 ( 0.2 ) | -0.61 ( -2.35 ) |
| Visual Impairment | 2 | 1.31 ( 0.3 - 5.68 ) | 1.31 ( 0.13 ) | 1.27 ( 0.37 ) | 0.35 ( -1.45 ) |
| Haematemesis | 2 | 0.99 ( 0.23 - 4.24 ) | 0.99 ( 0 ) | 1 ( 0.3 ) | -0.01 ( -1.77 ) |
| Injection Site Streaking | 2 | 10.45 ( 1.47 - 74.16 ) | 10.44 ( 8.54 ) | 5.72 ( 1.11 ) | 2.52 ( 0.41 ) |
| Oral Mucosal Eruption | 2 | 0.99 ( 0.23 - 4.24 ) | 0.99 ( 0 ) | 1 ( 0.3 ) | -0.01 ( -1.77 ) |
| Dysphonia | 2 | 0.75 ( 0.18 - 3.13 ) | 0.75 ( 0.16 ) | 0.76 ( 0.23 ) | -0.39 ( -2.14 ) |
| Weight Gain Poor | 2 | 0.87 ( 0.21 - 3.68 ) | 0.87 ( 0.04 ) | 0.88 ( 0.26 ) | -0.18 ( -1.94 ) |
| Ear Discomfort | 2 | 2.61 ( 0.55 - 12.3 ) | 2.61 ( 1.59 ) | 2.29 ( 0.63 ) | 1.19 ( -0.68 ) |
| Injection Site Eczema | 2 | 10.45 ( 1.47 - 74.16 ) | 10.44 ( 8.54 ) | 5.72 ( 1.11 ) | 2.52 ( 0.41 ) |
| Vaccination Site Haemorrhage | 2 | 4.18 ( 0.81 - 21.54 ) | 4.18 ( 3.45 ) | 3.27 ( 0.83 ) | 1.71 ( -0.24 ) |
| Exposure To Communicable Disease | 2 | 2.61 ( 0.55 - 12.3 ) | 2.61 ( 1.59 ) | 2.29 ( 0.63 ) | 1.19 ( -0.68 ) |
| Penile Erythema | 2 | 20.89 ( 1.89 - 230.42 ) | 20.89 ( 12.62 ) | 7.63 ( 1.02 ) | 2.93 ( 0.73 ) |
| Vasculitis | 2 | 2.09 ( 0.46 - 9.54 ) | 2.09 ( 0.95 ) | 1.91 ( 0.54 ) | 0.93 ( -0.92 ) |
| Injection Site Discharge | 2 | 1.04 ( 0.24 - 4.47 ) | 1.04 ( 0 ) | 1.04 ( 0.31 ) | 0.06 ( -1.71 ) |
| Initial Insomnia | 2 | 1.9 ( 0.42 - 8.57 ) | 1.9 ( 0.72 ) | 1.76 ( 0.5 ) | 0.82 ( -1.02 ) |
| Monocyte Count Increased | 2 | 0.34 ( 0.08 - 1.4 ) | 0.34 ( 2.45 ) | 0.36 ( 0.11 ) | -1.46 ( -3.17 ) |
| Asterixis | 2 | 2.32 ( 0.5 - 10.74 ) | 2.32 ( 1.23 ) | 2.08 ( 0.58 ) | 1.06 ( -0.81 ) |
| Joint Contracture | 2 | 4.18 ( 0.81 - 21.54 ) | 4.18 ( 3.45 ) | 3.27 ( 0.83 ) | 1.71 ( -0.24 ) |
| Blood Lactic Acid Increased | 2 | 0.84 ( 0.2 - 3.53 ) | 0.84 ( 0.06 ) | 0.85 ( 0.25 ) | -0.24 ( -1.99 ) |
| Livedo Reticularis | 2 | 0.43 ( 0.1 - 1.75 ) | 0.43 ( 1.48 ) | 0.45 ( 0.14 ) | -1.16 ( -2.87 ) |
| Blood Alkaline Phosphatase Increased | 2 | 0.47 ( 0.12 - 1.96 ) | 0.47 ( 1.11 ) | 0.5 ( 0.15 ) | -1.01 ( -2.73 ) |
| Feeling Hot | 2 | 0.27 ( 0.07 - 1.1 ) | 0.27 ( 3.82 ) | 0.29 ( 0.09 ) | -1.79 ( -3.48 ) |
| Muscle Strain | 2 | 3.48 ( 0.7 - 17.25 ) | 3.48 ( 2.65 ) | 2.86 ( 0.75 ) | 1.52 ( -0.41 ) |
| Paresis | 2 | 4.18 ( 0.81 - 21.54 ) | 4.18 ( 3.45 ) | 3.27 ( 0.83 ) | 1.71 ( -0.24 ) |
| Erythema Of Eyelid | 2 | 1.16 ( 0.27 - 5 ) | 1.16 ( 0.04 ) | 1.14 ( 0.34 ) | 0.19 ( -1.59 ) |
| Meningitis Pneumococcal | 2 | 1.61 ( 0.36 - 7.12 ) | 1.61 ( 0.4 ) | 1.53 ( 0.44 ) | 0.61 ( -1.21 ) |
| Magnetic Resonance Imaging Brain Abnormal | 2 | 0.99 ( 0.23 - 4.24 ) | 0.99 ( 0 ) | 1 ( 0.3 ) | -0.01 ( -1.77 ) |
| Injection Site Macule | 2 | 6.96 ( 1.16 - 41.68 ) | 6.96 ( 6.13 ) | 4.58 ( 1.02 ) | 2.19 ( 0.15 ) |
| Inability To Crawl | 2 | 0.91 ( 0.21 - 3.85 ) | 0.91 ( 0.02 ) | 0.92 ( 0.27 ) | -0.13 ( -1.89 ) |
| Hypothermia | 2 | 0.52 ( 0.13 - 2.16 ) | 0.52 ( 0.83 ) | 0.54 ( 0.17 ) | -0.88 ( -2.6 ) |
| Supraventricular Tachycardia | 2 | 2.09 ( 0.46 - 9.54 ) | 2.09 ( 0.95 ) | 1.91 ( 0.54 ) | 0.93 ( -0.92 ) |
| Sudden Death | 2 | 0.39 ( 0.09 - 1.59 ) | 0.39 ( 1.88 ) | 0.41 ( 0.13 ) | -1.29 ( -3 ) |
| Chronic Tonsillitis | 1 | 5.22 ( 0.47 - 57.6 ) | 5.22 ( 2.28 ) | 3.81 ( 0.51 ) | 1.93 ( -0.57 ) |
| Haemophilus Test Positive | 1 | 1.04 ( 0.13 - 8.16 ) | 1.04 ( 0 ) | 1.04 ( 0.19 ) | 0.06 ( -2.15 ) |
| Tonsillar Inflammation | 1 | 2.61 ( 0.29 - 23.36 ) | 2.61 ( 0.8 ) | 2.29 ( 0.37 ) | 1.19 ( -1.16 ) |
| Akinesia | 1 | 3.48 ( 0.36 - 33.47 ) | 3.48 ( 1.33 ) | 2.86 ( 0.43 ) | 1.52 ( -0.9 ) |
| Speech Sound Disorder | 1 | 10.44 ( 0.65 - 167 ) | 10.44 ( 4.27 ) | 5.72 ( 0.56 ) | 2.52 ( -0.12 ) |
| Conjunctival Hyperaemia | 1 | 0.52 ( 0.07 - 3.89 ) | 0.52 ( 0.42 ) | 0.54 ( 0.1 ) | -0.88 ( -3.01 ) |
| Soft Tissue Swelling | 1 | 1.31 ( 0.16 - 10.44 ) | 1.31 ( 0.06 ) | 1.27 ( 0.22 ) | 0.35 ( -1.89 ) |
| Toe Walking | 1 | 0.61 ( 0.08 - 4.62 ) | 0.61 ( 0.23 ) | 0.64 ( 0.12 ) | -0.65 ( -2.8 ) |
| Bradycardia | 1 | 0.17 ( 0.02 - 1.21 ) | 0.17 ( 4.04 ) | 0.18 ( 0.03 ) | -2.46 ( -4.53 ) |
| Hyperglycaemia | 1 | 1.31 ( 0.16 - 10.44 ) | 1.31 ( 0.06 ) | 1.27 ( 0.22 ) | 0.35 ( -1.89 ) |
| Proteinuria | 1 | 1.74 ( 0.21 - 14.46 ) | 1.74 ( 0.27 ) | 1.63 ( 0.28 ) | 0.71 ( -1.57 ) |
| Respiratory Acidosis | 1 | 3.48 ( 0.36 - 33.47 ) | 3.48 ( 1.33 ) | 2.86 ( 0.43 ) | 1.52 ( -0.9 ) |
| Product Dispensing Error | 1 | 0.7 ( 0.09 - 5.27 ) | 0.7 ( 0.12 ) | 0.72 ( 0.13 ) | -0.48 ( -2.64 ) |
| Sedation | 1 | 2.61 ( 0.29 - 23.36 ) | 2.61 ( 0.8 ) | 2.29 ( 0.37 ) | 1.19 ( -1.16 ) |
| Slow Response To Stimuli | 1 | 0.4 ( 0.05 - 2.96 ) | 0.4 ( 0.86 ) | 0.42 ( 0.08 ) | -1.24 ( -3.35 ) |
| Infant Sedation | 1 | 10.44 ( 0.65 - 167 ) | 10.44 ( 4.27 ) | 5.72 ( 0.56 ) | 2.52 ( -0.12 ) |
| Mutism | 1 | 10.44 ( 0.65 - 167 ) | 10.44 ( 4.27 ) | 5.72 ( 0.56 ) | 2.52 ( -0.12 ) |
| Bradykinesia | 1 | 5.22 ( 0.47 - 57.6 ) | 5.22 ( 2.28 ) | 3.81 ( 0.51 ) | 1.93 ( -0.57 ) |
| Product Distribution Issue | 1 | 1.16 ( 0.15 - 9.16 ) | 1.16 ( 0.02 ) | 1.14 ( 0.2 ) | 0.19 ( -2.02 ) |
| Enteric Duplication | 1 | 10.44 ( 0.65 - 167 ) | 10.44 ( 4.27 ) | 5.72 ( 0.56 ) | 2.52 ( -0.12 ) |
| Incorrect Product Administration Duration | 1 | 10.44 ( 0.65 - 167 ) | 10.44 ( 4.27 ) | 5.72 ( 0.56 ) | 2.52 ( -0.12 ) |
| Blood Chloride Decreased | 1 | 3.48 ( 0.36 - 33.47 ) | 3.48 ( 1.33 ) | 2.86 ( 0.43 ) | 1.52 ( -0.9 ) |
| Faecaloma | 1 | 3.48 ( 0.36 - 33.47 ) | 3.48 ( 1.33 ) | 2.86 ( 0.43 ) | 1.52 ( -0.9 ) |
| Brain Natriuretic Peptide Increased | 1 | 3.48 ( 0.36 - 33.47 ) | 3.48 ( 1.33 ) | 2.86 ( 0.43 ) | 1.52 ( -0.9 ) |
| Hyponatraemia | 1 | 0.87 ( 0.11 - 6.69 ) | 0.87 ( 0.02 ) | 0.88 ( 0.16 ) | -0.18 ( -2.37 ) |
| Benign Enlargement Of The Subarachnoid Spaces | 1 | 10.44 ( 0.65 - 167 ) | 10.44 ( 4.27 ) | 5.72 ( 0.56 ) | 2.52 ( -0.12 ) |
| Extrapyramidal Disorder | 1 | 1.74 ( 0.21 - 14.46 ) | 1.74 ( 0.27 ) | 1.63 ( 0.28 ) | 0.71 ( -1.57 ) |
| Escherichia Test Negative | 1 | 1.16 ( 0.15 - 9.16 ) | 1.16 ( 0.02 ) | 1.14 ( 0.2 ) | 0.19 ( -2.02 ) |
| Giardia Test Negative | 1 | 3.48 ( 0.36 - 33.47 ) | 3.48 ( 1.33 ) | 2.86 ( 0.43 ) | 1.52 ( -0.9 ) |
| Upper Respiratory Tract Inflammation | 1 | 1.04 ( 0.13 - 8.16 ) | 1.04 ( 0 ) | 1.04 ( 0.19 ) | 0.06 ( -2.15 ) |
| Hypokinesia | 1 | 0.35 ( 0.05 - 2.55 ) | 0.35 ( 1.18 ) | 0.37 ( 0.07 ) | -1.44 ( -3.54 ) |
| Anti-Ganglioside Antibody Negative | 1 | 5.22 ( 0.47 - 57.6 ) | 5.22 ( 2.28 ) | 3.81 ( 0.51 ) | 1.93 ( -0.57 ) |
| Sputum Discoloured | 1 | 3.48 ( 0.36 - 33.47 ) | 3.48 ( 1.33 ) | 2.86 ( 0.43 ) | 1.52 ( -0.9 ) |
| Acute Disseminated Encephalomyelitis | 1 | 0.58 ( 0.08 - 4.35 ) | 0.58 ( 0.29 ) | 0.6 ( 0.11 ) | -0.73 ( -2.87 ) |
| Varicella Virus Test Negative | 1 | 0.58 ( 0.08 - 4.35 ) | 0.58 ( 0.29 ) | 0.6 ( 0.11 ) | -0.73 ( -2.87 ) |
| Pneumonitis | 1 | 0.47 ( 0.06 - 3.52 ) | 0.47 ( 0.56 ) | 0.5 ( 0.09 ) | -1.01 ( -3.13 ) |
| Adverse Reaction | 1 | 0.37 ( 0.05 - 2.74 ) | 0.37 ( 1.02 ) | 0.39 ( 0.07 ) | -1.34 ( -3.45 ) |
| Allergy Test Negative | 1 | 1.16 ( 0.15 - 9.16 ) | 1.16 ( 0.02 ) | 1.14 ( 0.2 ) | 0.19 ( -2.02 ) |
| Thrombotic Thrombocytopenic Purpura | 1 | 3.48 ( 0.36 - 33.47 ) | 3.48 ( 1.33 ) | 2.86 ( 0.43 ) | 1.52 ( -0.9 ) |
| Lower Respiratory Tract Infection | 1 | 0.8 ( 0.11 - 6.14 ) | 0.8 ( 0.04 ) | 0.82 ( 0.15 ) | -0.29 ( -2.46 ) |
| Ear Inflammation | 1 | 2.09 ( 0.24 - 17.88 ) | 2.09 ( 0.47 ) | 1.91 ( 0.32 ) | 0.93 ( -1.38 ) |
| Pupillary Reflex Impaired | 1 | 5.22 ( 0.47 - 57.6 ) | 5.22 ( 2.28 ) | 3.81 ( 0.51 ) | 1.93 ( -0.57 ) |
| Maternal Exposure During Pregnancy | 1 | 10.44 ( 0.65 - 167 ) | 10.44 ( 4.27 ) | 5.72 ( 0.56 ) | 2.52 ( -0.12 ) |
| Vith Nerve Paralysis | 1 | 1.16 ( 0.15 - 9.16 ) | 1.16 ( 0.02 ) | 1.14 ( 0.2 ) | 0.19 ( -2.02 ) |
| Trismus | 1 | 0.75 ( 0.1 - 5.67 ) | 0.75 ( 0.08 ) | 0.76 ( 0.14 ) | -0.39 ( -2.55 ) |
| Tonsillar Disorder | 1 | 5.22 ( 0.47 - 57.6 ) | 5.22 ( 2.28 ) | 3.81 ( 0.51 ) | 1.93 ( -0.57 ) |
| Lymphadenitis | 1 | 0.47 ( 0.06 - 3.52 ) | 0.47 ( 0.56 ) | 0.5 ( 0.09 ) | -1.01 ( -3.13 ) |
| Ultrasound Doppler Abnormal | 1 | 1.16 ( 0.15 - 9.16 ) | 1.16 ( 0.02 ) | 1.14 ( 0.2 ) | 0.19 ( -2.02 ) |
| Scan With Contrast Abnormal | 1 | 0.61 ( 0.08 - 4.62 ) | 0.61 ( 0.23 ) | 0.64 ( 0.12 ) | -0.65 ( -2.8 ) |
| Skin Swelling | 1 | 0.58 ( 0.08 - 4.35 ) | 0.58 ( 0.29 ) | 0.6 ( 0.11 ) | -0.73 ( -2.87 ) |
| Aspiration Bone Marrow Abnormal | 1 | 2.61 ( 0.29 - 23.36 ) | 2.61 ( 0.8 ) | 2.29 ( 0.37 ) | 1.19 ( -1.16 ) |
| Candida Infection | 1 | 0.95 ( 0.12 - 7.35 ) | 0.95 ( 0 ) | 0.95 ( 0.17 ) | -0.07 ( -2.26 ) |
| Hiv Test Negative | 1 | 1.49 ( 0.18 - 12.13 ) | 1.49 ( 0.14 ) | 1.43 ( 0.25 ) | 0.52 ( -1.74 ) |
| Mumps | 1 | 0.87 ( 0.11 - 6.69 ) | 0.87 ( 0.02 ) | 0.88 ( 0.16 ) | -0.18 ( -2.37 ) |
| Rubella | 1 | 0.61 ( 0.08 - 4.62 ) | 0.61 ( 0.23 ) | 0.64 ( 0.12 ) | -0.65 ( -2.8 ) |
| Acute Respiratory Distress Syndrome | 1 | 1.74 ( 0.21 - 14.46 ) | 1.74 ( 0.27 ) | 1.63 ( 0.28 ) | 0.71 ( -1.57 ) |
| Hyperferritinaemia | 1 | 10.44 ( 0.65 - 167 ) | 10.44 ( 4.27 ) | 5.72 ( 0.56 ) | 2.52 ( -0.12 ) |
| Natural Killer Cell Count Decreased | 1 | 2.61 ( 0.29 - 23.36 ) | 2.61 ( 0.8 ) | 2.29 ( 0.37 ) | 1.19 ( -1.16 ) |
| Toxic Shock Syndrome | 1 | 10.44 ( 0.65 - 167 ) | 10.44 ( 4.27 ) | 5.72 ( 0.56 ) | 2.52 ( -0.12 ) |
| Biopsy Liver Abnormal | 1 | 5.22 ( 0.47 - 57.6 ) | 5.22 ( 2.28 ) | 3.81 ( 0.51 ) | 1.93 ( -0.57 ) |
| Blood Immunoglobulin M | 1 | 0.44 ( 0.06 - 3.22 ) | 0.44 ( 0.7 ) | 0.46 ( 0.09 ) | -1.13 ( -3.24 ) |
| Haemophagocytic Lymphohistiocytosis | 1 | 0.55 ( 0.07 - 4.11 ) | 0.55 ( 0.35 ) | 0.57 ( 0.11 ) | -0.81 ( -2.94 ) |
| Marrow Hyperplasia | 1 | 2.61 ( 0.29 - 23.36 ) | 2.61 ( 0.8 ) | 2.29 ( 0.37 ) | 1.19 ( -1.16 ) |
| Natural Killer Cell Count Increased | 1 | 2.09 ( 0.24 - 17.88 ) | 2.09 ( 0.47 ) | 1.91 ( 0.32 ) | 0.93 ( -1.38 ) |
| Blood Culture Positive | 1 | 0.65 ( 0.09 - 4.92 ) | 0.65 ( 0.17 ) | 0.67 ( 0.12 ) | -0.57 ( -2.72 ) |
| Epstein-Barr Virus Infection | 1 | 1.31 ( 0.16 - 10.44 ) | 1.31 ( 0.06 ) | 1.27 ( 0.22 ) | 0.35 ( -1.89 ) |
| Hepatitis | 1 | 1.16 ( 0.15 - 9.16 ) | 1.16 ( 0.02 ) | 1.14 ( 0.2 ) | 0.19 ( -2.02 ) |
| Rhabdomyolysis | 1 | 10.44 ( 0.65 - 167 ) | 10.44 ( 4.27 ) | 5.72 ( 0.56 ) | 2.52 ( -0.12 ) |
| Cd4 Lymphocytes Decreased | 1 | 1.04 ( 0.13 - 8.16 ) | 1.04 ( 0 ) | 1.04 ( 0.19 ) | 0.06 ( -2.15 ) |
| Epstein-Barr Virus Test Positive | 1 | 1.49 ( 0.18 - 12.13 ) | 1.49 ( 0.14 ) | 1.43 ( 0.25 ) | 0.52 ( -1.74 ) |
| Hepatosplenomegaly | 1 | 1.49 ( 0.18 - 12.13 ) | 1.49 ( 0.14 ) | 1.43 ( 0.25 ) | 0.52 ( -1.74 ) |
| Multiple Organ Dysfunction Syndrome | 1 | 0.95 ( 0.12 - 7.35 ) | 0.95 ( 0 ) | 0.95 ( 0.17 ) | -0.07 ( -2.26 ) |
| Panic Attack | 1 | 10.44 ( 0.65 - 167 ) | 10.44 ( 4.27 ) | 5.72 ( 0.56 ) | 2.52 ( -0.12 ) |
| Tension | 1 | 0.52 ( 0.07 - 3.89 ) | 0.52 ( 0.42 ) | 0.54 ( 0.1 ) | -0.88 ( -3.01 ) |
| Exaggerated Startle Response | 1 | 3.48 ( 0.36 - 33.47 ) | 3.48 ( 1.33 ) | 2.86 ( 0.43 ) | 1.52 ( -0.9 ) |
| Feeling Jittery | 1 | 3.48 ( 0.36 - 33.47 ) | 3.48 ( 1.33 ) | 2.86 ( 0.43 ) | 1.52 ( -0.9 ) |
| Generalised Oedema | 1 | 0.95 ( 0.12 - 7.35 ) | 0.95 ( 0 ) | 0.95 ( 0.17 ) | -0.07 ( -2.26 ) |
| Skin Induration | 1 | 1.31 ( 0.16 - 10.44 ) | 1.31 ( 0.06 ) | 1.27 ( 0.22 ) | 0.35 ( -1.89 ) |
| Otitis Media Chronic | 1 | 2.61 ( 0.29 - 23.36 ) | 2.61 ( 0.8 ) | 2.29 ( 0.37 ) | 1.19 ( -1.16 ) |
| Activated Partial Thromboplastin Time Shortened | 1 | 1.49 ( 0.18 - 12.13 ) | 1.49 ( 0.14 ) | 1.43 ( 0.25 ) | 0.52 ( -1.74 ) |
| Red Blood Cell Nucleated Morphology | 1 | 2.61 ( 0.29 - 23.36 ) | 2.61 ( 0.8 ) | 2.29 ( 0.37 ) | 1.19 ( -1.16 ) |
| Disturbance In Attention | 1 | 0.3 ( 0.04 - 2.18 ) | 0.3 ( 1.6 ) | 0.32 ( 0.06 ) | -1.65 ( -3.75 ) |
| Ileus Paralytic | 1 | 5.22 ( 0.47 - 57.6 ) | 5.22 ( 2.28 ) | 3.81 ( 0.51 ) | 1.93 ( -0.57 ) |
| Barium Enema Abnormal | 1 | 0.8 ( 0.11 - 6.14 ) | 0.8 ( 0.04 ) | 0.82 ( 0.15 ) | -0.29 ( -2.46 ) |
| Myositis | 1 | 0.7 ( 0.09 - 5.27 ) | 0.7 ( 0.12 ) | 0.72 ( 0.13 ) | -0.48 ( -2.64 ) |
| Infection Susceptibility Increased | 1 | 0.7 ( 0.09 - 5.27 ) | 0.7 ( 0.12 ) | 0.72 ( 0.13 ) | -0.48 ( -2.64 ) |
| Eosinophilic Oesophagitis | 1 | 10.44 ( 0.65 - 167 ) | 10.44 ( 4.27 ) | 5.72 ( 0.56 ) | 2.52 ( -0.12 ) |
| Barium Double Contrast Abnormal | 1 | 10.44 ( 0.65 - 167 ) | 10.44 ( 4.27 ) | 5.72 ( 0.56 ) | 2.52 ( -0.12 ) |
| Mastoiditis | 1 | 2.09 ( 0.24 - 17.88 ) | 2.09 ( 0.47 ) | 1.91 ( 0.32 ) | 0.93 ( -1.38 ) |
| Indifference | 1 | 10.44 ( 0.65 - 167 ) | 10.44 ( 4.27 ) | 5.72 ( 0.56 ) | 2.52 ( -0.12 ) |
| Urticaria Papular | 1 | 5.22 ( 0.47 - 57.6 ) | 5.22 ( 2.28 ) | 3.81 ( 0.51 ) | 1.93 ( -0.57 ) |
| Localised Oedema | 1 | 0.58 ( 0.08 - 4.35 ) | 0.58 ( 0.29 ) | 0.6 ( 0.11 ) | -0.73 ( -2.87 ) |
| Weight Increased | 1 | 2.61 ( 0.29 - 23.36 ) | 2.61 ( 0.8 ) | 2.29 ( 0.37 ) | 1.19 ( -1.16 ) |
| Intestinal Ischaemia | 1 | 10.44 ( 0.65 - 167 ) | 10.44 ( 4.27 ) | 5.72 ( 0.56 ) | 2.52 ( -0.12 ) |
| Cerumen Impaction | 1 | 5.22 ( 0.47 - 57.6 ) | 5.22 ( 2.28 ) | 3.81 ( 0.51 ) | 1.93 ( -0.57 ) |
| Use Of Accessory Respiratory Muscles | 1 | 0.52 ( 0.07 - 3.89 ) | 0.52 ( 0.42 ) | 0.54 ( 0.1 ) | -0.88 ( -3.01 ) |
| Tympanic Membrane Disorder | 1 | 3.48 ( 0.36 - 33.47 ) | 3.48 ( 1.33 ) | 2.86 ( 0.43 ) | 1.52 ( -0.9 ) |
| Respiratory Disorder | 1 | 0.31 ( 0.04 - 2.24 ) | 0.31 ( 1.52 ) | 0.33 ( 0.06 ) | -1.61 ( -3.71 ) |
| Abdominal Mass | 1 | 0.55 ( 0.07 - 4.11 ) | 0.55 ( 0.35 ) | 0.57 ( 0.11 ) | -0.81 ( -2.94 ) |
| Appendix Disorder | 1 | 10.44 ( 0.65 - 167 ) | 10.44 ( 4.27 ) | 5.72 ( 0.56 ) | 2.52 ( -0.12 ) |
| Intestinal Perforation | 1 | 0.8 ( 0.11 - 6.14 ) | 0.8 ( 0.04 ) | 0.82 ( 0.15 ) | -0.29 ( -2.46 ) |
| Functional Gastrointestinal Disorder | 1 | 1.49 ( 0.18 - 12.13 ) | 1.49 ( 0.14 ) | 1.43 ( 0.25 ) | 0.52 ( -1.74 ) |
| Micturition Disorder | 1 | 2.09 ( 0.24 - 17.88 ) | 2.09 ( 0.47 ) | 1.91 ( 0.32 ) | 0.93 ( -1.38 ) |
| Otoscopy Abnormal | 1 | 10.44 ( 0.65 - 167 ) | 10.44 ( 4.27 ) | 5.72 ( 0.56 ) | 2.52 ( -0.12 ) |
| Mydriasis | 1 | 1.31 ( 0.16 - 10.44 ) | 1.31 ( 0.06 ) | 1.27 ( 0.22 ) | 0.35 ( -1.89 ) |
| Multisystem Inflammatory Syndrome In Children | 1 | 2.61 ( 0.29 - 23.36 ) | 2.61 ( 0.8 ) | 2.29 ( 0.37 ) | 1.19 ( -1.16 ) |
| Red Blood Cell Count Increased | 1 | 3.48 ( 0.36 - 33.47 ) | 3.48 ( 1.33 ) | 2.86 ( 0.43 ) | 1.52 ( -0.9 ) |
| Mucosal Haemorrhage | 1 | 1.16 ( 0.15 - 9.16 ) | 1.16 ( 0.02 ) | 1.14 ( 0.2 ) | 0.19 ( -2.02 ) |
| Dermatitis | 1 | 0.32 ( 0.04 - 2.31 ) | 0.32 ( 1.43 ) | 0.34 ( 0.06 ) | -1.57 ( -3.67 ) |
| Disease Recurrence | 1 | 10.44 ( 0.65 - 167 ) | 10.44 ( 4.27 ) | 5.72 ( 0.56 ) | 2.52 ( -0.12 ) |
| Tachypnoea | 1 | 0.2 ( 0.03 - 1.48 ) | 0.2 ( 3.03 ) | 0.22 ( 0.04 ) | -2.18 ( -4.26 ) |
| Type Iii Immune Complex Mediated Reaction | 1 | 0.65 ( 0.09 - 4.92 ) | 0.65 ( 0.17 ) | 0.67 ( 0.12 ) | -0.57 ( -2.72 ) |
| Dysphemia | 1 | 0.61 ( 0.08 - 4.62 ) | 0.61 ( 0.23 ) | 0.64 ( 0.12 ) | -0.65 ( -2.8 ) |
| Epstein-Barr Virus Test Negative | 1 | 0.44 ( 0.06 - 3.22 ) | 0.44 ( 0.7 ) | 0.46 ( 0.09 ) | -1.13 ( -3.24 ) |
| Kidney Small | 1 | 10.44 ( 0.65 - 167 ) | 10.44 ( 4.27 ) | 5.72 ( 0.56 ) | 2.52 ( -0.12 ) |
| Therapy Non-Responder | 1 | 1.31 ( 0.16 - 10.44 ) | 1.31 ( 0.06 ) | 1.27 ( 0.22 ) | 0.35 ( -1.89 ) |
| Coronavirus Test Negative | 1 | 1.74 ( 0.21 - 14.46 ) | 1.74 ( 0.27 ) | 1.63 ( 0.28 ) | 0.71 ( -1.57 ) |
| Anti-Thyroid Antibody Negative | 1 | 5.22 ( 0.47 - 57.6 ) | 5.22 ( 2.28 ) | 3.81 ( 0.51 ) | 1.93 ( -0.57 ) |
| Haematology Test Abnormal | 1 | 3.48 ( 0.36 - 33.47 ) | 3.48 ( 1.33 ) | 2.86 ( 0.43 ) | 1.52 ( -0.9 ) |
| Antinuclear Antibody Positive | 1 | 5.22 ( 0.47 - 57.6 ) | 5.22 ( 2.28 ) | 3.81 ( 0.51 ) | 1.93 ( -0.57 ) |
| Antiphospholipid Antibodies Negative | 1 | 5.22 ( 0.47 - 57.6 ) | 5.22 ( 2.28 ) | 3.81 ( 0.51 ) | 1.93 ( -0.57 ) |
| Complement Factor C4 Decreased | 1 | 10.44 ( 0.65 - 167 ) | 10.44 ( 4.27 ) | 5.72 ( 0.56 ) | 2.52 ( -0.12 ) |
| Meningococcal Bacteraemia | 1 | 5.22 ( 0.47 - 57.6 ) | 5.22 ( 2.28 ) | 3.81 ( 0.51 ) | 1.93 ( -0.57 ) |
| Myelosuppression | 1 | 5.22 ( 0.47 - 57.6 ) | 5.22 ( 2.28 ) | 3.81 ( 0.51 ) | 1.93 ( -0.57 ) |
| Hepatomegaly | 1 | 1.31 ( 0.16 - 10.44 ) | 1.31 ( 0.06 ) | 1.27 ( 0.22 ) | 0.35 ( -1.89 ) |
| Activated Partial Thromboplastin Time Prolonged | 1 | 0.95 ( 0.12 - 7.35 ) | 0.95 ( 0 ) | 0.95 ( 0.17 ) | -0.07 ( -2.26 ) |
| Blood Immunoglobulin A Decreased | 1 | 0.65 ( 0.09 - 4.92 ) | 0.65 ( 0.17 ) | 0.67 ( 0.12 ) | -0.57 ( -2.72 ) |
| Blood Immunoglobulin M Increased | 1 | 1.49 ( 0.18 - 12.13 ) | 1.49 ( 0.14 ) | 1.43 ( 0.25 ) | 0.52 ( -1.74 ) |
| Blood Electrolytes Abnormal | 1 | 10.44 ( 0.65 - 167 ) | 10.44 ( 4.27 ) | 5.72 ( 0.56 ) | 2.52 ( -0.12 ) |
| Blood Ph Increased | 1 | 1.49 ( 0.18 - 12.13 ) | 1.49 ( 0.14 ) | 1.43 ( 0.25 ) | 0.52 ( -1.74 ) |
| Haemoglobin Increased | 1 | 0.47 ( 0.06 - 3.52 ) | 0.47 ( 0.56 ) | 0.5 ( 0.09 ) | -1.01 ( -3.13 ) |
| Brief Resolved Unexplained Event | 1 | 0.87 ( 0.11 - 6.69 ) | 0.87 ( 0.02 ) | 0.88 ( 0.16 ) | -0.18 ( -2.37 ) |
| Ultrasound Head Abnormal | 1 | 2.61 ( 0.29 - 23.36 ) | 2.61 ( 0.8 ) | 2.29 ( 0.37 ) | 1.19 ( -1.16 ) |
| Gastrointestinal Wall Thickening | 1 | 1.31 ( 0.16 - 10.44 ) | 1.31 ( 0.06 ) | 1.27 ( 0.22 ) | 0.35 ( -1.89 ) |
| Blood Creatine Phosphokinase Mb Increased | 1 | 0.87 ( 0.11 - 6.69 ) | 0.87 ( 0.02 ) | 0.88 ( 0.16 ) | -0.18 ( -2.37 ) |
| Blood Albumin Increased | 1 | 1.49 ( 0.18 - 12.13 ) | 1.49 ( 0.14 ) | 1.43 ( 0.25 ) | 0.52 ( -1.74 ) |
| Blood Creatine Phosphokinase Increased | 1 | 0.55 ( 0.07 - 4.11 ) | 0.55 ( 0.35 ) | 0.57 ( 0.11 ) | -0.81 ( -2.94 ) |
| Cerebrovascular Accident | 1 | 1.74 ( 0.21 - 14.46 ) | 1.74 ( 0.27 ) | 1.63 ( 0.28 ) | 0.71 ( -1.57 ) |
| Blood Bicarbonate Increased | 1 | 2.61 ( 0.29 - 23.36 ) | 2.61 ( 0.8 ) | 2.29 ( 0.37 ) | 1.19 ( -1.16 ) |
| Blood Ph Decreased | 1 | 0.47 ( 0.06 - 3.52 ) | 0.47 ( 0.56 ) | 0.5 ( 0.09 ) | -1.01 ( -3.13 ) |
| Pco2 Increased | 1 | 1.31 ( 0.16 - 10.44 ) | 1.31 ( 0.06 ) | 1.27 ( 0.22 ) | 0.35 ( -1.89 ) |
| Bronchospasm | 1 | 0.5 ( 0.07 - 3.7 ) | 0.5 ( 0.49 ) | 0.52 ( 0.1 ) | -0.94 ( -3.07 ) |
| C-Reactive Protein Decreased | 1 | 0.95 ( 0.12 - 7.35 ) | 0.95 ( 0 ) | 0.95 ( 0.17 ) | -0.07 ( -2.26 ) |
| Febrile Status Epilepticus | 1 | 5.22 ( 0.47 - 57.6 ) | 5.22 ( 2.28 ) | 3.81 ( 0.51 ) | 1.93 ( -0.57 ) |
| Aphthous Ulcer | 1 | 1.04 ( 0.13 - 8.16 ) | 1.04 ( 0 ) | 1.04 ( 0.19 ) | 0.06 ( -2.15 ) |
| Bone Scan Abnormal | 1 | 5.22 ( 0.47 - 57.6 ) | 5.22 ( 2.28 ) | 3.81 ( 0.51 ) | 1.93 ( -0.57 ) |
| Fluid Retention | 1 | 1.31 ( 0.16 - 10.44 ) | 1.31 ( 0.06 ) | 1.27 ( 0.22 ) | 0.35 ( -1.89 ) |
| Salivary Hypersecretion | 1 | 0.2 ( 0.03 - 1.48 ) | 0.2 ( 3.03 ) | 0.22 ( 0.04 ) | -2.18 ( -4.26 ) |
| Synovitis | 1 | 0.7 ( 0.09 - 5.27 ) | 0.7 ( 0.12 ) | 0.72 ( 0.13 ) | -0.48 ( -2.64 ) |
| Serum Ferritin Decreased | 1 | 5.22 ( 0.47 - 57.6 ) | 5.22 ( 2.28 ) | 3.81 ( 0.51 ) | 1.93 ( -0.57 ) |
| Haemangioma | 1 | 3.48 ( 0.36 - 33.47 ) | 3.48 ( 1.33 ) | 2.86 ( 0.43 ) | 1.52 ( -0.9 ) |
| Meningitis Viral | 1 | 1.04 ( 0.13 - 8.16 ) | 1.04 ( 0 ) | 1.04 ( 0.19 ) | 0.06 ( -2.15 ) |
| Pleocytosis | 1 | 2.09 ( 0.24 - 17.88 ) | 2.09 ( 0.47 ) | 1.91 ( 0.32 ) | 0.93 ( -1.38 ) |
| Csf Test Abnormal | 1 | 0.45 ( 0.06 - 3.36 ) | 0.45 ( 0.63 ) | 0.48 ( 0.09 ) | -1.07 ( -3.19 ) |
| Brain Oedema | 1 | 0.45 ( 0.06 - 3.36 ) | 0.45 ( 0.63 ) | 0.48 ( 0.09 ) | -1.07 ( -3.19 ) |
| Septic Shock | 1 | 0.87 ( 0.11 - 6.69 ) | 0.87 ( 0.02 ) | 0.88 ( 0.16 ) | -0.18 ( -2.37 ) |
| Capillary Nail Refill Test Abnormal | 1 | 1.31 ( 0.16 - 10.44 ) | 1.31 ( 0.06 ) | 1.27 ( 0.22 ) | 0.35 ( -1.89 ) |
| Product Formulation Issue | 1 | 5.22 ( 0.47 - 57.6 ) | 5.22 ( 2.28 ) | 3.81 ( 0.51 ) | 1.93 ( -0.57 ) |
| Gastroenteritis Viral | 1 | 0.8 ( 0.11 - 6.14 ) | 0.8 ( 0.04 ) | 0.82 ( 0.15 ) | -0.29 ( -2.46 ) |
| Bacteraemia | 1 | 0.95 ( 0.12 - 7.35 ) | 0.95 ( 0 ) | 0.95 ( 0.17 ) | -0.07 ( -2.26 ) |
| Lacrimation Disorder | 1 | 5.22 ( 0.47 - 57.6 ) | 5.22 ( 2.28 ) | 3.81 ( 0.51 ) | 1.93 ( -0.57 ) |
| Laparoscopy Abnormal | 1 | 10.44 ( 0.65 - 167 ) | 10.44 ( 4.27 ) | 5.72 ( 0.56 ) | 2.52 ( -0.12 ) |
| Imaging Procedure Abnormal | 1 | 1.04 ( 0.13 - 8.16 ) | 1.04 ( 0 ) | 1.04 ( 0.19 ) | 0.06 ( -2.15 ) |
| Exanthema Subitum | 1 | 0.55 ( 0.07 - 4.11 ) | 0.55 ( 0.35 ) | 0.57 ( 0.11 ) | -0.81 ( -2.94 ) |
| Vaccination Site Haematoma | 1 | 1.74 ( 0.21 - 14.46 ) | 1.74 ( 0.27 ) | 1.63 ( 0.28 ) | 0.71 ( -1.57 ) |
| Anger | 1 | 0.55 ( 0.07 - 4.11 ) | 0.55 ( 0.35 ) | 0.57 ( 0.11 ) | -0.81 ( -2.94 ) |
| Confusional State | 1 | 0.27 ( 0.04 - 1.95 ) | 0.27 ( 1.95 ) | 0.29 ( 0.05 ) | -1.81 ( -3.9 ) |
| Accidental Overdose | 1 | 0.19 ( 0.03 - 1.35 ) | 0.19 ( 3.49 ) | 0.2 ( 0.04 ) | -2.32 ( -4.39 ) |
| Culture Urine Positive | 1 | 0.52 ( 0.07 - 3.89 ) | 0.52 ( 0.42 ) | 0.54 ( 0.1 ) | -0.88 ( -3.01 ) |
| Pyelonephritis | 1 | 1.16 ( 0.15 - 9.16 ) | 1.16 ( 0.02 ) | 1.14 ( 0.2 ) | 0.19 ( -2.02 ) |
| Urine Leukocyte Esterase Positive | 1 | 2.61 ( 0.29 - 23.36 ) | 2.61 ( 0.8 ) | 2.29 ( 0.37 ) | 1.19 ( -1.16 ) |
| Urinary Tract Imaging Abnormal | 1 | 10.44 ( 0.65 - 167 ) | 10.44 ( 4.27 ) | 5.72 ( 0.56 ) | 2.52 ( -0.12 ) |
| Body Temperature Abnormal | 1 | 1.49 ( 0.18 - 12.13 ) | 1.49 ( 0.14 ) | 1.43 ( 0.25 ) | 0.52 ( -1.74 ) |
| Pneumonia Bacterial | 1 | 3.48 ( 0.36 - 33.47 ) | 3.48 ( 1.33 ) | 2.86 ( 0.43 ) | 1.52 ( -0.9 ) |
| Gastroenteritis Norovirus | 1 | 1.04 ( 0.13 - 8.16 ) | 1.04 ( 0 ) | 1.04 ( 0.19 ) | 0.06 ( -2.15 ) |
| Parainfluenzae Virus Infection | 1 | 1.49 ( 0.18 - 12.13 ) | 1.49 ( 0.14 ) | 1.43 ( 0.25 ) | 0.52 ( -1.74 ) |
| Haemangioma Of Skin | 1 | 10.44 ( 0.65 - 167 ) | 10.44 ( 4.27 ) | 5.72 ( 0.56 ) | 2.52 ( -0.12 ) |
| Abdominal Rigidity | 1 | 2.61 ( 0.29 - 23.36 ) | 2.61 ( 0.8 ) | 2.29 ( 0.37 ) | 1.19 ( -1.16 ) |
| Hand-Foot-And-Mouth Disease | 1 | 0.32 ( 0.04 - 2.31 ) | 0.32 ( 1.43 ) | 0.34 ( 0.06 ) | -1.57 ( -3.67 ) |
| Autoimmune Haemolytic Anaemia | 1 | 1.74 ( 0.21 - 14.46 ) | 1.74 ( 0.27 ) | 1.63 ( 0.28 ) | 0.71 ( -1.57 ) |
| Blood Bilirubin Increased | 1 | 0.75 ( 0.1 - 5.67 ) | 0.75 ( 0.08 ) | 0.76 ( 0.14 ) | -0.39 ( -2.55 ) |
| Toxoplasma Serology Positive | 1 | 10.44 ( 0.65 - 167 ) | 10.44 ( 4.27 ) | 5.72 ( 0.56 ) | 2.52 ( -0.12 ) |
| Complement Factor C3 Increased | 1 | 5.22 ( 0.47 - 57.6 ) | 5.22 ( 2.28 ) | 3.81 ( 0.51 ) | 1.93 ( -0.57 ) |
| Bilirubin Conjugated Decreased | 1 | 1.49 ( 0.18 - 12.13 ) | 1.49 ( 0.14 ) | 1.43 ( 0.25 ) | 0.52 ( -1.74 ) |
| Antibody Test Positive | 1 | 0.7 ( 0.09 - 5.27 ) | 0.7 ( 0.12 ) | 0.72 ( 0.13 ) | -0.48 ( -2.64 ) |
| Biopsy Bone Marrow Abnormal | 1 | 1.31 ( 0.16 - 10.44 ) | 1.31 ( 0.06 ) | 1.27 ( 0.22 ) | 0.35 ( -1.89 ) |
| Blood Calcium Decreased | 1 | 0.61 ( 0.08 - 4.62 ) | 0.61 ( 0.23 ) | 0.64 ( 0.12 ) | -0.65 ( -2.8 ) |
| Tonic Posturing | 1 | 3.48 ( 0.36 - 33.47 ) | 3.48 ( 1.33 ) | 2.86 ( 0.43 ) | 1.52 ( -0.9 ) |
| Torticollis | 1 | 1.74 ( 0.21 - 14.46 ) | 1.74 ( 0.27 ) | 1.63 ( 0.28 ) | 0.71 ( -1.57 ) |
| Anti-Platelet Antibody Positive | 1 | 2.61 ( 0.29 - 23.36 ) | 2.61 ( 0.8 ) | 2.29 ( 0.37 ) | 1.19 ( -1.16 ) |
| Critical Illness | 1 | 3.48 ( 0.36 - 33.47 ) | 3.48 ( 1.33 ) | 2.86 ( 0.43 ) | 1.52 ( -0.9 ) |
| Reticulocyte Percentage Increased | 1 | 10.44 ( 0.65 - 167 ) | 10.44 ( 4.27 ) | 5.72 ( 0.56 ) | 2.52 ( -0.12 ) |
| Cytomegalovirus Infection | 1 | 0.58 ( 0.08 - 4.35 ) | 0.58 ( 0.29 ) | 0.6 ( 0.11 ) | -0.73 ( -2.87 ) |
| Haemorrhage Subcutaneous | 1 | 0.2 ( 0.03 - 1.42 ) | 0.2 ( 3.21 ) | 0.21 ( 0.04 ) | -2.24 ( -4.32 ) |
| Rhesus Antibodies Positive | 1 | 5.22 ( 0.47 - 57.6 ) | 5.22 ( 2.28 ) | 3.81 ( 0.51 ) | 1.93 ( -0.57 ) |
| Tricuspid Valve Incompetence | 1 | 1.74 ( 0.21 - 14.46 ) | 1.74 ( 0.27 ) | 1.63 ( 0.28 ) | 0.71 ( -1.57 ) |
| Cytomegalovirus Test Positive | 1 | 0.44 ( 0.06 - 3.22 ) | 0.44 ( 0.7 ) | 0.46 ( 0.09 ) | -1.13 ( -3.24 ) |
| Laryngeal Oedema | 1 | 2.61 ( 0.29 - 23.36 ) | 2.61 ( 0.8 ) | 2.29 ( 0.37 ) | 1.19 ( -1.16 ) |
| Attention Deficit Hyperactivity Disorder | 1 | 0.75 ( 0.1 - 5.67 ) | 0.75 ( 0.08 ) | 0.76 ( 0.14 ) | -0.39 ( -2.55 ) |
| Subdural Effusion | 1 | 10.44 ( 0.65 - 167 ) | 10.44 ( 4.27 ) | 5.72 ( 0.56 ) | 2.52 ( -0.12 ) |
| Thermometry Abnormal | 1 | 10.44 ( 0.65 - 167 ) | 10.44 ( 4.27 ) | 5.72 ( 0.56 ) | 2.52 ( -0.12 ) |
| Seborrhoeic Dermatitis | 1 | 1.74 ( 0.21 - 14.46 ) | 1.74 ( 0.27 ) | 1.63 ( 0.28 ) | 0.71 ( -1.57 ) |
| Delirium | 1 | 1.74 ( 0.21 - 14.46 ) | 1.74 ( 0.27 ) | 1.63 ( 0.28 ) | 0.71 ( -1.57 ) |
| Red Blood Cell Schistocytes Present | 1 | 5.22 ( 0.47 - 57.6 ) | 5.22 ( 2.28 ) | 3.81 ( 0.51 ) | 1.93 ( -0.57 ) |
| Haemolytic Uraemic Syndrome | 1 | 1.04 ( 0.13 - 8.16 ) | 1.04 ( 0 ) | 1.04 ( 0.19 ) | 0.06 ( -2.15 ) |
| Atypical Haemolytic Uraemic Syndrome | 1 | 3.48 ( 0.36 - 33.47 ) | 3.48 ( 1.33 ) | 2.86 ( 0.43 ) | 1.52 ( -0.9 ) |
| Blood Creatinine Increased | 1 | 0.55 ( 0.07 - 4.11 ) | 0.55 ( 0.35 ) | 0.57 ( 0.11 ) | -0.81 ( -2.94 ) |
| Infantile Colic | 1 | 2.09 ( 0.24 - 17.88 ) | 2.09 ( 0.47 ) | 1.91 ( 0.32 ) | 0.93 ( -1.38 ) |
| Aspiration | 1 | 0.58 ( 0.08 - 4.35 ) | 0.58 ( 0.29 ) | 0.6 ( 0.11 ) | -0.73 ( -2.87 ) |
| Skin Ulcer | 1 | 0.87 ( 0.11 - 6.69 ) | 0.87 ( 0.02 ) | 0.88 ( 0.16 ) | -0.18 ( -2.37 ) |
| Aversion | 1 | 5.22 ( 0.47 - 57.6 ) | 5.22 ( 2.28 ) | 3.81 ( 0.51 ) | 1.93 ( -0.57 ) |
| Oral Candidiasis | 1 | 1.16 ( 0.15 - 9.16 ) | 1.16 ( 0.02 ) | 1.14 ( 0.2 ) | 0.19 ( -2.02 ) |
| Chest Pain | 1 | 0.87 ( 0.11 - 6.69 ) | 0.87 ( 0.02 ) | 0.88 ( 0.16 ) | -0.18 ( -2.37 ) |
| Food Aversion | 1 | 0.58 ( 0.08 - 4.35 ) | 0.58 ( 0.29 ) | 0.6 ( 0.11 ) | -0.73 ( -2.87 ) |
| Coronary Artery Aneurysm | 1 | 1.49 ( 0.18 - 12.13 ) | 1.49 ( 0.14 ) | 1.43 ( 0.25 ) | 0.52 ( -1.74 ) |
| Electrocardiogram St Segment Elevation | 1 | 10.44 ( 0.65 - 167 ) | 10.44 ( 4.27 ) | 5.72 ( 0.56 ) | 2.52 ( -0.12 ) |
| Troponin T Increased | 1 | 10.44 ( 0.65 - 167 ) | 10.44 ( 4.27 ) | 5.72 ( 0.56 ) | 2.52 ( -0.12 ) |
| Idiopathic Intracranial Hypertension | 1 | 5.22 ( 0.47 - 57.6 ) | 5.22 ( 2.28 ) | 3.81 ( 0.51 ) | 1.93 ( -0.57 ) |
| Staphylococcus Test Negative | 1 | 3.48 ( 0.36 - 33.47 ) | 3.48 ( 1.33 ) | 2.86 ( 0.43 ) | 1.52 ( -0.9 ) |
| Red Blood Cells Urine Negative | 1 | 0.87 ( 0.11 - 6.69 ) | 0.87 ( 0.02 ) | 0.88 ( 0.16 ) | -0.18 ( -2.37 ) |
| Urobilinogen Urine Decreased | 1 | 2.09 ( 0.24 - 17.88 ) | 2.09 ( 0.47 ) | 1.91 ( 0.32 ) | 0.93 ( -1.38 ) |
| Bacterial Test Positive | 1 | 0.39 ( 0.05 - 2.85 ) | 0.39 ( 0.94 ) | 0.41 ( 0.08 ) | -1.29 ( -3.4 ) |
| Histology Abnormal | 1 | 10.44 ( 0.65 - 167 ) | 10.44 ( 4.27 ) | 5.72 ( 0.56 ) | 2.52 ( -0.12 ) |
| Immunology Test Abnormal | 1 | 0.87 ( 0.11 - 6.69 ) | 0.87 ( 0.02 ) | 0.88 ( 0.16 ) | -0.18 ( -2.37 ) |
| Eosinophilia | 1 | 1.04 ( 0.13 - 8.16 ) | 1.04 ( 0 ) | 1.04 ( 0.19 ) | 0.06 ( -2.15 ) |
| Stool Analysis Abnormal | 1 | 0.22 ( 0.03 - 1.58 ) | 0.22 ( 2.76 ) | 0.23 ( 0.04 ) | -2.1 ( -4.18 ) |
| Laryngospasm | 1 | 0.95 ( 0.12 - 7.35 ) | 0.95 ( 0 ) | 0.95 ( 0.17 ) | -0.07 ( -2.26 ) |
| Oral Mucosal Blistering | 1 | 1.49 ( 0.18 - 12.13 ) | 1.49 ( 0.14 ) | 1.43 ( 0.25 ) | 0.52 ( -1.74 ) |
| Wrong Patient | 1 | 1.31 ( 0.16 - 10.44 ) | 1.31 ( 0.06 ) | 1.27 ( 0.22 ) | 0.35 ( -1.89 ) |
| Hypertrophic Cardiomyopathy | 1 | 10.44 ( 0.65 - 167 ) | 10.44 ( 4.27 ) | 5.72 ( 0.56 ) | 2.52 ( -0.12 ) |
| Vitreous Floaters | 1 | 10.44 ( 0.65 - 167 ) | 10.44 ( 4.27 ) | 5.72 ( 0.56 ) | 2.52 ( -0.12 ) |
| Cytogenetic Abnormality | 1 | 10.44 ( 0.65 - 167 ) | 10.44 ( 4.27 ) | 5.72 ( 0.56 ) | 2.52 ( -0.12 ) |
| Psychomotor Skills Impaired | 1 | 0.35 ( 0.05 - 2.55 ) | 0.35 ( 1.18 ) | 0.37 ( 0.07 ) | -1.44 ( -3.54 ) |
| Lactic Acidosis | 1 | 2.61 ( 0.29 - 23.36 ) | 2.61 ( 0.8 ) | 2.29 ( 0.37 ) | 1.19 ( -1.16 ) |
| Gene Mutation Identification Test Negative | 1 | 5.22 ( 0.47 - 57.6 ) | 5.22 ( 2.28 ) | 3.81 ( 0.51 ) | 1.93 ( -0.57 ) |
| Apoptosis | 1 | 10.44 ( 0.65 - 167 ) | 10.44 ( 4.27 ) | 5.72 ( 0.56 ) | 2.52 ( -0.12 ) |
| Electrocardiogram T Wave Inversion | 1 | 10.44 ( 0.65 - 167 ) | 10.44 ( 4.27 ) | 5.72 ( 0.56 ) | 2.52 ( -0.12 ) |
| Electrocardiogram Ambulatory Abnormal | 1 | 10.44 ( 0.65 - 167 ) | 10.44 ( 4.27 ) | 5.72 ( 0.56 ) | 2.52 ( -0.12 ) |
| Yellow Skin | 1 | 1.31 ( 0.16 - 10.44 ) | 1.31 ( 0.06 ) | 1.27 ( 0.22 ) | 0.35 ( -1.89 ) |
| Neurodevelopmental Disorder | 1 | 1.49 ( 0.18 - 12.13 ) | 1.49 ( 0.14 ) | 1.43 ( 0.25 ) | 0.52 ( -1.74 ) |
| Tongue Discolouration | 1 | 3.48 ( 0.36 - 33.47 ) | 3.48 ( 1.33 ) | 2.86 ( 0.43 ) | 1.52 ( -0.9 ) |
| Osteomyelitis | 1 | 0.7 ( 0.09 - 5.27 ) | 0.7 ( 0.12 ) | 0.72 ( 0.13 ) | -0.48 ( -2.64 ) |
| Muscle Abscess | 1 | 10.44 ( 0.65 - 167 ) | 10.44 ( 4.27 ) | 5.72 ( 0.56 ) | 2.52 ( -0.12 ) |
| Injection Site Scab | 1 | 0.95 ( 0.12 - 7.35 ) | 0.95 ( 0 ) | 0.95 ( 0.17 ) | -0.07 ( -2.26 ) |
| Affective Disorder | 1 | 2.61 ( 0.29 - 23.36 ) | 2.61 ( 0.8 ) | 2.29 ( 0.37 ) | 1.19 ( -1.16 ) |
| Skin Plaque | 1 | 0.58 ( 0.08 - 4.35 ) | 0.58 ( 0.29 ) | 0.6 ( 0.11 ) | -0.73 ( -2.87 ) |
| Croup Infectious | 1 | 0.29 ( 0.04 - 2.12 ) | 0.29 ( 1.69 ) | 0.31 ( 0.06 ) | -1.69 ( -3.79 ) |
| Pertussis | 1 | 0.55 ( 0.07 - 4.11 ) | 0.55 ( 0.35 ) | 0.57 ( 0.11 ) | -0.81 ( -2.94 ) |
| Scab | 1 | 0.16 ( 0.02 - 1.16 ) | 0.16 ( 4.32 ) | 0.17 ( 0.03 ) | -2.53 ( -4.6 ) |
| Affect Lability | 1 | 10.44 ( 0.65 - 167 ) | 10.44 ( 4.27 ) | 5.72 ( 0.56 ) | 2.52 ( -0.12 ) |
| Incarcerated Inguinal Hernia | 1 | 5.22 ( 0.47 - 57.6 ) | 5.22 ( 2.28 ) | 3.81 ( 0.51 ) | 1.93 ( -0.57 ) |
| Blood Magnesium Increased | 1 | 1.31 ( 0.16 - 10.44 ) | 1.31 ( 0.06 ) | 1.27 ( 0.22 ) | 0.35 ( -1.89 ) |
| Csf White Blood Cell Count Negative | 1 | 0.75 ( 0.1 - 5.67 ) | 0.75 ( 0.08 ) | 0.76 ( 0.14 ) | -0.39 ( -2.55 ) |
| Cranial Nerve Paralysis | 1 | 10.44 ( 0.65 - 167 ) | 10.44 ( 4.27 ) | 5.72 ( 0.56 ) | 2.52 ( -0.12 ) |
| Necrotising Colitis | 1 | 2.09 ( 0.24 - 17.88 ) | 2.09 ( 0.47 ) | 1.91 ( 0.32 ) | 0.93 ( -1.38 ) |
| Attention Deficit/Hyperactivity Disorder | 1 | 0.95 ( 0.12 - 7.35 ) | 0.95 ( 0 ) | 0.95 ( 0.17 ) | -0.07 ( -2.26 ) |
| Oppositional Defiant Disorder | 1 | 10.44 ( 0.65 - 167 ) | 10.44 ( 4.27 ) | 5.72 ( 0.56 ) | 2.52 ( -0.12 ) |
| Hypoperfusion | 1 | 2.61 ( 0.29 - 23.36 ) | 2.61 ( 0.8 ) | 2.29 ( 0.37 ) | 1.19 ( -1.16 ) |
| Angiogram Cerebral Abnormal | 1 | 3.48 ( 0.36 - 33.47 ) | 3.48 ( 1.33 ) | 2.86 ( 0.43 ) | 1.52 ( -0.9 ) |
| Vasogenic Cerebral Oedema | 1 | 10.44 ( 0.65 - 167 ) | 10.44 ( 4.27 ) | 5.72 ( 0.56 ) | 2.52 ( -0.12 ) |
| Hydrocephalus | 1 | 1.49 ( 0.18 - 12.13 ) | 1.49 ( 0.14 ) | 1.43 ( 0.25 ) | 0.52 ( -1.74 ) |
| Urine Odour Abnormal | 1 | 2.61 ( 0.29 - 23.36 ) | 2.61 ( 0.8 ) | 2.29 ( 0.37 ) | 1.19 ( -1.16 ) |
| Facial Paresis | 1 | 2.09 ( 0.24 - 17.88 ) | 2.09 ( 0.47 ) | 1.91 ( 0.32 ) | 0.93 ( -1.38 ) |
| Eyelid Margin Crusting | 1 | 10.44 ( 0.65 - 167 ) | 10.44 ( 4.27 ) | 5.72 ( 0.56 ) | 2.52 ( -0.12 ) |
| Nerve Injury | 1 | 2.09 ( 0.24 - 17.88 ) | 2.09 ( 0.47 ) | 1.91 ( 0.32 ) | 0.93 ( -1.38 ) |
| Social Avoidant Behaviour | 1 | 0.4 ( 0.05 - 2.96 ) | 0.4 ( 0.86 ) | 0.42 ( 0.08 ) | -1.24 ( -3.35 ) |
| Teething | 1 | 0.61 ( 0.08 - 4.62 ) | 0.61 ( 0.23 ) | 0.64 ( 0.12 ) | -0.65 ( -2.8 ) |
| Injury | 1 | 1.74 ( 0.21 - 14.46 ) | 1.74 ( 0.27 ) | 1.63 ( 0.28 ) | 0.71 ( -1.57 ) |
| Miosis | 1 | 2.09 ( 0.24 - 17.88 ) | 2.09 ( 0.47 ) | 1.91 ( 0.32 ) | 0.93 ( -1.38 ) |
| Scleral Disorder | 1 | 10.44 ( 0.65 - 167 ) | 10.44 ( 4.27 ) | 5.72 ( 0.56 ) | 2.52 ( -0.12 ) |
| Injury Associated With Device | 1 | 1.04 ( 0.13 - 8.16 ) | 1.04 ( 0 ) | 1.04 ( 0.19 ) | 0.06 ( -2.15 ) |
| Brain Stem Auditory Evoked Response Abnormal | 1 | 10.44 ( 0.65 - 167 ) | 10.44 ( 4.27 ) | 5.72 ( 0.56 ) | 2.52 ( -0.12 ) |
| Microcephaly | 1 | 3.48 ( 0.36 - 33.47 ) | 3.48 ( 1.33 ) | 2.86 ( 0.43 ) | 1.52 ( -0.9 ) |
| Psychomotor Retardation | 1 | 3.48 ( 0.36 - 33.47 ) | 3.48 ( 1.33 ) | 2.86 ( 0.43 ) | 1.52 ( -0.9 ) |
| Cognitive Disorder | 1 | 0.5 ( 0.07 - 3.7 ) | 0.5 ( 0.49 ) | 0.52 ( 0.1 ) | -0.94 ( -3.07 ) |
| Muscle Spasticity | 1 | 2.61 ( 0.29 - 23.36 ) | 2.61 ( 0.8 ) | 2.29 ( 0.37 ) | 1.19 ( -1.16 ) |
| Intellectual Disability | 1 | 0.75 ( 0.1 - 5.67 ) | 0.75 ( 0.08 ) | 0.76 ( 0.14 ) | -0.39 ( -2.55 ) |
| White Matter Lesion | 1 | 0.87 ( 0.11 - 6.69 ) | 0.87 ( 0.02 ) | 0.88 ( 0.16 ) | -0.18 ( -2.37 ) |
| Type I Hypersensitivity | 1 | 1.31 ( 0.16 - 10.44 ) | 1.31 ( 0.06 ) | 1.27 ( 0.22 ) | 0.35 ( -1.89 ) |
| Dropped Head Syndrome | 1 | 5.22 ( 0.47 - 57.6 ) | 5.22 ( 2.28 ) | 3.81 ( 0.51 ) | 1.93 ( -0.57 ) |
| Oral Mucosal Erythema | 1 | 0.95 ( 0.12 - 7.35 ) | 0.95 ( 0 ) | 0.95 ( 0.17 ) | -0.07 ( -2.26 ) |
| Catatonia | 1 | 5.22 ( 0.47 - 57.6 ) | 5.22 ( 2.28 ) | 3.81 ( 0.51 ) | 1.93 ( -0.57 ) |
| Injection Site Abscess Sterile | 1 | 1.49 ( 0.18 - 12.13 ) | 1.49 ( 0.14 ) | 1.43 ( 0.25 ) | 0.52 ( -1.74 ) |
| Enterobiasis | 1 | 10.44 ( 0.65 - 167 ) | 10.44 ( 4.27 ) | 5.72 ( 0.56 ) | 2.52 ( -0.12 ) |
| Sandifer's Syndrome | 1 | 5.22 ( 0.47 - 57.6 ) | 5.22 ( 2.28 ) | 3.81 ( 0.51 ) | 1.93 ( -0.57 ) |
| Dystonia | 1 | 1.16 ( 0.15 - 9.16 ) | 1.16 ( 0.02 ) | 1.14 ( 0.2 ) | 0.19 ( -2.02 ) |
| Vaccination Site Mass | 1 | 0.31 ( 0.04 - 2.24 ) | 0.31 ( 1.52 ) | 0.33 ( 0.06 ) | -1.61 ( -3.71 ) |
| Immunodeficiency | 1 | 0.45 ( 0.06 - 3.36 ) | 0.45 ( 0.63 ) | 0.48 ( 0.09 ) | -1.07 ( -3.19 ) |
| Sinusitis | 1 | 0.65 ( 0.09 - 4.92 ) | 0.65 ( 0.17 ) | 0.67 ( 0.12 ) | -0.57 ( -2.72 ) |
| Dermatitis Exfoliative Generalised | 1 | 5.22 ( 0.47 - 57.6 ) | 5.22 ( 2.28 ) | 3.81 ( 0.51 ) | 1.93 ( -0.57 ) |
| Arthropod Bite | 1 | 2.61 ( 0.29 - 23.36 ) | 2.61 ( 0.8 ) | 2.29 ( 0.37 ) | 1.19 ( -1.16 ) |
| Parasitic Blood Test Negative | 1 | 10.44 ( 0.65 - 167 ) | 10.44 ( 4.27 ) | 5.72 ( 0.56 ) | 2.52 ( -0.12 ) |
| Communication Disorder | 1 | 0.45 ( 0.06 - 3.36 ) | 0.45 ( 0.63 ) | 0.48 ( 0.09 ) | -1.07 ( -3.19 ) |
| Arnold-Chiari Malformation | 1 | 10.44 ( 0.65 - 167 ) | 10.44 ( 4.27 ) | 5.72 ( 0.56 ) | 2.52 ( -0.12 ) |
| Spinal Cord Disorder | 1 | 10.44 ( 0.65 - 167 ) | 10.44 ( 4.27 ) | 5.72 ( 0.56 ) | 2.52 ( -0.12 ) |
| Back Pain | 1 | 0.87 ( 0.11 - 6.69 ) | 0.87 ( 0.02 ) | 0.88 ( 0.16 ) | -0.18 ( -2.37 ) |
| Cerebral Haemorrhage | 1 | 0.8 ( 0.11 - 6.14 ) | 0.8 ( 0.04 ) | 0.82 ( 0.15 ) | -0.29 ( -2.46 ) |
| Urinary Tract Disorder | 1 | 10.44 ( 0.65 - 167 ) | 10.44 ( 4.27 ) | 5.72 ( 0.56 ) | 2.52 ( -0.12 ) |
| Herpes Virus Infection | 1 | 10.44 ( 0.65 - 167 ) | 10.44 ( 4.27 ) | 5.72 ( 0.56 ) | 2.52 ( -0.12 ) |
| Hiccups | 1 | 1.74 ( 0.21 - 14.46 ) | 1.74 ( 0.27 ) | 1.63 ( 0.28 ) | 0.71 ( -1.57 ) |
| Myoclonic Epilepsy | 1 | 1.04 ( 0.13 - 8.16 ) | 1.04 ( 0 ) | 1.04 ( 0.19 ) | 0.06 ( -2.15 ) |
| Balanoposthitis | 1 | 5.22 ( 0.47 - 57.6 ) | 5.22 ( 2.28 ) | 3.81 ( 0.51 ) | 1.93 ( -0.57 ) |
| Hypovitaminosis | 1 | 10.44 ( 0.65 - 167 ) | 10.44 ( 4.27 ) | 5.72 ( 0.56 ) | 2.52 ( -0.12 ) |
| Injection Site Dryness | 1 | 1.49 ( 0.18 - 12.13 ) | 1.49 ( 0.14 ) | 1.43 ( 0.25 ) | 0.52 ( -1.74 ) |
| Subglottic Laryngitis | 1 | 2.61 ( 0.29 - 23.36 ) | 2.61 ( 0.8 ) | 2.29 ( 0.37 ) | 1.19 ( -1.16 ) |
| Therapeutic Product Ineffective | 1 | 2.61 ( 0.29 - 23.36 ) | 2.61 ( 0.8 ) | 2.29 ( 0.37 ) | 1.19 ( -1.16 ) |
| Urticaria Chronic | 1 | 10.44 ( 0.65 - 167 ) | 10.44 ( 4.27 ) | 5.72 ( 0.56 ) | 2.52 ( -0.12 ) |
| Sluggishness | 1 | 0.42 ( 0.06 - 3.08 ) | 0.42 ( 0.78 ) | 0.44 ( 0.08 ) | -1.18 ( -3.3 ) |
| Glassy Eyes | 1 | 0.61 ( 0.08 - 4.62 ) | 0.61 ( 0.23 ) | 0.64 ( 0.12 ) | -0.65 ( -2.8 ) |
| Anal Incontinence | 1 | 2.09 ( 0.24 - 17.88 ) | 2.09 ( 0.47 ) | 1.91 ( 0.32 ) | 0.93 ( -1.38 ) |
| Pigmentation Disorder | 1 | 1.74 ( 0.21 - 14.46 ) | 1.74 ( 0.27 ) | 1.63 ( 0.28 ) | 0.71 ( -1.57 ) |
| Exercise Tolerance Decreased | 1 | 10.44 ( 0.65 - 167 ) | 10.44 ( 4.27 ) | 5.72 ( 0.56 ) | 2.52 ( -0.12 ) |
| Tourette's Disorder | 1 | 10.44 ( 0.65 - 167 ) | 10.44 ( 4.27 ) | 5.72 ( 0.56 ) | 2.52 ( -0.12 ) |
| Memory Impairment | 1 | 1.49 ( 0.18 - 12.13 ) | 1.49 ( 0.14 ) | 1.43 ( 0.25 ) | 0.52 ( -1.74 ) |
| Pulmonary Congestion | 1 | 1.31 ( 0.16 - 10.44 ) | 1.31 ( 0.06 ) | 1.27 ( 0.22 ) | 0.35 ( -1.89 ) |
| Acne | 1 | 0.4 ( 0.05 - 2.96 ) | 0.4 ( 0.86 ) | 0.42 ( 0.08 ) | -1.24 ( -3.35 ) |
| Irregular Breathing | 1 | 1.16 ( 0.15 - 9.16 ) | 1.16 ( 0.02 ) | 1.14 ( 0.2 ) | 0.19 ( -2.02 ) |
| Subdural Haematoma | 1 | 2.61 ( 0.29 - 23.36 ) | 2.61 ( 0.8 ) | 2.29 ( 0.37 ) | 1.19 ( -1.16 ) |
| Retinal Haemorrhage | 1 | 10.44 ( 0.65 - 167 ) | 10.44 ( 4.27 ) | 5.72 ( 0.56 ) | 2.52 ( -0.12 ) |
| Injection Site Pustule | 1 | 1.04 ( 0.13 - 8.16 ) | 1.04 ( 0 ) | 1.04 ( 0.19 ) | 0.06 ( -2.15 ) |
| Brain Death | 1 | 1.31 ( 0.16 - 10.44 ) | 1.31 ( 0.06 ) | 1.27 ( 0.22 ) | 0.35 ( -1.89 ) |
| Rosacea | 1 | 10.44 ( 0.65 - 167 ) | 10.44 ( 4.27 ) | 5.72 ( 0.56 ) | 2.52 ( -0.12 ) |
| Disseminated Varicella Zoster Vaccine Virus Infection | 1 | 2.61 ( 0.29 - 23.36 ) | 2.61 ( 0.8 ) | 2.29 ( 0.37 ) | 1.19 ( -1.16 ) |
| Neck Pain | 1 | 1.04 ( 0.13 - 8.16 ) | 1.04 ( 0 ) | 1.04 ( 0.19 ) | 0.06 ( -2.15 ) |
| Penile Swelling | 1 | 2.61 ( 0.29 - 23.36 ) | 2.61 ( 0.8 ) | 2.29 ( 0.37 ) | 1.19 ( -1.16 ) |
| Pyelocaliectasis | 1 | 2.61 ( 0.29 - 23.36 ) | 2.61 ( 0.8 ) | 2.29 ( 0.37 ) | 1.19 ( -1.16 ) |
| Gastric Disorder | 1 | 2.61 ( 0.29 - 23.36 ) | 2.61 ( 0.8 ) | 2.29 ( 0.37 ) | 1.19 ( -1.16 ) |
| Skin Disorder | 1 | 0.52 ( 0.07 - 3.89 ) | 0.52 ( 0.42 ) | 0.54 ( 0.1 ) | -0.88 ( -3.01 ) |
| Impetigo | 1 | 1.04 ( 0.13 - 8.16 ) | 1.04 ( 0 ) | 1.04 ( 0.19 ) | 0.06 ( -2.15 ) |
| Skin Infection | 1 | 0.8 ( 0.11 - 6.14 ) | 0.8 ( 0.04 ) | 0.82 ( 0.15 ) | -0.29 ( -2.46 ) |
| Wound Secretion | 1 | 3.48 ( 0.36 - 33.47 ) | 3.48 ( 1.33 ) | 2.86 ( 0.43 ) | 1.52 ( -0.9 ) |
| Vertigo | 1 | 5.22 ( 0.47 - 57.6 ) | 5.22 ( 2.28 ) | 3.81 ( 0.51 ) | 1.93 ( -0.57 ) |
| Mean Cell Haemoglobin Concentration Decreased | 1 | 0.65 ( 0.09 - 4.92 ) | 0.65 ( 0.17 ) | 0.67 ( 0.12 ) | -0.57 ( -2.72 ) |
| Blood Uric Acid Increased | 1 | 1.04 ( 0.13 - 8.16 ) | 1.04 ( 0 ) | 1.04 ( 0.19 ) | 0.06 ( -2.15 ) |
| Mean Platelet Volume Increased | 1 | 1.04 ( 0.13 - 8.16 ) | 1.04 ( 0 ) | 1.04 ( 0.19 ) | 0.06 ( -2.15 ) |
| Panic Reaction | 1 | 3.48 ( 0.36 - 33.47 ) | 3.48 ( 1.33 ) | 2.86 ( 0.43 ) | 1.52 ( -0.9 ) |
| Respiratory Symptom | 1 | 0.44 ( 0.06 - 3.22 ) | 0.44 ( 0.7 ) | 0.46 ( 0.09 ) | -1.13 ( -3.24 ) |
| Throat Irritation | 1 | 0.8 ( 0.11 - 6.14 ) | 0.8 ( 0.04 ) | 0.82 ( 0.15 ) | -0.29 ( -2.46 ) |
| Injection Site Haematoma | 1 | 0.65 ( 0.09 - 4.92 ) | 0.65 ( 0.17 ) | 0.67 ( 0.12 ) | -0.57 ( -2.72 ) |
| Mycoplasma Test | 1 | 0.95 ( 0.12 - 7.35 ) | 0.95 ( 0 ) | 0.95 ( 0.17 ) | -0.07 ( -2.26 ) |
| Sensory Loss | 1 | 2.61 ( 0.29 - 23.36 ) | 2.61 ( 0.8 ) | 2.29 ( 0.37 ) | 1.19 ( -1.16 ) |
| Amblyopia | 1 | 5.22 ( 0.47 - 57.6 ) | 5.22 ( 2.28 ) | 3.81 ( 0.51 ) | 1.93 ( -0.57 ) |
| Hypermetropia | 1 | 2.09 ( 0.24 - 17.88 ) | 2.09 ( 0.47 ) | 1.91 ( 0.32 ) | 0.93 ( -1.38 ) |
| Pityriasis Rosea | 1 | 10.44 ( 0.65 - 167 ) | 10.44 ( 4.27 ) | 5.72 ( 0.56 ) | 2.52 ( -0.12 ) |
| Keratosis Pilaris | 1 | 2.09 ( 0.24 - 17.88 ) | 2.09 ( 0.47 ) | 1.91 ( 0.32 ) | 0.93 ( -1.38 ) |
| Palpable Purpura | 1 | 5.22 ( 0.47 - 57.6 ) | 5.22 ( 2.28 ) | 3.81 ( 0.51 ) | 1.93 ( -0.57 ) |
| Injection Site Movement Impairment | 1 | 0.32 ( 0.04 - 2.31 ) | 0.32 ( 1.43 ) | 0.34 ( 0.06 ) | -1.57 ( -3.67 ) |
| Gastrointestinal Viral Infection | 1 | 5.22 ( 0.47 - 57.6 ) | 5.22 ( 2.28 ) | 3.81 ( 0.51 ) | 1.93 ( -0.57 ) |
| Tracheitis | 1 | 2.09 ( 0.24 - 17.88 ) | 2.09 ( 0.47 ) | 1.91 ( 0.32 ) | 0.93 ( -1.38 ) |
| Laryngitis | 1 | 0.95 ( 0.12 - 7.35 ) | 0.95 ( 0 ) | 0.95 ( 0.17 ) | -0.07 ( -2.26 ) |
| Chapped Lips | 1 | 1.16 ( 0.15 - 9.16 ) | 1.16 ( 0.02 ) | 1.14 ( 0.2 ) | 0.19 ( -2.02 ) |
| Skin Tightness | 1 | 1.31 ( 0.16 - 10.44 ) | 1.31 ( 0.06 ) | 1.27 ( 0.22 ) | 0.35 ( -1.89 ) |
| Photophobia | 1 | 0.42 ( 0.06 - 3.08 ) | 0.42 ( 0.78 ) | 0.44 ( 0.08 ) | -1.18 ( -3.3 ) |
| Lid Sulcus Deepened | 1 | 5.22 ( 0.47 - 57.6 ) | 5.22 ( 2.28 ) | 3.81 ( 0.51 ) | 1.93 ( -0.57 ) |
| Dry Mouth | 1 | 2.61 ( 0.29 - 23.36 ) | 2.61 ( 0.8 ) | 2.29 ( 0.37 ) | 1.19 ( -1.16 ) |
| Helicobacter Test Negative | 1 | 1.49 ( 0.18 - 12.13 ) | 1.49 ( 0.14 ) | 1.43 ( 0.25 ) | 0.52 ( -1.74 ) |
| Cow's Milk Intolerance | 1 | 2.09 ( 0.24 - 17.88 ) | 2.09 ( 0.47 ) | 1.91 ( 0.32 ) | 0.93 ( -1.38 ) |
| Anion Gap Increased | 1 | 10.44 ( 0.65 - 167 ) | 10.44 ( 4.27 ) | 5.72 ( 0.56 ) | 2.52 ( -0.12 ) |
| Blood Phosphorus Increased | 1 | 1.31 ( 0.16 - 10.44 ) | 1.31 ( 0.06 ) | 1.27 ( 0.22 ) | 0.35 ( -1.89 ) |
| Mean Platelet Volume Decreased | 1 | 0.8 ( 0.11 - 6.14 ) | 0.8 ( 0.04 ) | 0.82 ( 0.15 ) | -0.29 ( -2.46 ) |
| Injected Limb Mobility Decreased | 1 | 0.33 ( 0.04 - 2.39 ) | 0.33 ( 1.35 ) | 0.35 ( 0.07 ) | -1.53 ( -3.63 ) |
| Histamine Intolerance | 1 | 5.22 ( 0.47 - 57.6 ) | 5.22 ( 2.28 ) | 3.81 ( 0.51 ) | 1.93 ( -0.57 ) |
| Mastocytoma | 1 | 5.22 ( 0.47 - 57.6 ) | 5.22 ( 2.28 ) | 3.81 ( 0.51 ) | 1.93 ( -0.57 ) |
| Cardiomegaly | 1 | 1.49 ( 0.18 - 12.13 ) | 1.49 ( 0.14 ) | 1.43 ( 0.25 ) | 0.52 ( -1.74 ) |
| End-Tidal Co2 Decreased | 1 | 10.44 ( 0.65 - 167 ) | 10.44 ( 4.27 ) | 5.72 ( 0.56 ) | 2.52 ( -0.12 ) |
| Ophthalmological Examination Abnormal | 1 | 2.09 ( 0.24 - 17.88 ) | 2.09 ( 0.47 ) | 1.91 ( 0.32 ) | 0.93 ( -1.38 ) |
| Gastrointestinal Motility Disorder | 1 | 3.48 ( 0.36 - 33.47 ) | 3.48 ( 1.33 ) | 2.86 ( 0.43 ) | 1.52 ( -0.9 ) |
| Gene Mutation Identification Test Positive | 1 | 0.61 ( 0.08 - 4.62 ) | 0.61 ( 0.23 ) | 0.64 ( 0.12 ) | -0.65 ( -2.8 ) |
| Dermatitis Allergic | 1 | 0.24 ( 0.03 - 1.76 ) | 0.24 ( 2.31 ) | 0.26 ( 0.05 ) | -1.94 ( -4.03 ) |
| Dysbiosis | 1 | 1.16 ( 0.15 - 9.16 ) | 1.16 ( 0.02 ) | 1.14 ( 0.2 ) | 0.19 ( -2.02 ) |
| Juvenile Idiopathic Arthritis | 1 | 0.95 ( 0.12 - 7.35 ) | 0.95 ( 0 ) | 0.95 ( 0.17 ) | -0.07 ( -2.26 ) |
| Injection Site Joint Swelling | 1 | 5.22 ( 0.47 - 57.6 ) | 5.22 ( 2.28 ) | 3.81 ( 0.51 ) | 1.93 ( -0.57 ) |
| Csf Cell Count Increased | 1 | 0.75 ( 0.1 - 5.67 ) | 0.75 ( 0.08 ) | 0.76 ( 0.14 ) | -0.39 ( -2.55 ) |
| Csf White Blood Cell Count Increased | 1 | 0.8 ( 0.11 - 6.14 ) | 0.8 ( 0.04 ) | 0.82 ( 0.15 ) | -0.29 ( -2.46 ) |
| Meningitis Aseptic | 1 | 1.16 ( 0.15 - 9.16 ) | 1.16 ( 0.02 ) | 1.14 ( 0.2 ) | 0.19 ( -2.02 ) |
| Red Blood Cell Nucleated Morphology Present | 1 | 10.44 ( 0.65 - 167 ) | 10.44 ( 4.27 ) | 5.72 ( 0.56 ) | 2.52 ( -0.12 ) |
| Basophil Count Increased | 1 | 1.31 ( 0.16 - 10.44 ) | 1.31 ( 0.06 ) | 1.27 ( 0.22 ) | 0.35 ( -1.89 ) |
| Csf Glucose Decreased | 1 | 0.87 ( 0.11 - 6.69 ) | 0.87 ( 0.02 ) | 0.88 ( 0.16 ) | -0.18 ( -2.37 ) |
| Basophil Percentage Increased | 1 | 1.16 ( 0.15 - 9.16 ) | 1.16 ( 0.02 ) | 1.14 ( 0.2 ) | 0.19 ( -2.02 ) |
| Encephalomalacia | 1 | 10.44 ( 0.65 - 167 ) | 10.44 ( 4.27 ) | 5.72 ( 0.56 ) | 2.52 ( -0.12 ) |
| Transaminases Increased | 1 | 1.31 ( 0.16 - 10.44 ) | 1.31 ( 0.06 ) | 1.27 ( 0.22 ) | 0.35 ( -1.89 ) |
| Hypoalbuminaemia | 1 | 3.48 ( 0.36 - 33.47 ) | 3.48 ( 1.33 ) | 2.86 ( 0.43 ) | 1.52 ( -0.9 ) |
| Vision Blurred | 1 | 0.55 ( 0.07 - 4.11 ) | 0.55 ( 0.35 ) | 0.57 ( 0.11 ) | -0.81 ( -2.94 ) |
| Dysphagia | 1 | 0.26 ( 0.04 - 1.9 ) | 0.26 ( 2.04 ) | 0.28 ( 0.05 ) | -1.84 ( -3.93 ) |
| Skin Fissures | 1 | 1.31 ( 0.16 - 10.44 ) | 1.31 ( 0.06 ) | 1.27 ( 0.22 ) | 0.35 ( -1.89 ) |
| Neurological Decompensation | 1 | 3.48 ( 0.36 - 33.47 ) | 3.48 ( 1.33 ) | 2.86 ( 0.43 ) | 1.52 ( -0.9 ) |
| Genital Rash | 1 | 0.8 ( 0.11 - 6.14 ) | 0.8 ( 0.04 ) | 0.82 ( 0.15 ) | -0.29 ( -2.46 ) |
| Viral Test Positive | 1 | 0.58 ( 0.08 - 4.35 ) | 0.58 ( 0.29 ) | 0.6 ( 0.11 ) | -0.73 ( -2.87 ) |
| Csf Pressure Increased | 1 | 10.44 ( 0.65 - 167 ) | 10.44 ( 4.27 ) | 5.72 ( 0.56 ) | 2.52 ( -0.12 ) |
| Gross Motor Delay | 1 | 0.87 ( 0.11 - 6.69 ) | 0.87 ( 0.02 ) | 0.88 ( 0.16 ) | -0.18 ( -2.37 ) |
| Subdural Hygroma | 1 | 5.22 ( 0.47 - 57.6 ) | 5.22 ( 2.28 ) | 3.81 ( 0.51 ) | 1.93 ( -0.57 ) |
| Vaccination Site Inflammation | 1 | 0.58 ( 0.08 - 4.35 ) | 0.58 ( 0.29 ) | 0.6 ( 0.11 ) | -0.73 ( -2.87 ) |
| Malaria | 1 | 5.22 ( 0.47 - 57.6 ) | 5.22 ( 2.28 ) | 3.81 ( 0.51 ) | 1.93 ( -0.57 ) |
| Injection Site Indentation | 1 | 3.48 ( 0.36 - 33.47 ) | 3.48 ( 1.33 ) | 2.86 ( 0.43 ) | 1.52 ( -0.9 ) |
| White Blood Cells Urine Positive | 1 | 0.4 ( 0.05 - 2.96 ) | 0.4 ( 0.86 ) | 0.42 ( 0.08 ) | -1.24 ( -3.35 ) |
| Pathology Test | 1 | 0.7 ( 0.09 - 5.27 ) | 0.7 ( 0.12 ) | 0.72 ( 0.13 ) | -0.48 ( -2.64 ) |
| Anisocytosis | 1 | 3.48 ( 0.36 - 33.47 ) | 3.48 ( 1.33 ) | 2.86 ( 0.43 ) | 1.52 ( -0.9 ) |
| Joint Effusion | 1 | 1.74 ( 0.21 - 14.46 ) | 1.74 ( 0.27 ) | 1.63 ( 0.28 ) | 0.71 ( -1.57 ) |
| Lymphocytosis | 1 | 1.31 ( 0.16 - 10.44 ) | 1.31 ( 0.06 ) | 1.27 ( 0.22 ) | 0.35 ( -1.89 ) |
| X-Ray Of Pelvis And Hip Abnormal | 1 | 10.44 ( 0.65 - 167 ) | 10.44 ( 4.27 ) | 5.72 ( 0.56 ) | 2.52 ( -0.12 ) |
| Oropharyngeal Plaque | 1 | 10.44 ( 0.65 - 167 ) | 10.44 ( 4.27 ) | 5.72 ( 0.56 ) | 2.52 ( -0.12 ) |
| Tonsillar Exudate | 1 | 5.22 ( 0.47 - 57.6 ) | 5.22 ( 2.28 ) | 3.81 ( 0.51 ) | 1.93 ( -0.57 ) |
| Culture Negative | 1 | 0.36 ( 0.05 - 2.64 ) | 0.36 ( 1.1 ) | 0.38 ( 0.07 ) | -1.39 ( -3.5 ) |
| Red Blood Cell Anisocytes Present | 1 | 10.44 ( 0.65 - 167 ) | 10.44 ( 4.27 ) | 5.72 ( 0.56 ) | 2.52 ( -0.12 ) |
| Impaired Healing | 1 | 5.22 ( 0.47 - 57.6 ) | 5.22 ( 2.28 ) | 3.81 ( 0.51 ) | 1.93 ( -0.57 ) |
| Skin Erosion | 1 | 1.49 ( 0.18 - 12.13 ) | 1.49 ( 0.14 ) | 1.43 ( 0.25 ) | 0.52 ( -1.74 ) |
| Bronchopulmonary Dysplasia | 1 | 10.44 ( 0.65 - 167 ) | 10.44 ( 4.27 ) | 5.72 ( 0.56 ) | 2.52 ( -0.12 ) |
| Feeling Cold | 1 | 0.55 ( 0.07 - 4.11 ) | 0.55 ( 0.35 ) | 0.57 ( 0.11 ) | -0.81 ( -2.94 ) |
| Amino Acid Level Increased | 1 | 5.22 ( 0.47 - 57.6 ) | 5.22 ( 2.28 ) | 3.81 ( 0.51 ) | 1.93 ( -0.57 ) |
| Vitamin B12 Decreased | 1 | 10.44 ( 0.65 - 167 ) | 10.44 ( 4.27 ) | 5.72 ( 0.56 ) | 2.52 ( -0.12 ) |
| Rash Pustular | 1 | 0.34 ( 0.05 - 2.47 ) | 0.34 ( 1.26 ) | 0.36 ( 0.07 ) | -1.48 ( -3.58 ) |
| Skin Burning Sensation | 1 | 2.61 ( 0.29 - 23.36 ) | 2.61 ( 0.8 ) | 2.29 ( 0.37 ) | 1.19 ( -1.16 ) |
| Tardive Dyskinesia | 1 | 0.95 ( 0.12 - 7.35 ) | 0.95 ( 0 ) | 0.95 ( 0.17 ) | -0.07 ( -2.26 ) |
| Protein Urine Present | 1 | 1.49 ( 0.18 - 12.13 ) | 1.49 ( 0.14 ) | 1.43 ( 0.25 ) | 0.52 ( -1.74 ) |
| Leukocyturia | 1 | 1.74 ( 0.21 - 14.46 ) | 1.74 ( 0.27 ) | 1.63 ( 0.28 ) | 0.71 ( -1.57 ) |
| Urosepsis | 1 | 2.61 ( 0.29 - 23.36 ) | 2.61 ( 0.8 ) | 2.29 ( 0.37 ) | 1.19 ( -1.16 ) |
| Mouth Ulceration | 1 | 0.95 ( 0.12 - 7.35 ) | 0.95 ( 0 ) | 0.95 ( 0.17 ) | -0.07 ( -2.26 ) |
| Intestinal Obstruction | 1 | 0.32 ( 0.04 - 2.31 ) | 0.32 ( 1.43 ) | 0.34 ( 0.06 ) | -1.57 ( -3.67 ) |
| Central Nervous System Lesion | 1 | 0.87 ( 0.11 - 6.69 ) | 0.87 ( 0.02 ) | 0.88 ( 0.16 ) | -0.18 ( -2.37 ) |
| Pustule | 1 | 0.45 ( 0.06 - 3.36 ) | 0.45 ( 0.63 ) | 0.48 ( 0.09 ) | -1.07 ( -3.19 ) |
| Measles Antibody Negative | 1 | 0.75 ( 0.1 - 5.67 ) | 0.75 ( 0.08 ) | 0.76 ( 0.14 ) | -0.39 ( -2.55 ) |
| Gastrointestinal Haemorrhage | 1 | 0.87 ( 0.11 - 6.69 ) | 0.87 ( 0.02 ) | 0.88 ( 0.16 ) | -0.18 ( -2.37 ) |
| Limb Discomfort | 1 | 0.65 ( 0.09 - 4.92 ) | 0.65 ( 0.17 ) | 0.67 ( 0.12 ) | -0.57 ( -2.72 ) |
| Sensory Disturbance | 1 | 0.75 ( 0.1 - 5.67 ) | 0.75 ( 0.08 ) | 0.76 ( 0.14 ) | -0.39 ( -2.55 ) |
| Petit Mal Epilepsy | 1 | 0.3 ( 0.04 - 2.18 ) | 0.3 ( 1.6 ) | 0.32 ( 0.06 ) | -1.65 ( -3.75 ) |
| Acute Lymphocytic Leukaemia | 1 | 1.49 ( 0.18 - 12.13 ) | 1.49 ( 0.14 ) | 1.43 ( 0.25 ) | 0.52 ( -1.74 ) |
| Fear | 1 | 0.58 ( 0.08 - 4.35 ) | 0.58 ( 0.29 ) | 0.6 ( 0.11 ) | -0.73 ( -2.87 ) |
| Shock | 1 | 0.35 ( 0.05 - 2.55 ) | 0.35 ( 1.18 ) | 0.37 ( 0.07 ) | -1.44 ( -3.54 ) |
| Blood Smear Test Abnormal | 1 | 1.31 ( 0.16 - 10.44 ) | 1.31 ( 0.06 ) | 1.27 ( 0.22 ) | 0.35 ( -1.89 ) |
| Haemolytic Anaemia | 1 | 2.61 ( 0.29 - 23.36 ) | 2.61 ( 0.8 ) | 2.29 ( 0.37 ) | 1.19 ( -1.16 ) |
| Transient Hypogammaglobulinaemia Of Infancy | 1 | 10.44 ( 0.65 - 167 ) | 10.44 ( 4.27 ) | 5.72 ( 0.56 ) | 2.52 ( -0.12 ) |
| Liver Function Test Abnormal | 1 | 2.61 ( 0.29 - 23.36 ) | 2.61 ( 0.8 ) | 2.29 ( 0.37 ) | 1.19 ( -1.16 ) |
| Hepatic Enzyme Abnormal | 1 | 5.22 ( 0.47 - 57.6 ) | 5.22 ( 2.28 ) | 3.81 ( 0.51 ) | 1.93 ( -0.57 ) |
| Central Nervous System Inflammation | 1 | 5.22 ( 0.47 - 57.6 ) | 5.22 ( 2.28 ) | 3.81 ( 0.51 ) | 1.93 ( -0.57 ) |
| Magnetic Resonance Imaging Spinal Abnormal | 1 | 1.16 ( 0.15 - 9.16 ) | 1.16 ( 0.02 ) | 1.14 ( 0.2 ) | 0.19 ( -2.02 ) |
| Angioedema | 1 | 0.12 ( 0.02 - 0.89 ) | 0.12 ( 6.1 ) | 0.13 ( 0.03 ) | -2.89 ( -4.96 ) |
| Hemiplegia | 1 | 1.31 ( 0.16 - 10.44 ) | 1.31 ( 0.06 ) | 1.27 ( 0.22 ) | 0.35 ( -1.89 ) |
| Hypoxic-Ischaemic Encephalopathy | 1 | 1.31 ( 0.16 - 10.44 ) | 1.31 ( 0.06 ) | 1.27 ( 0.22 ) | 0.35 ( -1.89 ) |
| Pneumonia Aspiration | 1 | 3.48 ( 0.36 - 33.47 ) | 3.48 ( 1.33 ) | 2.86 ( 0.43 ) | 1.52 ( -0.9 ) |
| Hypotension | 1 | 0.45 ( 0.06 - 3.36 ) | 0.45 ( 0.63 ) | 0.48 ( 0.09 ) | -1.07 ( -3.19 ) |
| Cardiac Monitoring Abnormal | 1 | 3.48 ( 0.36 - 33.47 ) | 3.48 ( 1.33 ) | 2.86 ( 0.43 ) | 1.52 ( -0.9 ) |
| Ventricular Extrasystoles | 1 | 2.61 ( 0.29 - 23.36 ) | 2.61 ( 0.8 ) | 2.29 ( 0.37 ) | 1.19 ( -1.16 ) |
| Narcolepsy | 1 | 5.22 ( 0.47 - 57.6 ) | 5.22 ( 2.28 ) | 3.81 ( 0.51 ) | 1.93 ( -0.57 ) |
| X-Ray Gastrointestinal Tract Abnormal | 1 | 0.87 ( 0.11 - 6.69 ) | 0.87 ( 0.02 ) | 0.88 ( 0.16 ) | -0.18 ( -2.37 ) |
| Faeces Hard | 1 | 2.61 ( 0.29 - 23.36 ) | 2.61 ( 0.8 ) | 2.29 ( 0.37 ) | 1.19 ( -1.16 ) |
| Meningitis Bacterial | 1 | 0.58 ( 0.08 - 4.35 ) | 0.58 ( 0.29 ) | 0.6 ( 0.11 ) | -0.73 ( -2.87 ) |
