# Supplementary material for: Comparative safety evaluation of pentavalent (DTaP-IPV-Hib) and hexavalent (DTaP-IPV-Hib-HepB) vaccines in infants: a real-world analysis based on VAERS
Source: Front Cell Infect Microbiol. 2025 Oct 30;15:1666509. doi: 10.3389/fcimb.2025.1666509 (PMC12611864; doi:10.3389/fcimb.2025.1666509)
Supplement: Supplementary file 7 [file Table5.docx]

Table 5: PT-level distribution and signal strength of reported AEFIs in the hexavalent vaccine group.

| PT | N | ROR(95%Cl) | PRR(X^2^) | EBGM(EBGM05) | IC(IC025) |
| --- | --- | --- | --- | --- | --- |
| Pyrexia | 388 | 1.02 ( 0.92 - 1.14 ) | 1.02 ( 0.16 ) | 1.02 ( 0.93 ) | 0.03 ( -0.13 ) |
| Pallor | 146 | 3.73 ( 3.12 - 4.47 ) | 3.68 ( 237.22 ) | 3.22 ( 2.77 ) | 1.69 ( 1.43 ) |
| Vomiting | 119 | 1.06 ( 0.88 - 1.28 ) | 1.06 ( 0.4 ) | 1.06 ( 0.9 ) | 0.08 ( -0.19 ) |
| Diarrhoea | 102 | 1.18 ( 0.97 - 1.45 ) | 1.18 ( 2.71 ) | 1.17 ( 0.99 ) | 0.23 ( -0.07 ) |
| Hypotonia | 95 | 2.96 ( 2.38 - 3.69 ) | 2.94 ( 104.68 ) | 2.66 ( 2.22 ) | 1.41 ( 1.1 ) |
| Seizure | 95 | 1.52 ( 1.23 - 1.88 ) | 1.51 ( 15.38 ) | 1.47 ( 1.23 ) | 0.56 ( 0.25 ) |
| Rash | 94 | 0.61 ( 0.5 - 0.75 ) | 0.62 ( 22.19 ) | 0.63 ( 0.53 ) | -0.67 ( -0.97 ) |
| Cyanosis | 84 | 2.93 ( 2.33 - 3.7 ) | 2.91 ( 90.95 ) | 2.64 ( 2.18 ) | 1.4 ( 1.07 ) |
| Hypotonic-Hyporesponsive Episode | 79 | 3.73 ( 2.92 - 4.75 ) | 3.7 ( 129.05 ) | 3.23 ( 2.64 ) | 1.69 ( 1.34 ) |
| Febrile Convulsion | 66 | 1.11 ( 0.86 - 1.42 ) | 1.11 ( 0.65 ) | 1.1 ( 0.89 ) | 0.14 ( -0.23 ) |
| Somnolence | 65 | 2.07 ( 1.6 - 2.68 ) | 2.06 ( 31.91 ) | 1.95 ( 1.57 ) | 0.96 ( 0.59 ) |
| Erythema | 62 | 1.1 ( 0.85 - 1.43 ) | 1.1 ( 0.55 ) | 1.1 ( 0.88 ) | 0.13 ( -0.24 ) |
| Injection Site Erythema | 62 | 0.64 ( 0.49 - 0.82 ) | 0.64 ( 12.15 ) | 0.65 ( 0.53 ) | -0.61 ( -0.98 ) |
| Irritability | 56 | 0.67 ( 0.51 - 0.87 ) | 0.67 ( 9 ) | 0.68 ( 0.54 ) | -0.56 ( -0.95 ) |
| Unresponsive To Stimuli | 53 | 2.17 ( 1.63 - 2.89 ) | 2.16 ( 29.58 ) | 2.04 ( 1.6 ) | 1.03 ( 0.61 ) |
| Injection Site Swelling | 53 | 0.81 ( 0.61 - 1.06 ) | 0.81 ( 2.31 ) | 0.82 ( 0.65 ) | -0.29 ( -0.69 ) |
| Decreased Appetite | 51 | 0.9 ( 0.68 - 1.2 ) | 0.9 ( 0.49 ) | 0.91 ( 0.72 ) | -0.14 ( -0.55 ) |
| Apnoea | 46 | 3.68 ( 2.68 - 5.07 ) | 3.67 ( 74.12 ) | 3.21 ( 2.46 ) | 1.68 ( 1.23 ) |
| Urticaria | 46 | 0.71 ( 0.53 - 0.96 ) | 0.71 ( 5.13 ) | 0.72 ( 0.57 ) | -0.46 ( -0.89 ) |
| Body Temperature Increased | 43 | 1.54 ( 1.13 - 2.1 ) | 1.53 ( 7.41 ) | 1.49 ( 1.15 ) | 0.58 ( 0.12 ) |
| Loss Of Consciousness | 40 | 2.43 ( 1.74 - 3.38 ) | 2.42 ( 29.37 ) | 2.25 ( 1.7 ) | 1.17 ( 0.69 ) |
| Haematochezia | 39 | 1.09 ( 0.79 - 1.51 ) | 1.09 ( 0.3 ) | 1.09 ( 0.83 ) | 0.12 ( -0.35 ) |
| Restlessness | 38 | 2.19 ( 1.56 - 3.07 ) | 2.19 ( 21.83 ) | 2.06 ( 1.55 ) | 1.04 ( 0.55 ) |
| Dyspnoea | 38 | 1.73 ( 1.24 - 2.42 ) | 1.73 ( 10.65 ) | 1.66 ( 1.26 ) | 0.73 ( 0.25 ) |
| Hyperpyrexia | 37 | 1.62 ( 1.16 - 2.27 ) | 1.62 ( 8.07 ) | 1.57 ( 1.18 ) | 0.65 ( 0.16 ) |
| Death | 34 | 1.36 ( 0.96 - 1.93 ) | 1.36 ( 3.04 ) | 1.34 ( 1 ) | 0.42 ( -0.09 ) |
| Intussusception | 34 | 1.08 ( 0.77 - 1.53 ) | 1.08 ( 0.21 ) | 1.08 ( 0.81 ) | 0.11 ( -0.39 ) |
| Tremor | 33 | 1.53 ( 1.07 - 2.19 ) | 1.53 ( 5.62 ) | 1.49 ( 1.11 ) | 0.57 ( 0.06 ) |
| Pain | 33 | 1.5 ( 1.05 - 2.13 ) | 1.49 ( 4.97 ) | 1.45 ( 1.08 ) | 0.54 ( 0.03 ) |
| Infant Irritability | 32 | 3.46 ( 2.37 - 5.05 ) | 3.45 ( 46.64 ) | 3.05 ( 2.22 ) | 1.61 ( 1.07 ) |
| C-Reactive Protein Increased | 32 | 1.88 ( 1.31 - 2.71 ) | 1.88 ( 11.9 ) | 1.79 ( 1.32 ) | 0.84 ( 0.32 ) |
| Abdominal Pain | 29 | 2.81 ( 1.9 - 4.16 ) | 2.8 ( 29.07 ) | 2.56 ( 1.84 ) | 1.35 ( 0.79 ) |
| Lethargy | 27 | 0.71 ( 0.48 - 1.04 ) | 0.71 ( 3.12 ) | 0.72 ( 0.52 ) | -0.47 ( -1.03 ) |
| Peripheral Swelling | 27 | 2.04 ( 1.37 - 3.04 ) | 2.03 ( 12.76 ) | 1.93 ( 1.38 ) | 0.95 ( 0.37 ) |
| Dyskinesia | 26 | 2.03 ( 1.35 - 3.05 ) | 2.03 ( 12.17 ) | 1.92 ( 1.37 ) | 0.94 ( 0.36 ) |
| Musculoskeletal Stiffness | 26 | 2.55 ( 1.69 - 3.84 ) | 2.54 ( 21.27 ) | 2.35 ( 1.66 ) | 1.23 ( 0.64 ) |
| Fatigue | 26 | 0.94 ( 0.64 - 1.4 ) | 0.95 ( 0.08 ) | 0.95 ( 0.68 ) | -0.08 ( -0.65 ) |
| Malaise | 25 | 1.43 ( 0.95 - 2.15 ) | 1.43 ( 2.99 ) | 1.4 ( 0.99 ) | 0.48 ( -0.11 ) |
| Muscle Spasms | 24 | 4.36 ( 2.79 - 6.83 ) | 4.35 ( 49.85 ) | 3.69 ( 2.54 ) | 1.89 ( 1.25 ) |
| Cough | 24 | 0.63 ( 0.42 - 0.94 ) | 0.63 ( 5.09 ) | 0.64 ( 0.46 ) | -0.64 ( -1.23 ) |
| Apathy | 24 | 4.04 ( 2.59 - 6.29 ) | 4.02 ( 44.53 ) | 3.47 ( 2.39 ) | 1.79 ( 1.16 ) |
| Eye Movement Disorder | 23 | 1.57 ( 1.02 - 2.4 ) | 1.57 ( 4.34 ) | 1.52 ( 1.06 ) | 0.6 ( -0.01 ) |
| Depressed Level Of Consciousness | 22 | 5.3 ( 3.29 - 8.53 ) | 5.28 ( 58.96 ) | 4.3 ( 2.89 ) | 2.11 ( 1.44 ) |
| Skin Discolouration | 22 | 2.03 ( 1.3 - 3.16 ) | 2.03 ( 10.28 ) | 1.92 ( 1.33 ) | 0.94 ( 0.31 ) |
| Sleep Disorder | 21 | 1.89 ( 1.2 - 2.96 ) | 1.89 ( 7.91 ) | 1.8 ( 1.24 ) | 0.85 ( 0.2 ) |
| Petechiae | 21 | 1.68 ( 1.07 - 2.62 ) | 1.67 ( 5.22 ) | 1.62 ( 1.11 ) | 0.69 ( 0.05 ) |
| Rash Erythematous | 20 | 0.55 ( 0.35 - 0.86 ) | 0.55 ( 7.05 ) | 0.57 ( 0.39 ) | -0.82 ( -1.46 ) |
| Dysentery | 20 | 13.2 ( 7.4 - 23.55 ) | 13.17 ( 129.23 ) | 7.99 ( 4.92 ) | 3 ( 2.24 ) |
| Poor Feeding Infant | 19 | 2.64 ( 1.63 - 4.28 ) | 2.64 ( 16.86 ) | 2.43 ( 1.62 ) | 1.28 ( 0.59 ) |
| Gaze Palsy | 19 | 2.38 ( 1.48 - 3.85 ) | 2.38 ( 13.41 ) | 2.22 ( 1.48 ) | 1.15 ( 0.46 ) |
| Generalised Tonic-Clonic Seizure | 19 | 2.51 ( 1.55 - 4.05 ) | 2.5 ( 15.03 ) | 2.32 ( 1.55 ) | 1.21 ( 0.53 ) |
| Injection Site Reaction | 19 | 0.93 ( 0.58 - 1.47 ) | 0.93 ( 0.11 ) | 0.93 ( 0.63 ) | -0.11 ( -0.77 ) |
| Listless | 18 | 5.73 ( 3.36 - 9.74 ) | 5.71 ( 53 ) | 4.57 ( 2.93 ) | 2.19 ( 1.45 ) |
| Bradycardia | 18 | 7.13 ( 4.12 - 12.32 ) | 7.11 ( 67.55 ) | 5.36 ( 3.39 ) | 2.42 ( 1.67 ) |
| White Blood Cell Count Increased | 18 | 1.18 ( 0.73 - 1.9 ) | 1.18 ( 0.45 ) | 1.17 ( 0.78 ) | 0.22 ( -0.46 ) |
| Constipation | 17 | 2.31 ( 1.39 - 3.83 ) | 2.31 ( 11.15 ) | 2.16 ( 1.41 ) | 1.11 ( 0.39 ) |
| Anaphylactic Reaction | 17 | 2.88 ( 1.73 - 4.81 ) | 2.88 ( 17.95 ) | 2.62 ( 1.7 ) | 1.39 ( 0.66 ) |
| Injection Site Pain | 17 | 0.57 ( 0.35 - 0.92 ) | 0.57 ( 5.4 ) | 0.58 ( 0.39 ) | -0.78 ( -1.47 ) |
| Vomiting Projectile | 17 | 2.8 ( 1.68 - 4.68 ) | 2.8 ( 16.99 ) | 2.55 ( 1.66 ) | 1.35 ( 0.62 ) |
| Asthenia | 16 | 1.09 ( 0.66 - 1.8 ) | 1.09 ( 0.1 ) | 1.08 ( 0.71 ) | 0.11 ( -0.61 ) |
| Oxygen Saturation Decreased | 16 | 3.75 ( 2.19 - 6.43 ) | 3.74 ( 26.58 ) | 3.27 ( 2.08 ) | 1.71 ( 0.95 ) |
| Injection Site Mass | 16 | 1.37 ( 0.83 - 2.29 ) | 1.37 ( 1.51 ) | 1.35 ( 0.88 ) | 0.43 ( -0.3 ) |
| General Physical Health Deterioration | 15 | 5.14 ( 2.89 - 9.13 ) | 5.13 ( 38.7 ) | 4.2 ( 2.6 ) | 2.07 ( 1.27 ) |
| Epilepsy | 15 | 2.15 ( 1.26 - 3.68 ) | 2.15 ( 8.24 ) | 2.03 ( 1.29 ) | 1.02 ( 0.26 ) |
| Swelling | 15 | 0.66 ( 0.39 - 1.1 ) | 0.66 ( 2.6 ) | 0.67 ( 0.43 ) | -0.58 ( -1.32 ) |
| Respiratory Arrest | 15 | 1.72 ( 1.01 - 2.93 ) | 1.72 ( 4.13 ) | 1.66 ( 1.06 ) | 0.73 ( -0.03 ) |
| Condition Aggravated | 14 | 1.12 ( 0.65 - 1.93 ) | 1.12 ( 0.17 ) | 1.11 ( 0.71 ) | 0.16 ( -0.61 ) |
| Discomfort | 14 | 1.36 ( 0.79 - 2.34 ) | 1.36 ( 1.24 ) | 1.33 ( 0.85 ) | 0.42 ( -0.36 ) |
| Infantile Apnoea | 14 | 8.9 ( 4.69 - 16.92 ) | 8.89 ( 65.36 ) | 6.26 ( 3.66 ) | 2.65 ( 1.78 ) |
| Hypertonia | 14 | 2.93 ( 1.66 - 5.16 ) | 2.93 ( 15.27 ) | 2.66 ( 1.65 ) | 1.41 ( 0.61 ) |
| Blood Culture Negative | 14 | 1.59 ( 0.92 - 2.74 ) | 1.59 ( 2.78 ) | 1.54 ( 0.97 ) | 0.62 ( -0.16 ) |
| Ultrasound Abdomen Abnormal | 14 | 1.06 ( 0.62 - 1.82 ) | 1.06 ( 0.04 ) | 1.06 ( 0.67 ) | 0.08 ( -0.69 ) |
| Rash Macular | 14 | 0.77 ( 0.45 - 1.31 ) | 0.77 ( 0.94 ) | 0.78 ( 0.5 ) | -0.36 ( -1.12 ) |
| Floppy Infant | 13 | 12.18 ( 6.01 - 24.68 ) | 12.16 ( 79.1 ) | 7.63 ( 4.23 ) | 2.93 ( 2.01 ) |
| Mucous Stools | 13 | 1.42 ( 0.81 - 2.5 ) | 1.42 ( 1.48 ) | 1.39 ( 0.86 ) | 0.47 ( -0.33 ) |
| Livedo Reticularis | 13 | 6.09 ( 3.24 - 11.44 ) | 6.08 ( 41.14 ) | 4.79 ( 2.82 ) | 2.26 ( 1.39 ) |
| Eczema | 13 | 1.2 ( 0.69 - 2.11 ) | 1.2 ( 0.42 ) | 1.19 ( 0.74 ) | 0.25 ( -0.55 ) |
| Hypersensitivity | 13 | 0.65 ( 0.38 - 1.14 ) | 0.65 ( 2.29 ) | 0.67 ( 0.42 ) | -0.58 ( -1.37 ) |
| Abdominal Pain Upper | 13 | 3.86 ( 2.12 - 7.03 ) | 3.85 ( 22.57 ) | 3.34 ( 2.02 ) | 1.74 ( 0.9 ) |
| Sars-Cov-2 Test Negative | 13 | 0.92 ( 0.53 - 1.61 ) | 0.92 ( 0.08 ) | 0.93 ( 0.58 ) | -0.11 ( -0.9 ) |
| Infantile Spasms | 13 | 4.37 ( 2.38 - 8.01 ) | 4.36 ( 27.05 ) | 3.7 ( 2.23 ) | 1.89 ( 1.04 ) |
| Vaccination Site Erythema | 13 | 1.38 ( 0.78 - 2.42 ) | 1.38 ( 1.24 ) | 1.35 ( 0.84 ) | 0.43 ( -0.37 ) |
| Rotavirus Test Positive | 13 | 0.74 ( 0.42 - 1.29 ) | 0.74 ( 1.16 ) | 0.75 ( 0.47 ) | -0.42 ( -1.21 ) |
| Abnormal Behaviour | 13 | 0.89 ( 0.51 - 1.56 ) | 0.89 ( 0.16 ) | 0.9 ( 0.56 ) | -0.16 ( -0.95 ) |
| Dehydration | 13 | 1.32 ( 0.75 - 2.32 ) | 1.32 ( 0.94 ) | 1.3 ( 0.81 ) | 0.38 ( -0.42 ) |
| Injection Site Induration | 13 | 0.54 ( 0.31 - 0.95 ) | 0.55 ( 4.81 ) | 0.56 ( 0.35 ) | -0.84 ( -1.62 ) |
| Injection Site Nodule | 13 | 1.52 ( 0.86 - 2.68 ) | 1.52 ( 2.14 ) | 1.48 ( 0.92 ) | 0.56 ( -0.24 ) |
| Injection Site Warmth | 13 | 0.48 ( 0.27 - 0.83 ) | 0.48 ( 7.27 ) | 0.49 ( 0.31 ) | -1.03 ( -1.81 ) |
| Faeces Discoloured | 13 | 1.55 ( 0.88 - 2.74 ) | 1.55 ( 2.34 ) | 1.51 ( 0.94 ) | 0.59 ( -0.21 ) |
| Skin Warm | 13 | 0.82 ( 0.47 - 1.43 ) | 0.82 ( 0.5 ) | 0.83 ( 0.52 ) | -0.27 ( -1.06 ) |
| Vaccination Site Swelling | 12 | 1.29 ( 0.72 - 2.31 ) | 1.29 ( 0.71 ) | 1.27 ( 0.77 ) | 0.34 ( -0.49 ) |
| Status Epilepticus | 12 | 3.34 ( 1.8 - 6.19 ) | 3.33 ( 16.51 ) | 2.96 ( 1.77 ) | 1.57 ( 0.7 ) |
| Agitation | 12 | 2.37 ( 1.3 - 4.34 ) | 2.37 ( 8.39 ) | 2.21 ( 1.33 ) | 1.14 ( 0.29 ) |
| Flatulence | 12 | 3.05 ( 1.65 - 5.63 ) | 3.05 ( 14.1 ) | 2.75 ( 1.65 ) | 1.46 ( 0.6 ) |
| Syncope | 12 | 1.52 ( 0.85 - 2.75 ) | 1.52 ( 1.99 ) | 1.48 ( 0.9 ) | 0.57 ( -0.27 ) |
| Electroencephalogram Abnormal | 12 | 2.01 ( 1.11 - 3.66 ) | 2.01 ( 5.5 ) | 1.91 ( 1.16 ) | 0.93 ( 0.09 ) |
| Anxiety | 12 | 1.69 ( 0.94 - 3.06 ) | 1.69 ( 3.11 ) | 1.63 ( 0.99 ) | 0.71 ( -0.13 ) |
| Staring | 12 | 1.34 ( 0.75 - 2.41 ) | 1.34 ( 0.97 ) | 1.32 ( 0.81 ) | 0.4 ( -0.43 ) |
| Tachycardia | 12 | 1.98 ( 1.09 - 3.59 ) | 1.98 ( 5.2 ) | 1.88 ( 1.14 ) | 0.91 ( 0.07 ) |
| Hypophagia | 12 | 1.62 ( 0.9 - 2.92 ) | 1.62 ( 2.59 ) | 1.56 ( 0.95 ) | 0.65 ( -0.19 ) |
| Posture Abnormal | 12 | 2.2 ( 1.21 - 4.01 ) | 2.2 ( 6.99 ) | 2.07 ( 1.25 ) | 1.05 ( 0.2 ) |
| Respiratory Distress | 12 | 4.03 ( 2.15 - 7.54 ) | 4.02 ( 22.26 ) | 3.47 ( 2.05 ) | 1.79 ( 0.92 ) |
| High-Pitched Crying | 11 | 5.15 ( 2.63 - 10.08 ) | 5.15 ( 28.5 ) | 4.22 ( 2.4 ) | 2.08 ( 1.15 ) |
| Gait Disturbance | 11 | 0.59 ( 0.32 - 1.07 ) | 0.59 ( 3.06 ) | 0.6 ( 0.36 ) | -0.73 ( -1.58 ) |
| Sepsis | 11 | 1.32 ( 0.72 - 2.44 ) | 1.32 ( 0.8 ) | 1.3 ( 0.78 ) | 0.38 ( -0.49 ) |
| Vaccination Site Reaction | 11 | 1.31 ( 0.71 - 2.42 ) | 1.31 ( 0.76 ) | 1.29 ( 0.77 ) | 0.37 ( -0.5 ) |
| Injection Site Rash | 11 | 0.54 ( 0.3 - 0.98 ) | 0.54 ( 4.23 ) | 0.55 ( 0.33 ) | -0.86 ( -1.7 ) |
| Hyperhidrosis | 11 | 2.25 ( 1.2 - 4.21 ) | 2.25 ( 6.77 ) | 2.11 ( 1.25 ) | 1.08 ( 0.19 ) |
| Flushing | 10 | 2.22 ( 1.15 - 4.29 ) | 2.22 ( 5.98 ) | 2.09 ( 1.2 ) | 1.06 ( 0.14 ) |
| Infantile Vomiting | 10 | 3.79 ( 1.91 - 7.5 ) | 3.78 ( 16.88 ) | 3.29 ( 1.86 ) | 1.72 ( 0.77 ) |
| Feeling Abnormal | 10 | 1.32 ( 0.69 - 2.51 ) | 1.32 ( 0.71 ) | 1.29 ( 0.76 ) | 0.37 ( -0.53 ) |
| Chills | 10 | 0.94 ( 0.5 - 1.78 ) | 0.94 ( 0.04 ) | 0.94 ( 0.55 ) | -0.08 ( -0.98 ) |
| Developmental Delay | 10 | 1.91 ( 1 - 3.67 ) | 1.91 ( 3.93 ) | 1.82 ( 1.06 ) | 0.87 ( -0.05 ) |
| Mobility Decreased | 10 | 1.35 ( 0.71 - 2.56 ) | 1.35 ( 0.83 ) | 1.32 ( 0.77 ) | 0.4 ( -0.5 ) |
| Severe Myoclonic Epilepsy Of Infancy | 9 | 40.05 ( 12.33 - 130.08 ) | 40 ( 105.3 ) | 13 ( 4.85 ) | 3.7 ( 2.51 ) |
| Decreased Eye Contact | 9 | 0.97 ( 0.5 - 1.9 ) | 0.97 ( 0.01 ) | 0.97 ( 0.55 ) | -0.04 ( -0.98 ) |
| Rhinorrhoea | 9 | 0.39 ( 0.2 - 0.76 ) | 0.39 ( 8.28 ) | 0.41 ( 0.23 ) | -1.3 ( -2.22 ) |
| Muscle Rigidity | 9 | 3.41 ( 1.67 - 6.95 ) | 3.4 ( 12.83 ) | 3.02 ( 1.66 ) | 1.59 ( 0.6 ) |
| Oedema Peripheral | 9 | 2.81 ( 1.39 - 5.68 ) | 2.81 ( 9.05 ) | 2.56 ( 1.42 ) | 1.36 ( 0.38 ) |
| Culture Urine Negative | 9 | 1.6 ( 0.81 - 3.17 ) | 1.6 ( 1.86 ) | 1.55 ( 0.88 ) | 0.63 ( -0.32 ) |
| Abnormal Faeces | 9 | 2.19 ( 1.1 - 4.39 ) | 2.19 ( 5.19 ) | 2.06 ( 1.15 ) | 1.04 ( 0.08 ) |
| Pruritus | 9 | 0.58 ( 0.3 - 1.13 ) | 0.58 ( 2.66 ) | 0.59 ( 0.34 ) | -0.75 ( -1.68 ) |
| Gastroenteritis Rotavirus | 9 | 0.66 ( 0.34 - 1.29 ) | 0.66 ( 1.48 ) | 0.68 ( 0.39 ) | -0.56 ( -1.5 ) |
| Atonic Seizures | 8 | 20.34 ( 7.37 - 56.1 ) | 20.32 ( 68.57 ) | 10.01 ( 4.28 ) | 3.32 ( 2.12 ) |
| Tonic Clonic Movements | 8 | 5.93 ( 2.66 - 13.21 ) | 5.93 ( 24.57 ) | 4.69 ( 2.4 ) | 2.23 ( 1.15 ) |
| Salivary Hypersecretion | 8 | 3.23 ( 1.52 - 6.87 ) | 3.23 ( 10.44 ) | 2.89 ( 1.54 ) | 1.53 ( 0.49 ) |
| Gastrooesophageal Reflux Disease | 8 | 2.69 ( 1.28 - 5.65 ) | 2.68 ( 7.34 ) | 2.46 ( 1.32 ) | 1.3 ( 0.27 ) |
| Muscle Twitching | 8 | 1.17 ( 0.57 - 2.38 ) | 1.17 ( 0.18 ) | 1.16 ( 0.63 ) | 0.21 ( -0.79 ) |
| Opisthotonus | 8 | 2.45 ( 1.17 - 5.14 ) | 2.45 ( 6.05 ) | 2.28 ( 1.23 ) | 1.19 ( 0.16 ) |
| Dry Skin | 8 | 2.69 ( 1.28 - 5.65 ) | 2.68 ( 7.34 ) | 2.46 ( 1.32 ) | 1.3 ( 0.27 ) |
| Gastroenteritis | 8 | 0.99 ( 0.48 - 2.01 ) | 0.99 ( 0 ) | 0.99 ( 0.54 ) | -0.02 ( -1.01 ) |
| Neonatal Hypoxia | 8 | 8.37 ( 3.61 - 19.41 ) | 8.37 ( 35.29 ) | 6.01 ( 2.97 ) | 2.59 ( 1.47 ) |
| Influenza Virus Test Negative | 8 | 0.68 ( 0.34 - 1.39 ) | 0.68 ( 1.13 ) | 0.7 ( 0.39 ) | -0.52 ( -1.51 ) |
| Viral Test Negative | 8 | 1.8 ( 0.87 - 3.73 ) | 1.8 ( 2.59 ) | 1.73 ( 0.94 ) | 0.79 ( -0.22 ) |
| Platelet Count Increased | 8 | 1.39 ( 0.68 - 2.87 ) | 1.39 ( 0.83 ) | 1.37 ( 0.75 ) | 0.45 ( -0.55 ) |
| Blood Glucose Increased | 8 | 2.03 ( 0.98 - 4.23 ) | 2.03 ( 3.76 ) | 1.93 ( 1.04 ) | 0.95 ( -0.07 ) |
| Eye Swelling | 8 | 1.6 ( 0.78 - 3.3 ) | 1.6 ( 1.64 ) | 1.55 ( 0.85 ) | 0.63 ( -0.37 ) |
| Respiratory Rate Increased | 8 | 2.97 ( 1.4 - 6.27 ) | 2.96 ( 8.92 ) | 2.68 ( 1.43 ) | 1.42 ( 0.39 ) |
| Choking | 8 | 1.76 ( 0.85 - 3.63 ) | 1.76 ( 2.37 ) | 1.69 ( 0.92 ) | 0.76 ( -0.25 ) |
| Hypotension | 7 | 7.33 ( 3.04 - 17.67 ) | 7.32 ( 27.06 ) | 5.48 ( 2.62 ) | 2.45 ( 1.28 ) |
| Rash Papular | 7 | 0.45 ( 0.21 - 0.94 ) | 0.45 ( 4.71 ) | 0.46 ( 0.25 ) | -1.12 ( -2.16 ) |
| Pain In Extremity | 7 | 0.6 ( 0.28 - 1.27 ) | 0.6 ( 1.83 ) | 0.61 ( 0.33 ) | -0.71 ( -1.75 ) |
| Autism Spectrum Disorder | 7 | 0.78 ( 0.37 - 1.67 ) | 0.78 ( 0.41 ) | 0.79 ( 0.42 ) | -0.34 ( -1.38 ) |
| Cold Sweat | 7 | 3.89 ( 1.72 - 8.82 ) | 3.89 ( 12.33 ) | 3.37 ( 1.7 ) | 1.75 ( 0.63 ) |
| Peripheral Coldness | 7 | 2.22 ( 1.01 - 4.88 ) | 2.22 ( 4.18 ) | 2.09 ( 1.08 ) | 1.06 ( -0.02 ) |
| Vaccination Site Pain | 7 | 1.09 ( 0.51 - 2.34 ) | 1.09 ( 0.05 ) | 1.09 ( 0.57 ) | 0.12 ( -0.93 ) |
| Insomnia | 7 | 0.52 ( 0.24 - 1.09 ) | 0.52 ( 3.09 ) | 0.53 ( 0.28 ) | -0.92 ( -1.95 ) |
| Ultrasound Scan Abnormal | 7 | 1.17 ( 0.55 - 2.52 ) | 1.17 ( 0.17 ) | 1.16 ( 0.61 ) | 0.22 ( -0.84 ) |
| Vaccination Failure | 7 | 0.47 ( 0.22 - 1 ) | 0.47 ( 4.05 ) | 0.49 ( 0.26 ) | -1.04 ( -2.08 ) |
| Respiratory Tract Congestion | 7 | 0.81 ( 0.38 - 1.72 ) | 0.81 ( 0.31 ) | 0.82 ( 0.43 ) | -0.29 ( -1.34 ) |
| Leukocytosis | 7 | 2.01 ( 0.92 - 4.39 ) | 2.01 ( 3.18 ) | 1.9 ( 0.99 ) | 0.93 ( -0.15 ) |
| Psychomotor Skills Impaired | 7 | 5.19 ( 2.24 - 12.05 ) | 5.18 ( 18.31 ) | 4.24 ( 2.1 ) | 2.08 ( 0.94 ) |
| Respiratory Syncytial Virus Test Negative | 7 | 0.86 ( 0.4 - 1.85 ) | 0.86 ( 0.14 ) | 0.87 ( 0.46 ) | -0.2 ( -1.25 ) |
| Lividity | 7 | 2.49 ( 1.13 - 5.49 ) | 2.49 ( 5.47 ) | 2.31 ( 1.19 ) | 1.21 ( 0.12 ) |
| Pharyngitis | 7 | 1.62 ( 0.75 - 3.51 ) | 1.62 ( 1.51 ) | 1.56 ( 0.82 ) | 0.65 ( -0.42 ) |
| Neutrophil Count Increased | 7 | 2.18 ( 1 - 4.79 ) | 2.18 ( 4 ) | 2.05 ( 1.06 ) | 1.04 ( -0.04 ) |
| Cardiac Arrest | 7 | 1.98 ( 0.9 - 4.32 ) | 1.98 ( 3.03 ) | 1.88 ( 0.98 ) | 0.91 ( -0.17 ) |
| Lymphadenopathy | 7 | 0.7 ( 0.33 - 1.5 ) | 0.7 ( 0.85 ) | 0.71 ( 0.38 ) | -0.49 ( -1.53 ) |
| Hypersomnia | 6 | 0.93 ( 0.41 - 2.11 ) | 0.93 ( 0.03 ) | 0.93 ( 0.47 ) | -0.1 ( -1.22 ) |
| Emotional Distress | 6 | 0.87 ( 0.39 - 1.98 ) | 0.87 ( 0.1 ) | 0.88 ( 0.44 ) | -0.18 ( -1.3 ) |
| Thrombocytopenia | 6 | 0.9 ( 0.39 - 2.04 ) | 0.9 ( 0.07 ) | 0.9 ( 0.45 ) | -0.15 ( -1.27 ) |
| Milk Allergy | 6 | 2.54 ( 1.08 - 5.98 ) | 2.54 ( 4.9 ) | 2.35 ( 1.15 ) | 1.23 ( 0.06 ) |
| Kawasaki's Disease | 6 | 0.8 ( 0.35 - 1.8 ) | 0.8 ( 0.3 ) | 0.8 ( 0.41 ) | -0.31 ( -1.43 ) |
| Breath Holding | 6 | 1.59 ( 0.69 - 3.67 ) | 1.59 ( 1.21 ) | 1.54 ( 0.77 ) | 0.63 ( -0.52 ) |
| Cellulitis | 6 | 0.73 ( 0.32 - 1.65 ) | 0.73 ( 0.57 ) | 0.74 ( 0.37 ) | -0.43 ( -1.55 ) |
| Tachypnoea | 6 | 2.32 ( 0.99 - 5.43 ) | 2.32 ( 3.98 ) | 2.17 ( 1.06 ) | 1.12 ( -0.05 ) |
| Irritability Postvaccinal | 6 | 4.45 ( 1.82 - 10.88 ) | 4.44 ( 12.82 ) | 3.76 ( 1.78 ) | 1.91 ( 0.7 ) |
| Covid-19 | 6 | 0.9 ( 0.39 - 2.04 ) | 0.9 ( 0.07 ) | 0.9 ( 0.45 ) | -0.15 ( -1.27 ) |
| Middle Insomnia | 6 | 2.01 ( 0.87 - 4.69 ) | 2.01 ( 2.75 ) | 1.91 ( 0.94 ) | 0.93 ( -0.22 ) |
| Petit Mal Epilepsy | 6 | 3.56 ( 1.48 - 8.55 ) | 3.56 ( 9.19 ) | 3.13 ( 1.5 ) | 1.65 ( 0.46 ) |
| Sudden Infant Death Syndrome | 6 | 1.32 ( 0.57 - 3.02 ) | 1.32 ( 0.43 ) | 1.29 ( 0.65 ) | 0.37 ( -0.76 ) |
| Altered State Of Consciousness | 6 | 1.37 ( 0.6 - 3.14 ) | 1.37 ( 0.55 ) | 1.34 ( 0.67 ) | 0.42 ( -0.71 ) |
| Weight Decreased | 6 | 1.69 ( 0.73 - 3.91 ) | 1.69 ( 1.55 ) | 1.63 ( 0.81 ) | 0.71 ( -0.44 ) |
| Retching | 6 | 2.43 ( 1.03 - 5.69 ) | 2.42 ( 4.42 ) | 2.25 ( 1.1 ) | 1.17 ( 0.01 ) |
| Partial Seizures | 6 | 2.13 ( 0.91 - 4.98 ) | 2.13 ( 3.23 ) | 2.01 ( 0.99 ) | 1.01 ( -0.15 ) |
| Respiration Abnormal | 6 | 1.98 ( 0.85 - 4.59 ) | 1.98 ( 2.6 ) | 1.88 ( 0.93 ) | 0.91 ( -0.24 ) |
| Neurodermatitis | 6 | 21.35 ( 6.51 - 69.97 ) | 21.33 ( 52.86 ) | 10.24 ( 3.79 ) | 3.36 ( 1.98 ) |
| Speech Disorder | 6 | 1.04 ( 0.45 - 2.36 ) | 1.04 ( 0.01 ) | 1.03 ( 0.52 ) | 0.05 ( -1.08 ) |
| Frequent Bowel Movements | 6 | 1.72 ( 0.74 - 3.98 ) | 1.72 ( 1.65 ) | 1.66 ( 0.82 ) | 0.73 ( -0.42 ) |
| Gastrointestinal Disorder | 6 | 1.5 ( 0.65 - 3.46 ) | 1.5 ( 0.93 ) | 1.46 ( 0.73 ) | 0.55 ( -0.59 ) |
| Rectal Haemorrhage | 6 | 3.56 ( 1.48 - 8.55 ) | 3.56 ( 9.19 ) | 3.13 ( 1.5 ) | 1.65 ( 0.46 ) |
| Injection Site Haemorrhage | 6 | 4.85 ( 1.97 - 11.97 ) | 4.85 ( 14.4 ) | 4.02 ( 1.89 ) | 2.01 ( 0.79 ) |
| Wheezing | 6 | 0.88 ( 0.39 - 2 ) | 0.88 ( 0.09 ) | 0.89 ( 0.45 ) | -0.17 ( -1.29 ) |
| Blood Lactic Acid Increased | 6 | 5.08 ( 2.05 - 12.6 ) | 5.08 ( 15.29 ) | 4.17 ( 1.95 ) | 2.06 ( 0.84 ) |
| Injection Site Oedema | 6 | 1.09 ( 0.48 - 2.48 ) | 1.09 ( 0.04 ) | 1.08 ( 0.54 ) | 0.12 ( -1.01 ) |
| Fontanelle Bulging | 5 | 2.28 ( 0.9 - 5.79 ) | 2.28 ( 3.18 ) | 2.13 ( 0.98 ) | 1.09 ( -0.16 ) |
| Anaphylactic Shock | 5 | 2.28 ( 0.9 - 5.79 ) | 2.28 ( 3.18 ) | 2.13 ( 0.98 ) | 1.09 ( -0.16 ) |
| Neutrophil Count Decreased | 5 | 1.11 ( 0.45 - 2.74 ) | 1.11 ( 0.05 ) | 1.1 ( 0.52 ) | 0.14 ( -1.08 ) |
| Vaccination Site Induration | 5 | 1.1 ( 0.44 - 2.71 ) | 1.1 ( 0.04 ) | 1.09 ( 0.51 ) | 0.13 ( -1.09 ) |
| Electrocardiogram Abnormal | 5 | 3.42 ( 1.31 - 8.91 ) | 3.42 ( 7.18 ) | 3.03 ( 1.36 ) | 1.6 ( 0.31 ) |
| Polymerase Chain Reaction Positive | 5 | 0.63 ( 0.26 - 1.55 ) | 0.63 ( 1.01 ) | 0.65 ( 0.31 ) | -0.63 ( -1.83 ) |
| Haematemesis | 5 | 4.94 ( 1.83 - 13.31 ) | 4.94 ( 12.29 ) | 4.08 ( 1.78 ) | 2.03 ( 0.71 ) |
| Hypopnoea | 5 | 2.54 ( 1 - 6.49 ) | 2.54 ( 4.08 ) | 2.35 ( 1.07 ) | 1.23 ( -0.03 ) |
| Sars-Cov-2 Test Positive | 5 | 1.03 ( 0.42 - 2.55 ) | 1.03 ( 0.01 ) | 1.03 ( 0.49 ) | 0.05 ( -1.17 ) |
| Seizure Like Phenomena | 5 | 0.91 ( 0.37 - 2.23 ) | 0.91 ( 0.05 ) | 0.91 ( 0.43 ) | -0.13 ( -1.35 ) |
| Viral Infection | 5 | 0.69 ( 0.28 - 1.68 ) | 0.69 ( 0.68 ) | 0.7 ( 0.33 ) | -0.51 ( -1.72 ) |
| Depressed Mood | 5 | 5.56 ( 2.04 - 15.18 ) | 5.56 ( 14.23 ) | 4.47 ( 1.93 ) | 2.16 ( 0.83 ) |
| Urinary Tract Infection | 5 | 1.13 ( 0.46 - 2.78 ) | 1.13 ( 0.07 ) | 1.12 ( 0.52 ) | 0.16 ( -1.06 ) |
| Sopor | 5 | 12.71 ( 4.03 - 40.04 ) | 12.7 ( 31.43 ) | 7.82 ( 2.99 ) | 2.97 ( 1.54 ) |
| Heart Rate Decreased | 5 | 8.89 ( 3.04 - 26.03 ) | 8.89 ( 23.34 ) | 6.26 ( 2.55 ) | 2.65 ( 1.26 ) |
| Nausea | 5 | 0.74 ( 0.3 - 1.81 ) | 0.74 ( 0.44 ) | 0.75 ( 0.36 ) | -0.41 ( -1.62 ) |
| Alanine Aminotransferase Increased | 5 | 1.41 ( 0.57 - 3.51 ) | 1.41 ( 0.55 ) | 1.38 ( 0.64 ) | 0.47 ( -0.76 ) |
| Blood Alkaline Phosphatase Increased | 5 | 2.17 ( 0.86 - 5.49 ) | 2.17 ( 2.8 ) | 2.04 ( 0.94 ) | 1.03 ( -0.22 ) |
| Infantile Spitting Up | 5 | 1.19 ( 0.48 - 2.93 ) | 1.19 ( 0.14 ) | 1.17 ( 0.55 ) | 0.23 ( -0.99 ) |
| Foaming At Mouth | 5 | 2.12 ( 0.84 - 5.35 ) | 2.12 ( 2.63 ) | 2 ( 0.92 ) | 1 ( -0.25 ) |
| Rash Maculo-Papular | 5 | 0.46 ( 0.19 - 1.12 ) | 0.46 ( 3.08 ) | 0.47 ( 0.23 ) | -1.08 ( -2.27 ) |
| Diet Refusal | 5 | 0.92 ( 0.37 - 2.25 ) | 0.92 ( 0.04 ) | 0.92 ( 0.43 ) | -0.12 ( -1.33 ) |
| Chest X-Ray Abnormal | 5 | 0.88 ( 0.36 - 2.16 ) | 0.88 ( 0.08 ) | 0.89 ( 0.42 ) | -0.18 ( -1.39 ) |
| Platelet Count Decreased | 5 | 0.31 ( 0.13 - 0.75 ) | 0.31 ( 7.53 ) | 0.32 ( 0.15 ) | -1.63 ( -2.82 ) |
| Tension | 5 | 5.56 ( 2.04 - 15.18 ) | 5.56 ( 14.23 ) | 4.47 ( 1.93 ) | 2.16 ( 0.83 ) |
| Urine Output Decreased | 5 | 1.78 ( 0.71 - 4.46 ) | 1.78 ( 1.55 ) | 1.71 ( 0.79 ) | 0.77 ( -0.47 ) |
| Injection Site Discolouration | 5 | 1.25 ( 0.51 - 3.1 ) | 1.25 ( 0.24 ) | 1.24 ( 0.58 ) | 0.3 ( -0.92 ) |
| Pharyngeal Erythema | 5 | 0.95 ( 0.38 - 2.33 ) | 0.95 ( 0.01 ) | 0.95 ( 0.45 ) | -0.08 ( -1.29 ) |
| Haematocrit Decreased | 5 | 1.13 ( 0.46 - 2.78 ) | 1.13 ( 0.07 ) | 1.12 ( 0.52 ) | 0.16 ( -1.06 ) |
| Apparent Life Threatening Event | 5 | 5.56 ( 2.04 - 15.18 ) | 5.56 ( 14.23 ) | 4.47 ( 1.93 ) | 2.16 ( 0.83 ) |
| Meningitis | 5 | 1.53 ( 0.61 - 3.82 ) | 1.53 ( 0.85 ) | 1.49 ( 0.69 ) | 0.58 ( -0.66 ) |
| Movement Disorder | 4 | 1.69 ( 0.61 - 4.72 ) | 1.69 ( 1.04 ) | 1.63 ( 0.69 ) | 0.71 ( -0.65 ) |
| Coma | 4 | 5.47 ( 1.78 - 16.79 ) | 5.47 ( 11.17 ) | 4.42 ( 1.73 ) | 2.14 ( 0.68 ) |
| Food Allergy | 4 | 1.11 ( 0.4 - 3.05 ) | 1.11 ( 0.04 ) | 1.1 ( 0.47 ) | 0.14 ( -1.19 ) |
| Asthma | 4 | 1.51 ( 0.55 - 4.2 ) | 1.51 ( 0.64 ) | 1.47 ( 0.63 ) | 0.56 ( -0.79 ) |
| Cerebral Haemorrhage | 4 | 7.11 ( 2.23 - 22.69 ) | 7.11 ( 15.01 ) | 5.36 ( 2.03 ) | 2.42 ( 0.93 ) |
| Nervousness | 4 | 1.37 ( 0.49 - 3.78 ) | 1.37 ( 0.37 ) | 1.34 ( 0.57 ) | 0.42 ( -0.92 ) |
| Presyncope | 4 | 1.73 ( 0.62 - 4.84 ) | 1.73 ( 1.13 ) | 1.67 ( 0.71 ) | 0.74 ( -0.62 ) |
| Respiratory Tract Infection | 4 | 1.25 ( 0.45 - 3.44 ) | 1.25 ( 0.18 ) | 1.23 ( 0.53 ) | 0.3 ( -1.04 ) |
| Moaning | 4 | 1.58 ( 0.57 - 4.4 ) | 1.58 ( 0.78 ) | 1.53 ( 0.65 ) | 0.62 ( -0.74 ) |
| Vaccination Site Oedema | 4 | 0.63 ( 0.23 - 1.71 ) | 0.63 ( 0.84 ) | 0.64 ( 0.28 ) | -0.64 ( -1.96 ) |
| Eyelid Oedema | 4 | 2.96 ( 1.03 - 8.54 ) | 2.96 ( 4.46 ) | 2.68 ( 1.11 ) | 1.42 ( 0.03 ) |
| Induration | 4 | 0.51 ( 0.19 - 1.37 ) | 0.51 ( 1.86 ) | 0.52 ( 0.23 ) | -0.94 ( -2.25 ) |
| Nasopharyngitis | 4 | 0.6 ( 0.22 - 1.62 ) | 0.6 ( 1.05 ) | 0.61 ( 0.27 ) | -0.71 ( -2.03 ) |
| Nystagmus | 4 | 3.39 ( 1.16 - 9.87 ) | 3.39 ( 5.65 ) | 3 ( 1.23 ) | 1.59 ( 0.18 ) |
| Human Rhinovirus Test Positive | 4 | 1.32 ( 0.48 - 3.64 ) | 1.32 ( 0.28 ) | 1.29 ( 0.55 ) | 0.37 ( -0.97 ) |
| Ecchymosis | 4 | 1.73 ( 0.62 - 4.84 ) | 1.73 ( 1.13 ) | 1.67 ( 0.71 ) | 0.74 ( -0.62 ) |
| Irregular Breathing | 4 | 11.86 ( 3.35 - 42.03 ) | 11.85 ( 23.85 ) | 7.51 ( 2.61 ) | 2.91 ( 1.35 ) |
| Swelling Face | 4 | 0.46 ( 0.17 - 1.24 ) | 0.46 ( 2.49 ) | 0.47 ( 0.21 ) | -1.08 ( -2.39 ) |
| Metabolic Function Test Abnormal | 4 | 5.93 ( 1.91 - 18.39 ) | 5.93 ( 12.28 ) | 4.69 ( 1.82 ) | 2.23 ( 0.76 ) |
| Fluid Intake Reduced | 4 | 1.55 ( 0.56 - 4.3 ) | 1.55 ( 0.71 ) | 1.5 ( 0.64 ) | 0.59 ( -0.77 ) |
| Dysstasia | 4 | 1.05 ( 0.38 - 2.87 ) | 1.05 ( 0.01 ) | 1.04 ( 0.45 ) | 0.06 ( -1.27 ) |
| Lymphocyte Percentage Increased | 4 | 0.72 ( 0.26 - 1.95 ) | 0.72 ( 0.43 ) | 0.73 ( 0.32 ) | -0.46 ( -1.78 ) |
| Neutrophil Percentage Decreased | 4 | 0.63 ( 0.23 - 1.72 ) | 0.63 ( 0.81 ) | 0.65 ( 0.28 ) | -0.63 ( -1.95 ) |
| Encephalopathy | 4 | 1.09 ( 0.4 - 3 ) | 1.09 ( 0.03 ) | 1.09 ( 0.47 ) | 0.12 ( -1.21 ) |
| Mass | 4 | 2.74 ( 0.95 - 7.84 ) | 2.73 ( 3.82 ) | 2.5 ( 1.04 ) | 1.32 ( -0.07 ) |
| Inflammation | 4 | 0.71 ( 0.26 - 1.93 ) | 0.71 ( 0.45 ) | 0.72 ( 0.31 ) | -0.47 ( -1.79 ) |
| Aspartate Aminotransferase Increased | 4 | 0.87 ( 0.32 - 2.37 ) | 0.87 ( 0.08 ) | 0.87 ( 0.38 ) | -0.2 ( -1.52 ) |
| Dermatitis | 4 | 2.37 ( 0.84 - 6.73 ) | 2.37 ( 2.8 ) | 2.21 ( 0.92 ) | 1.14 ( -0.24 ) |
| Aphasia | 4 | 0.55 ( 0.2 - 1.49 ) | 0.55 ( 1.42 ) | 0.56 ( 0.25 ) | -0.82 ( -2.14 ) |
| Facial Paralysis | 4 | 1.82 ( 0.65 - 5.1 ) | 1.82 ( 1.35 ) | 1.75 ( 0.74 ) | 0.8 ( -0.56 ) |
| Body Temperature Decreased | 4 | 1.62 ( 0.58 - 4.5 ) | 1.62 ( 0.86 ) | 1.56 ( 0.66 ) | 0.65 ( -0.71 ) |
| Gait Inability | 4 | 0.5 ( 0.19 - 1.35 ) | 0.5 ( 1.94 ) | 0.51 ( 0.22 ) | -0.96 ( -2.27 ) |
| Bronchitis | 4 | 0.87 ( 0.32 - 2.37 ) | 0.87 ( 0.08 ) | 0.87 ( 0.38 ) | -0.2 ( -1.52 ) |
| Echocardiogram Abnormal | 4 | 1.92 ( 0.68 - 5.39 ) | 1.92 ( 1.6 ) | 1.83 ( 0.77 ) | 0.87 ( -0.49 ) |
| Inflammatory Marker Increased | 4 | 1.98 ( 0.7 - 5.55 ) | 1.98 ( 1.73 ) | 1.88 ( 0.79 ) | 0.91 ( -0.46 ) |
| Postictal State | 4 | 1.55 ( 0.56 - 4.3 ) | 1.55 ( 0.71 ) | 1.5 ( 0.64 ) | 0.59 ( -0.77 ) |
| Feeling Hot | 4 | 0.95 ( 0.35 - 2.59 ) | 0.95 ( 0.01 ) | 0.95 ( 0.41 ) | -0.07 ( -1.4 ) |
| Productive Cough | 4 | 1.37 ( 0.49 - 3.78 ) | 1.37 ( 0.37 ) | 1.34 ( 0.57 ) | 0.42 ( -0.92 ) |
| Rhinitis | 4 | 1.27 ( 0.46 - 3.5 ) | 1.27 ( 0.21 ) | 1.25 ( 0.54 ) | 0.32 ( -1.02 ) |
| Blood Potassium Increased | 4 | 1.82 ( 0.65 - 5.1 ) | 1.82 ( 1.35 ) | 1.75 ( 0.74 ) | 0.8 ( -0.56 ) |
| Influenza Like Illness | 4 | 2.45 ( 0.86 - 6.98 ) | 2.45 ( 3.02 ) | 2.28 ( 0.95 ) | 1.19 ( -0.2 ) |
| Monocyte Count Increased | 4 | 1.21 ( 0.44 - 3.32 ) | 1.21 ( 0.13 ) | 1.19 ( 0.51 ) | 0.25 ( -1.09 ) |
| Headache | 3 | 0.32 ( 0.1 - 1 ) | 0.32 ( 4.28 ) | 0.33 ( 0.13 ) | -1.59 ( -3.05 ) |
| Meningism | 3 | 5.33 ( 1.47 - 19.39 ) | 5.33 ( 8.13 ) | 4.33 ( 1.47 ) | 2.12 ( 0.48 ) |
| Haemorrhage | 3 | 1.05 ( 0.33 - 3.35 ) | 1.05 ( 0.01 ) | 1.04 ( 0.39 ) | 0.06 ( -1.43 ) |
| Dysphagia | 3 | 1.4 ( 0.43 - 4.55 ) | 1.4 ( 0.32 ) | 1.37 ( 0.51 ) | 0.46 ( -1.05 ) |
| Heart Rate Increased | 3 | 0.86 ( 0.27 - 2.74 ) | 0.86 ( 0.07 ) | 0.87 ( 0.33 ) | -0.21 ( -1.69 ) |
| Coronary Artery Aneurysm | 3 | 10.67 ( 2.55 - 44.66 ) | 10.67 ( 16.43 ) | 7.04 ( 2.13 ) | 2.82 ( 1.08 ) |
| Speech Disorder Developmental | 3 | 0.54 ( 0.17 - 1.72 ) | 0.54 ( 1.11 ) | 0.56 ( 0.21 ) | -0.84 ( -2.31 ) |
| Clonus | 3 | 3.81 ( 1.09 - 13.26 ) | 3.81 ( 5.12 ) | 3.31 ( 1.17 ) | 1.73 ( 0.13 ) |
| Hyperaesthesia | 3 | 1.98 ( 0.6 - 6.51 ) | 1.98 ( 1.3 ) | 1.88 ( 0.69 ) | 0.91 ( -0.62 ) |
| Cyanosis Central | 3 | 8.89 ( 2.22 - 35.56 ) | 8.89 ( 14 ) | 6.26 ( 1.96 ) | 2.65 ( 0.94 ) |
| Respiratory Rate Decreased | 3 | 4.1 ( 1.17 - 14.4 ) | 4.1 ( 5.72 ) | 3.52 ( 1.23 ) | 1.82 ( 0.21 ) |
| Enterocolitis | 3 | 3.56 ( 1.03 - 12.29 ) | 3.56 ( 4.59 ) | 3.13 ( 1.11 ) | 1.65 ( 0.06 ) |
| Tic | 3 | 1.72 ( 0.53 - 5.63 ) | 1.72 ( 0.83 ) | 1.66 ( 0.61 ) | 0.73 ( -0.79 ) |
| Epistaxis | 3 | 0.99 ( 0.31 - 3.16 ) | 0.99 ( 0 ) | 0.99 ( 0.37 ) | -0.02 ( -1.51 ) |
| Food Intolerance | 3 | 2.67 ( 0.79 - 8.98 ) | 2.67 ( 2.72 ) | 2.45 ( 0.89 ) | 1.29 ( -0.27 ) |
| Language Disorder | 3 | 2.13 ( 0.64 - 7.07 ) | 2.13 ( 1.61 ) | 2.01 ( 0.74 ) | 1.01 ( -0.53 ) |
| Slow Response To Stimuli | 3 | 2.22 ( 0.67 - 7.38 ) | 2.22 ( 1.79 ) | 2.09 ( 0.76 ) | 1.06 ( -0.48 ) |
| Neutropenia | 3 | 0.85 ( 0.27 - 2.7 ) | 0.85 ( 0.08 ) | 0.85 ( 0.32 ) | -0.23 ( -1.71 ) |
| Ear Infection | 3 | 0.26 ( 0.08 - 0.8 ) | 0.26 ( 6.34 ) | 0.27 ( 0.1 ) | -1.9 ( -3.36 ) |
| Injection Site Inflammation | 3 | 0.83 ( 0.26 - 2.65 ) | 0.83 ( 0.1 ) | 0.84 ( 0.32 ) | -0.25 ( -1.74 ) |
| Erythema Multiforme | 3 | 0.59 ( 0.19 - 1.85 ) | 0.59 ( 0.85 ) | 0.6 ( 0.23 ) | -0.74 ( -2.21 ) |
| Muscular Weakness | 3 | 0.67 ( 0.21 - 2.14 ) | 0.68 ( 0.45 ) | 0.69 ( 0.26 ) | -0.54 ( -2.02 ) |
| Injection Site Pruritus | 3 | 0.52 ( 0.16 - 1.63 ) | 0.52 ( 1.31 ) | 0.53 ( 0.2 ) | -0.91 ( -2.38 ) |
| Purpura | 3 | 0.77 ( 0.24 - 2.46 ) | 0.77 ( 0.19 ) | 0.78 ( 0.3 ) | -0.35 ( -1.84 ) |
| Vaccination Site Discolouration | 3 | 5.33 ( 1.47 - 19.39 ) | 5.33 ( 8.13 ) | 4.33 ( 1.47 ) | 2.12 ( 0.48 ) |
| Swollen Tongue | 3 | 2.67 ( 0.79 - 8.98 ) | 2.67 ( 2.72 ) | 2.45 ( 0.89 ) | 1.29 ( -0.27 ) |
| Csf Test Abnormal | 3 | 2.54 ( 0.76 - 8.52 ) | 2.54 ( 2.45 ) | 2.35 ( 0.85 ) | 1.23 ( -0.32 ) |
| Tenderness | 3 | 0.76 ( 0.24 - 2.42 ) | 0.76 ( 0.21 ) | 0.77 ( 0.29 ) | -0.37 ( -1.86 ) |
| Drooling | 3 | 0.99 ( 0.31 - 3.16 ) | 0.99 ( 0 ) | 0.99 ( 0.37 ) | -0.02 ( -1.51 ) |
| Bronchiolitis | 3 | 1.11 ( 0.35 - 3.57 ) | 1.11 ( 0.03 ) | 1.1 ( 0.42 ) | 0.14 ( -1.35 ) |
| Full Blood Count Abnormal | 3 | 1.24 ( 0.38 - 4 ) | 1.24 ( 0.13 ) | 1.22 ( 0.46 ) | 0.29 ( -1.21 ) |
| Focal Dyscognitive Seizures | 3 | 10.67 ( 2.55 - 44.66 ) | 10.67 ( 16.43 ) | 7.04 ( 2.13 ) | 2.82 ( 1.08 ) |
| Shock | 3 | 1.91 ( 0.58 - 6.27 ) | 1.9 ( 1.16 ) | 1.82 ( 0.67 ) | 0.86 ( -0.67 ) |
| Basophil Percentage Decreased | 3 | 1.44 ( 0.44 - 4.68 ) | 1.44 ( 0.38 ) | 1.41 ( 0.53 ) | 0.49 ( -1.02 ) |
| Eosinophil Percentage Decreased | 3 | 1.37 ( 0.42 - 4.43 ) | 1.37 ( 0.28 ) | 1.34 ( 0.5 ) | 0.42 ( -1.09 ) |
| Monocyte Percentage Increased | 3 | 0.94 ( 0.29 - 2.99 ) | 0.94 ( 0.01 ) | 0.94 ( 0.36 ) | -0.09 ( -1.58 ) |
| Red Blood Cell Count Decreased | 3 | 1.67 ( 0.51 - 5.44 ) | 1.67 ( 0.73 ) | 1.61 ( 0.6 ) | 0.69 ( -0.83 ) |
| Blood Sodium Decreased | 3 | 0.99 ( 0.31 - 3.16 ) | 0.99 ( 0 ) | 0.99 ( 0.37 ) | -0.02 ( -1.51 ) |
| Haemoglobin Increased | 3 | 2.67 ( 0.79 - 8.98 ) | 2.67 ( 2.72 ) | 2.45 ( 0.89 ) | 1.29 ( -0.27 ) |
| Neutrophil Percentage | 3 | 1.01 ( 0.31 - 3.22 ) | 1.01 ( 0 ) | 1.01 ( 0.38 ) | 0.01 ( -1.48 ) |
| Injection Site Urticaria | 3 | 0.28 ( 0.09 - 0.89 ) | 0.28 ( 5.35 ) | 0.29 ( 0.11 ) | -1.76 ( -3.22 ) |
| Haemoglobin Decreased | 3 | 0.61 ( 0.19 - 1.94 ) | 0.61 ( 0.71 ) | 0.63 ( 0.24 ) | -0.68 ( -2.15 ) |
| Local Reaction | 3 | 0.38 ( 0.12 - 1.2 ) | 0.38 ( 2.91 ) | 0.4 ( 0.15 ) | -1.33 ( -2.8 ) |
| Pneumonia | 3 | 0.2 ( 0.06 - 0.61 ) | 0.2 ( 9.75 ) | 0.21 ( 0.08 ) | -2.28 ( -3.74 ) |
| Culture Negative | 3 | 1.98 ( 0.6 - 6.51 ) | 1.98 ( 1.3 ) | 1.88 ( 0.69 ) | 0.91 ( -0.62 ) |
| Pco2 Decreased | 3 | 4.45 ( 1.25 - 15.76 ) | 4.44 ( 6.41 ) | 3.76 ( 1.3 ) | 1.91 ( 0.3 ) |
| Paralysis | 3 | 1.78 ( 0.54 - 5.83 ) | 1.78 ( 0.93 ) | 1.71 ( 0.63 ) | 0.77 ( -0.75 ) |
| Decreased Activity | 3 | 0.71 ( 0.22 - 2.25 ) | 0.71 ( 0.34 ) | 0.72 ( 0.27 ) | -0.47 ( -1.95 ) |
| Macule | 3 | 1.98 ( 0.6 - 6.51 ) | 1.98 ( 1.3 ) | 1.88 ( 0.69 ) | 0.91 ( -0.62 ) |
| Skin Reaction | 3 | 1.91 ( 0.58 - 6.27 ) | 1.9 ( 1.16 ) | 1.82 ( 0.67 ) | 0.86 ( -0.67 ) |
| Fontanelle Depressed | 3 | 6.67 ( 1.77 - 25.14 ) | 6.67 ( 10.51 ) | 5.12 ( 1.69 ) | 2.36 ( 0.69 ) |
| Muscle Tightness | 3 | 1.57 ( 0.48 - 5.11 ) | 1.57 ( 0.57 ) | 1.52 ( 0.57 ) | 0.61 ( -0.91 ) |
| Angioedema | 3 | 0.65 ( 0.21 - 2.06 ) | 0.65 ( 0.54 ) | 0.66 ( 0.25 ) | -0.59 ( -2.07 ) |
| Magnetic Resonance Imaging Head Abnormal | 3 | 0.97 ( 0.3 - 3.1 ) | 0.97 ( 0 ) | 0.97 ( 0.37 ) | -0.04 ( -1.53 ) |
| Culture Stool Negative | 3 | 1.48 ( 0.46 - 4.81 ) | 1.48 ( 0.43 ) | 1.44 ( 0.54 ) | 0.53 ( -0.98 ) |
| Otitis Media | 3 | 0.64 ( 0.2 - 2.03 ) | 0.64 ( 0.58 ) | 0.66 ( 0.25 ) | -0.61 ( -2.09 ) |
| Glassy Eyes | 3 | 3.56 ( 1.03 - 12.29 ) | 3.56 ( 4.59 ) | 3.13 ( 1.11 ) | 1.65 ( 0.06 ) |
| Pertussis | 3 | 3.14 ( 0.92 - 10.71 ) | 3.14 ( 3.71 ) | 2.82 ( 1.01 ) | 1.49 ( -0.08 ) |
| Sensory Processing Disorder | 3 | 3.14 ( 0.92 - 10.71 ) | 3.14 ( 3.71 ) | 2.82 ( 1.01 ) | 1.49 ( -0.08 ) |
| Eosinophil Count Decreased | 3 | 1.84 ( 0.56 - 6.04 ) | 1.84 ( 1.04 ) | 1.76 ( 0.65 ) | 0.82 ( -0.71 ) |
| Diarrhoea Haemorrhagic | 3 | 0.55 ( 0.17 - 1.73 ) | 0.55 ( 1.07 ) | 0.56 ( 0.22 ) | -0.83 ( -2.3 ) |
| Colitis | 3 | 3.56 ( 1.03 - 12.29 ) | 3.56 ( 4.59 ) | 3.13 ( 1.11 ) | 1.65 ( 0.06 ) |
| Hypoxia | 3 | 1.48 ( 0.46 - 4.81 ) | 1.48 ( 0.43 ) | 1.44 ( 0.54 ) | 0.53 ( -0.98 ) |
| Adenovirus Test Positive | 3 | 1.84 ( 0.56 - 6.04 ) | 1.84 ( 1.04 ) | 1.76 ( 0.65 ) | 0.82 ( -0.71 ) |
| Poor Quality Sleep | 3 | 0.55 ( 0.17 - 1.73 ) | 0.55 ( 1.07 ) | 0.56 ( 0.22 ) | -0.83 ( -2.3 ) |
| Hypothermia | 3 | 1.37 ( 0.42 - 4.43 ) | 1.37 ( 0.28 ) | 1.34 ( 0.5 ) | 0.42 ( -1.09 ) |
| Sudden Death | 3 | 1.01 ( 0.31 - 3.22 ) | 1.01 ( 0 ) | 1.01 ( 0.38 ) | 0.01 ( -1.48 ) |
| Dysphonia | 3 | 1.98 ( 0.6 - 6.51 ) | 1.98 ( 1.3 ) | 1.88 ( 0.69 ) | 0.91 ( -0.62 ) |
| Rash Generalised | 3 | 0.18 ( 0.06 - 0.56 ) | 0.18 ( 11.21 ) | 0.19 ( 0.07 ) | -2.42 ( -3.87 ) |
| Hypertension | 3 | 2.96 ( 0.87 - 10.06 ) | 2.96 ( 3.34 ) | 2.68 ( 0.96 ) | 1.42 ( -0.15 ) |
| Upper Respiratory Tract Infection | 3 | 0.32 ( 0.1 - 1.01 ) | 0.32 ( 4.18 ) | 0.34 ( 0.13 ) | -1.58 ( -3.04 ) |
| Dermatitis Atopic | 3 | 1.21 ( 0.38 - 3.9 ) | 1.21 ( 0.1 ) | 1.2 ( 0.45 ) | 0.26 ( -1.24 ) |
| Blood Pressure Decreased | 3 | 6.67 ( 1.77 - 25.14 ) | 6.67 ( 10.51 ) | 5.12 ( 1.69 ) | 2.36 ( 0.69 ) |
| White Blood Cell Count Decreased | 3 | 0.63 ( 0.2 - 1.98 ) | 0.63 ( 0.64 ) | 0.64 ( 0.24 ) | -0.64 ( -2.12 ) |
| Dizziness | 2 | 0.52 ( 0.13 - 2.1 ) | 0.52 ( 0.89 ) | 0.53 ( 0.16 ) | -0.92 ( -2.62 ) |
| Psychomotor Disadaptation Syndrome | 2 | 35.56 ( 3.22 - 392.26 ) | 35.55 ( 22.39 ) | 12.52 ( 1.68 ) | 3.65 ( 1.44 ) |
| Regurgitation | 2 | 0.91 ( 0.22 - 3.78 ) | 0.91 ( 0.02 ) | 0.92 ( 0.28 ) | -0.13 ( -1.85 ) |
| Allergy Test Positive | 2 | 1.78 ( 0.42 - 7.61 ) | 1.78 ( 0.62 ) | 1.71 ( 0.51 ) | 0.77 ( -1 ) |
| Coronavirus Infection | 2 | 35.56 ( 3.22 - 392.26 ) | 35.55 ( 22.39 ) | 12.52 ( 1.68 ) | 3.65 ( 1.44 ) |
| Illness | 2 | 0.96 ( 0.23 - 3.99 ) | 0.96 ( 0 ) | 0.96 ( 0.29 ) | -0.05 ( -1.78 ) |
| Neisseria Test Positive | 2 | 3.56 ( 0.78 - 16.23 ) | 3.56 ( 3.06 ) | 3.13 ( 0.88 ) | 1.65 ( -0.2 ) |
| Stupor | 2 | 5.93 ( 1.2 - 29.37 ) | 5.93 ( 6.14 ) | 4.69 ( 1.23 ) | 2.23 ( 0.31 ) |
| Blood Bicarbonate Decreased | 2 | 1.62 ( 0.38 - 6.87 ) | 1.62 ( 0.43 ) | 1.56 ( 0.47 ) | 0.65 ( -1.12 ) |
| Po2 Increased | 2 | 5.08 ( 1.06 - 24.46 ) | 5.08 ( 5.1 ) | 4.17 ( 1.12 ) | 2.06 ( 0.16 ) |
| Melaena | 2 | 1.32 ( 0.31 - 5.54 ) | 1.32 ( 0.14 ) | 1.29 ( 0.39 ) | 0.37 ( -1.37 ) |
| Dermatitis Allergic | 2 | 0.85 ( 0.2 - 3.5 ) | 0.85 ( 0.05 ) | 0.85 ( 0.26 ) | -0.23 ( -1.95 ) |
| Adenovirus Infection | 2 | 1.55 ( 0.36 - 6.56 ) | 1.55 ( 0.35 ) | 1.5 ( 0.45 ) | 0.59 ( -1.17 ) |
| Escherichia Infection | 2 | 3.23 ( 0.72 - 14.59 ) | 3.23 ( 2.61 ) | 2.89 ( 0.82 ) | 1.53 ( -0.31 ) |
| Clostridium Difficile Infection | 2 | 17.78 ( 2.5 - 126.26 ) | 17.78 ( 15.83 ) | 9.39 ( 1.82 ) | 3.23 ( 1.12 ) |
| Culture Stool Positive | 2 | 1.55 ( 0.36 - 6.56 ) | 1.55 ( 0.35 ) | 1.5 ( 0.45 ) | 0.59 ( -1.17 ) |
| Thrombosis | 2 | 11.85 ( 1.98 - 70.96 ) | 11.85 ( 11.92 ) | 7.51 ( 1.68 ) | 2.91 ( 0.87 ) |
| Stool Analysis Abnormal | 2 | 0.76 ( 0.18 - 3.11 ) | 0.76 ( 0.15 ) | 0.77 ( 0.23 ) | -0.38 ( -2.1 ) |
| Gross Motor Delay | 2 | 3.23 ( 0.72 - 14.59 ) | 3.23 ( 2.61 ) | 2.89 ( 0.82 ) | 1.53 ( -0.31 ) |
| Bronchospasm | 2 | 1.78 ( 0.42 - 7.61 ) | 1.78 ( 0.62 ) | 1.71 ( 0.51 ) | 0.77 ( -1 ) |
| Fine Motor Skill Dysfunction | 2 | 5.93 ( 1.2 - 29.37 ) | 5.93 ( 6.14 ) | 4.69 ( 1.23 ) | 2.23 ( 0.31 ) |
| Autoimmune Haemolytic Anaemia | 2 | 7.11 ( 1.38 - 36.67 ) | 7.11 ( 7.5 ) | 5.36 ( 1.36 ) | 2.42 ( 0.47 ) |
| Atrial Septal Defect | 2 | 2.54 ( 0.58 - 11.18 ) | 2.54 ( 1.63 ) | 2.35 ( 0.68 ) | 1.23 ( -0.58 ) |
| Visual Impairment | 2 | 2.22 ( 0.51 - 9.67 ) | 2.22 ( 1.2 ) | 2.09 ( 0.61 ) | 1.06 ( -0.73 ) |
| Behaviour Disorder | 2 | 3.23 ( 0.72 - 14.59 ) | 3.23 ( 2.61 ) | 2.89 ( 0.82 ) | 1.53 ( -0.31 ) |
| Injected Limb Mobility Decreased | 2 | 1.15 ( 0.27 - 4.79 ) | 1.15 ( 0.04 ) | 1.14 ( 0.34 ) | 0.19 ( -1.55 ) |
| Vaccination Site Mass | 2 | 1.08 ( 0.26 - 4.49 ) | 1.08 ( 0.01 ) | 1.07 ( 0.32 ) | 0.1 ( -1.63 ) |
| Skin Lesion | 2 | 0.21 ( 0.05 - 0.85 ) | 0.21 ( 5.87 ) | 0.22 ( 0.07 ) | -2.19 ( -3.87 ) |
| Vaccination Site Inflammation | 2 | 2.09 ( 0.48 - 9.06 ) | 2.09 ( 1.02 ) | 1.98 ( 0.58 ) | 0.98 ( -0.8 ) |
| Infantile Colic | 2 | 8.89 ( 1.63 - 48.55 ) | 8.89 ( 9.33 ) | 6.26 ( 1.51 ) | 2.65 ( 0.65 ) |
| Food Aversion | 2 | 2.09 ( 0.48 - 9.06 ) | 2.09 ( 1.02 ) | 1.98 ( 0.58 ) | 0.98 ( -0.8 ) |
| Laboratory Test Abnormal | 2 | 0.47 ( 0.11 - 1.9 ) | 0.47 ( 1.18 ) | 0.48 ( 0.15 ) | -1.05 ( -2.75 ) |
| Vaccination Site Pruritus | 2 | 3.56 ( 0.78 - 16.23 ) | 3.56 ( 3.06 ) | 3.13 ( 0.88 ) | 1.65 ( -0.2 ) |
| Dysarthria | 2 | 3.95 ( 0.85 - 18.29 ) | 3.95 ( 3.61 ) | 3.41 ( 0.95 ) | 1.77 ( -0.09 ) |
| Skin Disorder | 2 | 1.87 ( 0.44 - 8.04 ) | 1.87 ( 0.73 ) | 1.79 ( 0.53 ) | 0.84 ( -0.94 ) |
| Dyspepsia | 2 | 2.09 ( 0.48 - 9.06 ) | 2.09 ( 1.02 ) | 1.98 ( 0.58 ) | 0.98 ( -0.8 ) |
| Urine Analysis Abnormal | 2 | 1.08 ( 0.26 - 4.49 ) | 1.08 ( 0.01 ) | 1.07 ( 0.32 ) | 0.1 ( -1.63 ) |
| Lactose Intolerance | 2 | 3.56 ( 0.78 - 16.23 ) | 3.56 ( 3.06 ) | 3.13 ( 0.88 ) | 1.65 ( -0.2 ) |
| X-Ray Abnormal | 2 | 0.73 ( 0.18 - 2.98 ) | 0.73 ( 0.2 ) | 0.74 ( 0.23 ) | -0.44 ( -2.16 ) |
| Vision Blurred | 2 | 1.98 ( 0.46 - 8.52 ) | 1.98 ( 0.87 ) | 1.88 ( 0.55 ) | 0.91 ( -0.87 ) |
| Infantile Back Arching | 2 | 1.11 ( 0.27 - 4.64 ) | 1.11 ( 0.02 ) | 1.1 ( 0.33 ) | 0.14 ( -1.59 ) |
| Thrombocytosis | 2 | 2.96 ( 0.66 - 13.24 ) | 2.96 ( 2.23 ) | 2.68 ( 0.77 ) | 1.42 ( -0.4 ) |
| Sluggishness | 2 | 1.48 ( 0.35 - 6.27 ) | 1.48 ( 0.29 ) | 1.44 ( 0.43 ) | 0.53 ( -1.23 ) |
| Feeding Disorder | 2 | 0.68 ( 0.17 - 2.81 ) | 0.68 ( 0.28 ) | 0.7 ( 0.21 ) | -0.52 ( -2.24 ) |
| Metabolic Acidosis | 2 | 1.78 ( 0.42 - 7.61 ) | 1.78 ( 0.62 ) | 1.71 ( 0.51 ) | 0.77 ( -1 ) |
| Vasculitis | 2 | 3.56 ( 0.78 - 16.23 ) | 3.56 ( 3.06 ) | 3.13 ( 0.88 ) | 1.65 ( -0.2 ) |
| Intestinal Obstruction | 2 | 1.11 ( 0.27 - 4.64 ) | 1.11 ( 0.02 ) | 1.1 ( 0.33 ) | 0.14 ( -1.59 ) |
| Abdominal Discomfort | 2 | 0.81 ( 0.2 - 3.33 ) | 0.81 ( 0.09 ) | 0.82 ( 0.25 ) | -0.29 ( -2.01 ) |
| Blood Calcium Increased | 2 | 2.96 ( 0.66 - 13.24 ) | 2.96 ( 2.23 ) | 2.68 ( 0.77 ) | 1.42 ( -0.4 ) |
| Blood Magnesium Increased | 2 | 5.08 ( 1.06 - 24.46 ) | 5.08 ( 5.1 ) | 4.17 ( 1.12 ) | 2.06 ( 0.16 ) |
| Blood Fibrinogen Decreased | 2 | 3.95 ( 0.85 - 18.29 ) | 3.95 ( 3.61 ) | 3.41 ( 0.95 ) | 1.77 ( -0.09 ) |
| Haemoglobin | 2 | 0.67 ( 0.16 - 2.75 ) | 0.67 ( 0.31 ) | 0.68 ( 0.21 ) | -0.55 ( -2.26 ) |
| Allergy To Vaccine | 2 | 1.23 ( 0.29 - 5.14 ) | 1.23 ( 0.08 ) | 1.21 ( 0.37 ) | 0.28 ( -1.47 ) |
| Nodule | 2 | 0.99 ( 0.24 - 4.1 ) | 0.99 ( 0 ) | 0.99 ( 0.3 ) | -0.02 ( -1.75 ) |
| Blood Albumin | 2 | 5.93 ( 1.2 - 29.37 ) | 5.93 ( 6.14 ) | 4.69 ( 1.23 ) | 2.23 ( 0.31 ) |
| Disturbance In Attention | 2 | 1.05 ( 0.25 - 4.35 ) | 1.05 ( 0 ) | 1.04 ( 0.32 ) | 0.06 ( -1.67 ) |
| Streptococcus Test Negative | 2 | 0.3 ( 0.07 - 1.2 ) | 0.3 ( 3.29 ) | 0.31 ( 0.1 ) | -1.7 ( -3.39 ) |
| Neurodevelopmental Disorder | 2 | 5.93 ( 1.2 - 29.37 ) | 5.93 ( 6.14 ) | 4.69 ( 1.23 ) | 2.23 ( 0.31 ) |
| Cytogenetic Analysis | 2 | 1.69 ( 0.4 - 7.22 ) | 1.69 ( 0.52 ) | 1.63 ( 0.49 ) | 0.71 ( -1.06 ) |
| Monoparesis | 2 | 3.23 ( 0.72 - 14.59 ) | 3.23 ( 2.61 ) | 2.89 ( 0.82 ) | 1.53 ( -0.31 ) |
| Monoplegia | 2 | 2.96 ( 0.66 - 13.24 ) | 2.96 ( 2.23 ) | 2.68 ( 0.77 ) | 1.42 ( -0.4 ) |
| Administration Site Swelling | 2 | 17.78 ( 2.5 - 126.26 ) | 17.78 ( 15.83 ) | 9.39 ( 1.82 ) | 3.23 ( 1.12 ) |
| Psychomotor Hyperactivity | 2 | 1.42 ( 0.34 - 6.01 ) | 1.42 ( 0.23 ) | 1.39 ( 0.42 ) | 0.48 ( -1.28 ) |
| Swelling Of Eyelid | 2 | 0.87 ( 0.21 - 3.59 ) | 0.87 ( 0.04 ) | 0.87 ( 0.27 ) | -0.2 ( -1.92 ) |
| Dyschezia | 2 | 3.23 ( 0.72 - 14.59 ) | 3.23 ( 2.61 ) | 2.89 ( 0.82 ) | 1.53 ( -0.31 ) |
| Coordination Abnormal | 2 | 1.27 ( 0.3 - 5.33 ) | 1.27 ( 0.11 ) | 1.25 ( 0.38 ) | 0.32 ( -1.42 ) |
| Disorientation | 2 | 2.22 ( 0.51 - 9.67 ) | 2.22 ( 1.2 ) | 2.09 ( 0.61 ) | 1.06 ( -0.73 ) |
| Gene Mutation Identification Test Negative | 2 | 35.56 ( 3.22 - 392.26 ) | 35.55 ( 22.39 ) | 12.52 ( 1.68 ) | 3.65 ( 1.44 ) |
| Autoimmune Disorder | 2 | 2.37 ( 0.54 - 10.37 ) | 2.37 ( 1.4 ) | 2.21 ( 0.64 ) | 1.14 ( -0.66 ) |
| Juvenile Idiopathic Arthritis | 2 | 3.56 ( 0.78 - 16.23 ) | 3.56 ( 3.06 ) | 3.13 ( 0.88 ) | 1.65 ( -0.2 ) |
| Meningitis Viral | 2 | 3.95 ( 0.85 - 18.29 ) | 3.95 ( 3.61 ) | 3.41 ( 0.95 ) | 1.77 ( -0.09 ) |
| Granuloma | 2 | 1.87 ( 0.44 - 8.04 ) | 1.87 ( 0.73 ) | 1.79 ( 0.53 ) | 0.84 ( -0.94 ) |
| Computerised Tomogram Thorax Abnormal | 2 | 2.22 ( 0.51 - 9.67 ) | 2.22 ( 1.2 ) | 2.09 ( 0.61 ) | 1.06 ( -0.73 ) |
| Bradypnoea | 2 | 35.56 ( 3.22 - 392.26 ) | 35.55 ( 22.39 ) | 12.52 ( 1.68 ) | 3.65 ( 1.44 ) |
| Developmental Regression | 2 | 0.36 ( 0.09 - 1.47 ) | 0.36 ( 2.2 ) | 0.38 ( 0.12 ) | -1.41 ( -3.1 ) |
| Skin Exfoliation | 2 | 0.87 ( 0.21 - 3.59 ) | 0.87 ( 0.04 ) | 0.87 ( 0.27 ) | -0.2 ( -1.92 ) |
| Barium Swallow | 2 | 8.89 ( 1.63 - 48.55 ) | 8.89 ( 9.33 ) | 6.26 ( 1.51 ) | 2.65 ( 0.65 ) |
| Musculoskeletal Disorder | 2 | 2.37 ( 0.54 - 10.37 ) | 2.37 ( 1.4 ) | 2.21 ( 0.64 ) | 1.14 ( -0.66 ) |
| Head Titubation | 2 | 2.09 ( 0.48 - 9.06 ) | 2.09 ( 1.02 ) | 1.98 ( 0.58 ) | 0.98 ( -0.8 ) |
| Rash Pruritic | 2 | 0.24 ( 0.06 - 0.97 ) | 0.24 ( 4.75 ) | 0.25 ( 0.08 ) | -2 ( -3.68 ) |
| Muscle Tone Disorder | 2 | 3.56 ( 0.78 - 16.23 ) | 3.56 ( 3.06 ) | 3.13 ( 0.88 ) | 1.65 ( -0.2 ) |
| Grunting | 2 | 0.87 ( 0.21 - 3.59 ) | 0.87 ( 0.04 ) | 0.87 ( 0.27 ) | -0.2 ( -1.92 ) |
| Transaminases Increased | 2 | 5.08 ( 1.06 - 24.46 ) | 5.08 ( 5.1 ) | 4.17 ( 1.12 ) | 2.06 ( 0.16 ) |
| Motor Developmental Delay | 2 | 2.22 ( 0.51 - 9.67 ) | 2.22 ( 1.2 ) | 2.09 ( 0.61 ) | 1.06 ( -0.73 ) |
| Allergy Test Negative | 2 | 4.45 ( 0.94 - 20.94 ) | 4.44 ( 4.27 ) | 3.76 ( 1.03 ) | 1.91 ( 0.03 ) |
| Clonic Convulsion | 2 | 3.56 ( 0.78 - 16.23 ) | 3.56 ( 3.06 ) | 3.13 ( 0.88 ) | 1.65 ( -0.2 ) |
| Influenza A Virus Test Negative | 2 | 0.54 ( 0.13 - 2.2 ) | 0.54 ( 0.77 ) | 0.55 ( 0.17 ) | -0.86 ( -2.56 ) |
| Influenza B Virus Test Negative | 2 | 4.45 ( 0.94 - 20.94 ) | 4.44 ( 4.27 ) | 3.76 ( 1.03 ) | 1.91 ( 0.03 ) |
| Infantile Diarrhoea | 2 | 1.98 ( 0.46 - 8.52 ) | 1.98 ( 0.87 ) | 1.88 ( 0.55 ) | 0.91 ( -0.87 ) |
| Enterovirus Test Positive | 2 | 0.96 ( 0.23 - 3.99 ) | 0.96 ( 0 ) | 0.96 ( 0.29 ) | -0.05 ( -1.78 ) |
| Deafness | 2 | 2.09 ( 0.48 - 9.06 ) | 2.09 ( 1.02 ) | 1.98 ( 0.58 ) | 0.98 ( -0.8 ) |
| Strabismus | 2 | 0.63 ( 0.15 - 2.6 ) | 0.63 ( 0.41 ) | 0.65 ( 0.2 ) | -0.63 ( -2.34 ) |
| Lip Swelling | 2 | 0.53 ( 0.13 - 2.17 ) | 0.53 ( 0.81 ) | 0.54 ( 0.17 ) | -0.88 ( -2.58 ) |
| Metabolic Disorder | 2 | 3.56 ( 0.78 - 16.23 ) | 3.56 ( 3.06 ) | 3.13 ( 0.88 ) | 1.65 ( -0.2 ) |
| Gastrointestinal Necrosis | 2 | 3.56 ( 0.78 - 16.23 ) | 3.56 ( 3.06 ) | 3.13 ( 0.88 ) | 1.65 ( -0.2 ) |
| Acute Kidney Injury | 2 | 3.56 ( 0.78 - 16.23 ) | 3.56 ( 3.06 ) | 3.13 ( 0.88 ) | 1.65 ( -0.2 ) |
| Oedema | 2 | 0.68 ( 0.17 - 2.81 ) | 0.68 ( 0.28 ) | 0.7 ( 0.21 ) | -0.52 ( -2.24 ) |
| Procalcitonin Increased | 2 | 0.77 ( 0.19 - 3.18 ) | 0.77 ( 0.13 ) | 0.78 ( 0.24 ) | -0.35 ( -2.07 ) |
| Eosinophil Percentage | 2 | 1.19 ( 0.28 - 4.96 ) | 1.19 ( 0.05 ) | 1.17 ( 0.35 ) | 0.23 ( -1.51 ) |
| Muscle Contracture | 2 | 8.89 ( 1.63 - 48.55 ) | 8.89 ( 9.33 ) | 6.26 ( 1.51 ) | 2.65 ( 0.65 ) |
| Injection Site Discharge | 2 | 1.78 ( 0.42 - 7.61 ) | 1.78 ( 0.62 ) | 1.71 ( 0.51 ) | 0.77 ( -1 ) |
| Bacterial Test Negative | 2 | 0.52 ( 0.13 - 2.13 ) | 0.52 ( 0.85 ) | 0.54 ( 0.17 ) | -0.9 ( -2.6 ) |
| Ear Disorder | 2 | 8.89 ( 1.63 - 48.55 ) | 8.89 ( 9.33 ) | 6.26 ( 1.51 ) | 2.65 ( 0.65 ) |
| Respiratory Disorder | 2 | 1.08 ( 0.26 - 4.49 ) | 1.08 ( 0.01 ) | 1.07 ( 0.32 ) | 0.1 ( -1.63 ) |
| Extensive Swelling Of Vaccinated Limb | 2 | 0.74 ( 0.18 - 3.05 ) | 0.74 ( 0.17 ) | 0.75 ( 0.23 ) | -0.41 ( -2.13 ) |
| Abdominal Distension | 2 | 0.5 ( 0.12 - 2.04 ) | 0.5 ( 0.97 ) | 0.51 ( 0.16 ) | -0.96 ( -2.66 ) |
| Red Cell Distribution Width Increased | 2 | 1.62 ( 0.38 - 6.87 ) | 1.62 ( 0.43 ) | 1.56 ( 0.47 ) | 0.65 ( -1.12 ) |
| Lymphocyte Count Increased | 2 | 0.51 ( 0.12 - 2.07 ) | 0.51 ( 0.93 ) | 0.52 ( 0.16 ) | -0.94 ( -2.64 ) |
| Blood Immunoglobulin M | 2 | 1.55 ( 0.36 - 6.56 ) | 1.55 ( 0.35 ) | 1.5 ( 0.45 ) | 0.59 ( -1.17 ) |
| Mean Cell Haemoglobin Decreased | 2 | 0.66 ( 0.16 - 2.7 ) | 0.66 ( 0.34 ) | 0.67 ( 0.21 ) | -0.58 ( -2.29 ) |
| Mean Cell Volume Decreased | 2 | 0.54 ( 0.13 - 2.2 ) | 0.54 ( 0.77 ) | 0.55 ( 0.17 ) | -0.86 ( -2.56 ) |
| Mydriasis | 2 | 5.08 ( 1.06 - 24.46 ) | 5.08 ( 5.1 ) | 4.17 ( 1.12 ) | 2.06 ( 0.16 ) |
| Influenza | 2 | 0.65 ( 0.16 - 2.65 ) | 0.65 ( 0.37 ) | 0.66 ( 0.2 ) | -0.6 ( -2.31 ) |
| Injection Site Abscess | 2 | 0.76 ( 0.18 - 3.11 ) | 0.76 ( 0.15 ) | 0.77 ( 0.23 ) | -0.38 ( -2.1 ) |
| Gastrointestinal Inflammation | 2 | 7.11 ( 1.38 - 36.67 ) | 7.11 ( 7.5 ) | 5.36 ( 1.36 ) | 2.42 ( 0.47 ) |
| Lymphadenitis | 2 | 1.69 ( 0.4 - 7.22 ) | 1.69 ( 0.52 ) | 1.63 ( 0.49 ) | 0.71 ( -1.06 ) |
| Staphylococcal Infection | 2 | 2.22 ( 0.51 - 9.67 ) | 2.22 ( 1.2 ) | 2.09 ( 0.61 ) | 1.06 ( -0.73 ) |
| Food Refusal | 2 | 0.83 ( 0.2 - 3.41 ) | 0.83 ( 0.07 ) | 0.83 ( 0.25 ) | -0.26 ( -1.98 ) |
| Enteritis | 2 | 1.15 ( 0.27 - 4.79 ) | 1.15 ( 0.04 ) | 1.14 ( 0.34 ) | 0.19 ( -1.55 ) |
| Encephalitis | 2 | 0.46 ( 0.11 - 1.88 ) | 0.46 ( 1.22 ) | 0.48 ( 0.15 ) | -1.07 ( -2.77 ) |
| Personality Change | 2 | 0.99 ( 0.24 - 4.1 ) | 0.99 ( 0 ) | 0.99 ( 0.3 ) | -0.02 ( -1.75 ) |
| Candida Infection | 2 | 3.56 ( 0.78 - 16.23 ) | 3.56 ( 3.06 ) | 3.13 ( 0.88 ) | 1.65 ( -0.2 ) |
| Gastritis | 2 | 5.08 ( 1.06 - 24.46 ) | 5.08 ( 5.1 ) | 4.17 ( 1.12 ) | 2.06 ( 0.16 ) |
| Globulin | 2 | 3.56 ( 0.78 - 16.23 ) | 3.56 ( 3.06 ) | 3.13 ( 0.88 ) | 1.65 ( -0.2 ) |
| Supraventricular Tachycardia | 2 | 3.56 ( 0.78 - 16.23 ) | 3.56 ( 3.06 ) | 3.13 ( 0.88 ) | 1.65 ( -0.2 ) |
| Abscess Limb | 2 | 8.89 ( 1.63 - 48.55 ) | 8.89 ( 9.33 ) | 6.26 ( 1.51 ) | 2.65 ( 0.65 ) |
| Mood Altered | 2 | 0.3 ( 0.07 - 1.21 ) | 0.3 ( 3.24 ) | 0.31 ( 0.1 ) | -1.69 ( -3.37 ) |
| Liver Function Test Increased | 2 | 5.08 ( 1.06 - 24.46 ) | 5.08 ( 5.1 ) | 4.17 ( 1.12 ) | 2.06 ( 0.16 ) |
| Base Excess Increased | 2 | 35.56 ( 3.22 - 392.26 ) | 35.55 ( 22.39 ) | 12.52 ( 1.68 ) | 3.65 ( 1.44 ) |
| Hypokinesia | 2 | 1.23 ( 0.29 - 5.14 ) | 1.23 ( 0.08 ) | 1.21 ( 0.37 ) | 0.28 ( -1.47 ) |
| Protein Total Increased | 2 | 4.45 ( 0.94 - 20.94 ) | 4.44 ( 4.27 ) | 3.76 ( 1.03 ) | 1.91 ( 0.03 ) |
| Vaccination Site Granuloma | 2 | 3.95 ( 0.85 - 18.29 ) | 3.95 ( 3.61 ) | 3.41 ( 0.95 ) | 1.77 ( -0.09 ) |
| Capillary Nail Refill Test Abnormal | 2 | 5.08 ( 1.06 - 24.46 ) | 5.08 ( 5.1 ) | 4.17 ( 1.12 ) | 2.06 ( 0.16 ) |
| Meningitis Pneumococcal | 2 | 2.74 ( 0.62 - 12.12 ) | 2.73 ( 1.91 ) | 2.5 ( 0.72 ) | 1.32 ( -0.49 ) |
| Immune System Disorder | 2 | 1.69 ( 0.4 - 7.22 ) | 1.69 ( 0.52 ) | 1.63 ( 0.49 ) | 0.71 ( -1.06 ) |
| Infection | 2 | 0.37 ( 0.09 - 1.5 ) | 0.37 ( 2.1 ) | 0.38 ( 0.12 ) | -1.38 ( -3.08 ) |
| Autoimmune Neutropenia | 2 | 11.85 ( 1.98 - 70.96 ) | 11.85 ( 11.92 ) | 7.51 ( 1.68 ) | 2.91 ( 0.87 ) |
| Body Temperature Abnormal | 2 | 5.93 ( 1.2 - 29.37 ) | 5.93 ( 6.14 ) | 4.69 ( 1.23 ) | 2.23 ( 0.31 ) |
| Eosinophil Count Increased | 2 | 1.78 ( 0.42 - 7.61 ) | 1.78 ( 0.62 ) | 1.71 ( 0.51 ) | 0.77 ( -1 ) |
| Lumbar Puncture Abnormal | 2 | 2.09 ( 0.48 - 9.06 ) | 2.09 ( 1.02 ) | 1.98 ( 0.58 ) | 0.98 ( -0.8 ) |
| Blood Potassium Decreased | 2 | 2.09 ( 0.48 - 9.06 ) | 2.09 ( 1.02 ) | 1.98 ( 0.58 ) | 0.98 ( -0.8 ) |
| Akathisia | 2 | 35.56 ( 3.22 - 392.26 ) | 35.55 ( 22.39 ) | 12.52 ( 1.68 ) | 3.65 ( 1.44 ) |
| Tonic Convulsion | 2 | 1.69 ( 0.4 - 7.22 ) | 1.69 ( 0.52 ) | 1.63 ( 0.49 ) | 0.71 ( -1.06 ) |
| Myoclonic Epilepsy | 2 | 3.95 ( 0.85 - 18.29 ) | 3.95 ( 3.61 ) | 3.41 ( 0.95 ) | 1.77 ( -0.09 ) |
| Dysphemia | 2 | 2.22 ( 0.51 - 9.67 ) | 2.22 ( 1.2 ) | 2.09 ( 0.61 ) | 1.06 ( -0.73 ) |
| Antibody Test Negative | 2 | 1.27 ( 0.3 - 5.33 ) | 1.27 ( 0.11 ) | 1.25 ( 0.38 ) | 0.32 ( -1.42 ) |
| Neutrophil Percentage Increased | 2 | 1.11 ( 0.27 - 4.64 ) | 1.11 ( 0.02 ) | 1.1 ( 0.33 ) | 0.14 ( -1.59 ) |
| Acute Respiratory Failure | 2 | 1.69 ( 0.4 - 7.22 ) | 1.69 ( 0.52 ) | 1.63 ( 0.49 ) | 0.71 ( -1.06 ) |
| Scan Abnormal | 1 | 17.78 ( 1.11 - 284.29 ) | 17.78 ( 7.92 ) | 9.39 ( 0.92 ) | 3.23 ( 0.6 ) |
| Infant Sedation | 1 | 17.78 ( 1.11 - 284.29 ) | 17.78 ( 7.92 ) | 9.39 ( 0.92 ) | 3.23 ( 0.6 ) |
| Growth Failure | 1 | 8.89 ( 0.81 - 98.05 ) | 8.89 ( 4.67 ) | 6.26 ( 0.84 ) | 2.65 ( 0.15 ) |
| Anaemia | 1 | 0.26 ( 0.04 - 1.88 ) | 0.26 ( 2.06 ) | 0.27 ( 0.05 ) | -1.88 ( -3.95 ) |
| B Precursor Type Acute Leukaemia | 1 | 8.89 ( 0.81 - 98.05 ) | 8.89 ( 4.67 ) | 6.26 ( 0.84 ) | 2.65 ( 0.15 ) |
| Pancytopenia | 1 | 1.48 ( 0.19 - 11.4 ) | 1.48 ( 0.14 ) | 1.44 ( 0.26 ) | 0.53 ( -1.65 ) |
| Blood Immunoglobulin E Increased | 1 | 4.44 ( 0.5 - 39.77 ) | 4.44 ( 2.14 ) | 3.76 ( 0.6 ) | 1.91 ( -0.45 ) |
| Meningococcal Infection | 1 | 2.54 ( 0.31 - 20.65 ) | 2.54 ( 0.82 ) | 2.35 ( 0.41 ) | 1.23 ( -1.02 ) |
| Meningococcal Sepsis | 1 | 4.44 ( 0.5 - 39.77 ) | 4.44 ( 2.14 ) | 3.76 ( 0.6 ) | 1.91 ( -0.45 ) |
| Hypovolaemia | 1 | 8.89 ( 0.81 - 98.05 ) | 8.89 ( 4.67 ) | 6.26 ( 0.84 ) | 2.65 ( 0.15 ) |
| Breath Sounds Abnormal | 1 | 0.27 ( 0.04 - 1.91 ) | 0.27 ( 2.01 ) | 0.28 ( 0.05 ) | -1.86 ( -3.93 ) |
| Cerebral Palsy | 1 | 4.44 ( 0.5 - 39.77 ) | 4.44 ( 2.14 ) | 3.76 ( 0.6 ) | 1.91 ( -0.45 ) |
| Base Excess | 1 | 1.48 ( 0.19 - 11.4 ) | 1.48 ( 0.14 ) | 1.44 ( 0.26 ) | 0.53 ( -1.65 ) |
| Ph Body Fluid | 1 | 5.93 ( 0.62 - 56.98 ) | 5.93 ( 3.07 ) | 4.69 ( 0.71 ) | 2.23 ( -0.18 ) |
| Emotional Disorder | 1 | 1.05 ( 0.14 - 7.86 ) | 1.05 ( 0 ) | 1.04 ( 0.19 ) | 0.06 ( -2.08 ) |
| Cerebral Infarction | 1 | 1.62 ( 0.21 - 12.52 ) | 1.62 ( 0.22 ) | 1.56 ( 0.28 ) | 0.65 ( -1.55 ) |
| Cataract | 1 | 17.78 ( 1.11 - 284.29 ) | 17.78 ( 7.92 ) | 9.39 ( 0.92 ) | 3.23 ( 0.6 ) |
| Joint Hyperextension | 1 | 8.89 ( 0.81 - 98.05 ) | 8.89 ( 4.67 ) | 6.26 ( 0.84 ) | 2.65 ( 0.15 ) |
| Dysbiosis | 1 | 1.98 ( 0.25 - 15.59 ) | 1.98 ( 0.43 ) | 1.88 ( 0.33 ) | 0.91 ( -1.31 ) |
| Malabsorption | 1 | 17.78 ( 1.11 - 284.29 ) | 17.78 ( 7.92 ) | 9.39 ( 0.92 ) | 3.23 ( 0.6 ) |
| Viral Test Positive | 1 | 0.99 ( 0.13 - 7.4 ) | 0.99 ( 0 ) | 0.99 ( 0.18 ) | -0.02 ( -2.16 ) |
| Palatal Oedema | 1 | 17.78 ( 1.11 - 284.29 ) | 17.78 ( 7.92 ) | 9.39 ( 0.92 ) | 3.23 ( 0.6 ) |
| Vaccination Site Warmth | 1 | 0.33 ( 0.05 - 2.38 ) | 0.33 ( 1.34 ) | 0.34 ( 0.07 ) | -1.55 ( -3.63 ) |
| Cytomegalovirus Test Positive | 1 | 0.74 ( 0.1 - 5.48 ) | 0.74 ( 0.09 ) | 0.75 ( 0.14 ) | -0.41 ( -2.53 ) |
| Functional Gastrointestinal Disorder | 1 | 2.54 ( 0.31 - 20.65 ) | 2.54 ( 0.82 ) | 2.35 ( 0.41 ) | 1.23 ( -1.02 ) |
| Neisseria Test Negative | 1 | 17.78 ( 1.11 - 284.29 ) | 17.78 ( 7.92 ) | 9.39 ( 0.92 ) | 3.23 ( 0.6 ) |
| Occult Blood Positive | 1 | 0.21 ( 0.03 - 1.48 ) | 0.21 ( 3.01 ) | 0.22 ( 0.04 ) | -2.21 ( -4.28 ) |
| Drug Hypersensitivity | 1 | 1.48 ( 0.19 - 11.4 ) | 1.48 ( 0.14 ) | 1.44 ( 0.26 ) | 0.53 ( -1.65 ) |
| Exophthalmos | 1 | 17.78 ( 1.11 - 284.29 ) | 17.78 ( 7.92 ) | 9.39 ( 0.92 ) | 3.23 ( 0.6 ) |
| Erythema Of Eyelid | 1 | 0.94 ( 0.13 - 6.99 ) | 0.94 ( 0 ) | 0.94 ( 0.17 ) | -0.09 ( -2.23 ) |
| Illusion | 1 | 5.93 ( 0.62 - 56.98 ) | 5.93 ( 3.07 ) | 4.69 ( 0.71 ) | 2.23 ( -0.18 ) |
| Hypogammaglobulinaemia | 1 | 2.96 ( 0.36 - 24.62 ) | 2.96 ( 1.11 ) | 2.68 ( 0.46 ) | 1.42 ( -0.86 ) |
| Food Protein-Induced Enterocolitis Syndrome | 1 | 8.89 ( 0.81 - 98.05 ) | 8.89 ( 4.67 ) | 6.26 ( 0.84 ) | 2.65 ( 0.15 ) |
| Neurological Symptom | 1 | 0.85 ( 0.11 - 6.29 ) | 0.85 ( 0.03 ) | 0.85 ( 0.16 ) | -0.23 ( -2.36 ) |
| Grimacing | 1 | 1.98 ( 0.25 - 15.59 ) | 1.98 ( 0.43 ) | 1.88 ( 0.33 ) | 0.91 ( -1.31 ) |
| Muscle Contractions Involuntary | 1 | 1.98 ( 0.25 - 15.59 ) | 1.98 ( 0.43 ) | 1.88 ( 0.33 ) | 0.91 ( -1.31 ) |
| Soft Tissue Swelling | 1 | 2.22 ( 0.28 - 17.77 ) | 2.22 ( 0.6 ) | 2.09 ( 0.37 ) | 1.06 ( -1.17 ) |
| Conjunctivitis | 1 | 0.23 ( 0.03 - 1.64 ) | 0.23 ( 2.58 ) | 0.24 ( 0.05 ) | -2.07 ( -4.14 ) |
| Iron Deficiency | 1 | 2.96 ( 0.36 - 24.62 ) | 2.96 ( 1.11 ) | 2.68 ( 0.46 ) | 1.42 ( -0.86 ) |
| Tonsillitis | 1 | 0.32 ( 0.04 - 2.34 ) | 0.32 ( 1.39 ) | 0.34 ( 0.06 ) | -1.58 ( -3.65 ) |
| Polyp | 1 | 17.78 ( 1.11 - 284.29 ) | 17.78 ( 7.92 ) | 9.39 ( 0.92 ) | 3.23 ( 0.6 ) |
| Febrile Infection | 1 | 4.44 ( 0.5 - 39.77 ) | 4.44 ( 2.14 ) | 3.76 ( 0.6 ) | 1.91 ( -0.45 ) |
| Hypoacusis | 1 | 2.22 ( 0.28 - 17.77 ) | 2.22 ( 0.6 ) | 2.09 ( 0.37 ) | 1.06 ( -1.17 ) |
| Pustule | 1 | 0.77 ( 0.1 - 5.72 ) | 0.77 ( 0.06 ) | 0.78 ( 0.15 ) | -0.35 ( -2.47 ) |
| Rotavirus Infection | 1 | 0.13 ( 0.02 - 0.91 ) | 0.13 ( 5.97 ) | 0.13 ( 0.03 ) | -2.91 ( -4.96 ) |
| Dissociation | 1 | 17.78 ( 1.11 - 284.29 ) | 17.78 ( 7.92 ) | 9.39 ( 0.92 ) | 3.23 ( 0.6 ) |
| Blood Iron Decreased | 1 | 2.22 ( 0.28 - 17.77 ) | 2.22 ( 0.6 ) | 2.09 ( 0.37 ) | 1.06 ( -1.17 ) |
| Pneumonia Viral | 1 | 1.62 ( 0.21 - 12.52 ) | 1.62 ( 0.22 ) | 1.56 ( 0.28 ) | 0.65 ( -1.55 ) |
| Joint Contracture | 1 | 2.96 ( 0.36 - 24.62 ) | 2.96 ( 1.11 ) | 2.68 ( 0.46 ) | 1.42 ( -0.86 ) |
| Coagulopathy | 1 | 3.56 ( 0.42 - 30.44 ) | 3.56 ( 1.53 ) | 3.13 ( 0.52 ) | 1.65 ( -0.67 ) |
| Immune Thrombocytopenia | 1 | 0.14 ( 0.02 - 1.01 ) | 0.14 ( 5.19 ) | 0.15 ( 0.03 ) | -2.76 ( -4.81 ) |
| Thrombocytopenic Purpura | 1 | 0.08 ( 0.01 - 0.59 ) | 0.08 ( 10.09 ) | 0.09 ( 0.02 ) | -3.52 ( -5.57 ) |
| Bradycardia Neonatal | 1 | 8.89 ( 0.81 - 98.05 ) | 8.89 ( 4.67 ) | 6.26 ( 0.84 ) | 2.65 ( 0.15 ) |
| Eructation | 1 | 2.96 ( 0.36 - 24.62 ) | 2.96 ( 1.11 ) | 2.68 ( 0.46 ) | 1.42 ( -0.86 ) |
| Blood Bicarbonate Increased | 1 | 4.44 ( 0.5 - 39.77 ) | 4.44 ( 2.14 ) | 3.76 ( 0.6 ) | 1.91 ( -0.45 ) |
| Lip Discolouration | 1 | 0.74 ( 0.1 - 5.48 ) | 0.74 ( 0.09 ) | 0.75 ( 0.14 ) | -0.41 ( -2.53 ) |
| Urine Ketone Body Present | 1 | 0.99 ( 0.13 - 7.4 ) | 0.99 ( 0 ) | 0.99 ( 0.18 ) | -0.02 ( -2.16 ) |
| Faecal Volume Increased | 1 | 2.96 ( 0.36 - 24.62 ) | 2.96 ( 1.11 ) | 2.68 ( 0.46 ) | 1.42 ( -0.86 ) |
| Hyperthermia | 1 | 0.81 ( 0.11 - 6 ) | 0.81 ( 0.04 ) | 0.82 ( 0.15 ) | -0.29 ( -2.42 ) |
| Weight Bearing Difficulty | 1 | 0.26 ( 0.04 - 1.88 ) | 0.26 ( 2.06 ) | 0.27 ( 0.05 ) | -1.88 ( -3.95 ) |
| Morose | 1 | 5.93 ( 0.62 - 56.98 ) | 5.93 ( 3.07 ) | 4.69 ( 0.71 ) | 2.23 ( -0.18 ) |
| Giardia Test Negative | 1 | 5.93 ( 0.62 - 56.98 ) | 5.93 ( 3.07 ) | 4.69 ( 0.71 ) | 2.23 ( -0.18 ) |
| Infrequent Bowel Movements | 1 | 2.54 ( 0.31 - 20.65 ) | 2.54 ( 0.82 ) | 2.35 ( 0.41 ) | 1.23 ( -1.02 ) |
| Skin Laceration | 1 | 5.93 ( 0.62 - 56.98 ) | 5.93 ( 3.07 ) | 4.69 ( 0.71 ) | 2.23 ( -0.18 ) |
| Joint Swelling | 1 | 0.43 ( 0.06 - 3.15 ) | 0.43 ( 0.72 ) | 0.45 ( 0.09 ) | -1.16 ( -3.25 ) |
| Pneumococcal Infection | 1 | 0.81 ( 0.11 - 6 ) | 0.81 ( 0.04 ) | 0.82 ( 0.15 ) | -0.29 ( -2.42 ) |
| Inability To Crawl | 1 | 0.74 ( 0.1 - 5.48 ) | 0.74 ( 0.09 ) | 0.75 ( 0.14 ) | -0.41 ( -2.53 ) |
| Extrasystoles | 1 | 17.78 ( 1.11 - 284.29 ) | 17.78 ( 7.92 ) | 9.39 ( 0.92 ) | 3.23 ( 0.6 ) |
| Acute Lymphocytic Leukaemia | 1 | 2.54 ( 0.31 - 20.65 ) | 2.54 ( 0.82 ) | 2.35 ( 0.41 ) | 1.23 ( -1.02 ) |
| Blood Creatinine Decreased | 1 | 0.46 ( 0.06 - 3.32 ) | 0.46 ( 0.63 ) | 0.47 ( 0.09 ) | -1.09 ( -3.18 ) |
| Circulatory Collapse | 1 | 0.99 ( 0.13 - 7.4 ) | 0.99 ( 0 ) | 0.99 ( 0.18 ) | -0.02 ( -2.16 ) |
| Brain Death | 1 | 2.22 ( 0.28 - 17.77 ) | 2.22 ( 0.6 ) | 2.09 ( 0.37 ) | 1.06 ( -1.17 ) |
| Moraxella Test Positive | 1 | 17.78 ( 1.11 - 284.29 ) | 17.78 ( 7.92 ) | 9.39 ( 0.92 ) | 3.23 ( 0.6 ) |
| Blood Culture Positive | 1 | 1.11 ( 0.15 - 8.38 ) | 1.11 ( 0.01 ) | 1.1 ( 0.2 ) | 0.14 ( -2.01 ) |
| Vaccination Site Cellulitis | 1 | 0.94 ( 0.13 - 6.99 ) | 0.94 ( 0 ) | 0.94 ( 0.17 ) | -0.09 ( -2.23 ) |
| Rhinovirus Infection | 1 | 0.68 ( 0.09 - 5.04 ) | 0.68 ( 0.14 ) | 0.7 ( 0.13 ) | -0.52 ( -2.64 ) |
| Computerised Tomogram Head Abnormal | 1 | 0.85 ( 0.11 - 6.29 ) | 0.85 ( 0.03 ) | 0.85 ( 0.16 ) | -0.23 ( -2.36 ) |
| Cataplexy | 1 | 5.93 ( 0.62 - 56.98 ) | 5.93 ( 3.07 ) | 4.69 ( 0.71 ) | 2.23 ( -0.18 ) |
| Coronavirus Test Positive | 1 | 2.54 ( 0.31 - 20.65 ) | 2.54 ( 0.82 ) | 2.35 ( 0.41 ) | 1.23 ( -1.02 ) |
| Shock Symptom | 1 | 5.93 ( 0.62 - 56.98 ) | 5.93 ( 3.07 ) | 4.69 ( 0.71 ) | 2.23 ( -0.18 ) |
| Cerebral Cyst | 1 | 8.89 ( 0.81 - 98.05 ) | 8.89 ( 4.67 ) | 6.26 ( 0.84 ) | 2.65 ( 0.15 ) |
| Stress Urinary Incontinence | 1 | 17.78 ( 1.11 - 284.29 ) | 17.78 ( 7.92 ) | 9.39 ( 0.92 ) | 3.23 ( 0.6 ) |
| Ultrasound Head Abnormal | 1 | 4.44 ( 0.5 - 39.77 ) | 4.44 ( 2.14 ) | 3.76 ( 0.6 ) | 1.91 ( -0.45 ) |
| Sinus Tachycardia | 1 | 2.22 ( 0.28 - 17.77 ) | 2.22 ( 0.6 ) | 2.09 ( 0.37 ) | 1.06 ( -1.17 ) |
| Histamine Intolerance | 1 | 8.89 ( 0.81 - 98.05 ) | 8.89 ( 4.67 ) | 6.26 ( 0.84 ) | 2.65 ( 0.15 ) |
| Gamma-Glutamyltransferase Increased | 1 | 1.27 ( 0.17 - 9.66 ) | 1.27 ( 0.05 ) | 1.25 ( 0.23 ) | 0.32 ( -1.84 ) |
| Electrocardiogram Qt Interval | 1 | 17.78 ( 1.11 - 284.29 ) | 17.78 ( 7.92 ) | 9.39 ( 0.92 ) | 3.23 ( 0.6 ) |
| Clostridium Test Negative | 1 | 2.22 ( 0.28 - 17.77 ) | 2.22 ( 0.6 ) | 2.09 ( 0.37 ) | 1.06 ( -1.17 ) |
| Hypertransaminasaemia | 1 | 2.96 ( 0.36 - 24.62 ) | 2.96 ( 1.11 ) | 2.68 ( 0.46 ) | 1.42 ( -0.86 ) |
| Tri-Iodothyronine Free | 1 | 17.78 ( 1.11 - 284.29 ) | 17.78 ( 7.92 ) | 9.39 ( 0.92 ) | 3.23 ( 0.6 ) |
| Reduced Facial Expression | 1 | 1.78 ( 0.23 - 13.89 ) | 1.78 ( 0.31 ) | 1.71 ( 0.31 ) | 0.77 ( -1.43 ) |
| Aspiration | 1 | 0.99 ( 0.13 - 7.4 ) | 0.99 ( 0 ) | 0.99 ( 0.18 ) | -0.02 ( -2.16 ) |
| Investigation Abnormal | 1 | 17.78 ( 1.11 - 284.29 ) | 17.78 ( 7.92 ) | 9.39 ( 0.92 ) | 3.23 ( 0.6 ) |
| Keratosis Pilaris | 1 | 3.56 ( 0.42 - 30.44 ) | 3.56 ( 1.53 ) | 3.13 ( 0.52 ) | 1.65 ( -0.67 ) |
| Anxiety Disorder | 1 | 8.89 ( 0.81 - 98.05 ) | 8.89 ( 4.67 ) | 6.26 ( 0.84 ) | 2.65 ( 0.15 ) |
| Injection Site Haematoma | 1 | 1.11 ( 0.15 - 8.38 ) | 1.11 ( 0.01 ) | 1.1 ( 0.2 ) | 0.14 ( -2.01 ) |
| Septic Shock | 1 | 1.48 ( 0.19 - 11.4 ) | 1.48 ( 0.14 ) | 1.44 ( 0.26 ) | 0.53 ( -1.65 ) |
| Coma Scale Abnormal | 1 | 3.56 ( 0.42 - 30.44 ) | 3.56 ( 1.53 ) | 3.13 ( 0.52 ) | 1.65 ( -0.67 ) |
| Neurological Decompensation | 1 | 5.93 ( 0.62 - 56.98 ) | 5.93 ( 3.07 ) | 4.69 ( 0.71 ) | 2.23 ( -0.18 ) |
| Administration Site Erythema | 1 | 3.56 ( 0.42 - 30.44 ) | 3.56 ( 1.53 ) | 3.13 ( 0.52 ) | 1.65 ( -0.67 ) |
| Obstructive Airways Disorder | 1 | 1.98 ( 0.25 - 15.59 ) | 1.98 ( 0.43 ) | 1.88 ( 0.33 ) | 0.91 ( -1.31 ) |
| Blood Magnesium Decreased | 1 | 2.96 ( 0.36 - 24.62 ) | 2.96 ( 1.11 ) | 2.68 ( 0.46 ) | 1.42 ( -0.86 ) |
| Blood Osmolarity | 1 | 4.44 ( 0.5 - 39.77 ) | 4.44 ( 2.14 ) | 3.76 ( 0.6 ) | 1.91 ( -0.45 ) |
| Blood Phosphorus Decreased | 1 | 2.54 ( 0.31 - 20.65 ) | 2.54 ( 0.82 ) | 2.35 ( 0.41 ) | 1.23 ( -1.02 ) |
| Type 1 Diabetes Mellitus | 1 | 0.77 ( 0.1 - 5.72 ) | 0.77 ( 0.06 ) | 0.78 ( 0.15 ) | -0.35 ( -2.47 ) |
| Injection Site Extravasation | 1 | 0.94 ( 0.13 - 6.99 ) | 0.94 ( 0 ) | 0.94 ( 0.17 ) | -0.09 ( -2.23 ) |
| Infection Susceptibility Increased | 1 | 1.19 ( 0.16 - 8.97 ) | 1.19 ( 0.03 ) | 1.17 ( 0.22 ) | 0.23 ( -1.93 ) |
| Balance Disorder | 1 | 0.21 ( 0.03 - 1.52 ) | 0.21 ( 2.9 ) | 0.22 ( 0.04 ) | -2.18 ( -4.24 ) |
| Neutrophilia | 1 | 1.37 ( 0.18 - 10.46 ) | 1.37 ( 0.09 ) | 1.34 ( 0.24 ) | 0.42 ( -1.75 ) |
| Catarrh | 1 | 0.57 ( 0.08 - 4.2 ) | 0.57 ( 0.31 ) | 0.59 ( 0.11 ) | -0.77 ( -2.87 ) |
| Jaundice | 1 | 1.27 ( 0.17 - 9.66 ) | 1.27 ( 0.05 ) | 1.25 ( 0.23 ) | 0.32 ( -1.84 ) |
| Red Blood Cell Abnormality | 1 | 17.78 ( 1.11 - 284.29 ) | 17.78 ( 7.92 ) | 9.39 ( 0.92 ) | 3.23 ( 0.6 ) |
| Soft Tissue Disorder | 1 | 4.44 ( 0.5 - 39.77 ) | 4.44 ( 2.14 ) | 3.76 ( 0.6 ) | 1.91 ( -0.45 ) |
| Haemophagocytic Lymphohistiocytosis | 1 | 0.94 ( 0.13 - 6.99 ) | 0.94 ( 0 ) | 0.94 ( 0.17 ) | -0.09 ( -2.23 ) |
| Splenomegaly | 1 | 1.78 ( 0.23 - 13.89 ) | 1.78 ( 0.31 ) | 1.71 ( 0.31 ) | 0.77 ( -1.43 ) |
| B-Lymphocyte Count Increased | 1 | 17.78 ( 1.11 - 284.29 ) | 17.78 ( 7.92 ) | 9.39 ( 0.92 ) | 3.23 ( 0.6 ) |
| Hypoalbuminaemia | 1 | 5.93 ( 0.62 - 56.98 ) | 5.93 ( 3.07 ) | 4.69 ( 0.71 ) | 2.23 ( -0.18 ) |
| Coronary Artery Disease | 1 | 17.78 ( 1.11 - 284.29 ) | 17.78 ( 7.92 ) | 9.39 ( 0.92 ) | 3.23 ( 0.6 ) |
| Hypofibrinogenaemia | 1 | 17.78 ( 1.11 - 284.29 ) | 17.78 ( 7.92 ) | 9.39 ( 0.92 ) | 3.23 ( 0.6 ) |
| Serum Ferritin Increased | 1 | 1.78 ( 0.23 - 13.89 ) | 1.78 ( 0.31 ) | 1.71 ( 0.31 ) | 0.77 ( -1.43 ) |
| Perioral Dermatitis | 1 | 4.44 ( 0.5 - 39.77 ) | 4.44 ( 2.14 ) | 3.76 ( 0.6 ) | 1.91 ( -0.45 ) |
| Meningitis Bacterial | 1 | 0.99 ( 0.13 - 7.4 ) | 0.99 ( 0 ) | 0.99 ( 0.18 ) | -0.02 ( -2.16 ) |
| Acute Haemorrhagic Oedema Of Infancy | 1 | 2.54 ( 0.31 - 20.65 ) | 2.54 ( 0.82 ) | 2.35 ( 0.41 ) | 1.23 ( -1.02 ) |
| Mental Status Changes | 1 | 0.63 ( 0.09 - 4.67 ) | 0.63 ( 0.2 ) | 0.65 ( 0.12 ) | -0.63 ( -2.73 ) |
| Sleep Apnoea Syndrome | 1 | 1.37 ( 0.18 - 10.46 ) | 1.37 ( 0.09 ) | 1.34 ( 0.24 ) | 0.42 ( -1.75 ) |
| Rash Vesicular | 1 | 0.1 ( 0.01 - 0.68 ) | 0.1 ( 8.52 ) | 0.1 ( 0.02 ) | -3.32 ( -5.37 ) |
| Acid Base Balance | 1 | 17.78 ( 1.11 - 284.29 ) | 17.78 ( 7.92 ) | 9.39 ( 0.92 ) | 3.23 ( 0.6 ) |
| Blood Ph Decreased | 1 | 0.81 ( 0.11 - 6 ) | 0.81 ( 0.04 ) | 0.82 ( 0.15 ) | -0.29 ( -2.42 ) |
| Lung Consolidation | 1 | 4.44 ( 0.5 - 39.77 ) | 4.44 ( 2.14 ) | 3.76 ( 0.6 ) | 1.91 ( -0.45 ) |
| Respiratory Acidosis | 1 | 5.93 ( 0.62 - 56.98 ) | 5.93 ( 3.07 ) | 4.69 ( 0.71 ) | 2.23 ( -0.18 ) |
| Blister | 1 | 0.12 ( 0.02 - 0.83 ) | 0.12 ( 6.63 ) | 0.12 ( 0.02 ) | -3.03 ( -5.08 ) |
| Injection Site Joint Swelling | 1 | 8.89 ( 0.81 - 98.05 ) | 8.89 ( 4.67 ) | 6.26 ( 0.84 ) | 2.65 ( 0.15 ) |
| Brain Injury | 1 | 0.63 ( 0.09 - 4.67 ) | 0.63 ( 0.2 ) | 0.65 ( 0.12 ) | -0.63 ( -2.73 ) |
| Skin Fissures | 1 | 2.22 ( 0.28 - 17.77 ) | 2.22 ( 0.6 ) | 2.09 ( 0.37 ) | 1.06 ( -1.17 ) |
| Periorbital Swelling | 1 | 0.57 ( 0.08 - 4.2 ) | 0.57 ( 0.31 ) | 0.59 ( 0.11 ) | -0.77 ( -2.87 ) |
| Excessive Eye Blinking | 1 | 0.85 ( 0.11 - 6.29 ) | 0.85 ( 0.03 ) | 0.85 ( 0.16 ) | -0.23 ( -2.36 ) |
| Epigastric Discomfort | 1 | 17.78 ( 1.11 - 284.29 ) | 17.78 ( 7.92 ) | 9.39 ( 0.92 ) | 3.23 ( 0.6 ) |
| Influenza Virus Test Positive | 1 | 2.22 ( 0.28 - 17.77 ) | 2.22 ( 0.6 ) | 2.09 ( 0.37 ) | 1.06 ( -1.17 ) |
| Eye Pruritus | 1 | 2.22 ( 0.28 - 17.77 ) | 2.22 ( 0.6 ) | 2.09 ( 0.37 ) | 1.06 ( -1.17 ) |
| Feeling Of Despair | 1 | 17.78 ( 1.11 - 284.29 ) | 17.78 ( 7.92 ) | 9.39 ( 0.92 ) | 3.23 ( 0.6 ) |
| Rectal Swab | 1 | 17.78 ( 1.11 - 284.29 ) | 17.78 ( 7.92 ) | 9.39 ( 0.92 ) | 3.23 ( 0.6 ) |
| Faeces Pale | 1 | 1.78 ( 0.23 - 13.89 ) | 1.78 ( 0.31 ) | 1.71 ( 0.31 ) | 0.77 ( -1.43 ) |
| Bronchitis Chronic | 1 | 8.89 ( 0.81 - 98.05 ) | 8.89 ( 4.67 ) | 6.26 ( 0.84 ) | 2.65 ( 0.15 ) |
| Upper Respiratory Tract Inflammation | 1 | 1.78 ( 0.23 - 13.89 ) | 1.78 ( 0.31 ) | 1.71 ( 0.31 ) | 0.77 ( -1.43 ) |
| Tardive Dyskinesia | 1 | 1.62 ( 0.21 - 12.52 ) | 1.62 ( 0.22 ) | 1.56 ( 0.28 ) | 0.65 ( -1.55 ) |
| Gastrointestinal Sounds Abnormal | 1 | 2.96 ( 0.36 - 24.62 ) | 2.96 ( 1.11 ) | 2.68 ( 0.46 ) | 1.42 ( -0.86 ) |
| Laryngospasm | 1 | 1.62 ( 0.21 - 12.52 ) | 1.62 ( 0.22 ) | 1.56 ( 0.28 ) | 0.65 ( -1.55 ) |
| Naevus Flammeus | 1 | 17.78 ( 1.11 - 284.29 ) | 17.78 ( 7.92 ) | 9.39 ( 0.92 ) | 3.23 ( 0.6 ) |
| Sinusitis | 1 | 1.11 ( 0.15 - 8.38 ) | 1.11 ( 0.01 ) | 1.1 ( 0.2 ) | 0.14 ( -2.01 ) |
| Injection Site Infection | 1 | 0.81 ( 0.11 - 6 ) | 0.81 ( 0.04 ) | 0.82 ( 0.15 ) | -0.29 ( -2.42 ) |
| Brief Resolved Unexplained Event | 1 | 1.48 ( 0.19 - 11.4 ) | 1.48 ( 0.14 ) | 1.44 ( 0.26 ) | 0.53 ( -1.65 ) |
| Hypoxic-Ischaemic Encephalopathy | 1 | 2.22 ( 0.28 - 17.77 ) | 2.22 ( 0.6 ) | 2.09 ( 0.37 ) | 1.06 ( -1.17 ) |
| Injection Site Abscess Sterile | 1 | 2.54 ( 0.31 - 20.65 ) | 2.54 ( 0.82 ) | 2.35 ( 0.41 ) | 1.23 ( -1.02 ) |
| Injection Site Cellulitis | 1 | 0.17 ( 0.02 - 1.2 ) | 0.17 ( 4.1 ) | 0.18 ( 0.03 ) | -2.51 ( -4.57 ) |
| Brain Oedema | 1 | 0.77 ( 0.1 - 5.72 ) | 0.77 ( 0.06 ) | 0.78 ( 0.15 ) | -0.35 ( -2.47 ) |
| Opsoclonus Myoclonus | 1 | 5.93 ( 0.62 - 56.98 ) | 5.93 ( 3.07 ) | 4.69 ( 0.71 ) | 2.23 ( -0.18 ) |
| Subdural Effusion | 1 | 17.78 ( 1.11 - 284.29 ) | 17.78 ( 7.92 ) | 9.39 ( 0.92 ) | 3.23 ( 0.6 ) |
| Bell's Palsy | 1 | 4.44 ( 0.5 - 39.77 ) | 4.44 ( 2.14 ) | 3.76 ( 0.6 ) | 1.91 ( -0.45 ) |
| Hypermetropia | 1 | 3.56 ( 0.42 - 30.44 ) | 3.56 ( 1.53 ) | 3.13 ( 0.52 ) | 1.65 ( -0.67 ) |
| Localised Oedema | 1 | 0.99 ( 0.13 - 7.4 ) | 0.99 ( 0 ) | 0.99 ( 0.18 ) | -0.02 ( -2.16 ) |
| Appendix Disorder | 1 | 17.78 ( 1.11 - 284.29 ) | 17.78 ( 7.92 ) | 9.39 ( 0.92 ) | 3.23 ( 0.6 ) |
| Necrosis | 1 | 4.44 ( 0.5 - 39.77 ) | 4.44 ( 2.14 ) | 3.76 ( 0.6 ) | 1.91 ( -0.45 ) |
| General Physical Condition Abnormal | 1 | 1.11 ( 0.15 - 8.38 ) | 1.11 ( 0.01 ) | 1.1 ( 0.2 ) | 0.14 ( -2.01 ) |
| Hypoaesthesia | 1 | 1.19 ( 0.16 - 8.97 ) | 1.19 ( 0.03 ) | 1.17 ( 0.22 ) | 0.23 ( -1.93 ) |
| Lung Opacity | 1 | 1.62 ( 0.21 - 12.52 ) | 1.62 ( 0.22 ) | 1.56 ( 0.28 ) | 0.65 ( -1.55 ) |
| Injection Site Scar | 1 | 2.54 ( 0.31 - 20.65 ) | 2.54 ( 0.82 ) | 2.35 ( 0.41 ) | 1.23 ( -1.02 ) |
| Bronchopulmonary Dysplasia | 1 | 17.78 ( 1.11 - 284.29 ) | 17.78 ( 7.92 ) | 9.39 ( 0.92 ) | 3.23 ( 0.6 ) |
| Intraventricular Haemorrhage | 1 | 17.78 ( 1.11 - 284.29 ) | 17.78 ( 7.92 ) | 9.39 ( 0.92 ) | 3.23 ( 0.6 ) |
| Dependent Personality Disorder | 1 | 8.89 ( 0.81 - 98.05 ) | 8.89 ( 4.67 ) | 6.26 ( 0.84 ) | 2.65 ( 0.15 ) |
| Drug Screen Negative | 1 | 2.96 ( 0.36 - 24.62 ) | 2.96 ( 1.11 ) | 2.68 ( 0.46 ) | 1.42 ( -0.86 ) |
| Urinary Incontinence | 1 | 1.78 ( 0.23 - 13.89 ) | 1.78 ( 0.31 ) | 1.71 ( 0.31 ) | 0.77 ( -1.43 ) |
| Blood Smear Test Abnormal | 1 | 2.22 ( 0.28 - 17.77 ) | 2.22 ( 0.6 ) | 2.09 ( 0.37 ) | 1.06 ( -1.17 ) |
| Red Cell Distribution Width | 1 | 1.27 ( 0.17 - 9.66 ) | 1.27 ( 0.05 ) | 1.25 ( 0.23 ) | 0.32 ( -1.84 ) |
| Poliomyelitis | 1 | 17.78 ( 1.11 - 284.29 ) | 17.78 ( 7.92 ) | 9.39 ( 0.92 ) | 3.23 ( 0.6 ) |
| White Blood Cells Urine Negative | 1 | 0.71 ( 0.1 - 5.25 ) | 0.71 ( 0.11 ) | 0.72 ( 0.14 ) | -0.47 ( -2.58 ) |
| Nitrite Urine Absent | 1 | 0.63 ( 0.09 - 4.67 ) | 0.63 ( 0.2 ) | 0.65 ( 0.12 ) | -0.63 ( -2.73 ) |
| Pharyngeal Disorder | 1 | 8.89 ( 0.81 - 98.05 ) | 8.89 ( 4.67 ) | 6.26 ( 0.84 ) | 2.65 ( 0.15 ) |
| Cognitive Disorder | 1 | 0.85 ( 0.11 - 6.29 ) | 0.85 ( 0.03 ) | 0.85 ( 0.16 ) | -0.23 ( -2.36 ) |
| Neurodevelopmental Delay | 1 | 17.78 ( 1.11 - 284.29 ) | 17.78 ( 7.92 ) | 9.39 ( 0.92 ) | 3.23 ( 0.6 ) |
| Bradykinesia | 1 | 8.89 ( 0.81 - 98.05 ) | 8.89 ( 4.67 ) | 6.26 ( 0.84 ) | 2.65 ( 0.15 ) |
| Increased Bronchial Secretion | 1 | 5.93 ( 0.62 - 56.98 ) | 5.93 ( 3.07 ) | 4.69 ( 0.71 ) | 2.23 ( -0.18 ) |
| Eczema Infantile | 1 | 4.44 ( 0.5 - 39.77 ) | 4.44 ( 2.14 ) | 3.76 ( 0.6 ) | 1.91 ( -0.45 ) |
| Specific Gravity Urine Decreased | 1 | 5.93 ( 0.62 - 56.98 ) | 5.93 ( 3.07 ) | 4.69 ( 0.71 ) | 2.23 ( -0.18 ) |
| Hydronephrosis | 1 | 17.78 ( 1.11 - 284.29 ) | 17.78 ( 7.92 ) | 9.39 ( 0.92 ) | 3.23 ( 0.6 ) |
| Plateletcrit Increased | 1 | 17.78 ( 1.11 - 284.29 ) | 17.78 ( 7.92 ) | 9.39 ( 0.92 ) | 3.23 ( 0.6 ) |
| Basophil Count Decreased | 1 | 0.54 ( 0.07 - 3.94 ) | 0.54 ( 0.38 ) | 0.55 ( 0.1 ) | -0.86 ( -2.95 ) |
| Plateletcrit | 1 | 1.27 ( 0.17 - 9.66 ) | 1.27 ( 0.05 ) | 1.25 ( 0.23 ) | 0.32 ( -1.84 ) |
| Lymph Node Pain | 1 | 1.48 ( 0.19 - 11.4 ) | 1.48 ( 0.14 ) | 1.44 ( 0.26 ) | 0.53 ( -1.65 ) |
| Rales | 1 | 1.19 ( 0.16 - 8.97 ) | 1.19 ( 0.03 ) | 1.17 ( 0.22 ) | 0.23 ( -1.93 ) |
| Tonsillar Hypertrophy | 1 | 0.51 ( 0.07 - 3.71 ) | 0.51 ( 0.46 ) | 0.52 ( 0.1 ) | -0.94 ( -3.03 ) |
| Vaccination Site Nodule | 1 | 0.66 ( 0.09 - 4.85 ) | 0.66 ( 0.17 ) | 0.67 ( 0.13 ) | -0.58 ( -2.69 ) |
| Nasal Inflammation | 1 | 4.44 ( 0.5 - 39.77 ) | 4.44 ( 2.14 ) | 3.76 ( 0.6 ) | 1.91 ( -0.45 ) |
| Lymph Node Abscess | 1 | 8.89 ( 0.81 - 98.05 ) | 8.89 ( 4.67 ) | 6.26 ( 0.84 ) | 2.65 ( 0.15 ) |
| Staphylococcus Test Positive | 1 | 1.19 ( 0.16 - 8.97 ) | 1.19 ( 0.03 ) | 1.17 ( 0.22 ) | 0.23 ( -1.93 ) |
| Idiopathic Intracranial Hypertension | 1 | 8.89 ( 0.81 - 98.05 ) | 8.89 ( 4.67 ) | 6.26 ( 0.84 ) | 2.65 ( 0.15 ) |
| Salmonella Test Negative | 1 | 1.05 ( 0.14 - 7.86 ) | 1.05 ( 0 ) | 1.04 ( 0.19 ) | 0.06 ( -2.08 ) |
| Helicobacter Test Negative | 1 | 2.54 ( 0.31 - 20.65 ) | 2.54 ( 0.82 ) | 2.35 ( 0.41 ) | 1.23 ( -1.02 ) |
| Yersinia Test Negative | 1 | 5.93 ( 0.62 - 56.98 ) | 5.93 ( 3.07 ) | 4.69 ( 0.71 ) | 2.23 ( -0.18 ) |
| Anuria | 1 | 3.56 ( 0.42 - 30.44 ) | 3.56 ( 1.53 ) | 3.13 ( 0.52 ) | 1.65 ( -0.67 ) |
| Troponin Increased | 1 | 3.56 ( 0.42 - 30.44 ) | 3.56 ( 1.53 ) | 3.13 ( 0.52 ) | 1.65 ( -0.67 ) |
| Nuclear Magnetic Resonance Imaging Abnormal | 1 | 1.98 ( 0.25 - 15.59 ) | 1.98 ( 0.43 ) | 1.88 ( 0.33 ) | 0.91 ( -1.31 ) |
| Abdominal Lymphadenopathy | 1 | 4.44 ( 0.5 - 39.77 ) | 4.44 ( 2.14 ) | 3.76 ( 0.6 ) | 1.91 ( -0.45 ) |
| Chest Pain | 1 | 1.48 ( 0.19 - 11.4 ) | 1.48 ( 0.14 ) | 1.44 ( 0.26 ) | 0.53 ( -1.65 ) |
| Poor Sucking Reflex | 1 | 0.74 ( 0.1 - 5.48 ) | 0.74 ( 0.09 ) | 0.75 ( 0.14 ) | -0.41 ( -2.53 ) |
| Thirst Decreased | 1 | 1.37 ( 0.18 - 10.46 ) | 1.37 ( 0.09 ) | 1.34 ( 0.24 ) | 0.42 ( -1.75 ) |
| Exaggerated Startle Response | 1 | 5.93 ( 0.62 - 56.98 ) | 5.93 ( 3.07 ) | 4.69 ( 0.71 ) | 2.23 ( -0.18 ) |
| Sputum Discoloured | 1 | 5.93 ( 0.62 - 56.98 ) | 5.93 ( 3.07 ) | 4.69 ( 0.71 ) | 2.23 ( -0.18 ) |
| Seizure Cluster | 1 | 2.54 ( 0.31 - 20.65 ) | 2.54 ( 0.82 ) | 2.35 ( 0.41 ) | 1.23 ( -1.02 ) |
| Quality Of Life Decreased | 1 | 8.89 ( 0.81 - 98.05 ) | 8.89 ( 4.67 ) | 6.26 ( 0.84 ) | 2.65 ( 0.15 ) |
| Injection Site Plaque | 1 | 5.93 ( 0.62 - 56.98 ) | 5.93 ( 3.07 ) | 4.69 ( 0.71 ) | 2.23 ( -0.18 ) |
| Blood Albumin Decreased | 1 | 0.52 ( 0.07 - 3.82 ) | 0.52 ( 0.42 ) | 0.54 ( 0.1 ) | -0.9 ( -2.99 ) |
| Cardiac Monitoring Abnormal | 1 | 5.93 ( 0.62 - 56.98 ) | 5.93 ( 3.07 ) | 4.69 ( 0.71 ) | 2.23 ( -0.18 ) |
| Cardio-Respiratory Arrest | 1 | 0.36 ( 0.05 - 2.63 ) | 0.36 ( 1.1 ) | 0.38 ( 0.07 ) | -1.41 ( -3.49 ) |
| Ventricular Fibrillation | 1 | 17.78 ( 1.11 - 284.29 ) | 17.78 ( 7.92 ) | 9.39 ( 0.92 ) | 3.23 ( 0.6 ) |
| Oxygen Saturation | 1 | 2.54 ( 0.31 - 20.65 ) | 2.54 ( 0.82 ) | 2.35 ( 0.41 ) | 1.23 ( -1.02 ) |
| Application Site Erythema | 1 | 8.89 ( 0.81 - 98.05 ) | 8.89 ( 4.67 ) | 6.26 ( 0.84 ) | 2.65 ( 0.15 ) |
| Puncture Site Oedema | 1 | 17.78 ( 1.11 - 284.29 ) | 17.78 ( 7.92 ) | 9.39 ( 0.92 ) | 3.23 ( 0.6 ) |
| Stridor | 1 | 0.71 ( 0.1 - 5.25 ) | 0.71 ( 0.11 ) | 0.72 ( 0.14 ) | -0.47 ( -2.58 ) |
| Throat Tightness | 1 | 2.22 ( 0.28 - 17.77 ) | 2.22 ( 0.6 ) | 2.09 ( 0.37 ) | 1.06 ( -1.17 ) |
| Clumsiness | 1 | 2.22 ( 0.28 - 17.77 ) | 2.22 ( 0.6 ) | 2.09 ( 0.37 ) | 1.06 ( -1.17 ) |
| Vasculitic Rash | 1 | 8.89 ( 0.81 - 98.05 ) | 8.89 ( 4.67 ) | 6.26 ( 0.84 ) | 2.65 ( 0.15 ) |
| Torticollis | 1 | 2.96 ( 0.36 - 24.62 ) | 2.96 ( 1.11 ) | 2.68 ( 0.46 ) | 1.42 ( -0.86 ) |
| Cytogenetic Analysis Abnormal | 1 | 1.62 ( 0.21 - 12.52 ) | 1.62 ( 0.22 ) | 1.56 ( 0.28 ) | 0.65 ( -1.55 ) |
| Hemiparesis | 1 | 0.77 ( 0.1 - 5.72 ) | 0.77 ( 0.06 ) | 0.78 ( 0.15 ) | -0.35 ( -2.47 ) |
| Neurological Examination Abnormal | 1 | 1.37 ( 0.18 - 10.46 ) | 1.37 ( 0.09 ) | 1.34 ( 0.24 ) | 0.42 ( -1.75 ) |
| Peripheral Circulatory Failure | 1 | 8.89 ( 0.81 - 98.05 ) | 8.89 ( 4.67 ) | 6.26 ( 0.84 ) | 2.65 ( 0.15 ) |
| Type I Hypersensitivity | 1 | 2.22 ( 0.28 - 17.77 ) | 2.22 ( 0.6 ) | 2.09 ( 0.37 ) | 1.06 ( -1.17 ) |
| Amnesia | 1 | 8.89 ( 0.81 - 98.05 ) | 8.89 ( 4.67 ) | 6.26 ( 0.84 ) | 2.65 ( 0.15 ) |
| Lymphocytosis | 1 | 2.22 ( 0.28 - 17.77 ) | 2.22 ( 0.6 ) | 2.09 ( 0.37 ) | 1.06 ( -1.17 ) |
| Stomatitis | 1 | 0.63 ( 0.09 - 4.67 ) | 0.63 ( 0.2 ) | 0.65 ( 0.12 ) | -0.63 ( -2.73 ) |
| Cardiomegaly | 1 | 2.54 ( 0.31 - 20.65 ) | 2.54 ( 0.82 ) | 2.35 ( 0.41 ) | 1.23 ( -1.02 ) |
| N-Terminal Prohormone Brain Natriuretic Peptide Increased | 1 | 5.93 ( 0.62 - 56.98 ) | 5.93 ( 3.07 ) | 4.69 ( 0.71 ) | 2.23 ( -0.18 ) |
| Haemolytic Uraemic Syndrome | 1 | 1.78 ( 0.23 - 13.89 ) | 1.78 ( 0.31 ) | 1.71 ( 0.31 ) | 0.77 ( -1.43 ) |
| Renal Impairment | 1 | 3.56 ( 0.42 - 30.44 ) | 3.56 ( 1.53 ) | 3.13 ( 0.52 ) | 1.65 ( -0.67 ) |
| Growth Retardation | 1 | 1.78 ( 0.23 - 13.89 ) | 1.78 ( 0.31 ) | 1.71 ( 0.31 ) | 0.77 ( -1.43 ) |
| Exanthema Subitum | 1 | 0.94 ( 0.13 - 6.99 ) | 0.94 ( 0 ) | 0.94 ( 0.17 ) | -0.09 ( -2.23 ) |
| Oral Herpes | 1 | 0.94 ( 0.13 - 6.99 ) | 0.94 ( 0 ) | 0.94 ( 0.17 ) | -0.09 ( -2.23 ) |
| Otitis Media Acute | 1 | 0.47 ( 0.06 - 3.41 ) | 0.47 ( 0.59 ) | 0.48 ( 0.09 ) | -1.05 ( -3.15 ) |
| Yellow Skin | 1 | 2.22 ( 0.28 - 17.77 ) | 2.22 ( 0.6 ) | 2.09 ( 0.37 ) | 1.06 ( -1.17 ) |
| Red Blood Cell Microcytes Present | 1 | 8.89 ( 0.81 - 98.05 ) | 8.89 ( 4.67 ) | 6.26 ( 0.84 ) | 2.65 ( 0.15 ) |
| Binocular Eye Movement Disorder | 1 | 17.78 ( 1.11 - 284.29 ) | 17.78 ( 7.92 ) | 9.39 ( 0.92 ) | 3.23 ( 0.6 ) |
| Blood Calcium Decreased | 1 | 1.05 ( 0.14 - 7.86 ) | 1.05 ( 0 ) | 1.04 ( 0.19 ) | 0.06 ( -2.08 ) |
| Muscular Dystrophy | 1 | 17.78 ( 1.11 - 284.29 ) | 17.78 ( 7.92 ) | 9.39 ( 0.92 ) | 3.23 ( 0.6 ) |
| Red Blood Cell Anisocytes Present | 1 | 17.78 ( 1.11 - 284.29 ) | 17.78 ( 7.92 ) | 9.39 ( 0.92 ) | 3.23 ( 0.6 ) |
| Red Blood Cell Macrocytes Present | 1 | 17.78 ( 1.11 - 284.29 ) | 17.78 ( 7.92 ) | 9.39 ( 0.92 ) | 3.23 ( 0.6 ) |
| Bowel Movement Irregularity | 1 | 1.78 ( 0.23 - 13.89 ) | 1.78 ( 0.31 ) | 1.71 ( 0.31 ) | 0.77 ( -1.43 ) |
| Amylase Decreased | 1 | 3.56 ( 0.42 - 30.44 ) | 3.56 ( 1.53 ) | 3.13 ( 0.52 ) | 1.65 ( -0.67 ) |
| Blood Fibrinogen Increased | 1 | 1.98 ( 0.25 - 15.59 ) | 1.98 ( 0.43 ) | 1.88 ( 0.33 ) | 0.91 ( -1.31 ) |
| Base Excess Decreased | 1 | 1.27 ( 0.17 - 9.66 ) | 1.27 ( 0.05 ) | 1.25 ( 0.23 ) | 0.32 ( -1.84 ) |
| Hyporeflexia | 1 | 3.56 ( 0.42 - 30.44 ) | 3.56 ( 1.53 ) | 3.13 ( 0.52 ) | 1.65 ( -0.67 ) |
| Neuropathy Peripheral | 1 | 2.54 ( 0.31 - 20.65 ) | 2.54 ( 0.82 ) | 2.35 ( 0.41 ) | 1.23 ( -1.02 ) |
| Guillain-Barre Syndrome | 1 | 0.68 ( 0.09 - 5.04 ) | 0.68 ( 0.14 ) | 0.7 ( 0.13 ) | -0.52 ( -2.64 ) |
| Retinal Haemorrhage | 1 | 17.78 ( 1.11 - 284.29 ) | 17.78 ( 7.92 ) | 9.39 ( 0.92 ) | 3.23 ( 0.6 ) |
| Ultrasound Skull Abnormal | 1 | 2.22 ( 0.28 - 17.77 ) | 2.22 ( 0.6 ) | 2.09 ( 0.37 ) | 1.06 ( -1.17 ) |
| Nuclear Magnetic Resonance Imaging Brain Abnormal | 1 | 0.94 ( 0.13 - 6.99 ) | 0.94 ( 0 ) | 0.94 ( 0.17 ) | -0.09 ( -2.23 ) |
| Intracranial Pressure Increased | 1 | 8.89 ( 0.81 - 98.05 ) | 8.89 ( 4.67 ) | 6.26 ( 0.84 ) | 2.65 ( 0.15 ) |
| Ophthalmological Examination Abnormal | 1 | 3.56 ( 0.42 - 30.44 ) | 3.56 ( 1.53 ) | 3.13 ( 0.52 ) | 1.65 ( -0.67 ) |
| Subdural Hygroma | 1 | 8.89 ( 0.81 - 98.05 ) | 8.89 ( 4.67 ) | 6.26 ( 0.84 ) | 2.65 ( 0.15 ) |
| Dermatitis Exfoliative Generalised | 1 | 8.89 ( 0.81 - 98.05 ) | 8.89 ( 4.67 ) | 6.26 ( 0.84 ) | 2.65 ( 0.15 ) |
| Culture Throat Negative | 1 | 1.48 ( 0.19 - 11.4 ) | 1.48 ( 0.14 ) | 1.44 ( 0.26 ) | 0.53 ( -1.65 ) |
| Csf Culture Negative | 1 | 0.39 ( 0.05 - 2.8 ) | 0.39 ( 0.95 ) | 0.4 ( 0.08 ) | -1.32 ( -3.41 ) |
| Motor Dysfunction | 1 | 0.74 ( 0.1 - 5.48 ) | 0.74 ( 0.09 ) | 0.75 ( 0.14 ) | -0.41 ( -2.53 ) |
| Nervous System Disorder | 1 | 0.47 ( 0.06 - 3.41 ) | 0.47 ( 0.59 ) | 0.48 ( 0.09 ) | -1.05 ( -3.15 ) |
| Psychomotor Retardation | 1 | 5.93 ( 0.62 - 56.98 ) | 5.93 ( 3.07 ) | 4.69 ( 0.71 ) | 2.23 ( -0.18 ) |
| Polyarthritis | 1 | 4.44 ( 0.5 - 39.77 ) | 4.44 ( 2.14 ) | 3.76 ( 0.6 ) | 1.91 ( -0.45 ) |
| Cytomegalovirus Infection | 1 | 0.99 ( 0.13 - 7.4 ) | 0.99 ( 0 ) | 0.99 ( 0.18 ) | -0.02 ( -2.16 ) |
| Oral Disorder | 1 | 1.62 ( 0.21 - 12.52 ) | 1.62 ( 0.22 ) | 1.56 ( 0.28 ) | 0.65 ( -1.55 ) |
| Immunodeficiency | 1 | 0.77 ( 0.1 - 5.72 ) | 0.77 ( 0.06 ) | 0.78 ( 0.15 ) | -0.35 ( -2.47 ) |
| Anger | 1 | 0.94 ( 0.13 - 6.99 ) | 0.94 ( 0 ) | 0.94 ( 0.17 ) | -0.09 ( -2.23 ) |
| Appetite Disorder | 1 | 1.11 ( 0.15 - 8.38 ) | 1.11 ( 0.01 ) | 1.1 ( 0.2 ) | 0.14 ( -2.01 ) |
| Capillary Nail Refill Test | 1 | 1.48 ( 0.19 - 11.4 ) | 1.48 ( 0.14 ) | 1.44 ( 0.26 ) | 0.53 ( -1.65 ) |
| Body Temperature Fluctuation | 1 | 2.96 ( 0.36 - 24.62 ) | 2.96 ( 1.11 ) | 2.68 ( 0.46 ) | 1.42 ( -0.86 ) |
| Face Oedema | 1 | 1.19 ( 0.16 - 8.97 ) | 1.19 ( 0.03 ) | 1.17 ( 0.22 ) | 0.23 ( -1.93 ) |
| Pneumonia Aspiration | 1 | 5.93 ( 0.62 - 56.98 ) | 5.93 ( 3.07 ) | 4.69 ( 0.71 ) | 2.23 ( -0.18 ) |
| Generalised Erythema | 1 | 2.22 ( 0.28 - 17.77 ) | 2.22 ( 0.6 ) | 2.09 ( 0.37 ) | 1.06 ( -1.17 ) |
| Fungal Skin Infection | 1 | 5.93 ( 0.62 - 56.98 ) | 5.93 ( 3.07 ) | 4.69 ( 0.71 ) | 2.23 ( -0.18 ) |
| Gastrointestinal Motility Disorder | 1 | 5.93 ( 0.62 - 56.98 ) | 5.93 ( 3.07 ) | 4.69 ( 0.71 ) | 2.23 ( -0.18 ) |
| Reflux Gastritis | 1 | 17.78 ( 1.11 - 284.29 ) | 17.78 ( 7.92 ) | 9.39 ( 0.92 ) | 3.23 ( 0.6 ) |
| Cerebral Disorder | 1 | 1.62 ( 0.21 - 12.52 ) | 1.62 ( 0.22 ) | 1.56 ( 0.28 ) | 0.65 ( -1.55 ) |
| Oral Candidiasis | 1 | 1.98 ( 0.25 - 15.59 ) | 1.98 ( 0.43 ) | 1.88 ( 0.33 ) | 0.91 ( -1.31 ) |
| Sandifer's Syndrome | 1 | 8.89 ( 0.81 - 98.05 ) | 8.89 ( 4.67 ) | 6.26 ( 0.84 ) | 2.65 ( 0.15 ) |
| Photophobia | 1 | 0.71 ( 0.1 - 5.25 ) | 0.71 ( 0.11 ) | 0.72 ( 0.14 ) | -0.47 ( -2.58 ) |
| Irritable Bowel Syndrome | 1 | 3.56 ( 0.42 - 30.44 ) | 3.56 ( 1.53 ) | 3.13 ( 0.52 ) | 1.65 ( -0.67 ) |
| Allergy To Metals | 1 | 0.89 ( 0.12 - 6.62 ) | 0.89 ( 0.01 ) | 0.89 ( 0.17 ) | -0.16 ( -2.29 ) |
| Oral Mucosal Eruption | 1 | 0.81 ( 0.11 - 6 ) | 0.81 ( 0.04 ) | 0.82 ( 0.15 ) | -0.29 ( -2.42 ) |
| Reaction To Excipient | 1 | 1.19 ( 0.16 - 8.97 ) | 1.19 ( 0.03 ) | 1.17 ( 0.22 ) | 0.23 ( -1.93 ) |
| Wound | 1 | 1.19 ( 0.16 - 8.97 ) | 1.19 ( 0.03 ) | 1.17 ( 0.22 ) | 0.23 ( -1.93 ) |
| Fungal Infection | 1 | 2.22 ( 0.28 - 17.77 ) | 2.22 ( 0.6 ) | 2.09 ( 0.37 ) | 1.06 ( -1.17 ) |
| Skin Test Positive | 1 | 0.99 ( 0.13 - 7.4 ) | 0.99 ( 0 ) | 0.99 ( 0.18 ) | -0.02 ( -2.16 ) |
| Atelectasis | 1 | 3.56 ( 0.42 - 30.44 ) | 3.56 ( 1.53 ) | 3.13 ( 0.52 ) | 1.65 ( -0.67 ) |
| Blood Creatinine Increased | 1 | 0.94 ( 0.13 - 6.99 ) | 0.94 ( 0 ) | 0.94 ( 0.17 ) | -0.09 ( -2.23 ) |
| Haemolysis | 1 | 2.54 ( 0.31 - 20.65 ) | 2.54 ( 0.82 ) | 2.35 ( 0.41 ) | 1.23 ( -1.02 ) |
| Weight Gain Poor | 1 | 0.71 ( 0.1 - 5.25 ) | 0.71 ( 0.11 ) | 0.72 ( 0.14 ) | -0.47 ( -2.58 ) |
| Crepitations | 1 | 3.56 ( 0.42 - 30.44 ) | 3.56 ( 1.53 ) | 3.13 ( 0.52 ) | 1.65 ( -0.67 ) |
| Failure To Thrive | 1 | 0.81 ( 0.11 - 6 ) | 0.81 ( 0.04 ) | 0.82 ( 0.15 ) | -0.29 ( -2.42 ) |
| Interstitial Lung Disease | 1 | 2.22 ( 0.28 - 17.77 ) | 2.22 ( 0.6 ) | 2.09 ( 0.37 ) | 1.06 ( -1.17 ) |
| Use Of Accessory Respiratory Muscles | 1 | 0.89 ( 0.12 - 6.62 ) | 0.89 ( 0.01 ) | 0.89 ( 0.17 ) | -0.16 ( -2.29 ) |
| Nasal Flaring | 1 | 8.89 ( 0.81 - 98.05 ) | 8.89 ( 4.67 ) | 6.26 ( 0.84 ) | 2.65 ( 0.15 ) |
| Vaccination Site Dryness | 1 | 17.78 ( 1.11 - 284.29 ) | 17.78 ( 7.92 ) | 9.39 ( 0.92 ) | 3.23 ( 0.6 ) |
| Vaccination Site Papule | 1 | 5.93 ( 0.62 - 56.98 ) | 5.93 ( 3.07 ) | 4.69 ( 0.71 ) | 2.23 ( -0.18 ) |
| Secretion Discharge | 1 | 0.44 ( 0.06 - 3.23 ) | 0.44 ( 0.68 ) | 0.46 ( 0.09 ) | -1.13 ( -3.22 ) |
| Skin Wound | 1 | 2.54 ( 0.31 - 20.65 ) | 2.54 ( 0.82 ) | 2.35 ( 0.41 ) | 1.23 ( -1.02 ) |
| Postictal Paralysis | 1 | 2.54 ( 0.31 - 20.65 ) | 2.54 ( 0.82 ) | 2.35 ( 0.41 ) | 1.23 ( -1.02 ) |
| Cow's Milk Intolerance | 1 | 3.56 ( 0.42 - 30.44 ) | 3.56 ( 1.53 ) | 3.13 ( 0.52 ) | 1.65 ( -0.67 ) |
| Bordetella Test Positive | 1 | 4.44 ( 0.5 - 39.77 ) | 4.44 ( 2.14 ) | 3.76 ( 0.6 ) | 1.91 ( -0.45 ) |
| Breathing-Related Sleep Disorder | 1 | 8.89 ( 0.81 - 98.05 ) | 8.89 ( 4.67 ) | 6.26 ( 0.84 ) | 2.65 ( 0.15 ) |
| Glomerular Filtration Rate Decreased | 1 | 17.78 ( 1.11 - 284.29 ) | 17.78 ( 7.92 ) | 9.39 ( 0.92 ) | 3.23 ( 0.6 ) |
| Antithrombin Iii | 1 | 17.78 ( 1.11 - 284.29 ) | 17.78 ( 7.92 ) | 9.39 ( 0.92 ) | 3.23 ( 0.6 ) |
| Hallucination | 1 | 1.98 ( 0.25 - 15.59 ) | 1.98 ( 0.43 ) | 1.88 ( 0.33 ) | 0.91 ( -1.31 ) |
| Blood Fibrinogen | 1 | 2.96 ( 0.36 - 24.62 ) | 2.96 ( 1.11 ) | 2.68 ( 0.46 ) | 1.42 ( -0.86 ) |
| Cerebral Venous Sinus Thrombosis | 1 | 8.89 ( 0.81 - 98.05 ) | 8.89 ( 4.67 ) | 6.26 ( 0.84 ) | 2.65 ( 0.15 ) |
| Dry Mouth | 1 | 4.44 ( 0.5 - 39.77 ) | 4.44 ( 2.14 ) | 3.76 ( 0.6 ) | 1.91 ( -0.45 ) |
| Magnetic Resonance Imaging Brain Abnormal | 1 | 0.81 ( 0.11 - 6 ) | 0.81 ( 0.04 ) | 0.82 ( 0.15 ) | -0.29 ( -2.42 ) |
| Mucosal Dryness | 1 | 4.44 ( 0.5 - 39.77 ) | 4.44 ( 2.14 ) | 3.76 ( 0.6 ) | 1.91 ( -0.45 ) |
| Protein Urine | 1 | 4.44 ( 0.5 - 39.77 ) | 4.44 ( 2.14 ) | 3.76 ( 0.6 ) | 1.91 ( -0.45 ) |
| Streptococcus Test Positive | 1 | 0.36 ( 0.05 - 2.57 ) | 0.36 ( 1.15 ) | 0.37 ( 0.07 ) | -1.44 ( -3.52 ) |
| Mean Cell Haemoglobin | 1 | 2.22 ( 0.28 - 17.77 ) | 2.22 ( 0.6 ) | 2.09 ( 0.37 ) | 1.06 ( -1.17 ) |
| Prothrombin Time Shortened | 1 | 2.54 ( 0.31 - 20.65 ) | 2.54 ( 0.82 ) | 2.35 ( 0.41 ) | 1.23 ( -1.02 ) |
| Urine Ketone Body | 1 | 5.93 ( 0.62 - 56.98 ) | 5.93 ( 3.07 ) | 4.69 ( 0.71 ) | 2.23 ( -0.18 ) |
| Confusional State | 1 | 0.46 ( 0.06 - 3.32 ) | 0.46 ( 0.63 ) | 0.47 ( 0.09 ) | -1.09 ( -3.18 ) |
| Protrusion Tongue | 1 | 1.37 ( 0.18 - 10.46 ) | 1.37 ( 0.09 ) | 1.34 ( 0.24 ) | 0.42 ( -1.75 ) |
| Carbon Dioxide Decreased | 1 | 0.66 ( 0.09 - 4.85 ) | 0.66 ( 0.17 ) | 0.67 ( 0.13 ) | -0.58 ( -2.69 ) |
| Blood Urea Decreased | 1 | 0.81 ( 0.11 - 6 ) | 0.81 ( 0.04 ) | 0.82 ( 0.15 ) | -0.29 ( -2.42 ) |
| Haemorrhage Subcutaneous | 1 | 0.34 ( 0.05 - 2.43 ) | 0.34 ( 1.29 ) | 0.35 ( 0.07 ) | -1.52 ( -3.6 ) |
| Mouth Haemorrhage | 1 | 1.05 ( 0.14 - 7.86 ) | 1.05 ( 0 ) | 1.04 ( 0.19 ) | 0.06 ( -2.08 ) |
| Peripheral Vascular Disorder | 1 | 5.93 ( 0.62 - 56.98 ) | 5.93 ( 3.07 ) | 4.69 ( 0.71 ) | 2.23 ( -0.18 ) |
| Activated Partial Thromboplastin Time Prolonged | 1 | 1.62 ( 0.21 - 12.52 ) | 1.62 ( 0.22 ) | 1.56 ( 0.28 ) | 0.65 ( -1.55 ) |
| Prothrombin Time Prolonged | 1 | 1.48 ( 0.19 - 11.4 ) | 1.48 ( 0.14 ) | 1.44 ( 0.26 ) | 0.53 ( -1.65 ) |
| White Blood Cells Urine Positive | 1 | 0.68 ( 0.09 - 5.04 ) | 0.68 ( 0.14 ) | 0.7 ( 0.13 ) | -0.52 ( -2.64 ) |
| Culture Urine Positive | 1 | 0.89 ( 0.12 - 6.62 ) | 0.89 ( 0.01 ) | 0.89 ( 0.17 ) | -0.16 ( -2.29 ) |
| Hepatic Enzyme Increased | 1 | 0.77 ( 0.1 - 5.72 ) | 0.77 ( 0.06 ) | 0.78 ( 0.15 ) | -0.35 ( -2.47 ) |
| Lung Infiltration | 1 | 1.37 ( 0.18 - 10.46 ) | 1.37 ( 0.09 ) | 1.34 ( 0.24 ) | 0.42 ( -1.75 ) |
| Lip Erythema | 1 | 0.89 ( 0.12 - 6.62 ) | 0.89 ( 0.01 ) | 0.89 ( 0.17 ) | -0.16 ( -2.29 ) |
| Osteomyelitis | 1 | 1.19 ( 0.16 - 8.97 ) | 1.19 ( 0.03 ) | 1.17 ( 0.22 ) | 0.23 ( -1.93 ) |
| Pharyngeal Swelling | 1 | 3.56 ( 0.42 - 30.44 ) | 3.56 ( 1.53 ) | 3.13 ( 0.52 ) | 1.65 ( -0.67 ) |
| Eosinophilia | 1 | 1.78 ( 0.23 - 13.89 ) | 1.78 ( 0.31 ) | 1.71 ( 0.31 ) | 0.77 ( -1.43 ) |
| Viral Upper Respiratory Tract Infection | 1 | 1.11 ( 0.15 - 8.38 ) | 1.11 ( 0.01 ) | 1.1 ( 0.2 ) | 0.14 ( -2.01 ) |
| Familial Mediterranean Fever | 1 | 17.78 ( 1.11 - 284.29 ) | 17.78 ( 7.92 ) | 9.39 ( 0.92 ) | 3.23 ( 0.6 ) |
| Vitamin B12 Decreased | 1 | 17.78 ( 1.11 - 284.29 ) | 17.78 ( 7.92 ) | 9.39 ( 0.92 ) | 3.23 ( 0.6 ) |
| Gene Mutation Identification Test Positive | 1 | 1.05 ( 0.14 - 7.86 ) | 1.05 ( 0 ) | 1.04 ( 0.19 ) | 0.06 ( -2.08 ) |
| Decreased Interest | 1 | 1.19 ( 0.16 - 8.97 ) | 1.19 ( 0.03 ) | 1.17 ( 0.22 ) | 0.23 ( -1.93 ) |
| Hypoglycaemia | 1 | 1.05 ( 0.14 - 7.86 ) | 1.05 ( 0 ) | 1.04 ( 0.19 ) | 0.06 ( -2.08 ) |
| Vaccination Site Infection | 1 | 4.44 ( 0.5 - 39.77 ) | 4.44 ( 2.14 ) | 3.76 ( 0.6 ) | 1.91 ( -0.45 ) |
| Ear Swelling | 1 | 1.11 ( 0.15 - 8.38 ) | 1.11 ( 0.01 ) | 1.1 ( 0.2 ) | 0.14 ( -2.01 ) |
| Haemorrhage Intracranial | 1 | 4.44 ( 0.5 - 39.77 ) | 4.44 ( 2.14 ) | 3.76 ( 0.6 ) | 1.91 ( -0.45 ) |
| Inflammatory Bowel Disease | 1 | 5.93 ( 0.62 - 56.98 ) | 5.93 ( 3.07 ) | 4.69 ( 0.71 ) | 2.23 ( -0.18 ) |
| Arrhythmia | 1 | 1.98 ( 0.25 - 15.59 ) | 1.98 ( 0.43 ) | 1.88 ( 0.33 ) | 0.91 ( -1.31 ) |
| Heart Rate Irregular | 1 | 8.89 ( 0.81 - 98.05 ) | 8.89 ( 4.67 ) | 6.26 ( 0.84 ) | 2.65 ( 0.15 ) |
| Oropharyngeal Pain | 1 | 0.33 ( 0.05 - 2.38 ) | 0.33 ( 1.34 ) | 0.34 ( 0.07 ) | -1.55 ( -3.63 ) |
| Sudden Onset Of Sleep | 1 | 17.78 ( 1.11 - 284.29 ) | 17.78 ( 7.92 ) | 9.39 ( 0.92 ) | 3.23 ( 0.6 ) |
| Epileptic Encephalopathy | 1 | 4.44 ( 0.5 - 39.77 ) | 4.44 ( 2.14 ) | 3.76 ( 0.6 ) | 1.91 ( -0.45 ) |
| Ocular Hyperaemia | 1 | 0.28 ( 0.04 - 2.03 ) | 0.28 ( 1.8 ) | 0.29 ( 0.06 ) | -1.77 ( -3.84 ) |
| Tongue Movement Disturbance | 1 | 5.93 ( 0.62 - 56.98 ) | 5.93 ( 3.07 ) | 4.69 ( 0.71 ) | 2.23 ( -0.18 ) |
| Butterfly Rash | 1 | 17.78 ( 1.11 - 284.29 ) | 17.78 ( 7.92 ) | 9.39 ( 0.92 ) | 3.23 ( 0.6 ) |
| Vaccination Site Joint Pain | 1 | 8.89 ( 0.81 - 98.05 ) | 8.89 ( 4.67 ) | 6.26 ( 0.84 ) | 2.65 ( 0.15 ) |
| Vaccination Site Joint Erythema | 1 | 8.89 ( 0.81 - 98.05 ) | 8.89 ( 4.67 ) | 6.26 ( 0.84 ) | 2.65 ( 0.15 ) |
| Abdominal Pain Lower | 1 | 2.54 ( 0.31 - 20.65 ) | 2.54 ( 0.82 ) | 2.35 ( 0.41 ) | 1.23 ( -1.02 ) |
| Oral Administration Complication | 1 | 0.59 ( 0.08 - 4.35 ) | 0.59 ( 0.27 ) | 0.61 ( 0.11 ) | -0.72 ( -2.83 ) |
| Basophil Degranulation Test | 1 | 5.93 ( 0.62 - 56.98 ) | 5.93 ( 3.07 ) | 4.69 ( 0.71 ) | 2.23 ( -0.18 ) |
| Blood Pressure Increased | 1 | 8.89 ( 0.81 - 98.05 ) | 8.89 ( 4.67 ) | 6.26 ( 0.84 ) | 2.65 ( 0.15 ) |
| Head Lag Abnormal | 1 | 17.78 ( 1.11 - 284.29 ) | 17.78 ( 7.92 ) | 9.39 ( 0.92 ) | 3.23 ( 0.6 ) |
| Mood Swings | 1 | 2.22 ( 0.28 - 17.77 ) | 2.22 ( 0.6 ) | 2.09 ( 0.37 ) | 1.06 ( -1.17 ) |
| Gastrointestinal Hypermotility | 1 | 8.89 ( 0.81 - 98.05 ) | 8.89 ( 4.67 ) | 6.26 ( 0.84 ) | 2.65 ( 0.15 ) |
| Peristalsis Visible | 1 | 17.78 ( 1.11 - 284.29 ) | 17.78 ( 7.92 ) | 9.39 ( 0.92 ) | 3.23 ( 0.6 ) |
| Intellectual Disability | 1 | 1.27 ( 0.17 - 9.66 ) | 1.27 ( 0.05 ) | 1.25 ( 0.23 ) | 0.32 ( -1.84 ) |
| Epstein-Barr Virus Test Negative | 1 | 0.74 ( 0.1 - 5.48 ) | 0.74 ( 0.09 ) | 0.75 ( 0.14 ) | -0.41 ( -2.53 ) |
| Blood Glucose Decreased | 1 | 1.37 ( 0.18 - 10.46 ) | 1.37 ( 0.09 ) | 1.34 ( 0.24 ) | 0.42 ( -1.75 ) |
| Cytomegalovirus Test Negative | 1 | 0.61 ( 0.08 - 4.5 ) | 0.61 ( 0.24 ) | 0.63 ( 0.12 ) | -0.68 ( -2.78 ) |
| Lymphocyte Percentage Decreased | 1 | 0.68 ( 0.09 - 5.04 ) | 0.68 ( 0.14 ) | 0.7 ( 0.13 ) | -0.52 ( -2.64 ) |
| Red Blood Cell Sedimentation Rate Increased | 1 | 0.29 ( 0.04 - 2.1 ) | 0.29 ( 1.7 ) | 0.3 ( 0.06 ) | -1.72 ( -3.8 ) |
| Herpes Simplex Test Negative | 1 | 0.36 ( 0.05 - 2.63 ) | 0.36 ( 1.1 ) | 0.38 ( 0.07 ) | -1.41 ( -3.49 ) |
| Rotavirus Test Negative | 1 | 0.37 ( 0.05 - 2.68 ) | 0.37 ( 1.05 ) | 0.38 ( 0.07 ) | -1.38 ( -3.46 ) |
| Activated Partial Thromboplastin Time Shortened | 1 | 2.54 ( 0.31 - 20.65 ) | 2.54 ( 0.82 ) | 2.35 ( 0.41 ) | 1.23 ( -1.02 ) |
| Basophil Count Increased | 1 | 2.22 ( 0.28 - 17.77 ) | 2.22 ( 0.6 ) | 2.09 ( 0.37 ) | 1.06 ( -1.17 ) |
| Hyperresponsive To Stimuli | 1 | 5.93 ( 0.62 - 56.98 ) | 5.93 ( 3.07 ) | 4.69 ( 0.71 ) | 2.23 ( -0.18 ) |
| Aggression | 1 | 0.27 ( 0.04 - 1.91 ) | 0.27 ( 2.01 ) | 0.28 ( 0.05 ) | -1.86 ( -3.93 ) |
| Feeling Cold | 1 | 0.94 ( 0.13 - 6.99 ) | 0.94 ( 0 ) | 0.94 ( 0.17 ) | -0.09 ( -2.23 ) |
| Ataxia | 1 | 0.48 ( 0.07 - 3.5 ) | 0.48 ( 0.55 ) | 0.49 ( 0.09 ) | -1.02 ( -3.11 ) |
| Myoclonus | 1 | 0.54 ( 0.07 - 3.94 ) | 0.54 ( 0.38 ) | 0.55 ( 0.1 ) | -0.86 ( -2.95 ) |
| Gastrointestinal Haemorrhage | 1 | 1.48 ( 0.19 - 11.4 ) | 1.48 ( 0.14 ) | 1.44 ( 0.26 ) | 0.53 ( -1.65 ) |
| Hiccups | 1 | 2.96 ( 0.36 - 24.62 ) | 2.96 ( 1.11 ) | 2.68 ( 0.46 ) | 1.42 ( -0.86 ) |
| Mean Cell Haemoglobin Concentration Increased | 1 | 2.54 ( 0.31 - 20.65 ) | 2.54 ( 0.82 ) | 2.35 ( 0.41 ) | 1.23 ( -1.02 ) |
| Mean Cell Haemoglobin Increased | 1 | 5.93 ( 0.62 - 56.98 ) | 5.93 ( 3.07 ) | 4.69 ( 0.71 ) | 2.23 ( -0.18 ) |
| Csf Cell Count Increased | 1 | 1.27 ( 0.17 - 9.66 ) | 1.27 ( 0.05 ) | 1.25 ( 0.23 ) | 0.32 ( -1.84 ) |
| Csf Culture Positive | 1 | 8.89 ( 0.81 - 98.05 ) | 8.89 ( 4.67 ) | 6.26 ( 0.84 ) | 2.65 ( 0.15 ) |
| Fear | 1 | 0.99 ( 0.13 - 7.4 ) | 0.99 ( 0 ) | 0.99 ( 0.18 ) | -0.02 ( -2.16 ) |
| Blood Glucose Abnormal | 1 | 5.93 ( 0.62 - 56.98 ) | 5.93 ( 3.07 ) | 4.69 ( 0.71 ) | 2.23 ( -0.18 ) |
| Lactic Acidosis | 1 | 4.44 ( 0.5 - 39.77 ) | 4.44 ( 2.14 ) | 3.76 ( 0.6 ) | 1.91 ( -0.45 ) |
| Systemic Inflammatory Response Syndrome | 1 | 0.71 ( 0.1 - 5.25 ) | 0.71 ( 0.11 ) | 0.72 ( 0.14 ) | -0.47 ( -2.58 ) |
| Throat Irritation | 1 | 1.37 ( 0.18 - 10.46 ) | 1.37 ( 0.09 ) | 1.34 ( 0.24 ) | 0.42 ( -1.75 ) |
| Monocyte Percentage Decreased | 1 | 5.93 ( 0.62 - 56.98 ) | 5.93 ( 3.07 ) | 4.69 ( 0.71 ) | 2.23 ( -0.18 ) |
| Microcephaly | 1 | 5.93 ( 0.62 - 56.98 ) | 5.93 ( 3.07 ) | 4.69 ( 0.71 ) | 2.23 ( -0.18 ) |
| Communication Disorder | 1 | 0.77 ( 0.1 - 5.72 ) | 0.77 ( 0.06 ) | 0.78 ( 0.15 ) | -0.35 ( -2.47 ) |
| Blood Test Abnormal | 1 | 0.25 ( 0.03 - 1.8 ) | 0.25 ( 2.21 ) | 0.26 ( 0.05 ) | -1.94 ( -4.01 ) |
| Vith Nerve Paralysis | 1 | 1.98 ( 0.25 - 15.59 ) | 1.98 ( 0.43 ) | 1.88 ( 0.33 ) | 0.91 ( -1.31 ) |
| Blood Urea | 1 | 0.77 ( 0.1 - 5.72 ) | 0.77 ( 0.06 ) | 0.78 ( 0.15 ) | -0.35 ( -2.47 ) |
| Sensory Disturbance | 1 | 1.27 ( 0.17 - 9.66 ) | 1.27 ( 0.05 ) | 1.25 ( 0.23 ) | 0.32 ( -1.84 ) |
