# Supplementary material for: Comparative safety evaluation of pentavalent (DTaP-IPV-Hib) and hexavalent (DTaP-IPV-Hib-HepB) vaccines in infants: a real-world analysis based on VAERS
Source: Front Cell Infect Microbiol. 2025 Oct 30;15:1666509. doi: 10.3389/fcimb.2025.1666509 (PMC12611864; doi:10.3389/fcimb.2025.1666509)
Supplement: Supplementary file 8 [file Table6.docx]

Table 6: Reported frequencies of AEFIs for pentavalent and hexavalent vaccines in infants aged 6 weeks to 4 months, 4 to 8 months, and 8 months to 2 years.

| Age | 6 weeks to 4 months | 4 to 8 months | 8 months to 2 years |
| --- | --- | --- | --- |
| Pentavalent vaccines | | | |
| Overall | 1519 | 708 | 1032 |
| hexavalent vaccines | | | |
| Overall | 1099 | 317 | 304 |
